# Supplementary material for: Learning in continuous action space for developing high dimensional potential energy models
Source: Nat Commun. 2022 Jan 18;13:368. doi: 10.1038/s41467-021-27849-6 (PMC8766468; doi:10.1038/s41467-021-27849-6)
Supplement: Supplementary file 1 — Supplementary Information [file 41467_2021_27849_MOESM1_ESM.pdf]

Supplementary Information for
Learning in Continuous Action Space for Developing High Dimensional Potential Energy Models

S. Manna et al.

December 10, 2021

1 Supplementary Tables

Supplementary Table 1: Performance comparison among c-MCTS, Bayesian, particle swarm, and random sampling methods to find optimal solutions in 25 different trial functions. Two performance metrics were chosen: the number of iterations to reach the target solution and the quality of the best solution obtained from the optimizer. Results up to a maximum of 30000 iterations are provided. D denotes the dimension of the trial function, while the tolerance indicates the accepted difference from the exact optimal solution of the trial function. Bold font highlight the winning solution in each row.

				c-MCTS		Bayesian		Particle Swarm		Random	
Trial Function	D	Exact Solution	Tolerance	# of Iterations	Best Solution	# of Iterations	Best Solution	# of Iterations	Best Solution	# of Iterations	Best Solution
Ackley	2	0.000	1.000	269	0 ± 0.0	**146**	**0.6±0.125**	167	0±0	687	1.198 ±0.515
Ackley	4	0.000	1.000	601	0 ± 0.0	803	2.743 ± 0.342	**507**	**0±0**	>30000	6.854 ± 3.071
Ackley	10	0.000	1.000	**601**	**0.001 ± 0.0**	>30000	6.408 ± 0.573	1372	0.0 ± 0.0	>30000	15.802 ± 1.205
Ackley	15	0.000	1.000	**601**	**0.002 ± 0.0**	>30000	10.604 ± 1.435	1814	0.635 ± 0.426	>30000	17.869 ± 0.471
Ackley	30	0.000	1.000	**601**	**0.005 ± 0.0**	>30000	16.466 ± 0.0	>30000	4.2 ± 0.847	>30000	19.777 ± 0.036
Ackley	50	0.000	1.000	**601**	**0.002 ± 0.0**	>30000	18.354 ± 0.052	15072	4.287 ± 0.625	>30000	20.304 ± 0.014
Damavandi	2	0.000	1.000	**2821**	**0.0207 ± 0.001**	>30000	2.0 ± 0.0	>30000	2.0 ± 0.0	29761	0.437 ± 0.156
Dixon Price	13	0.000	1.000	**9482**	**0.605 ± 0.043**	>30000	1.542 ± 0.0	5758	0.705 ± 0.01	>30000	20680.725 ± 64499917.399
EggHolder	2	-959.000	1.000	**13025**	**-959.364 ± 0.665**	>30000	-937.074 ± 930.271	>30000	-950.075 ± 89.247	>30000	-948.275 ± 50.051
Levy	2	0.000	1.000	7217	0.626 ± 0.068	**21**	**0.0 ± 0.0**	>30000	90.994 ± 768.539	>30000	2748.049 ± 30219.815
Schwefel	3	0.000	0.100	13277	0.029 ± 0.001	>30000	4.196 ± 451.225	**4964**	**0.0 ± 0.0**	>30000	0.124 ± 0.009
HyperRastrigin	2	0.000	5.000	115	0.013 ± 0.001	**74**	**0.41 ± 0.251**	133	0.0 ± 0.0	139	0.246 ± 0.044
HyperRastrigin	9	0.000	5.000	**5215**	**0.997 ± 0.0**	>30000	431.285 ± 338.013	>30000	116.225 ± 577.774	>30000	637.065 ± 347.21
HyperRastrigin	50	0.000	5.000	**5307**	**1.0 ± 0.0**	>30000	436.267 ± 129.964	>30000	11.356 ± 11.788	>30000	97.939 ± 62.296
Dejong	2	0.000	0.001	**821**	**0.0 ± 0.0**	>30000	1.1 ± 0.09	>30000	0.033 ± 0.032	171162	0.0 ± 0.0
Dejong	9	0.000	0.001	**1985**	**0.0 ± 0.0**	>30000	3.467 ± 0.249	>30000	12.4 ± 4.507	>30000	15.333 ± 8.089
Dejong	15	0.000	0.001	**2386**	**0.0 ± 0.0**	>30000	11.2 ± 1.56	>30000	31.733 ± 17.929	>30000	37.033 ± 25.499
Dejong	50	0.000	0.001	**4968**	**0.0 ± 0.0**	>30000	103.333 ± 66.889	>30000	158.167 ± 142.339	>30000	189.933 ± 33.796
Dropwave	2	-1.000	0.010	**2369**	**-0.998 ± 0.0**	>30000	-0.976 ± 0	2979	-1.0 ± -0.0	>30000	-0.972 ± 0.001
Nestorov	13	0.000	1.00E-07	>30000	0.21 ± 0.017	>30000	2.198 ± 0.1	>30000	0.318 ± 0.039	>30000	5.513 ± 0.353
Grammacy & Lee	1	-0.869	1.00E-07	**235**	**-0.869 ± 0**	2105	-0.869 ± 0.0	>30000	-0.869 ± 0.0	10964	-0.869 ± 0.0
HyperPlane	2	-5.120	1.000	**14**	**-5.12 ± 0.0**	23	-4.718 ± 0.113	98	-5.078 ± 0.001	60	-5.05 ± 0.001
HyperPlane	9	-5.120	1.000	1050	-5.12 ± -0.0	**404**	**-5.01 ± 0.0**	>30000	-3.629 ± 0.056	>30000	-3.701 ± 0.054
HyperPlane	15	-5.120	1.000	**1246**	**-5.114 ± 0.001**	3578	-4.682 ± 0.002	>30000	-2.909 ± 0.171	>30000	-2.925 ± 0.017
HyperPlane	50	-5.120	1.000	**2360**	**-5.102 ± 0.001**	>30000	-3.413 ± 0.0	>30000	-2.357 ± 0.043	>30000	-1.687 ± 0.006

2 Supplementary Figures

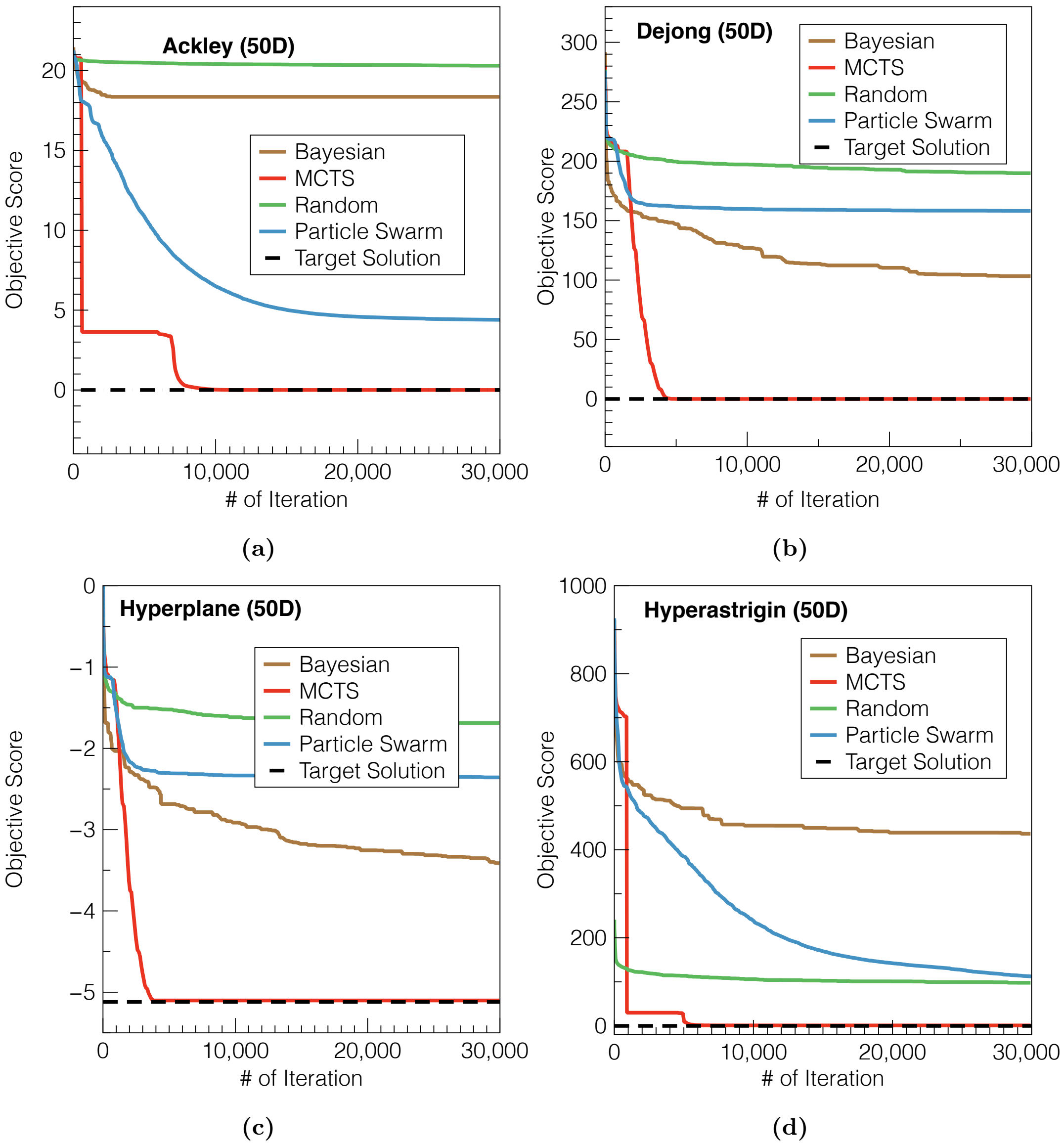

Supplementary Figure 1: Performance of c-MCTS for 50-dimensional (a) Ackley's (b) Dejong's Step (c) Hyperplane and (d) Hyperrastrigin functions. In all cases, c-MCTS outperforms other optimizers both in terms of the number of iterations to reach the solution and the quality of the best solution.

a.

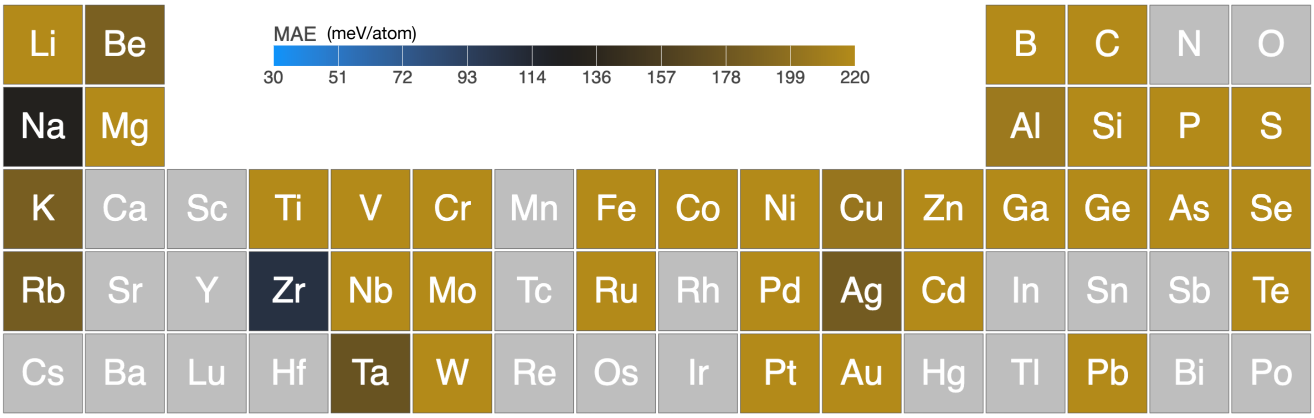

b.

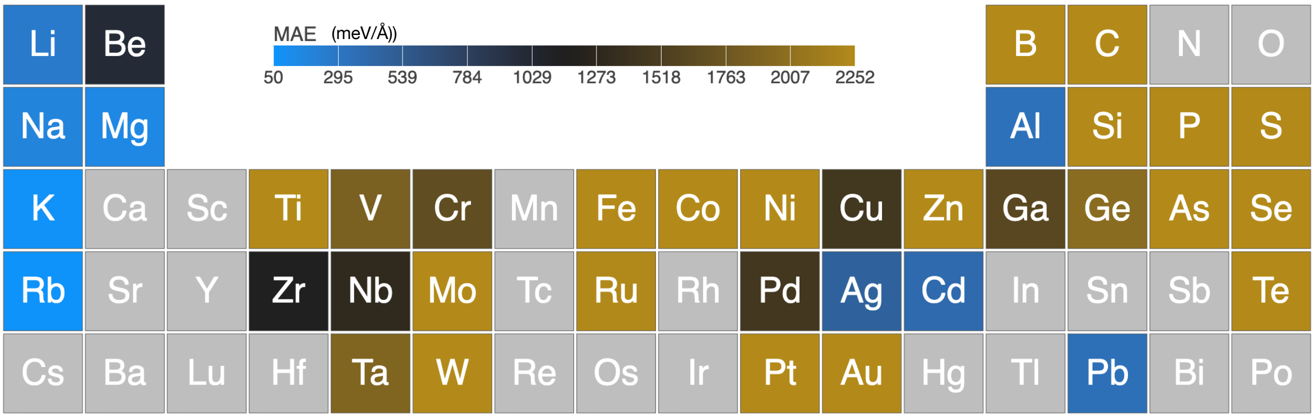

Supplementary Figure 2: Performance of the best available force fields collected from literature on predicting energies and forces of different elemental cluster configurations. For better error comparison with Figure 2 of the main manuscript, consistent error range and color scheme are used. Elements for which comparison studies were omitted are colored grey. More rigorous performance evaluations (for both energies and forces) covering a wider range of popular force fields can be found in Supplemental Note 2.

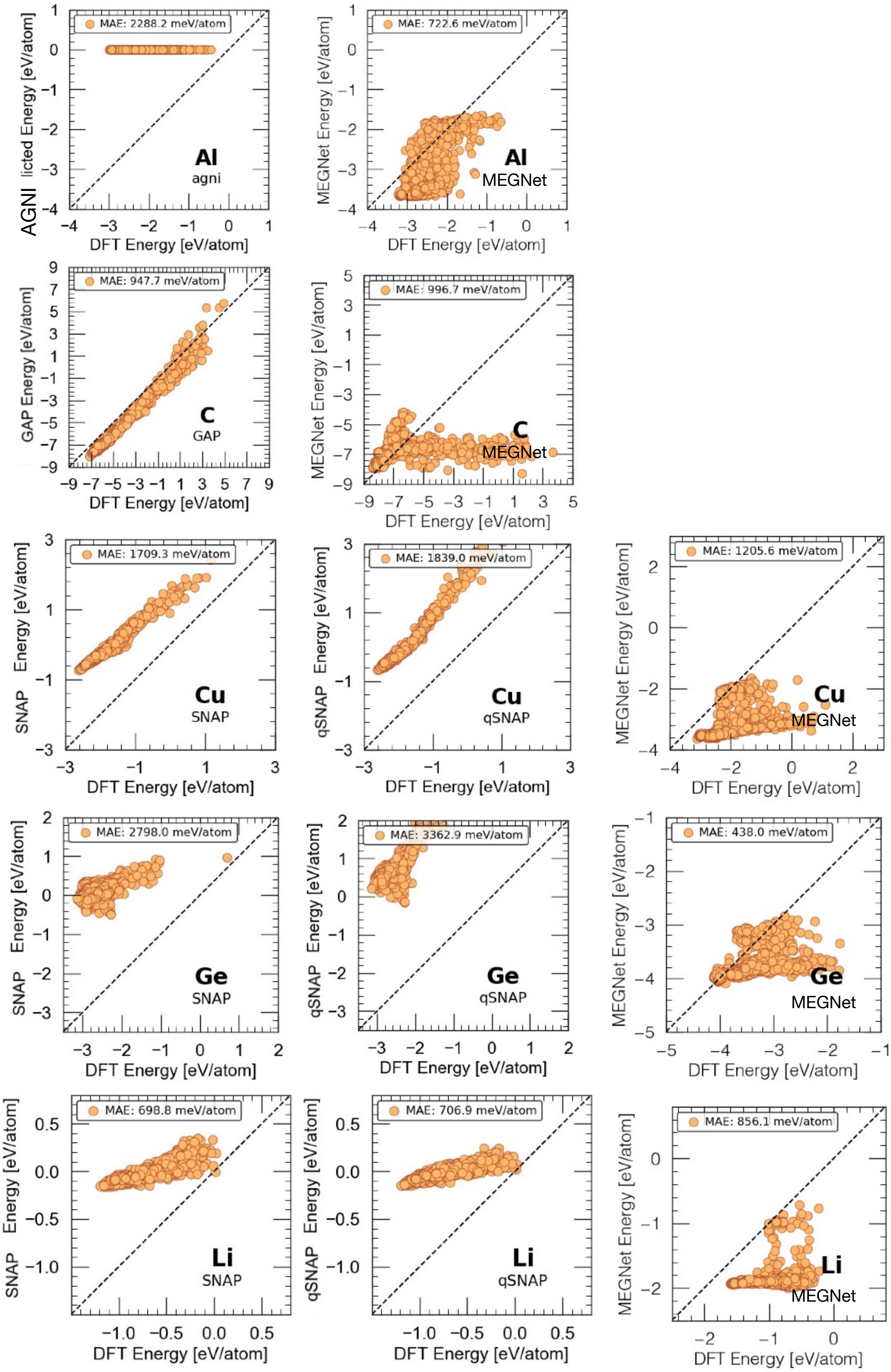

Supplementary Figure 3: Performance of state-of-the-art ML potentials on predicting energies of clusters. Mean Absolute Errors (MAE) are provided in the legend of each subplot.

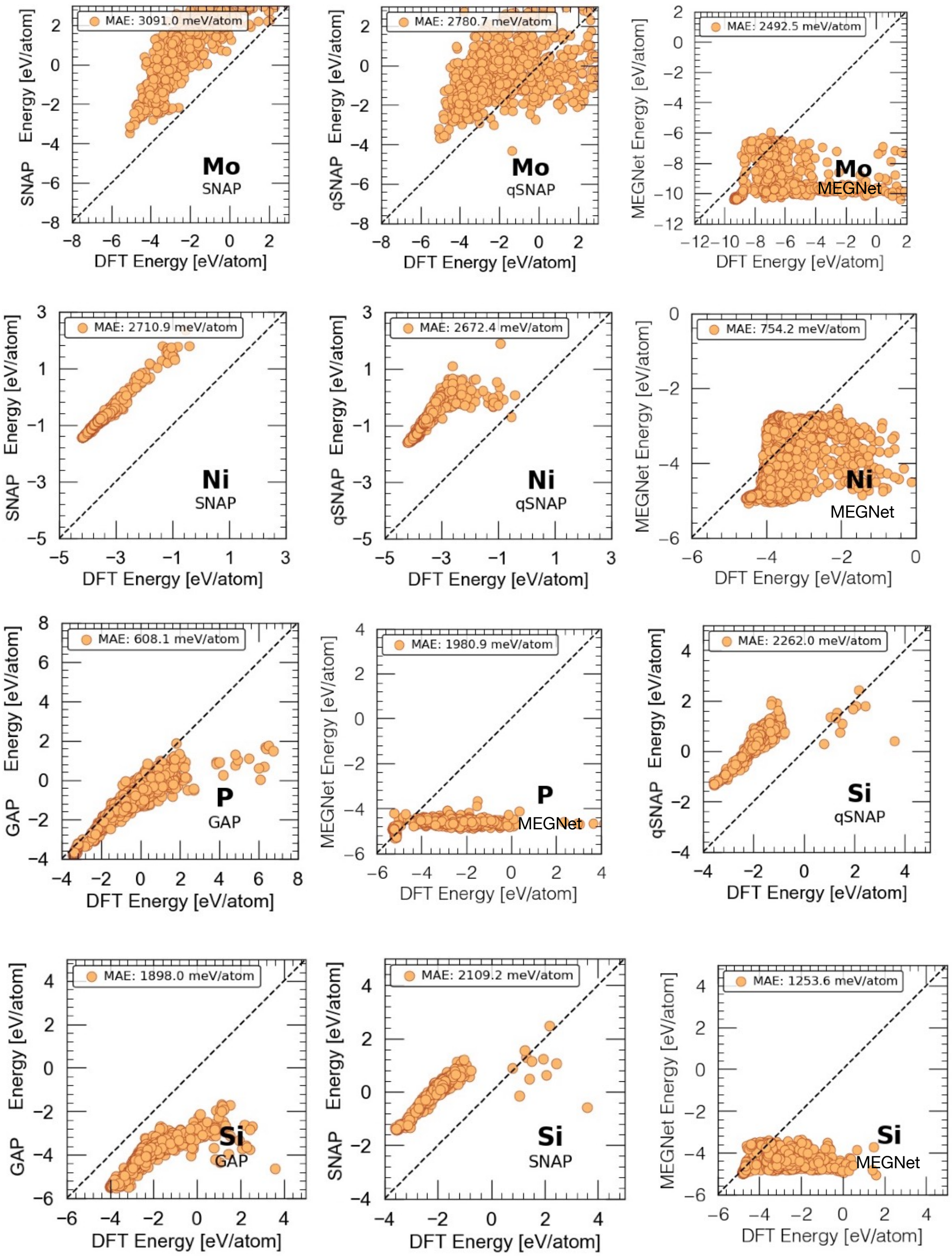

Supplementary Figure 4: Performance of state-of-the-art ML potentials on predicting energies of clusters. Mean Absolute Errors (MAE) are provided in the legend of each subplot.

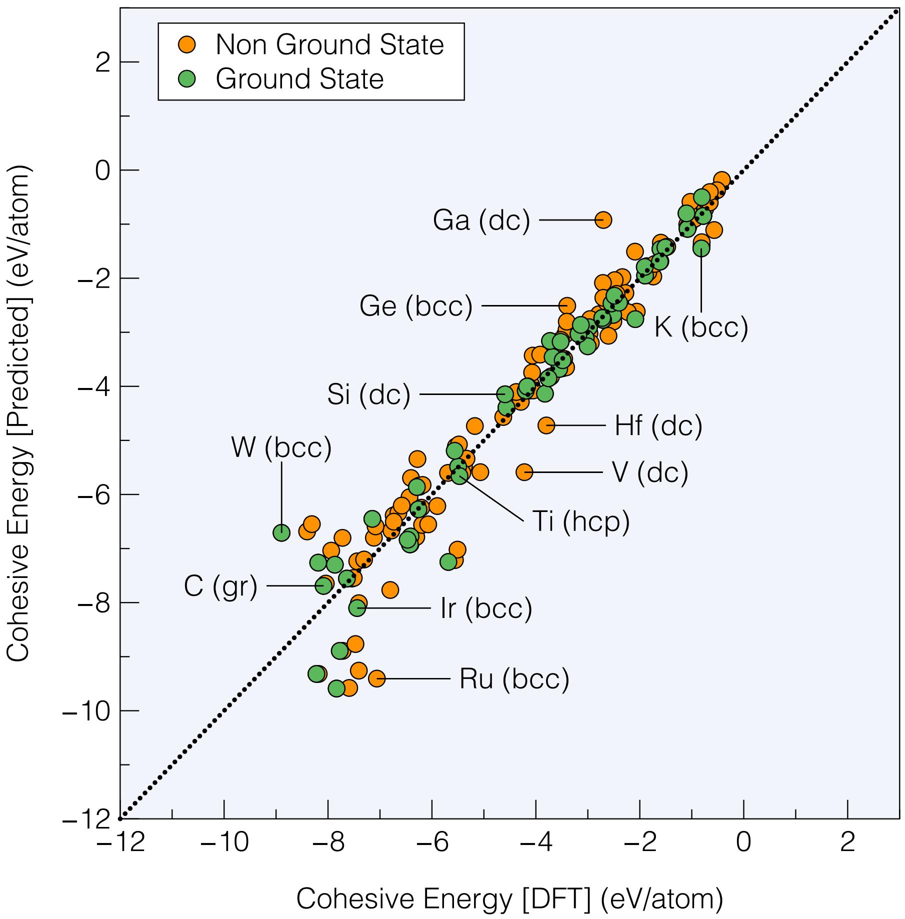

Supplementary Figure 5: Accuracy of predicted cohesive energies of the different polymorphs (including the ground state) of 54 elements from c-MCTS optimized HyBOP potentials in this study.

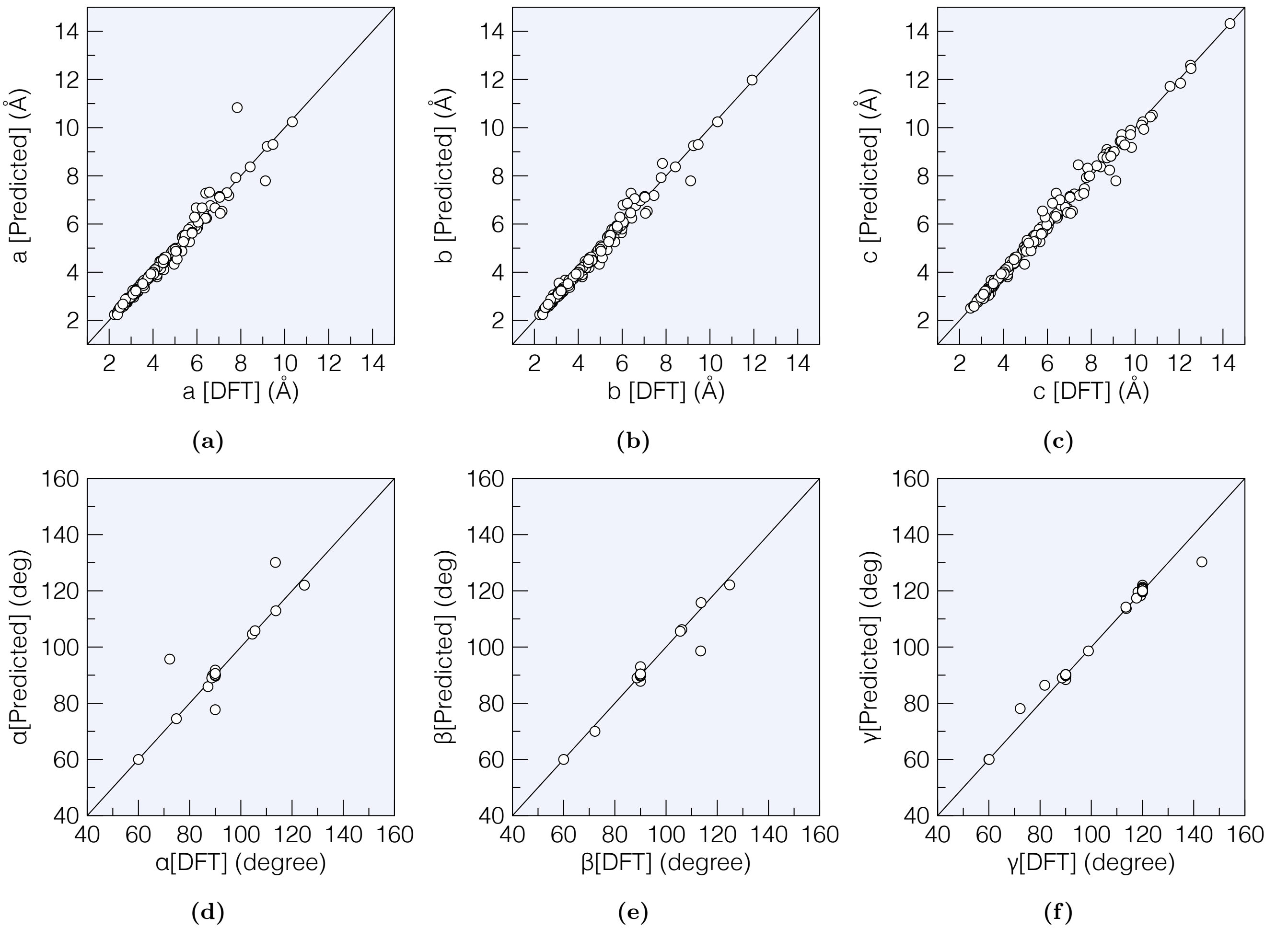

Supplementary Figure 6: Accuracy of predicted lattice parameters of the different polymorphs (including the ground state) of 54 elements from c-MCTS optimized HyBOP potentials in this study.

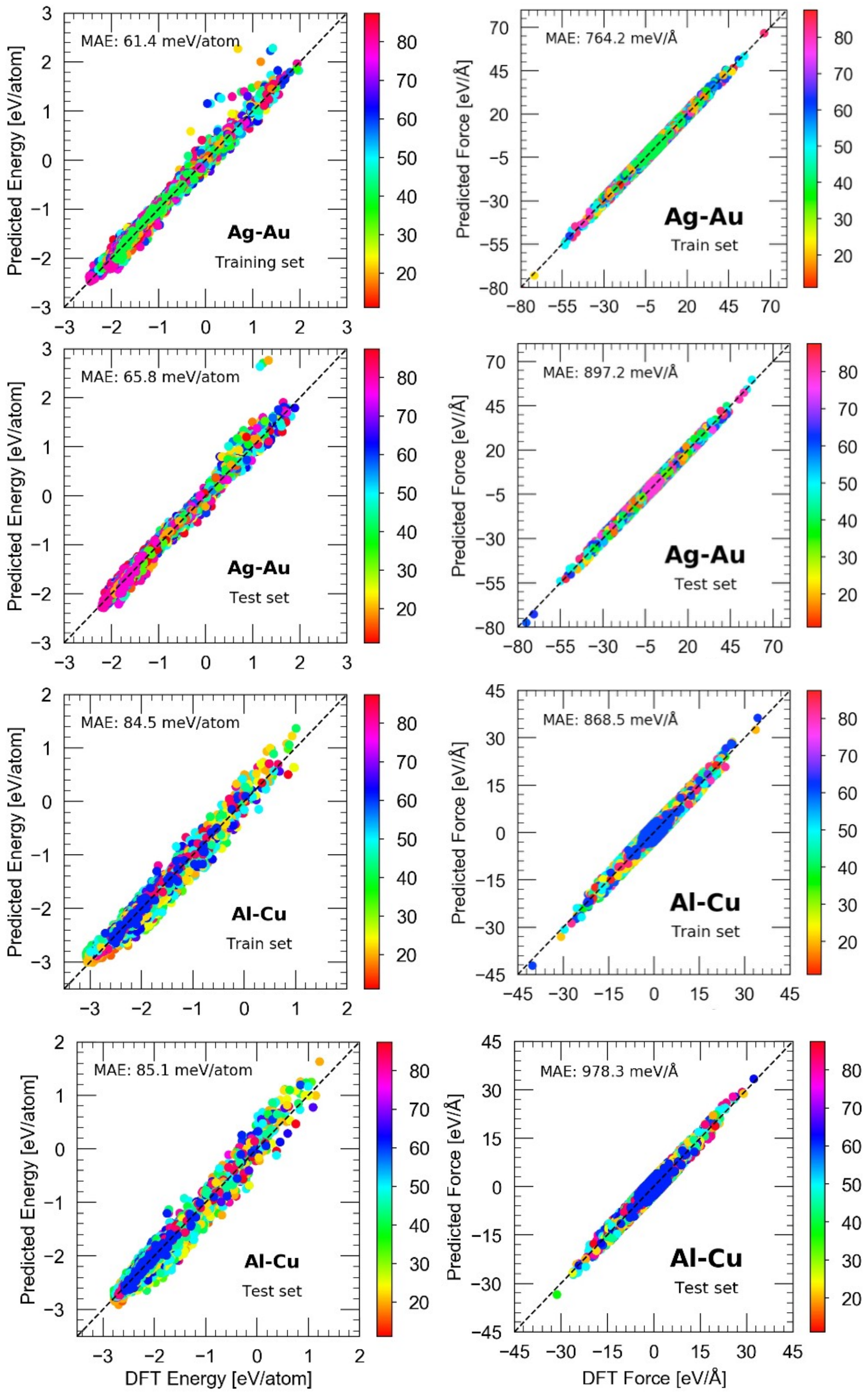

Supplementary Figure 7: Energy and force correlation plots for predicting energies and forces of Ag-Au (synthesized experimentally [1, 2]) and Al-Cu alloy. The heat map shows the concentration of Au in Ag-Au alloy and Cu in Al-Cu clusters in atomic %. The MAE for Ag-Au alloy in train and test data for predicting energies are 61.4 meV/atom and 65.8 meV/atom respectively over a 4 eV/atom energy span. In case of predicting force the MAE's are ∼ 765 meV/Å and 900 meV/Å for train and test data respectively over a span of ∼ 160 eV/Å. In case of Al-Cu alloys, the MAE in predicting energies ∼ 85 meV/atom for train and test data over a span of ∼4 eV/atom energy span. The MAE in predicting forces are ∼ 868 meV/Å and ∼ 978 meV/Å respectively. This suggests that the developed RL technique is not only limited to predicting PES of elemental systems but can also be easily extended to alloy systems (both ordered and disordered solid solution systems) over a wide compositional range.

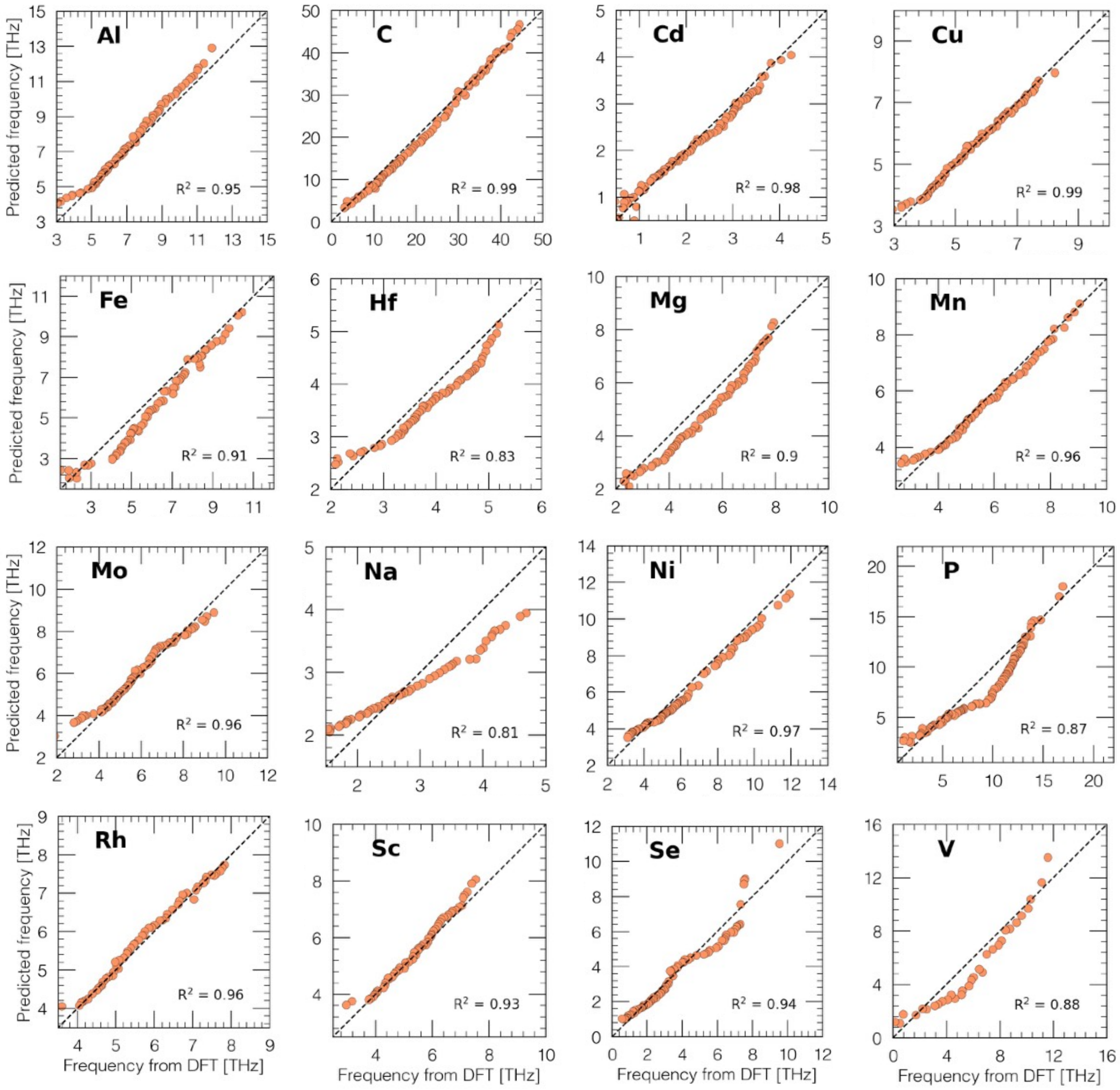

Supplementary Figure 8: Normal Modes analysis. The normal modes using the c-MCTS trained force fields for at least one representative element from each group of the Periodic table and compared them against those from DFT computations. The computational methodology is discussed Supplementary Note 7. The agreement is quite remarkable ($R^2 > 0.8$) considering the diversity of the cluster configurations sampled in this work.

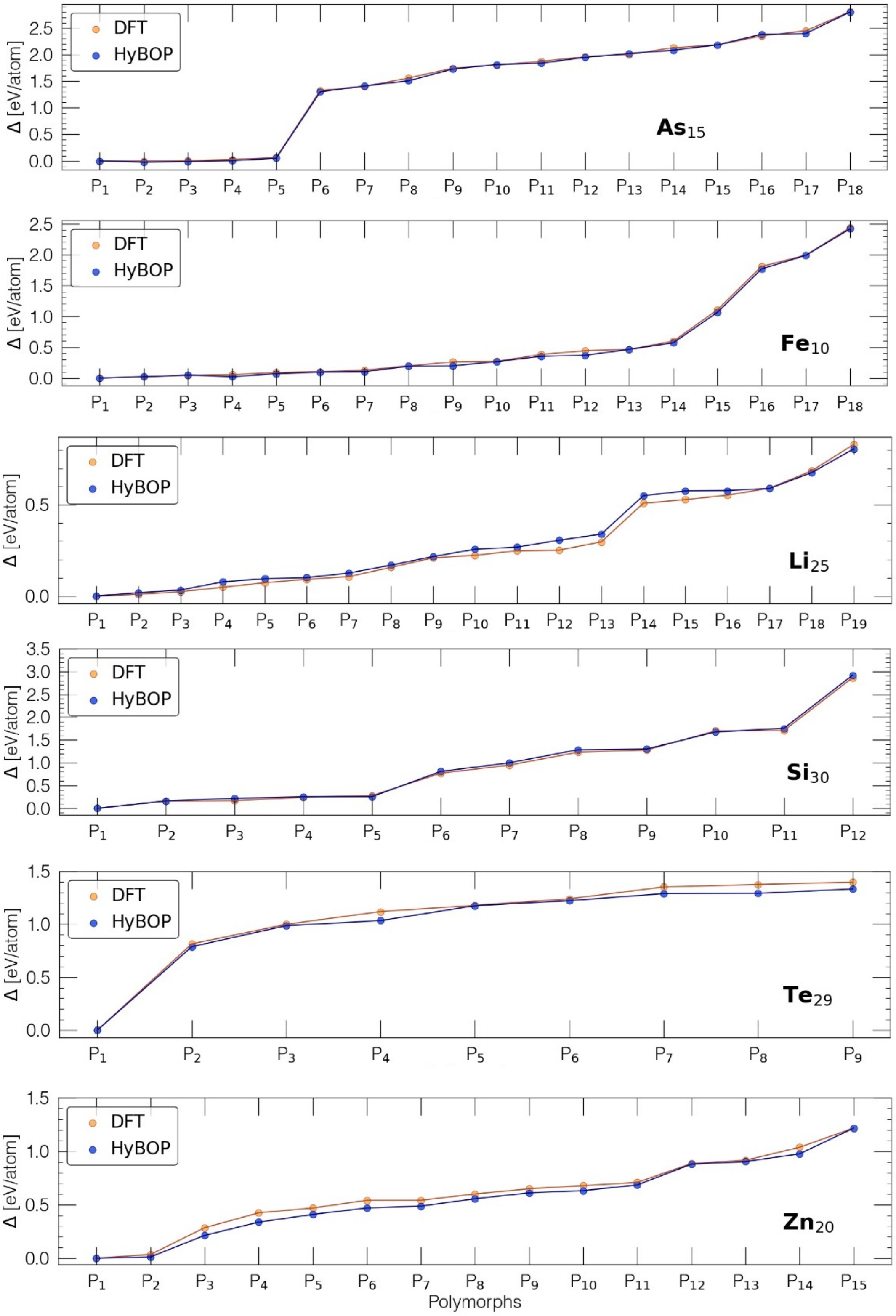

Supplementary Figure 9: Relative stability analysis. Representative plots for As_{15}, Fe_{10}, Li_{25}, Si_{30}, Te_{29}, and Zn_{20} clusters and their relative energy ordering from DFT as well as our developed force field using c-MCTS. Additional plots and other details can be found in Supplementary Note 7.

3 Supplementary Methods

3.1 Monte Carlo Tree Search in a Continuous Action Space

Monte Carlo tress search (MCTS) has emerged as a powerful global optimization method that has found wide-spread applications in discrete search problems that include computer games such as Alpha Go, games such as Bridge, Poker and many other video games [3]. MCTS is a probabilistic and heuristic search algorithm that integrates a tree search algorithm with machine learning principles of reinforcement learning [4]. Being originally designed for board games, the core idea behind the traditional MCTS algorithm is to create a decision tree that corresponds to a sequence of potential moves a player can make. Traditionally this decision tree was explored via more direct algorithms such as the classical minimax algorithm which would hope to search a big enough portion of the action tree and determine which path had the highest reward behind it, but when the search space grew very large these direct methods would run into problems related to computational efficiency, memory, etc. that would ultimately force the algorithm to work on a much smaller subset of information that was required to make a properly informed decision. As such exploring the tree became a problem of making the best decision using the spare information about the action space. The way the MCTS algorithm would perform is via four steps: (1) Selection, (2) Expansion, (3) Simulation, and (4) Back Propagation. The first of these, the selection step, is performed by rank ordering the nodes according to a selection rule, traditionally some variant of the Upper Confidence Bound (UCB) selection rule, and picking the node with the highest score. The selection rule typically has an exploit component which gives a higher weight to a node with the best win/loss ratio at the moment and an explore component which gives a high weight to nodes that have not been explored as aggressively. Once selected, the expansion step creates a child node by performing a valid move (IE in chess moving say a pawn up a space) to transform the parent's game state into a child game state. From this new state, a simulation is performed by randomly generating moves from the child node's game state until a win or loss state is achieved. This is also known as a playout since it is a play till the end of the game is reached. Once completed, the number of wins and losses are tallied and sent back up the tree. This is known as back propagation in which the reward information of a given child node is returned back to all of the parent nodes in a given lineage. The algorithm can then choose based on which path results in the greatest reward, in the case of board games the move that results in the best chance of winning. The c-MCTS uses a lot of similar ideas to traditional MCTS, but modifies each of the constituent parts in order to adapt it to problems that are both non-discrete and without a clear "win" condition. First the definition of a node instead of being a game state, represents a point within the global search space. Each node acts as an anchor point that is the center of a hyper-sphere. The hyper-sphere being a neighborhood of points located within a fixed radius of the node's parameter set. In a c-MCTS algorithm the decision tree represents a refinement of the total search space. (The details of how these hyper-spheres are used to converge to a solution will be discussed in the following sections). Likewise the idea of a playout in this space is instead defined as a random sample within the neighborhood of a given node.

The algorithm simultaneously explores potentially better pathways to reach the optimal point in a search space and exploits a single pathway that has the greatest estimate value of the search function. This combination of exploration vs. exploitation and an appropriate trade-off mechanism between them are found to be the most efficient strategy of identifying optimal point for a given function. An advantage of the MCTS is that if the search gets trapped in a metastable or suboptimal point, it can quickly find another pathway by growing other branches of the tree utilizing the trade-off mechanism between exploration and exploitation. To-date, MCTS has primarily found success in discrete action space problems.

3.1.1 Importance of Continuous Action Space Searching Strategy

MCTS is a planning and optimization technique that has shown remarkable success in search problems associated with computer games such as Go [5]. But unlike a Go game, the problems in materials application such as high-dimensional parameter search or configurational search demand techniques which can operate in continuous action space. Most of the techniques that are used to perform searches for materials design are based on local or gradient based optimization. The global optimization strategies such as evolutionary search, Bayesian or Particle Swarm tend to struggle when scaling to high dimensions and/or are sluggish in convergence to solution (as was shown by comparing several high-dimensional trial functions. We are attempting to translate the extraordinary success of MCTS in discrete action space such as Go game to continuous action space search problems, with an emphasis on materials application. MCTS is a global derivative free optimization strategy that can be applied to both continuous and discrete action space search problems. The nanoscale PES is one such application that involves parameter search

to capture the energetics and dynamics of cluster configuration over a wide degree of non-equilibrium. The method developed here can be extended to other continuous action space problems beyond materials science (Section 4.3 for more details). For instance, in case of self driving car, the steering wheel can be turned at a specific angle (could accept any values $\in$ [-720°, 720°]), or the gas pedal (usually from 0 to 1) can be pressed with different levels of force. Both of these actions are continuous (not discrete) and require to be optimized for better control when autonomously driving a car.

Below we present three important aspects to be considered when adapting MCTS for continuous action space i.e. c-MCTS.

3.2 Enhanced Exploration and Avoiding Degeneracy:

A major problem in continuous action space search problems is degeneracy i.e. it is possible for two initially separate branches to converge to the same area in the search space. This often results in the c-MCTS wasting computational cycles on degenerate solutions instead of spending time exploring solutions that it has not previously encountered. Additionally, for many physical problems of interest, there are often cases where two or more parameters can be swapped interchangeably to arrive at the same solution. As such, it is important to include information about what solutions have already been explored into the node selection to guide the algorithm toward solutions that will provide new information. In order to do this, we introduce a function that defines a "uniqueness criteria" - this is added to the exploration side of the node selection rule in order to account for degeneracies in the search space. The design of this function is to scale the exploration side down toward 0 in the event that the solutions are degenerate with what has already been discovered. In addition, when a node has a solution that is very unique or located in a region that is under-explored, the function will scale to a higher value which promotes searches in these regions. For simple parameter searches, where swap degeneracies are not an issue, we find a simple definition as depicted below:

$$f(\vec{r_i}) = \frac{1.5}{1 + \sum_{j \neq i}^{N_{points}} \delta(|r_i - r_j|)} \tag{1}$$

$$\delta(|r_i - r_j|) = \begin{cases} 1, & |r_i - r_j| < r_{max} \\ 0, & |r_i - r_j| \geq r_{max} \end{cases} \tag{2}$$

Where r_{max} is the same r_{max} as found in the window depth scaling and $|r_i - r_j|$ is the distance between sample points i and j in the reduced parameter space. This value is effectively counting the number of points found within an area and scales the uniqueness with the number of points found within the same window. Since previously sampled points do not change their position, one only has to keep a running tally of the number of points have been sampled in the same area as a given node. This means that one only has to update this function by comparing existing points to the newly added points which in practice is a very fast operation. The final node selection rule used is very similar to the classic UCT or UCB with a few key modifications which is called the Upper Confidence Bound for Parameters or UCP.

$$\text{UCP}(\theta_j) = -\min(r_1, r_2, ..., r_{n_i}) + c \cdot f(\theta_j) \cdot \sqrt{\frac{lnN_i}{n_i}} \tag{3}$$

where θ_j represents node j in the c-MCTS structure, r is the reward for a given playout, c is the exploration constant, $f(\theta_j)$ is the uniqueness criteria value for this node, i is the number of playout samples taken by this node and all of its child nodes, and N_i is a similar value as n_i except it is the parent node's playout count instead of this node's. Unlike other problems, the reward is given as the best playout reward discovered as opposed to average. This is because the algorithm is trying to find the best solution instead of the highest probability of winning like in many other MCTS formalisms. Outside of that, the formalism will be familiar to those experienced in MCTS theory.

3.2.1 Playouts with Adaptive Sampling:

In a board game setting, traditionally playouts were performed by randomly moving pieces trying to find situations in which a game ended in a victory or a loss. A challenge in a continuous action space, however, is that there isn't a clear cut "win" condition. As such the meaning of a playout must also change. Instead of wins and losses, we view playouts as a request for additional random sampling around a given point. When a node is selected for a playout, random vector displacements from the parameter set contained in the node are performed. This acts as a random

walk through the phase space that is guided by the c-MCTS algorithm. Initially these playouts are contained within the Node as additional information, but it has also been set up that the c-MCTS is able to pull playout trials from the node and turn them into child nodes.

With regards to generating the random vectors for the playouts, more care needs to be taken when performing stochastic sampling as the number of variable dimensions increase. In order to allow the reinforcement learning to properly determine what path to take next, it is important to ensure that the generated sample points is high in quality. However, there are a great many stochastical traps that one can fall into if new sample points are generated in a naive way. This is due to what is commonly referred to as “the curse of dimensionality" where intuitions that are completely valid in three dimensions or fewer fall apart as the dimensionality of the system increases. In this case, randomly generating a displacement vector from the node's starting parameter set such that each dimension of the vector is generated independently of the others will almost certainly result in a bad displacement vector. For example, if each vector component is generated uniformly within an the interval $(-dx, dx)$, the probability that a single component will fall between $(-0.1 * dx, 0.1 * dx)$ is 10%. The probability that all the components of the factor will fall within $(-0.1 * dx, 0.1 * dx)$ on their respective distributions is 0.1^N. For a 3 component vector i.e. $N = 3$, this probability is 10^{-3} which while rare is still a high enough frequency that it can be observed. However, for a 18 component vector, the frequency becomes 10^{-18} which all but guarantees it will never be generated except through extraordinary luck. In fact, for 18 dimensions there is a 18% chance that at least one of the components will be approximately dx in magnitude. As such, the vector will almost always amount to a large displacement from the initial position. Even a distribution such as the N-Gaussian falls victim to this. If there's a 66% chance that a single variable will be generated within one standard deviation from the starting point, then for a 18 dimensional Gaussian the probability is 0.66^N which is 5.6×10^{-4}. While better than uniform, this is still an unreasonably low probability since it will take roughly 1000 trials to finally generate a vector that is not a large displacement.

In order to overcome this challenge especially for materials applications dimensionality can be much larger than 18, the choice of distribution must scale with dimensionality. However, a simple choice that is surprisingly effective is to generate a vector uniformly on the surface of an N-Sphere of radius 1 and then uniformly pick the vector length. On the surface, this may not appear to be any different than picking each component. But because we are picking the distance, R, which is a collective variable, we can show that it is actually a biased distribution.

$$\int_0^{r_{max}} dr = \int_0^{r_{max}} J(r) f(r) dr \tag{4}$$

Where $J(r)$ is the radial component of the Jacobian for the polar coordinates and $f(r)$ is the probability density function. For visual simplicity the normalization constant is neglected from this equation. This of course assumes that the angular components have already been fixed and thus integrated out. In order to have a distribution that is uniform on r, the product of the probability density function and the Jacobian must equal a constant. This implies

$$f(r) = \frac{1}{J(r)} \tag{5}$$

If we examine the radial component of the Jacobian for a N-Sphere, we find it is simply given by

$$J(r) = r^{N-1} \tag{6}$$

As such the probability density function regardless of the number of dimensions must equal

$$f(r) = \frac{1}{J(r)} = \frac{1}{r^{N-1}} \tag{7}$$

This implies the probability distribution in Cartesian space is given by

$$\int_0^{r=r_{max}} = \frac{1}{(\sum_{i=1}^{N} x_i^2)^{\frac{(N-1)}{2}}} dx_1 dx_2 ... dx_N \tag{8}$$

Or that the distribution is biased toward the origin. Unlike picking each component individually, regardless of the number of dimensions, there will always be a reasonable chance of picking both large and small displacement vectors. In a continuous action space c-MCTS algorithm, this allows the reinforcement learning to determine what size of vector is needed in order to find a better reward function. If short vectors are never generated, it is possible for the algorithm to be close to a good solution, but be unable to find it since it will always overshoot it. The above modifications address this challenge in high-dimensional search space.

3.2.2 Exploitation in Continuous Action Space:

A last consideration when modifying MCTS for a continuous space is that there must be some way for the algorithm to narrow in on a solution and eventually converge. We find that using a constant maximum vector length, one would find a decent solution but the search is highly inefficient. If the step size is too large, the algorithm does no better than a random search. If the step size is too small, it would take many node expansions to finally find a good solution.

In addition, we note that a fundamental part of a continuous action c-MCTS tree is missing in that there exists little correlation between the information stored in a node and the information stored inside of its parent node.

In a board game c-MCTS algorithm, each node contains a "game state" which essentially captures the various game piece's positions on the game board. When you make a child node, this corresponds to making a legal move within the game. As such, the child node is related to its parent by the fact that you can obtain the child's position by moving a single piece from the parent's position. Restoring this correlation is paramount to instill logical sense into the c-MCTS algorithm formalism, in addition to ensuring that its results are consistent. To do this, we introduce a window scaling scheme. In this scheme, the r_{max}, which represents the largest vector distance that can be generated, is assigned to smaller and smaller values depending on the depth of the corresponding node in the c-MCTS tree. For example, the head node is effectively "unscaled" or that it is allowed to generate a point anywhere within the global parameter search boundary. Its children nodes, depth of 2 in the c-MCTS tree, in contrast are only allowed to pick r_{max} values such that no single parameter can change more than 25% of its total search boundary. This again is scaled further and further as tree branches are expanded to deeper and deeper values. This telescoping window scaling approach ensures the algorithm is incrementally refining the phase space. This allows the algorithm to initially make larger scans of the phase space as it finds interesting regions where it is allowed to zoom in and begin exploring in more detail. This restores the correlation between the parent and the child node – the child node is a zoomed in region around the parent node which gives the algorithm some direction to ensure that one is not simply performing a purely random walk. It also allows it to converge sufficiently close to an optimal solution since it is making smaller and smaller adjustments as it expands the tree depth. The modified exploitation allows for faster convergence even while allowing exploration over a much larger continuous action search space.

3.2.3 Initial Design

In practice during initial start up of the c-MCTS tree, a collection of approximately 20-50 child nodes with 2-3 playouts each are added to the head node before the formal selection process begins. This effectively forces the head node expansion. This is done to seed the algorithm with the information that it can then use to decide how it wishes to start exploration. It was found this improves the quality of the MC tree, as simply starting from the head node can result in some inconsistent expansions. This seed data can be generated either through purely random sampling, latin hyper cube sampling [6], or any other suitable method. In addition, one node is always set as the center point of the hyper cube. This is done because, as mentioned previously, it is far more likely to randomly generate a point on the edge of the hyper cube than at the center for high dimensional problems. Although for the trial test function described in the next section this was not done because the center point is often the analytical solution of the trial function and thus would be cheating to start from there and instead all points were generated uniformly. For the force field fitting however the center point is always included in the initial design.

3.3 State-of-the art c-MCTS approaches versus the c-MCTS developed in this work

In this section we briefly the differences between some of the popular c-MCTS approaches and our method. The continuous action space MCTS developed by Couetoux et al [7]), which is also known as progressive unpruning (Chaslot et al. 2008 [8]), gradually increases the number of available actions at each node based on the visitation count. The other MCTS algorithm developed by Lee et al [9] is equipped with the gradient-based fine-tuning of the finite set of action samples which performs MCTS for searching globally and search locally via action-value gradient ascent. Both of these algorithms as well as several other gradient free optimization schemes are based on sequentially constructing partitions of the search space where, at each iteration, a finer resolution partition is created inside the most promising cell of the current partition. A challenge with existing c-MCTS approaches lies in their poor scalability – most of them cannot scale to dimensionality > 20.

The approach by Kim et al.[9] mentioned by the reviewer introduces Value-Gradient UCT (VG-UCT), which combines a traditional MCTS with gradient-based optimization of action particles. Note that this methodology

requires gradient information and works well when well-defined gradients exist in the search landscape. For searches will ill-defined gradients which is typically the case with most materials optimization problems, this approach does not lead to performance improvement. On the other hand, the approach by Couetoux et al. maintains a finite list of child nodes and a new child node is added depending on the number of visitations to the parent node. Here, the main concern is when to sample a new action as against which action to sample. They assume that the probability of sampling a near optimal action is the same in every visit to the node. This can often lead to a sluggish performance. This approach suffers from coarse-grained discretization and requires a vast number of child nodes to be sampled to precisely select optimal actions.

In our scheme, we utilize a "window scaling scheme" wherein the child node searches within a narrower region of the parent node – this ensures a better correlation between the child and parent node and also accelerates the convergence within any given branch of the tree. Moreover, we also introduce adaptive playouts where the random simulations were biased to sample those regions that were closer to the parent node tends to significantly improve the performance. The improved quality of the playouts leads to faster convergence especially in the case of high dimensional search space. Additionally, we note that a major problem with materials optimization in continuous action space is the high level of degenerate solution which we address by defining a uniqueness criteria – this is different from any of the prior c-MCTS approaches. This avoids the common issue of convergence of two initially separate MCTS branches to the same region of the continuous search space. More importantly, this resolves the problem of multiple representations of the same (degenerate) solution as often encountered in several physical problems.

Thus, our scheme addresses some of the major limitations of existing c-MCTS approaches i.e. the issue of scalability to extremely high dimensions, the issue of degenerate solutions and the issue of finding an optimal solution when the search landscape has ill defined gradients.

4 Supplementary Discussion

4.1 Performance of c-MCTS on Trial Functions

To demonstrate the performance of the continuous action c-MCTS compared to other optimization schemes, we performed comparative tests using nearly 25 trails functions [10, 11]. The performance of each algorithm was evaluated in terms of the solution quality—if it reached the target solution—and efficiency—number of iterations to reach the target solution. The performance of c-MCTS along with other three optimizer are summarised in Supplementary Table 1. Each method was ran 30 times and averaged for each function. Here, in this section we have provided the progress of the optimizer to reach the target solutions in terms of number of iterations.

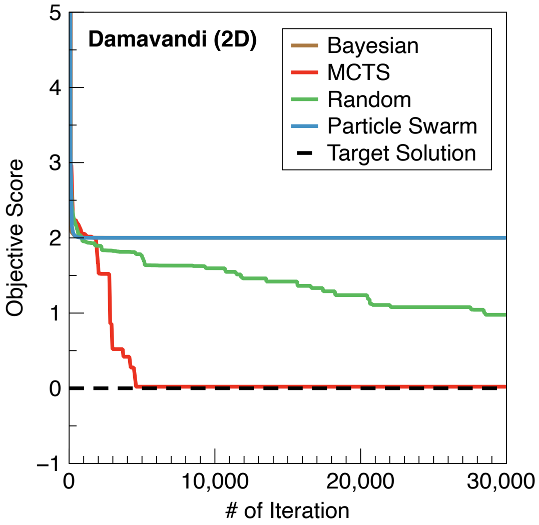

Supplementary Figure 10: Performance of c-MCTS in comparison to other optimizer on Damavandi's function. We observe that Bayesian and particle swarm optimizer trapped in local minima whereas c-MCTS is able reach the global minima in < 3000 iterations.

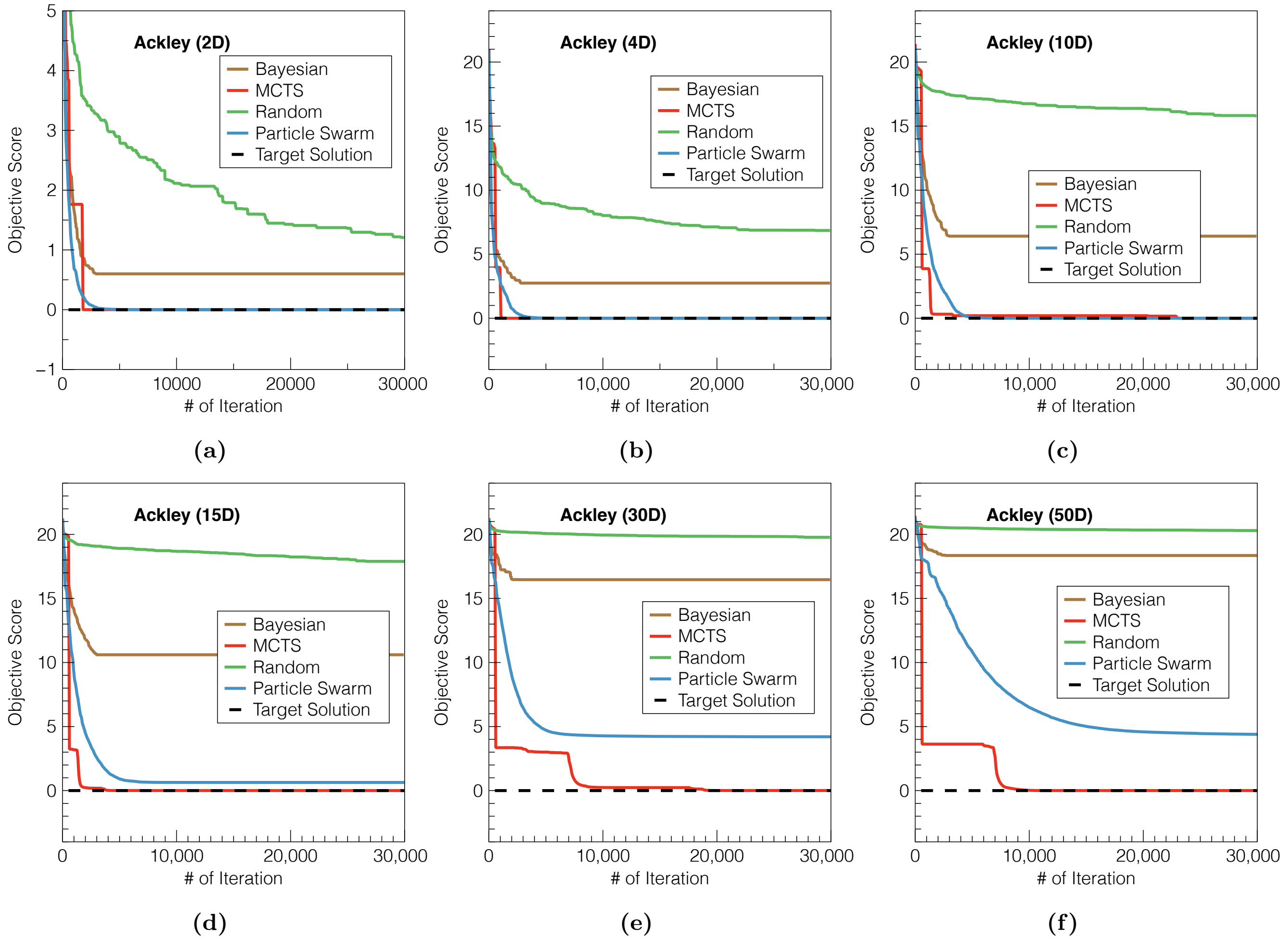

Supplementary Figure 11: Performance of c-MCTS in comparison to Bayesian, particle swarm, and random sampling techniques to reach the target solution of different dimensions of Ackley's function.

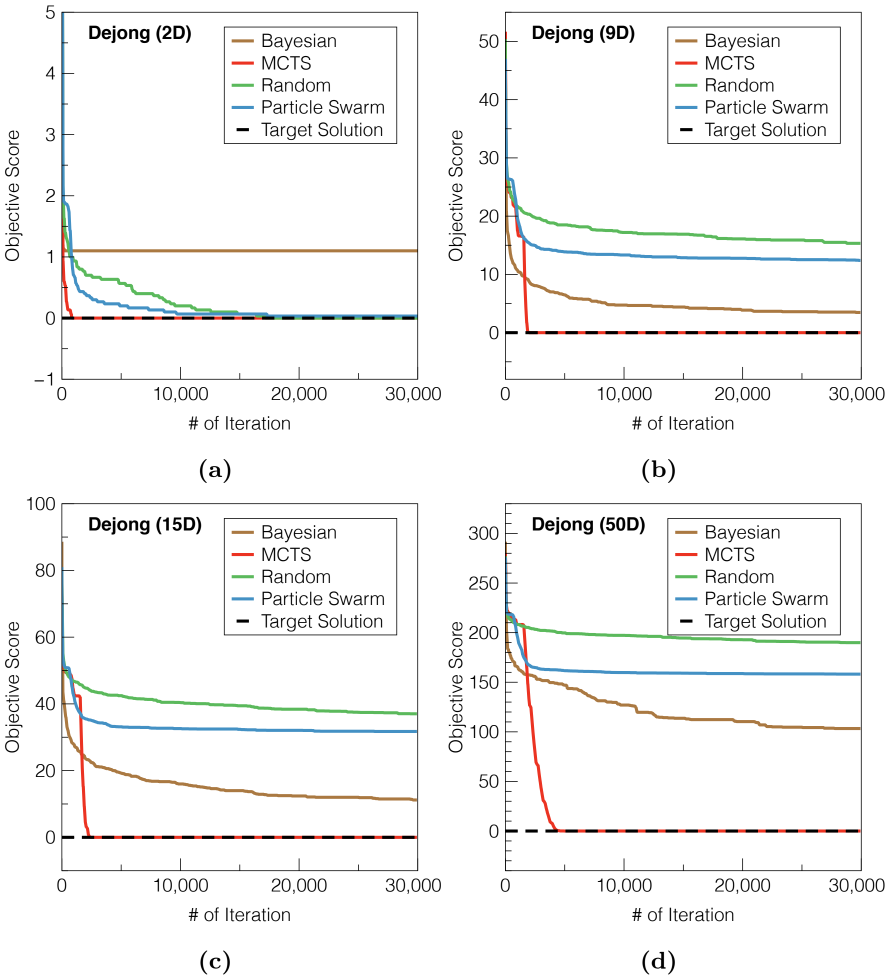

Supplementary Figure 12: Performance of c-MCTS in comparison to Bayesian, particle swarm, and random sampling techniques to reach the target solution of different dimensions of Dejong's step function.

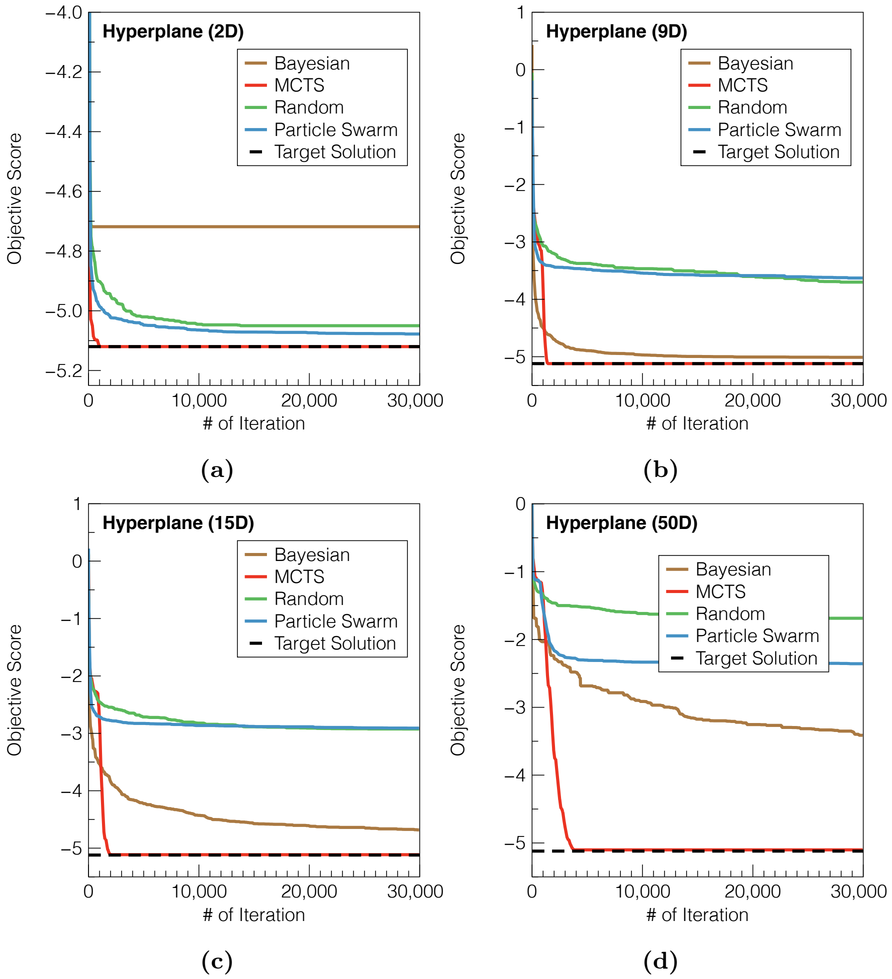

Supplementary Figure 13: Performance of c-MCTS in comparison to Bayesian, particle swarm, and random sampling techniques to reach the target solution of different dimensions of Hyperplane function.

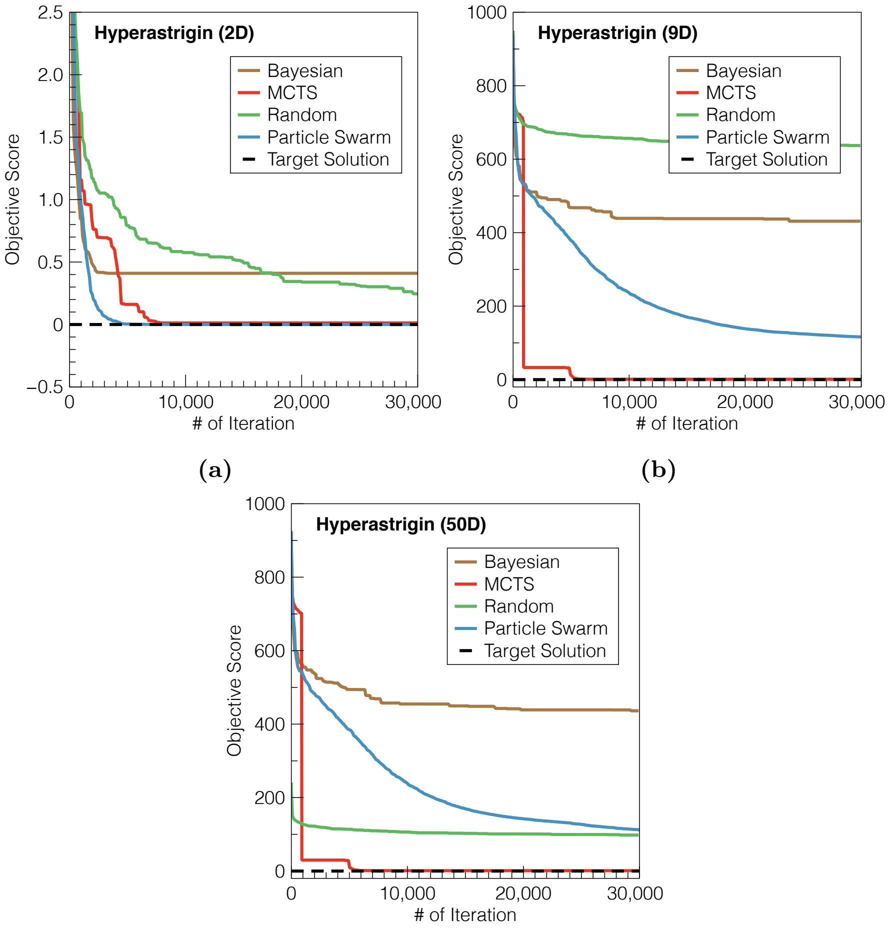

(c)

Supplementary Figure 14: Performance of c-MCTS in comparison to Bayesian, particle swarm, and random sampling techniques to reach the target solution of different dimensions of Hyperastrigin function.

4.2 Scalability in Particle Swarm Optimization (PSO) and Bayesian optimization (BO)

Particle Swarm Optimization (PSO) is a global optimization scheme wherein the search optimization is dependent on the swarm size [12, 13]. If the swarm is less than the problem dimensionality, then it is not possible for the swarm to sample adequately the various regions in the search space. Typical swarm sizes are $\sim$ 50-100 meaning that scaling to high dimensional surfaces » 100 are often problematic and prone to convergence issues arising from inadequate sampling. Another challenge often encountered in Particle Swarm optimization is the possibility of velocity explosion [12] in the initial few iterations of PSO. In such cases, the particles leave the search space (often away from the likely solution) and never return to the region for the duration of the search. PSO is largely dependent on initialization strategies [12], as problem dimensionality increases, initialization strategies that only generate positions within a small region of the search space perform better than strategies that attempt to maximize coverage of the search space. The issues with scalability and sampling are evident as we increase the trial function dimensionality to 50D and higher.

Bayesian optimization is also a global optimizer that combines the predicted value and its uncertainty in a single acquisition function to select the next location for measurement, thus, balancing exploration and exploitation [14]. The acquisition function can be designed in many different ways, including maximum probability of improvement, maximum expected improvement and Thompson sampling [15]. These methods are collectively classified as Bayesian optimization or active learning. An important caveat to bear in mind is that the benefits of Bayesian optimization

are strongly influenced by the model parameters and the definition of the acquisition function, as underscored in Ref. [16] Bayesian optimization can tend to be inefficient or expensive when the parameter search space is enormous (for example, in the game of Go or in the search for the lowest-energy configuration such as in a high-entropy alloy compositional search). This is due to the computational complexity of optimizing in a high-dimensional space where the optimization objective is non-convex (such as the case with some of the trial functions deployed for comparison). Nonetheless, Bayesian optimization is one of the most successful techniques in the materials discovery and design, especially for problems with dimensionality < 100. A more detailed explanation of various machine learning algorithms can be found in this review article [17].

4.3 c-MCTS for Reinforcement Learning in Non Materials Science Problems

We had tested this algorithm on a wide class of reinforcement learning problems. Here are few examples.
Example 1: The first of which is a PyGame based Flappy Bird clone.

https://github.com/SeppeDeWinter/NEAT_flappy_bird

In this a 10x10x10x1 network with 7 input nodes takes various information about the Bird's velocity, position, etc. were used to determine if the bird should take no action or to jump.

Example 2: We trained a convolution neural network that is capable of playing the first level of Sonic the Hedgehog for the Sega Genesis.

github.com/mrnucleation/SonicBot

In this a 2D Convolutional Network was set up to read the game's video output and convert it down into an action space which corresponds to the buttons being pressed on a virtual controller. The network structure was a 102x50 Conv2D input layer that was flattened into a Dense 5 node output layer for a total of 240,707 parameters.

Example 3: We had also trained a large network capable of playing Super Mario Brothers 1 for the NES. We were able to train networks to play the first level of the game. The first being a 64x64x64 2D-Convolutional input that is then flattened into 512x64x6 Dense layers. The final network was capable of beating the first level. We primarily did this as a stress test to see if it was capable of being extended into higher dimensions.

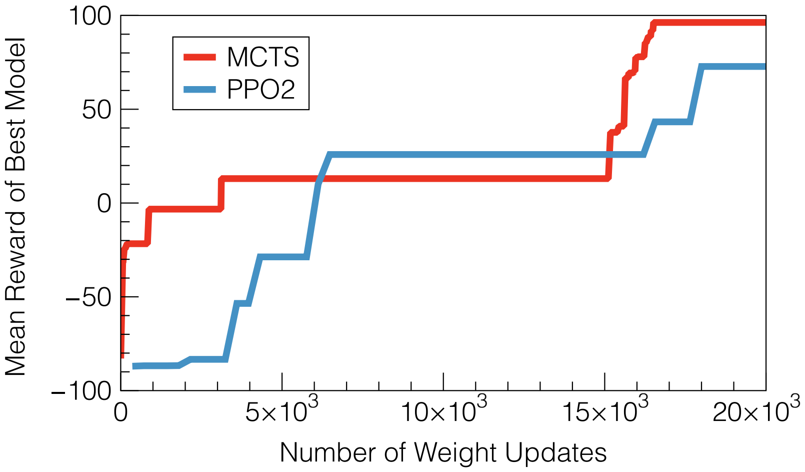

Supplementary Figure 15: A plot showing the average reward of the best performing weight set as a function of the number of total weight sets sampled. 100 is a perfect score for each trial run.

Example 4: In addition to objective optimization, it was found that the c-MCTS performed well for classic Reinforcement learning problems. The c-MCTS was applied to the Continuous Mountain Car (MountainCarContinuous-v0) and Continuous Lunar Lander (LunarLanderContinuous-v2) physics environments that are part of Open AI's Gym package. In both simulation environments a neural network with hyperbolic tangent activation functions was trained. A neural network with a node structure of 10:10:5 for a total of three hidden layers was used for both environments. The inputs and outputs of the network were sized to match the number of observable and number of controls in both cases. The reward was given by the OpenAI environment. A single set of network parameters was tested on 5 random start positions and ran till the finished condition was returned by the OpenAI interface. No other information about the system apart from what was directly supplied by the default environment binding was given

to the network. The mean reward of the 5 tests is then returned as the objective to the c-MCTS which is then asked to maximize this value. For final evaluation the best network was taken and ran on 500 randoms start configurations to test for consistency. It was found that in 537 total weight sets a solution that was capable of clearing all 500 trials for the mountain car was found while for the lunar lander a solution that was able to clear 321 of the 500 trials was found in 3119 weight sets while one that was able to clear all 500 was found in 16254 weight sets. The results for the Lunar Lander are showing in Supplementary Figure 15. Videos for both runs are included in the supporting files.

4.4 Methods to Generate Cluster configurations

4.4.1 Nested Ensemble Based Search for Entropically Favored Clusters

A Boltzmann based Metropolis sampling and a nested ensemble-based approach [18, 19, 20] were used to generate the structures. This was done to gather information on both thermally relevant structures predicted by DFT as well as higher energy structures which may still be important for creating an accurate model. The Metropolis simulation was run for 5,000 Monte-Carlo (MC) cycles at 300K with the initial structure being randomly picked from dimer. The Nested Ensemble simulations were run for another 500,000 cycles with configurations saved after each 1,000 cycles. For each simulation method, a cycle consists of N moves where N is set to the number of atoms in a given simulation. This procedure allow us to generate cluster configurations with a wide range of energy window. For each element, number of atoms varied from 5 to 30. In order to ensure proper clustering during the MC simulations, a Stillinger Cluster Criteria [21] was imposed to ensure connectivity. Any configuration that would result in the destruction of a cluster is rejected in favor of the previous position. For each system the cluster criteria distance was set to the first neighbor distance between minimized atoms with an additional tolerance of 0.5 Å for some structural flexibility.

4.4.2 Structure Mining for Low Energy Cluster Configurations

While the nested ensemble based structures may not include the structures that have low energy configurations. To include the low energy cluster configuration we mined the structure from Quantum cluster Database [22]. The Quantum Cluster Database contains the low energy cluster energy configurations across the Periodic table including the clusters from Cambridge cluster database [23] as well as nanoclusters mined from the supplemental information from the published literature. This database contains the atomic structures of 54 different elements with size range from 2-50 atoms.

4.4.3 Chemical Replacements in Structure Prototype (CRISP)

For elements which do not have readily available low energy clusters for a given size, we replace the element type in known configurations with the bond distances approximated using the hard sphere radii [24, 25].

4.4.4 Genetic Algorithm

We also use genetic algorithm based on GASP [26] to find out the low energy configurations of nanoclusters of the required size. This method has been used rigorously to predict the atomic structures of nanoclusters (of different sizes) and bulk systems in the past [27, 28].

4.5 Details of the DFT calculations

The Vienna Ab-initio Software Package (VASP) [29] with the Perdew-Burke-Eznerhof (PBE) [30] exchange-correlation functional was used to perform all density functional theory (DFT) calculations for evaluating energy and forces of clusters in the training and test datasets. The spin polarization was included in this DFT calculations. The projector-augmented wave (PAW) potentials used in these calculations are summarised in Supplementary Table 2. A single k-point at the center of the Brillouin zone was used for each calculation. Gaussian smearing with a width of 0.001 eV was used to set partial occupancies. The convergence criteria for the electronic self-consistent iteration and the ionic relaxation loop were set to be 0.1 meV and 1 meV per cluster, respectively.

The elemental bulk structures were collected from the Materials Project database [31]. A dense k-point grid, defined by $n_{\text{atoms}} \times n_{\text{kpoints}} \approx 1000$, where n_{atoms} is the number of atoms in the primitive cell and n_{kpoints} is the number of k-points. The DFT calculations were used to evaluate cohesive energies and lattice parameters. A relatively high tolerance of 10^{-6} eV for energy convergence was employed. The elastic constants were determined by employing suitable lattice distortions [32, 33, 34, 35, 36, 37, 38] as implemented in the VASP finite difference methods.

PBE DFT was used employed to study elemental system. One could include spin orbit coupling for clusters with heavy metals to further improve the quality of the training dataset. For example, we performed PBE + SOC [39] on 9 different heavy elements as described in Ref. [40, 41] from the periodic table. The correlation plot is shown in Supplementary Figure 16. As seen from the correlation plot, including SOC is not expected to change the quality of the results obtained. In principle, one can use beyond DFT and higher fidelity quantum calculations to reduce the inherent error in the training dataset. With PBE DFT, the typical errors are $\sim$ 20 meV/atom. With CCSD [42], one can expect these errors to go down to less than 5 meV/atom depending on the system [43]. The accuracy of the fit is ultimately limited by the accuracy of the training data.

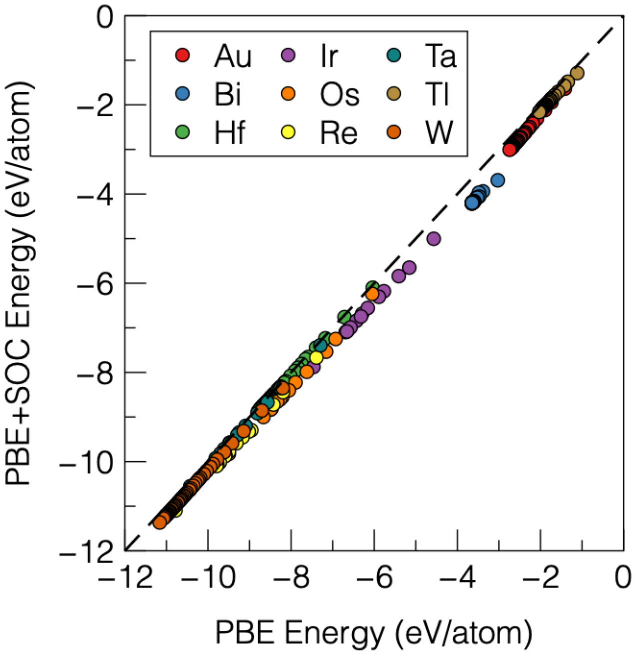

Supplementary Figure 16: Energy computed using standard PBE and PBE + SOC for the clusters of 9 different heavy elements.

Supplementary Table 2: PAW pseudopotentials used in VASP software to calculate cluster energies and forces.

Element	POTCAR	Element	POTCAR
Ag	Ag 02Apr2005	Nb	Nb_pv 08Apr2002
Al	Al 04Jan2001	Ni	Ni 02Aug2007
As	As 22Sep2009	O	O 08Apr2002
Au	Au 04Oct2007	Os	Os 17Jan2003
B	B 06Sep2000	P	P 06Sep2000
Ba	Ba_sv 06Sep2000	Pb	Pb 08Apr2002
Be	Be 06Sep2000	Pd	Pd 04Jan2005
Bi	Bi 08Apr2002	Pt	Pt 04Feb2005
Br	Br 06Sep2000	Rb	Rb_pv 06Sep2000
C	C 08Apr2002	Re	Re 17Jan2003
Ca	Ca_pv 06Sep2000	Rh	Rh 04Feb2005
Cd	Cd 06Sep2000	Ru	Ru 04Feb2005
Cl	Cl 06Sep2000	S	S 06Sep2000
Co	Co 02Aug2007	Sb	Sb 06Sep2000
Cr	Cr 06Sep2000	Sc	Sc 04Feb2005
Cs	Cs_sv_GW 23Mar2010	Se	Se 06Sep2000
Cu	Cu 22Jun2005	Si	Si 05Jan2001
F	F 08Apr2002	Sn	Sn 08Apr2002
Fe	Fe 06Sep2000	Sr	Sr_sv 07Sep2000
Ga	Ga 08Apr2002	Ta	Ta 17Jan2003
Ge	Ge 05Jan2001	Te	Te 08Apr2002
H	H 15Jun2001	Ti	Ti 08Apr2002
Hf	Hf 20Jan2003	Tl	Tl 08Apr2002
Hg	Hg 06Sep2000	V	V 08Apr2002
I	I 08Apr2002	W	W 08Apr2002
In	In 08Apr2002	Y	Y_sv 25May2007
Ir	Ir 06Sep2000	Zn	Zn 06Sep2000
K	K_pv 17Jan2003	Zr	Zr_sv 04Jan2005
Li	Li 17Jan2003		
Mg	Mg 13Apr2007		
Mn	Mn 06Sep2000		
Mo	Mo 08Apr2002		
N	N 08Apr2002		
Na	Na 08Apr2002		

5 Supplementary Note 1

5.1 Performance Comparison with Existing ML based Potentials

Supplementary Table 3: Summary of ML trained FF available in literature. Note that these models often show MAE of 1-5 meV/atom on bulk data but errors on cluster datasets are much higher as shown below.

ML trained Force Fields	Al	C	Cu	Ge	Li	Mo	Ni	P	Si
SNAP (meV/atom)	-	-	1709.5	2798	698.8	3091.0	2710.9	-	2109.2
qSNAP (meV/atom)	-	-	1839.0	3362.9	706.9	2780.7	2672.4	-	2262.0
GAP (meV/atom)	-	947.7	-	-	-	-	-	608.1	1898.0
MEGNet (meV/atom)	722.6	996.7	1205.6	438.0	856.1	2492.5	754.2	1980.9	1253.6
c-MCTS trained ML-HyBOP (meV/atom)	65.5	143.5	56.3	71.7	89.5	200.1	61.1	145.7	77.7

Supplementary Table 4: Computational cost for different potential model for Si_{55} cluster at 300 K.

Force Field	Computational Cost in core sec for 10 ps
ML-HyBOP	6.47
GAP	8117.39
SNAP	1008.36
qSNAP	1471.22

6 Supplementary Note 2

6.1 Performance Comparison with Existing Potentials

Using the developed nanocluster dataset as described in section 4.4), we evaluated the performance of several state-of-the-art empirical potential models [44, 45] that are regularly adopted for materials simulations. Specifically, we compared the energy and force predictions of well-known and a wide variety of potentials (including EAM [46, 47], MEAM [48, 49, 50], ADP [51, 52], SW [53], BOP [54, 55, 56, 57], Tersoff [58, 59] and other formalisms) against that of the DFT values. Comparison results across 38 elements, covering over 120 different potentials are described in section 6.2 - 6.38. These results serve as a benchmark to the HyBOP potentials developed in this work, and also underscores the difficulty of training an accurate potential for nanocluster systems. Importantly, we note that our HyBOP models accurately predict energies and forces across a wide range of cluster sizes and bulk, and capture their dynamic stability. Most of the force fields collected from literature are trained on bulk properties, rather than nanocluster systems. This explains their poor performance in predicting properties of nanoclusters.

Supplementary Table 5: Summary of the force fields collected from literature to predict nanocluster energies and forces. The references for each force field is mentioned in the square brackets.

Element	Functional Form	Element	Functional Form	Element	Functional Form
Ag	eam/fs [60], eam/alloy [61], sutton chen [62]	Ir	sutton Chen [62]	Ru	eam/fs [63]
Al	eam/fs [64], meam [65], sutton chen [62], adp [66], meam, agni [67]	K	eam/fs [68]	S	sw [69]
As	tersoff [70], bop [56]	Li	eam/fs [68]	Se	sw [69], bop [71]
Au	eam/fs [60], eam/alloy [44], adp [72], sutton chen [62]	Mg	eam/fs [73], eam/alloy [74], adp [75]	Si	sw [53], tersoff [76], edip [77], vashista [78], meam/spline [79], tersoff/mod/c [80]
B	tersoff [81], extep [82]	Mn	.	Sn	.
Be	eam/alloy [83], tersoff [84]	Mo	eam/fs [85], eam/alloy [74], meam/spline [86]	Ta	eam/fs [87], eam/alloy [74], adp [88]
C	airebo [89], lcbop [90], bop [91], airrebo/morse [92], tersoff [81], vashistha [78],	Na	eam/fs [68]	Te	sw [69], bop [71]
Cd	sw [69], bop [71]	Nb	eam/fs [93], eam/alloy [94], adp [95]	Ti	eam/alloy [74], meam/spline [96], eam/fs [97]
Co	eam/alloy [98]	Ni	eam/fs [93]	V	eam/fs [99], eam/alloy [100]
Cr	adp [101], eam/alloy [102]	P	eam/fs [103]	W	eam/fs [104], eam/alloy [100],
Cs	.	Pb	eam/alloy [105], eam/fs [74]	Zn	sw [69]
Cu	eam/fs [106], eam/alloy [74]	Pd	eam/alloy [74]	Zr	eam/fs [107], eam/alloy [74], adp [95]
Fe	eam/fs [100], eam/alloy [108], meam [109], tersoff [110]	Pt	eam/alloy[74]		
Ga	tersoff [70], sw [111]	Rb	eam/fs [68]		
Ge	tersoff [112], tersoff [113]	Ru	eam/fs [63]		
		Rh	Sutton Chen [62]		

6.2 Ag

6.2.1 Energy Correlation Plots

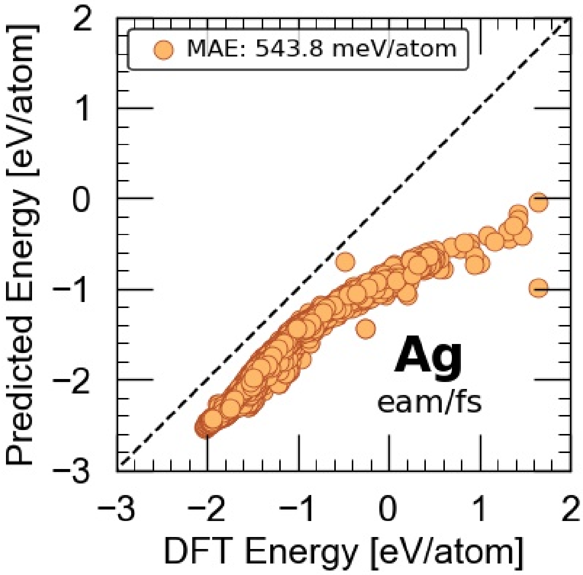

(a)

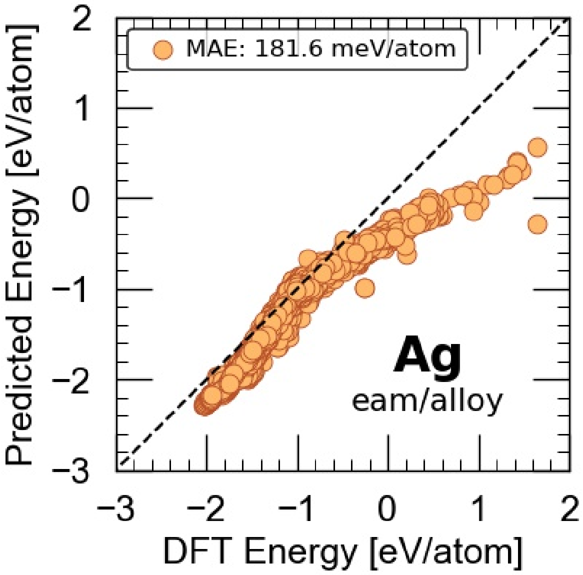

(b)

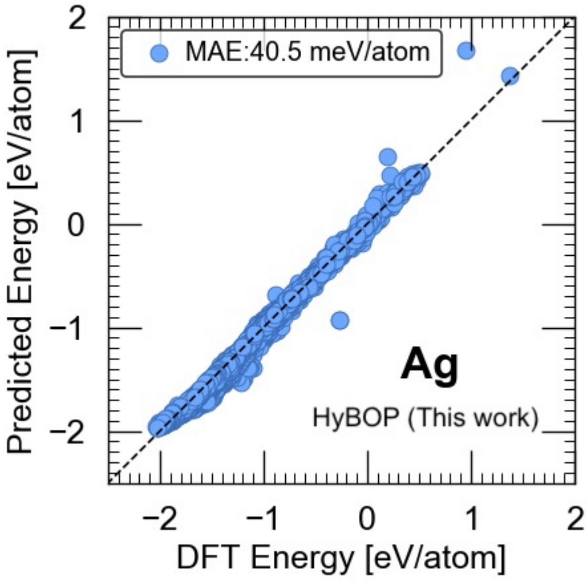

(c)

6.2.2 Force Correlation Plots

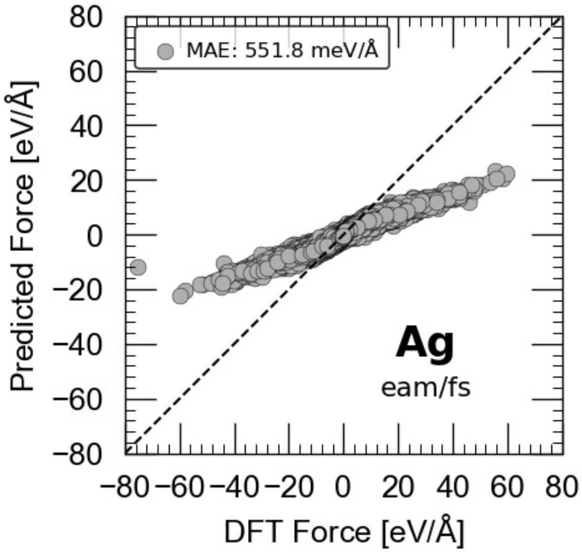

(a)

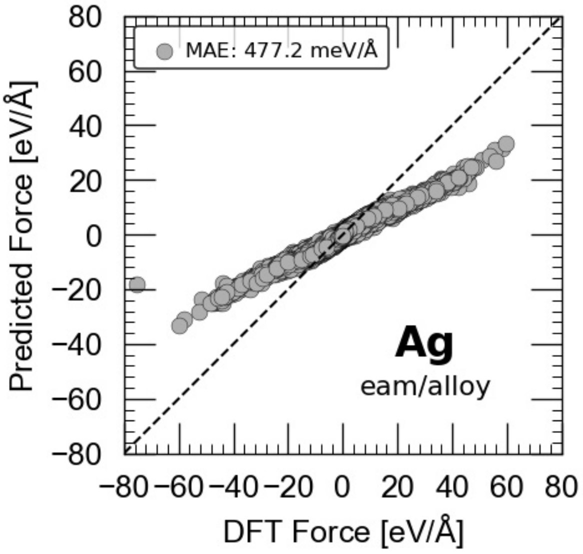

(b)

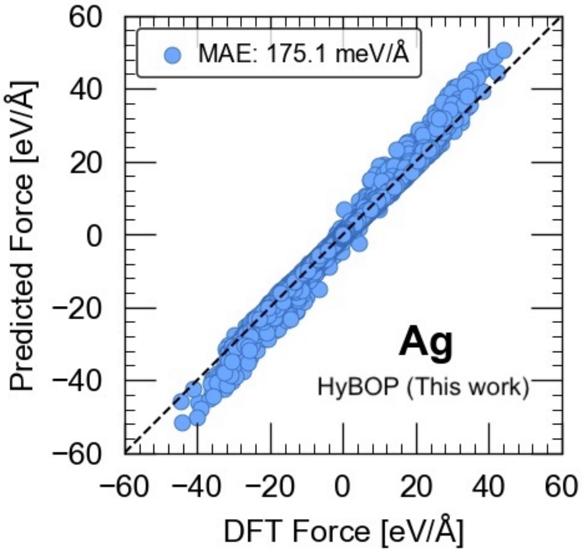

(c)

6.3 Al

6.3.1 Energy Correlation Plots

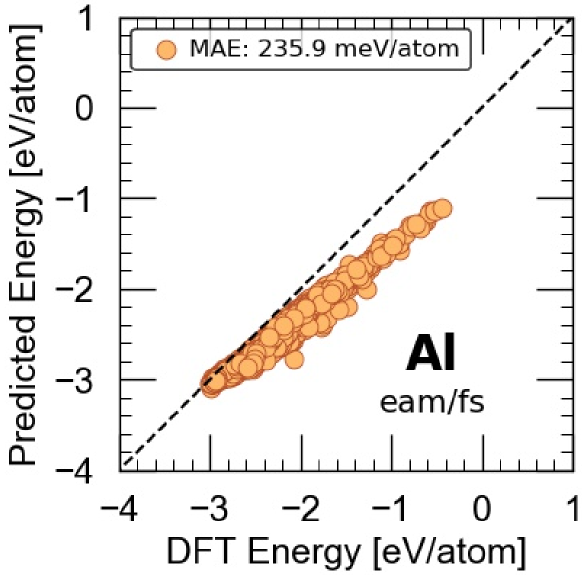

(a)

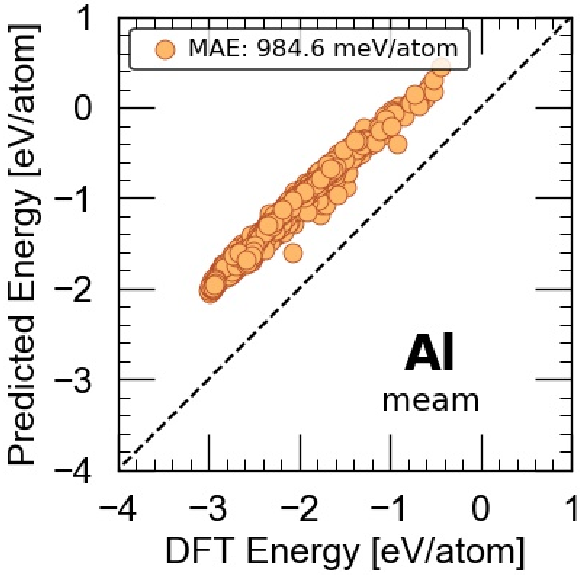

(b)

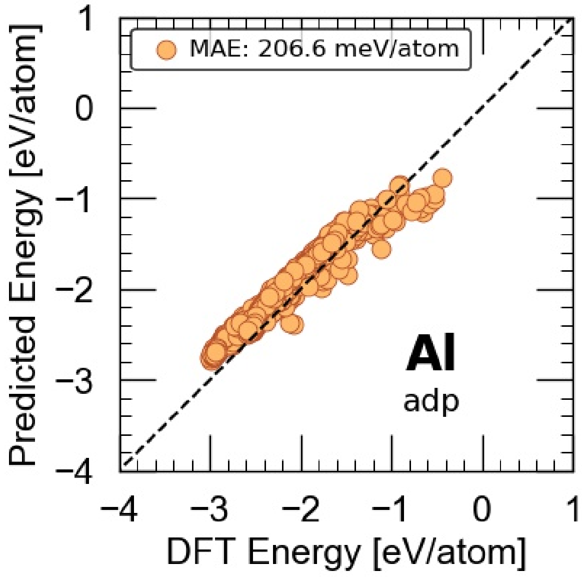

(c)

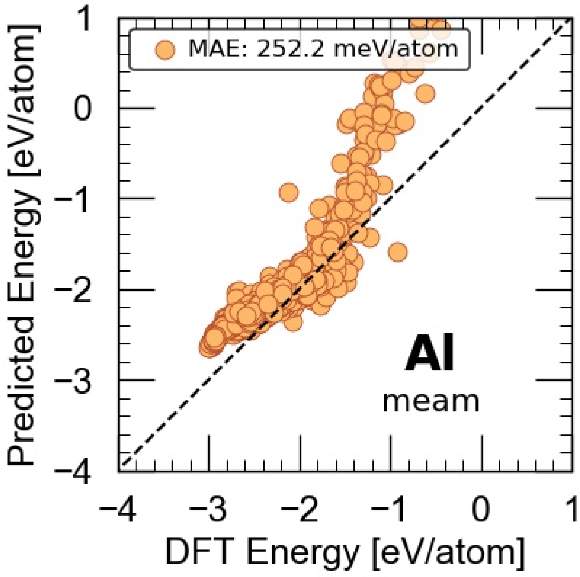

(d)

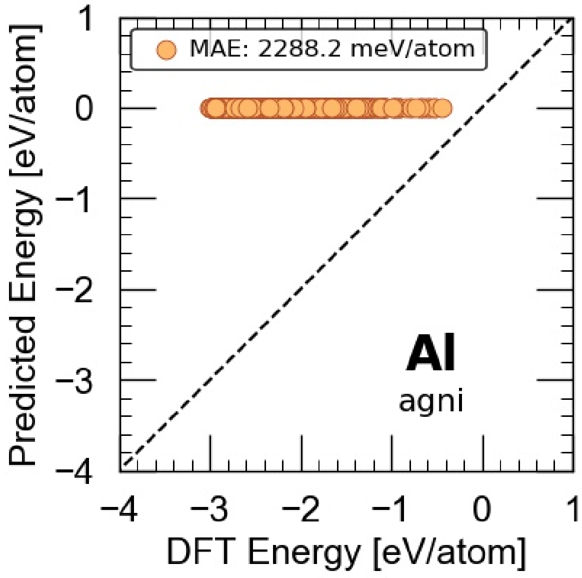
MAE: 2288.2 meV/atom
Al
agni
Predicted Energy [eV/atom]
DFT Energy [eV/atom]
1
0
−1
−2
−3
−4
−4
−3
−2
−1
0
1

(a)

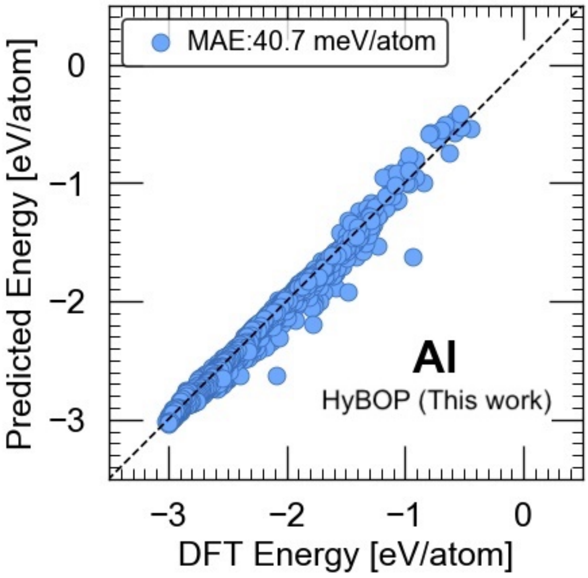
MAE:40.7 meV/atom
Al
HyBOP (This work)
Predicted Energy [eV/atom]
DFT Energy [eV/atom]
0
−1
−2
−3
−3
−2
−1
0

(b)

6.3.2 Force Correlation Plots

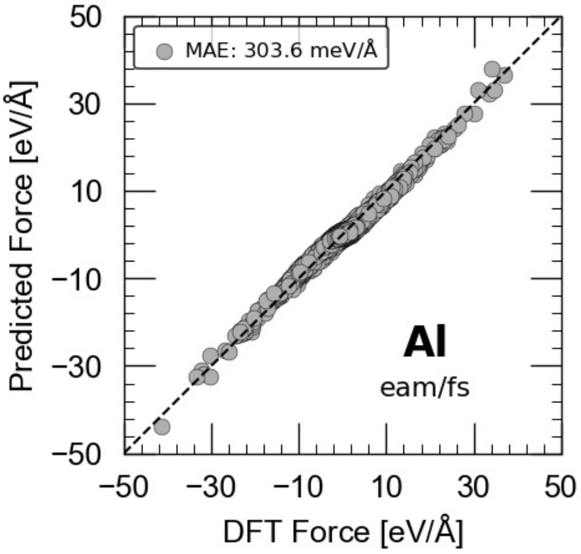

(a)

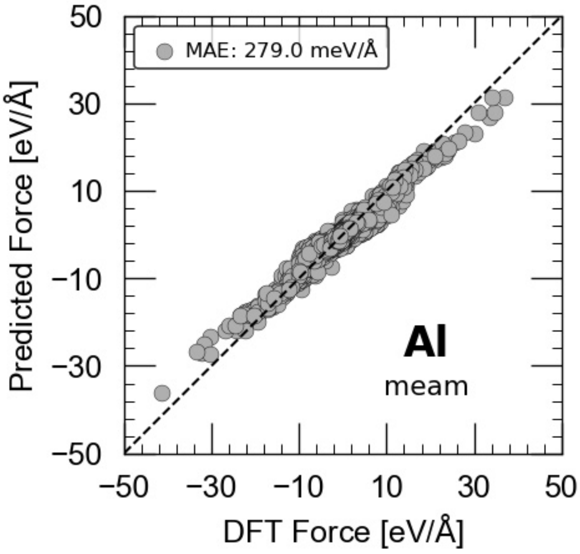

(b)

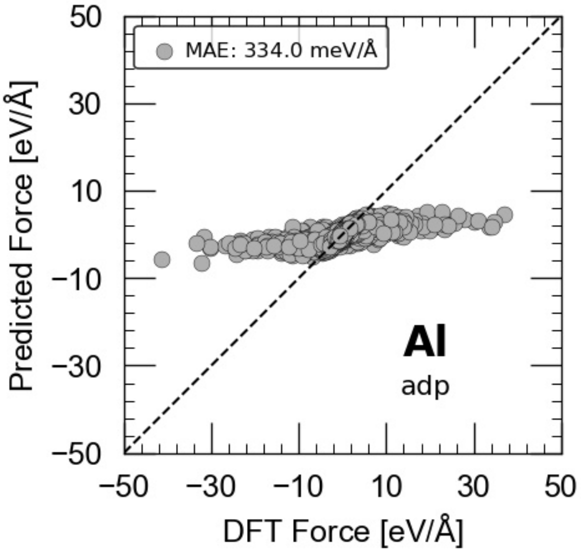

(c)

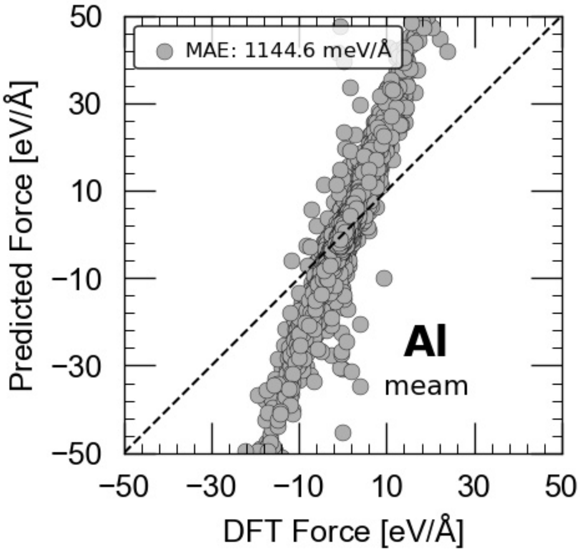

(d)

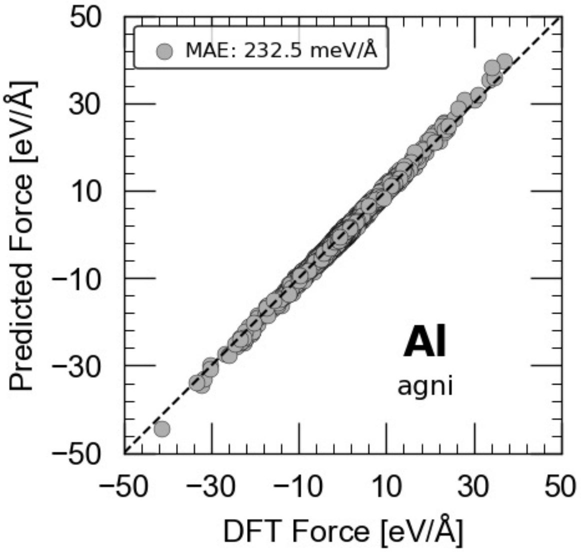
MAE: 232.5 meV/Å
Al
agni
Predicted Force [eV/Å]
DFT Force [eV/Å]
50
30
10
−10
−30
−50
−50
−30
−10
10
30
50

(a)

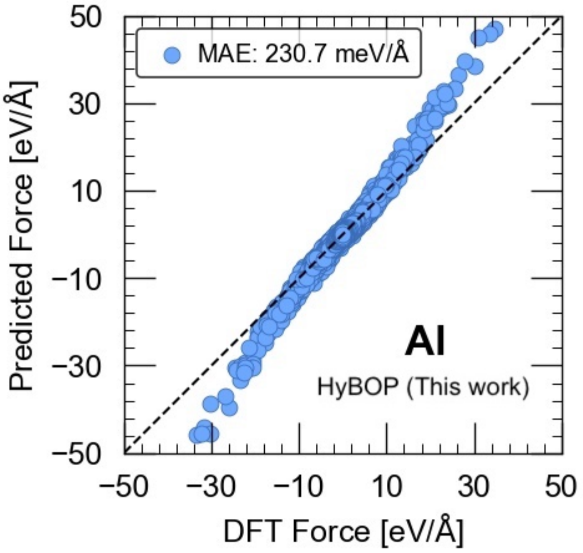
MAE: 230.7 meV/Å
Al
HyBOP (This work)
Predicted Force [eV/Å]
DFT Force [eV/Å]
50
30
10
−10
−30
−50
−50
−30
−10
10
30
50

(b)

6.4 As

6.4.1 Energy Correlation Plots

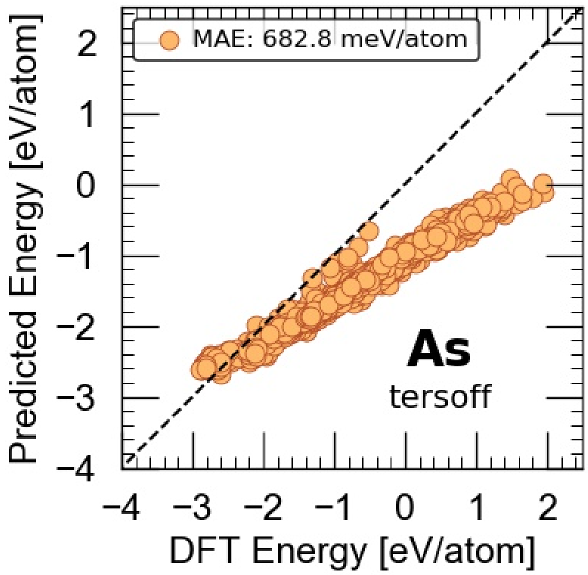

(a)

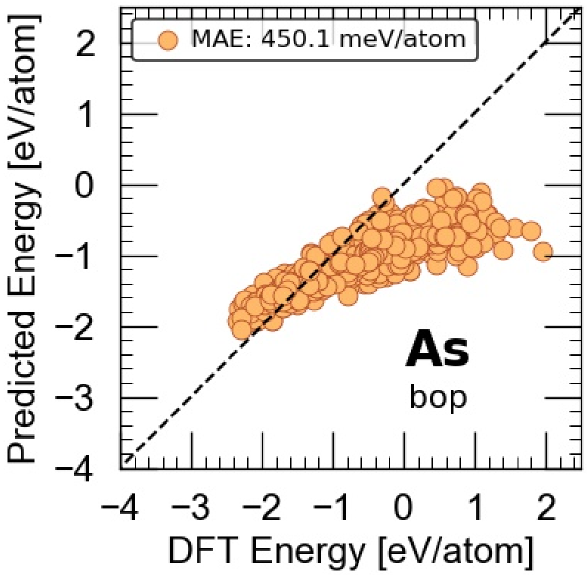

(b)

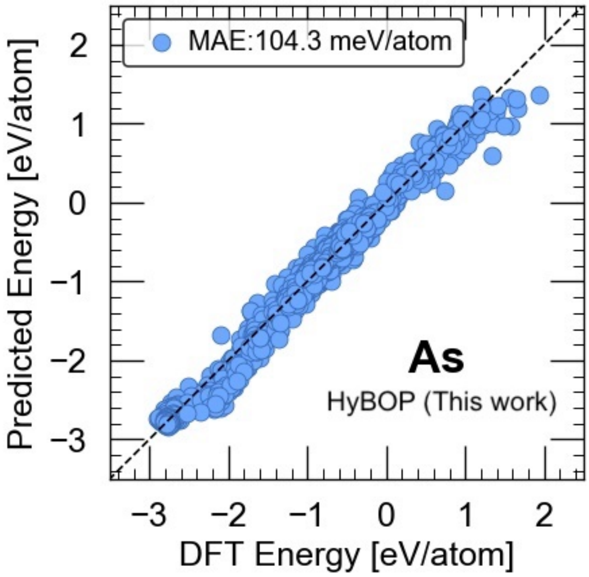

(c)

6.4.2 Force Correlation Plots

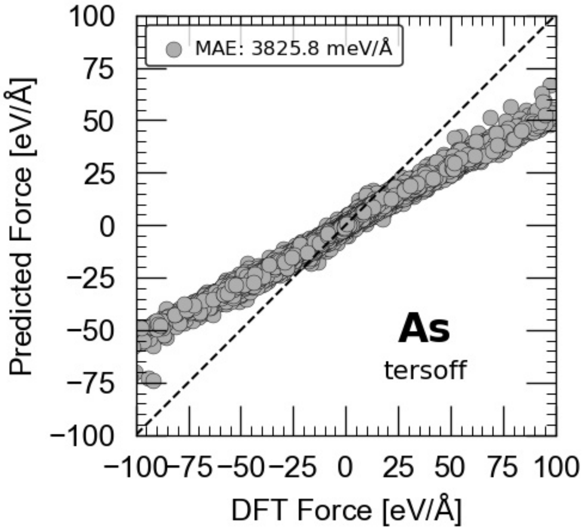

(a)

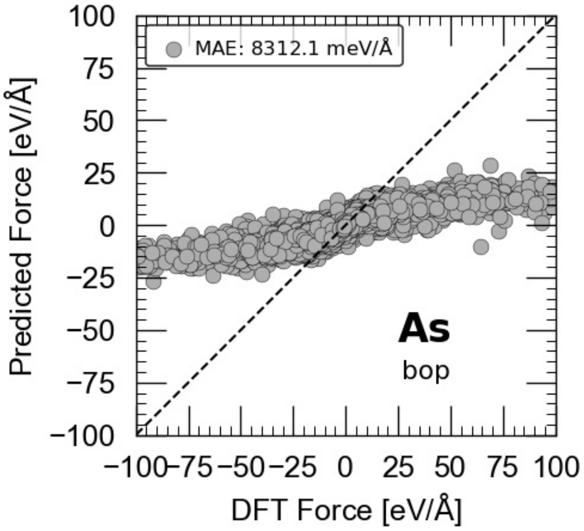

(b)

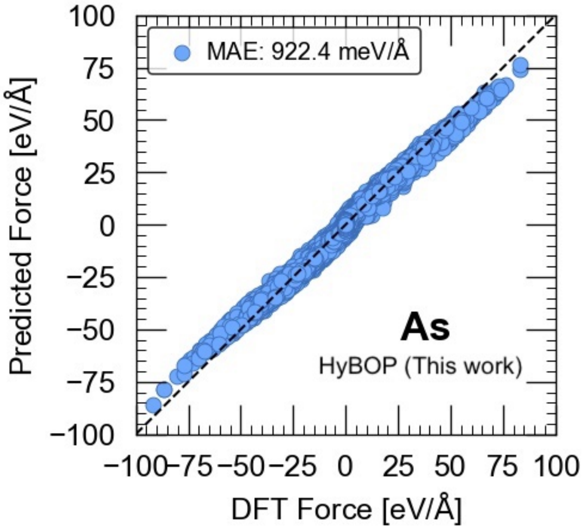

(c)

6.5 Au

6.5.1 Energy Correlation Plots

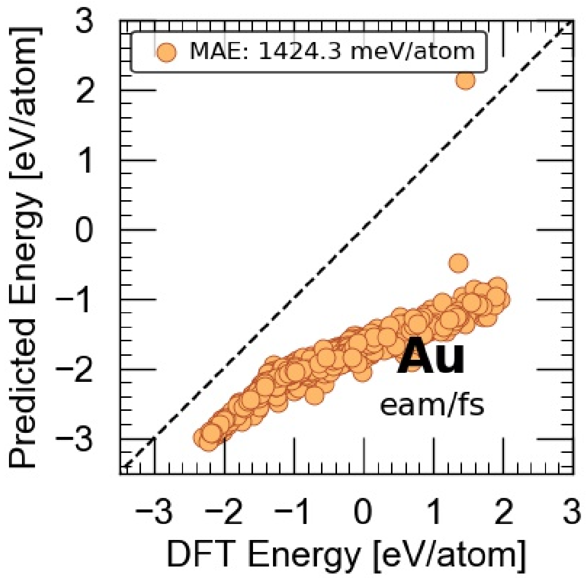

(a)

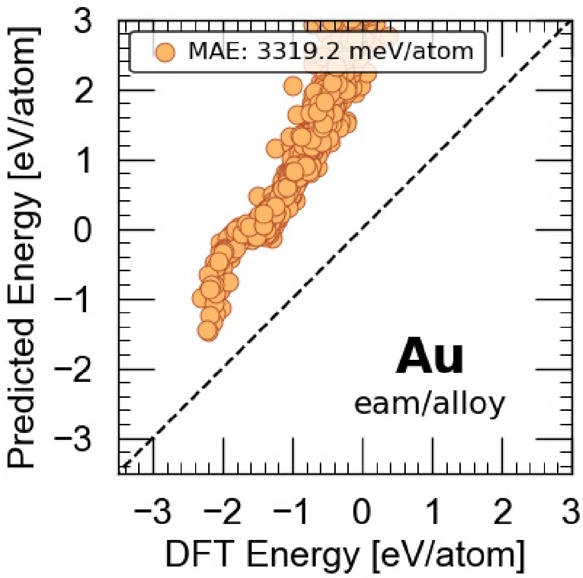

(b)

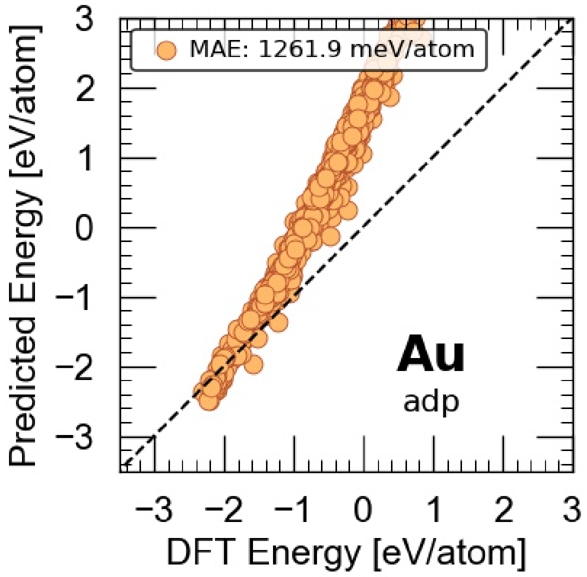

(c)

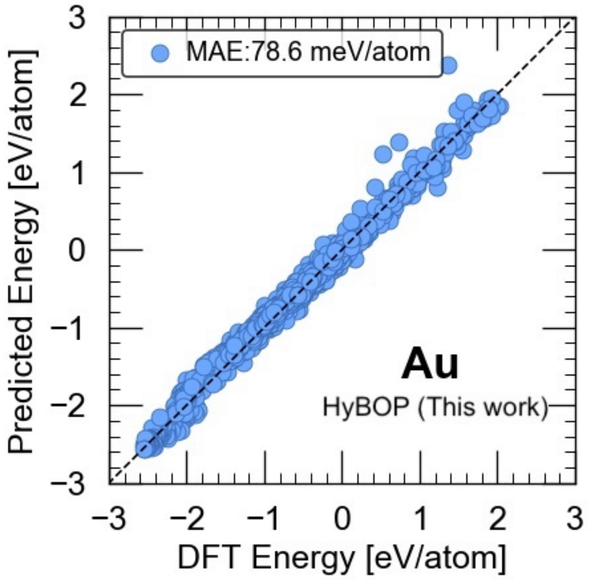

(d)

6.5.2 Force Correlation Plots

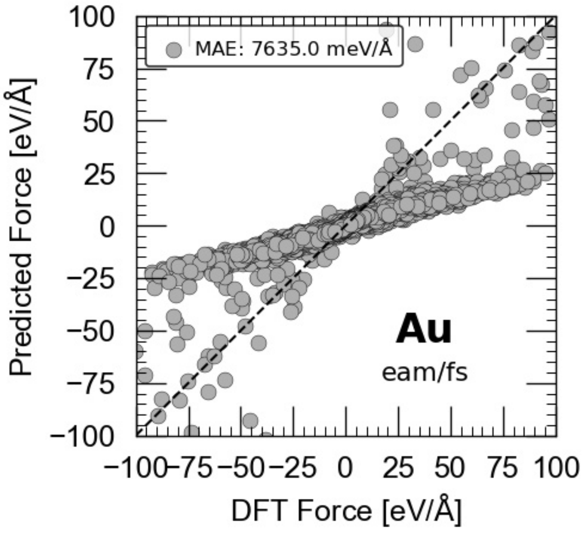

(a)

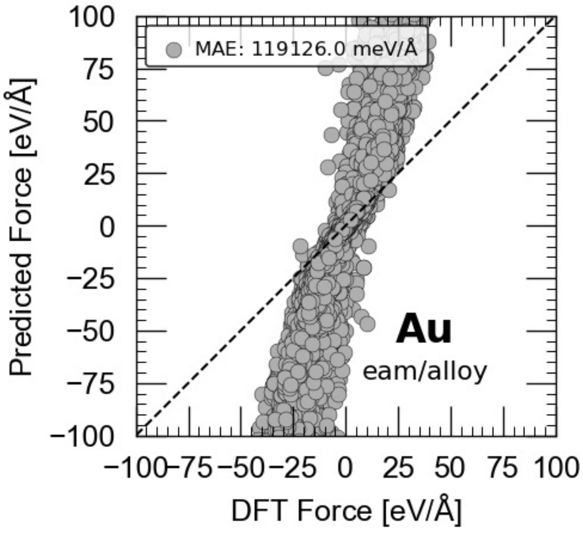

(b)

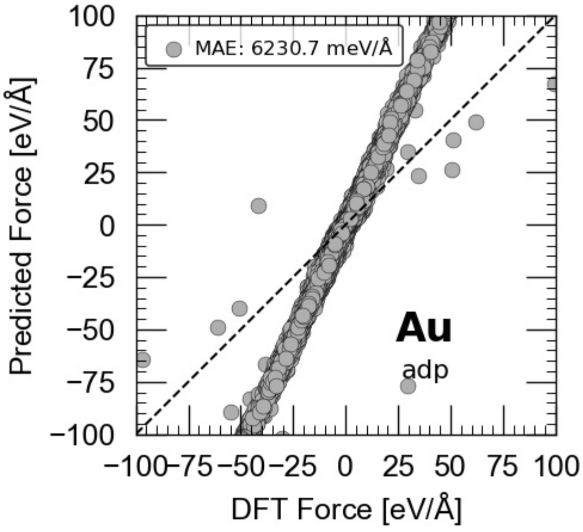

(c)

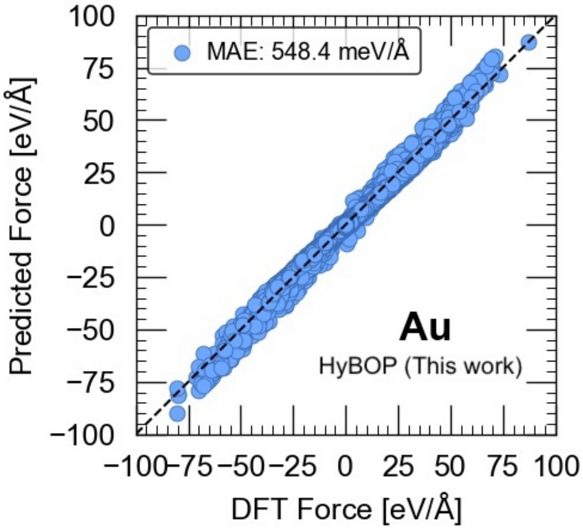

(d)

6.6 B

6.6.1 Energy Correlation Plots

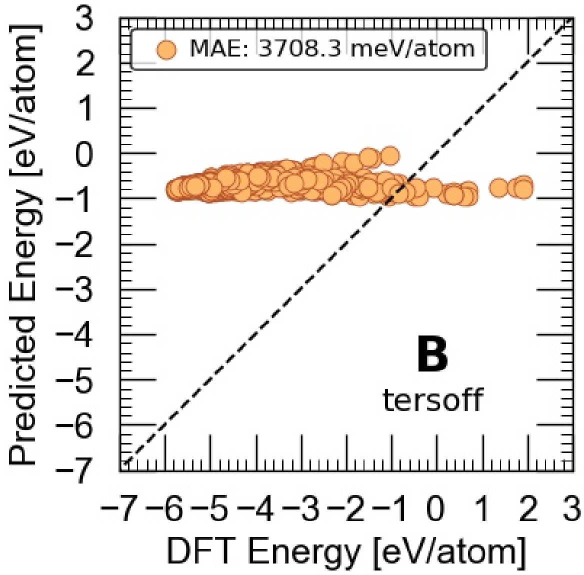

(a)

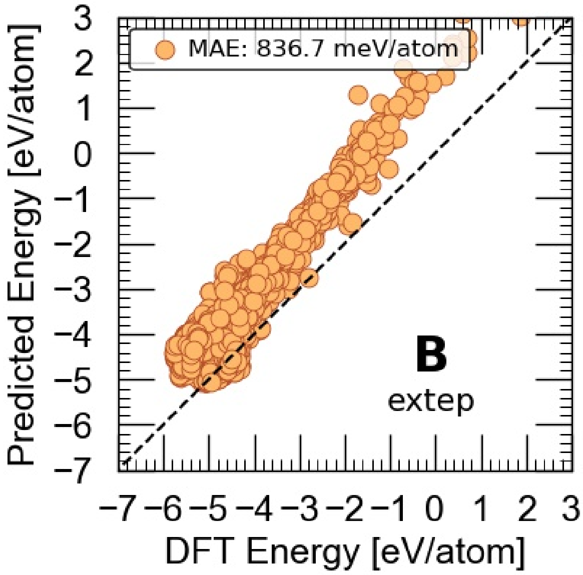

(b)

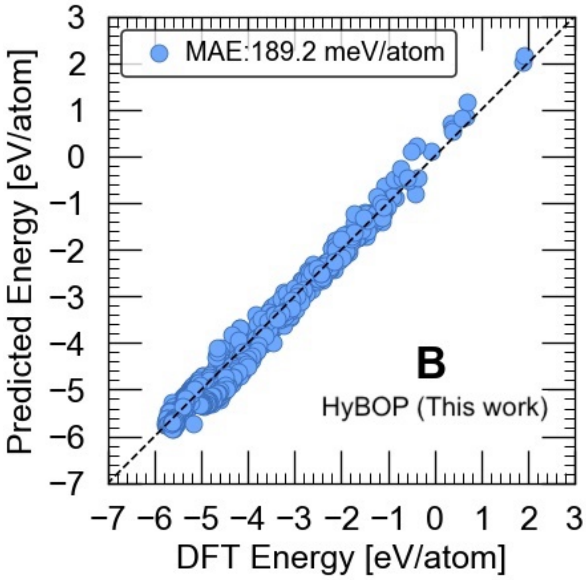

(c)

6.6.2 Force Correlation Plots

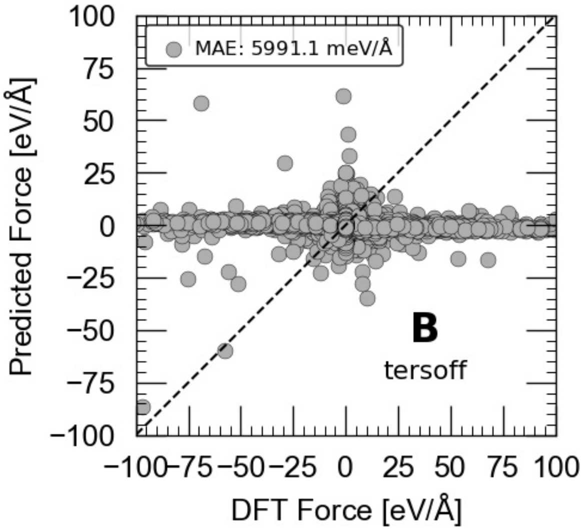

(a)

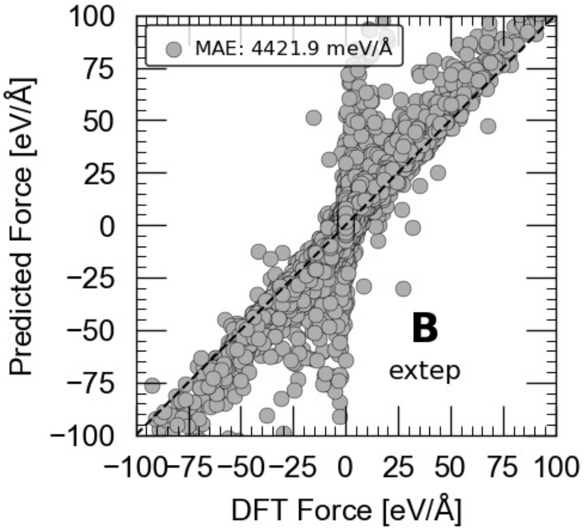

(b)

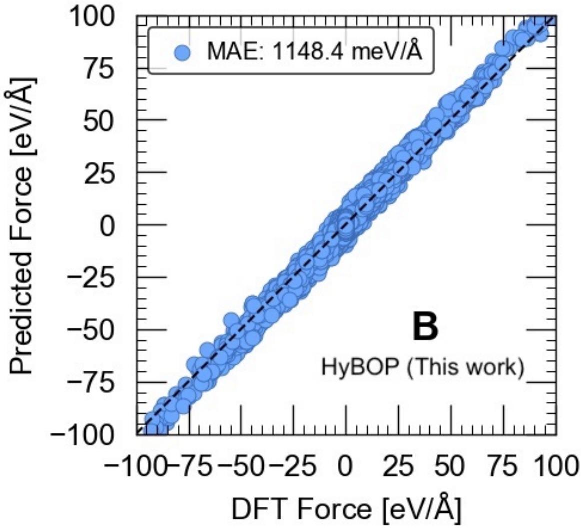

(c)

6.7 Be

6.7.1 Energy Correlation Plots

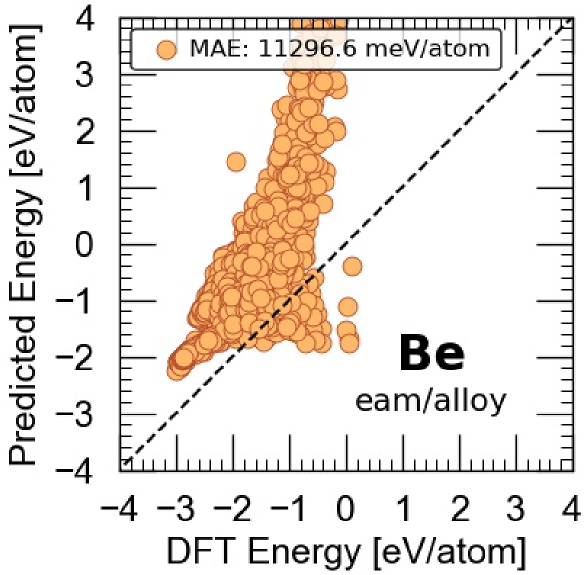

(a)

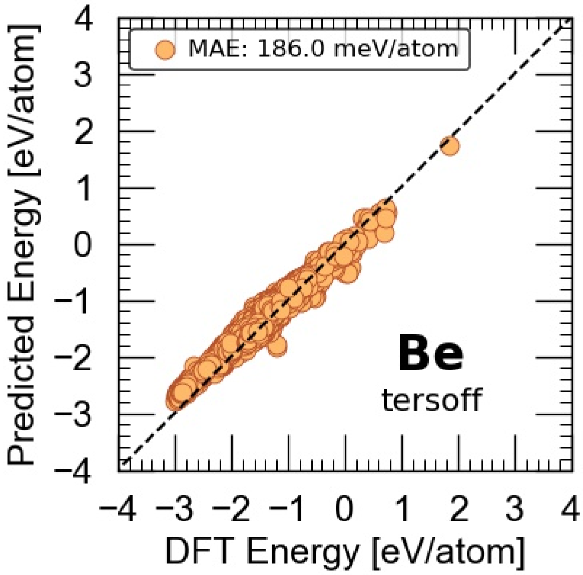

(b)

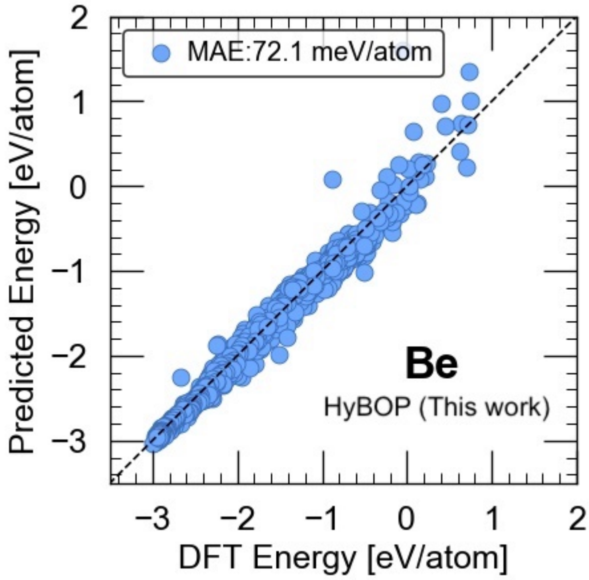

(c)

6.7.2 Force Correlation Plots

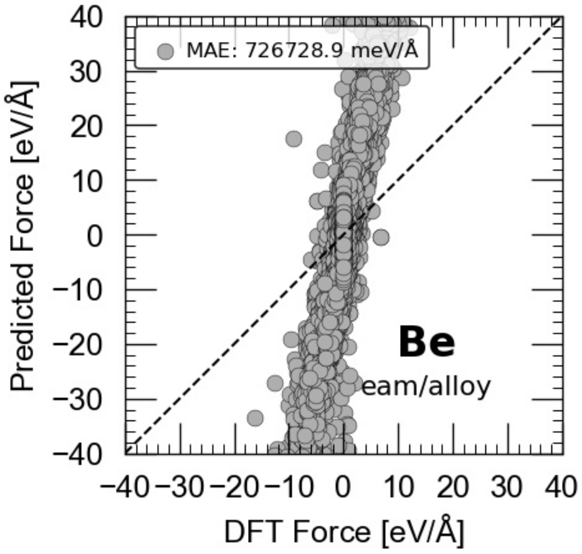

(a)

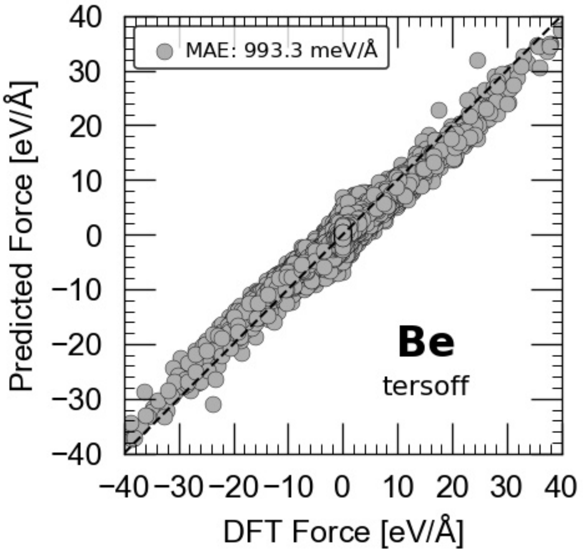

(b)

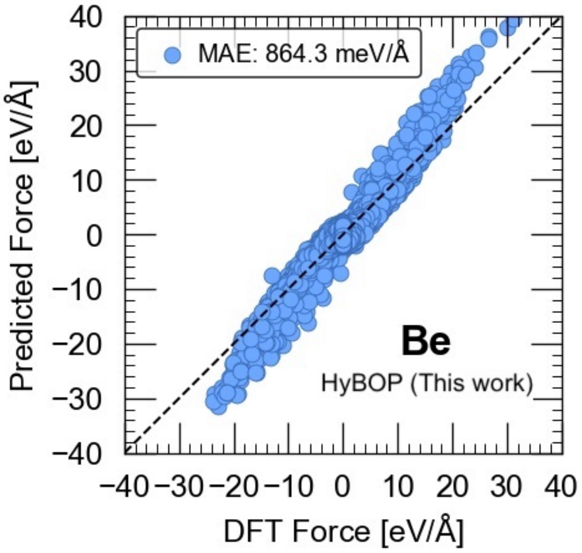

(c)

6.8 C

6.8.1 Energy Correlation Plots

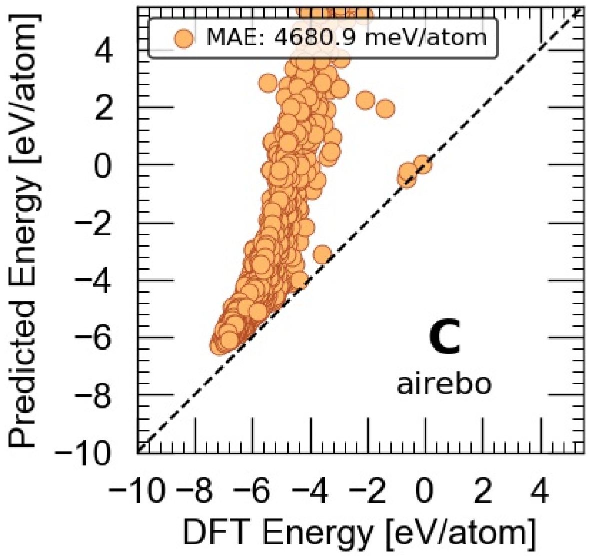

(a)

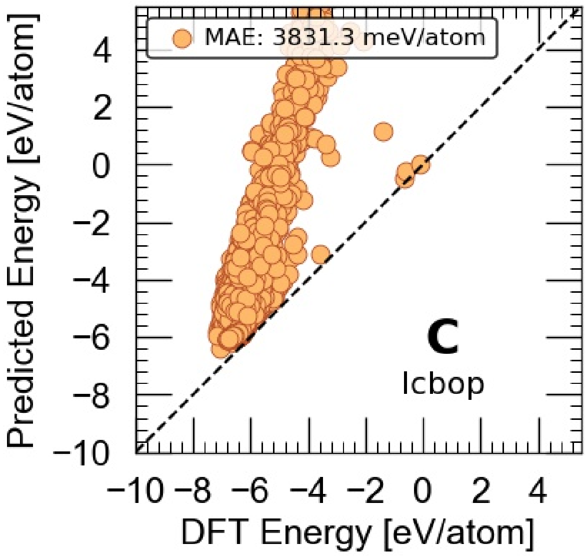

(b)

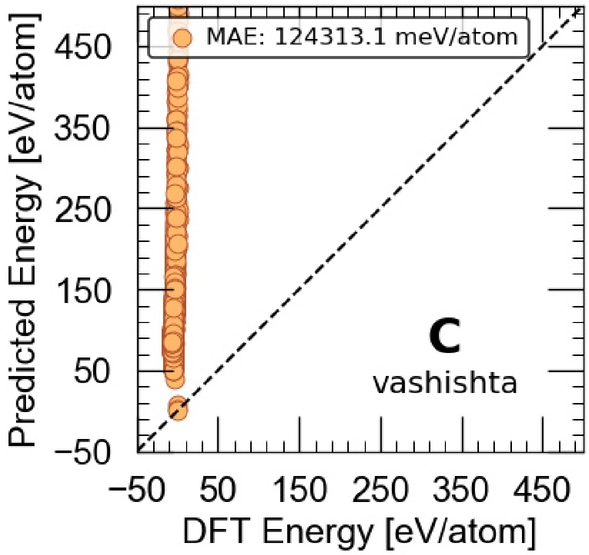

(c)

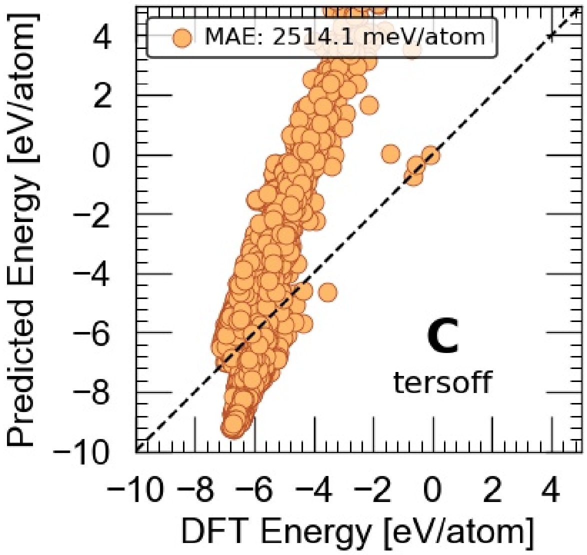

(d)

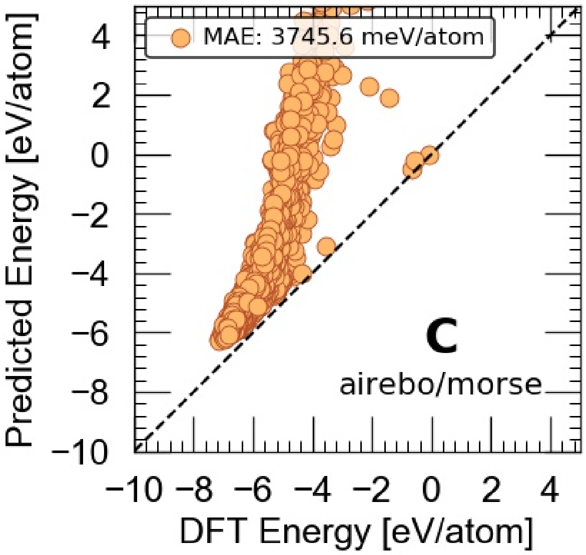
MAE: 3745.6 meV/atom
C
airebo/morse
Predicted Energy [eV/atom]
DFT Energy [eV/atom]

(a)

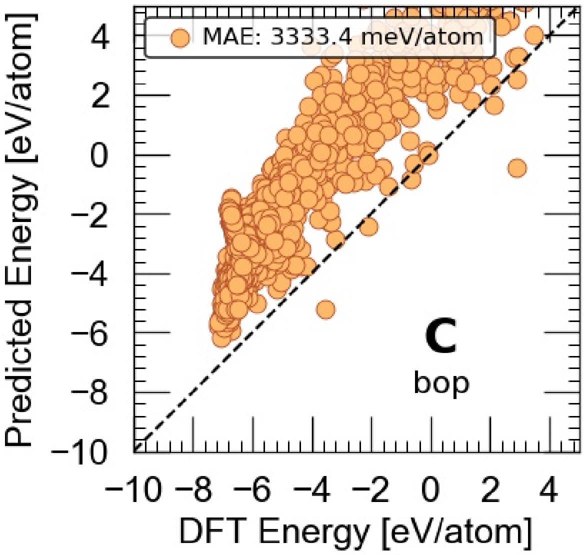
MAE: 3333.4 meV/atom
C
bop
Predicted Energy [eV/atom]
DFT Energy [eV/atom]

(b)

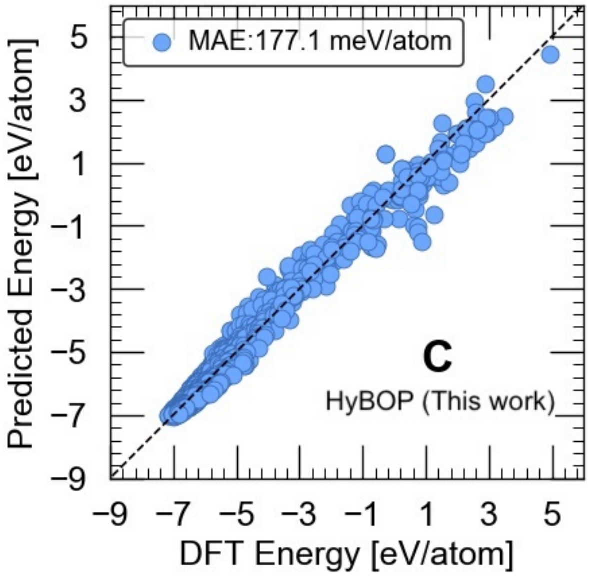
MAE:177.1 meV/atom
C
HyBOP (This work)
Predicted Energy [eV/atom]
DFT Energy [eV/atom]

(c)

6.8.2 Force Correlation Plots

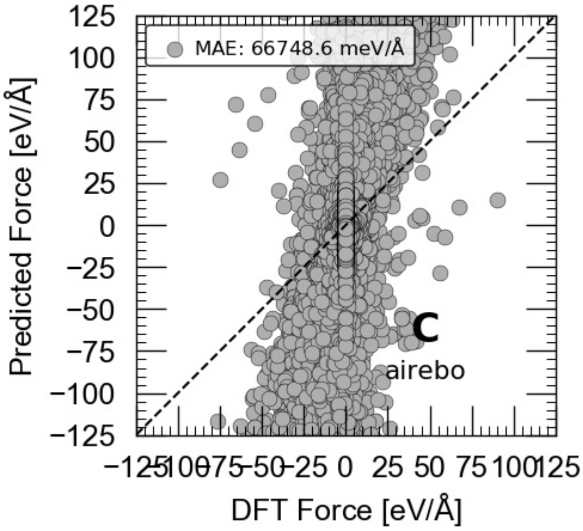

(a)

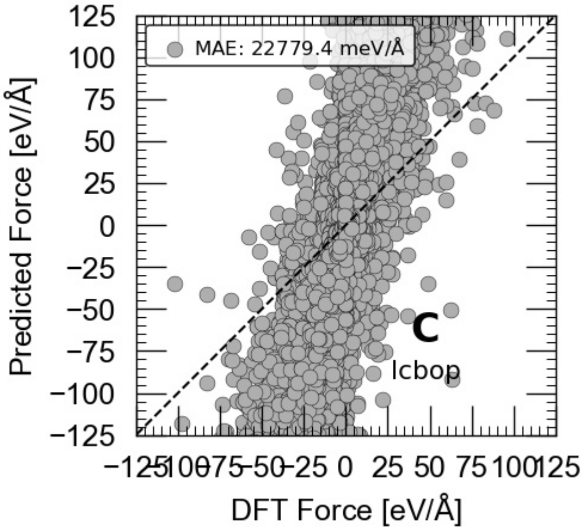

(b)

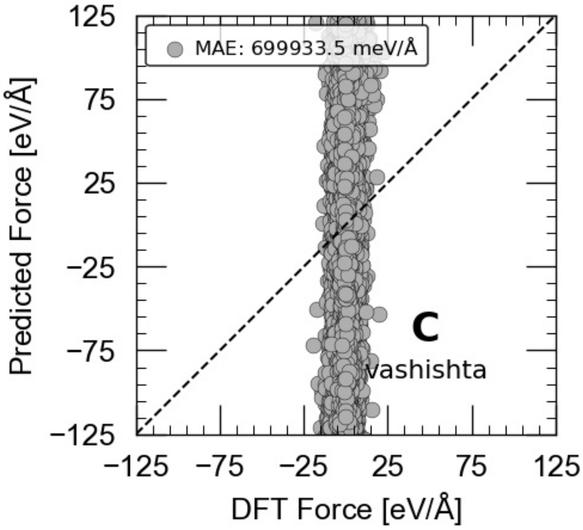

(c)

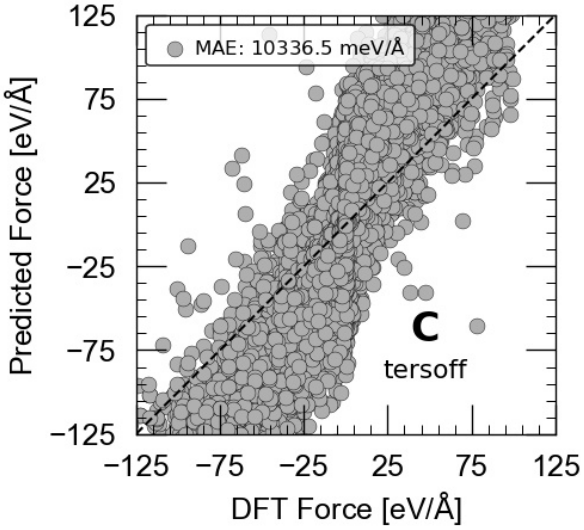

(d)

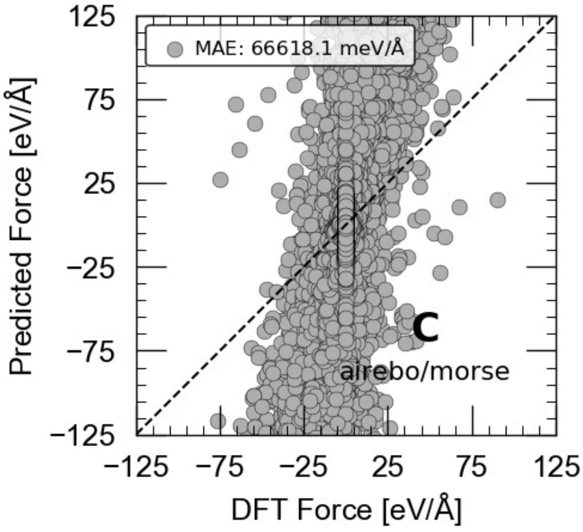
MAE: 66618.1 meV/Å
C
airebo/morse
Predicted Force [eV/Å]
DFT Force [eV/Å]
125
75
25
−25
−75
−125
−125
−75
−25
25
75
125

(a)

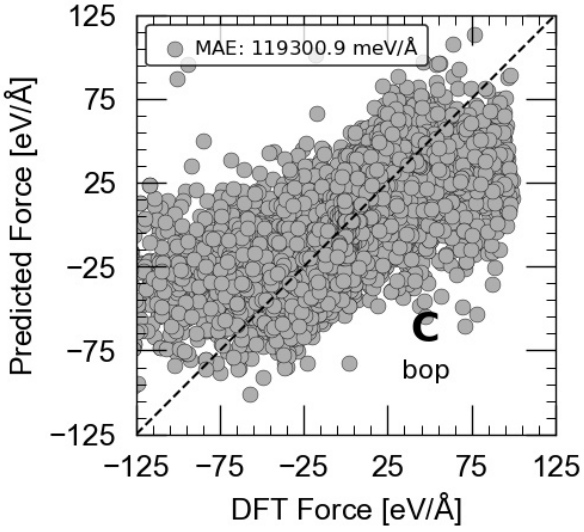
MAE: 119300.9 meV/Å
C
bop
Predicted Force [eV/Å]
DFT Force [eV/Å]
125
75
25
−25
−75
−125
−125
−75
−25
25
75
125

(b)

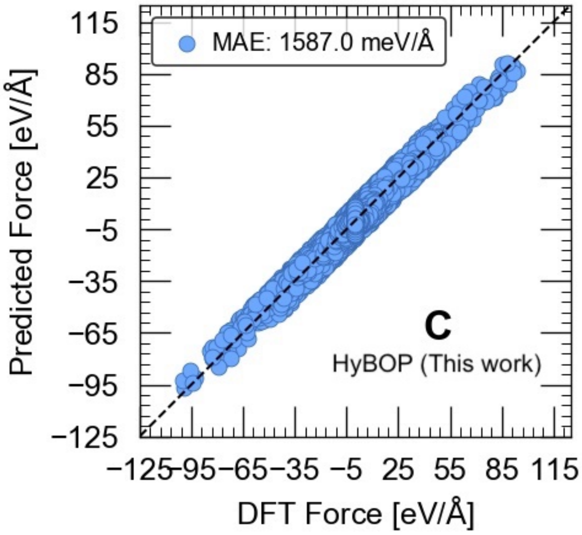
MAE: 1587.0 meV/Å
C
HyBOP (This work)
Predicted Force [eV/Å]
DFT Force [eV/Å]
115
85
55
25
−5
−35
−65
−95
−125
−125
−95
−65
−35
−5
25
55
85
115

(c)

6.9 Cd

6.9.1 Energy Correlation Plots

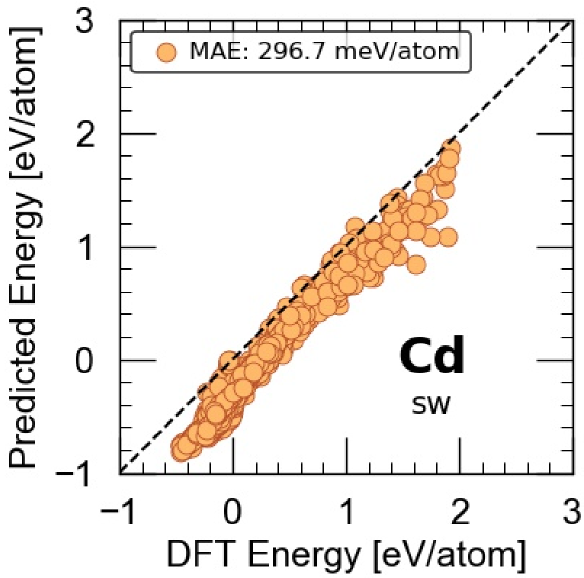

(a)

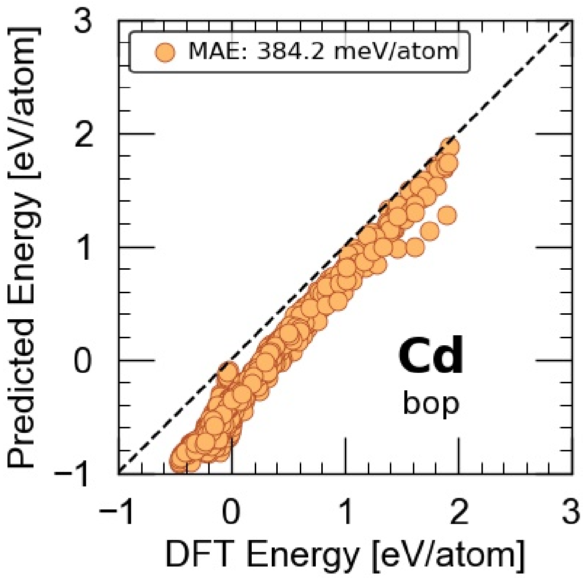

(b)

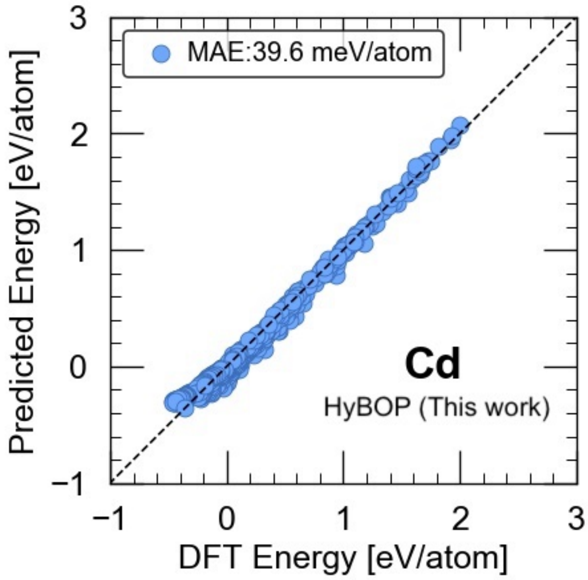

(c)

6.9.2 Force Correlation Plots

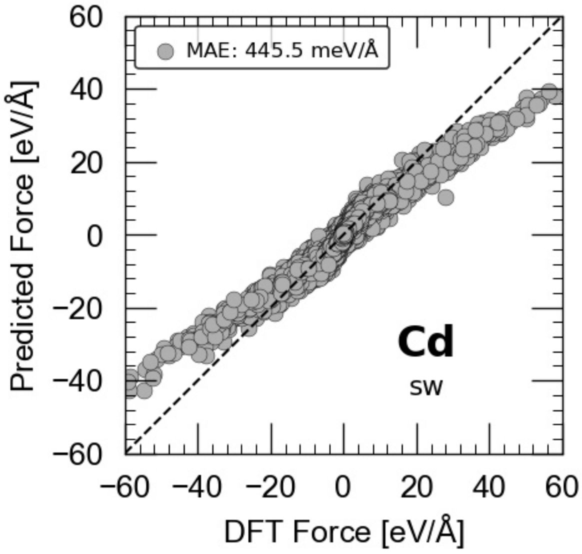

(a)

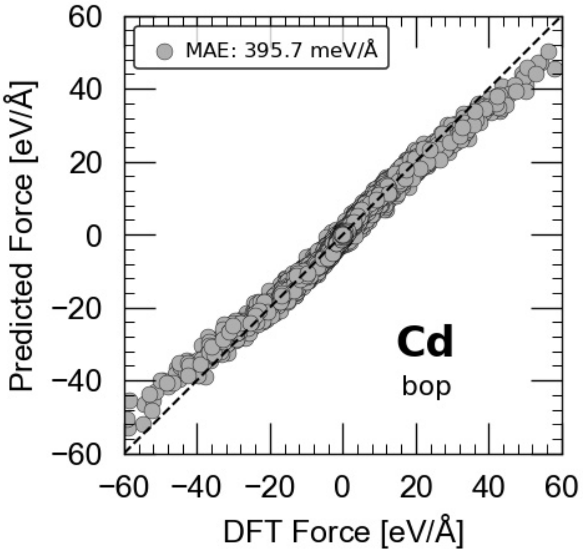

(b)

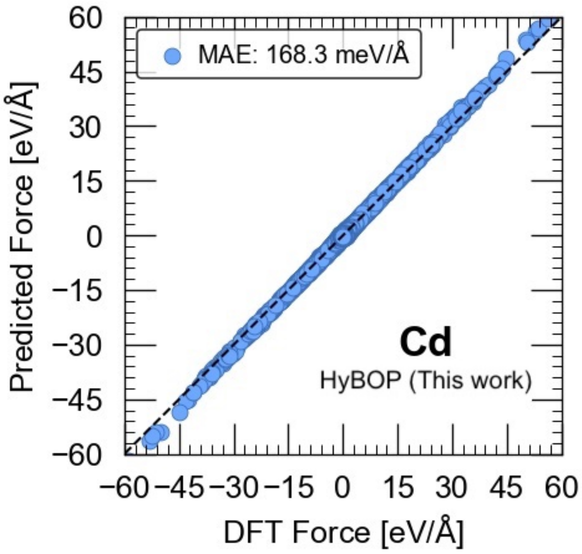

(c)

6.10 Co

6.10.1 Energy Correlation Plots

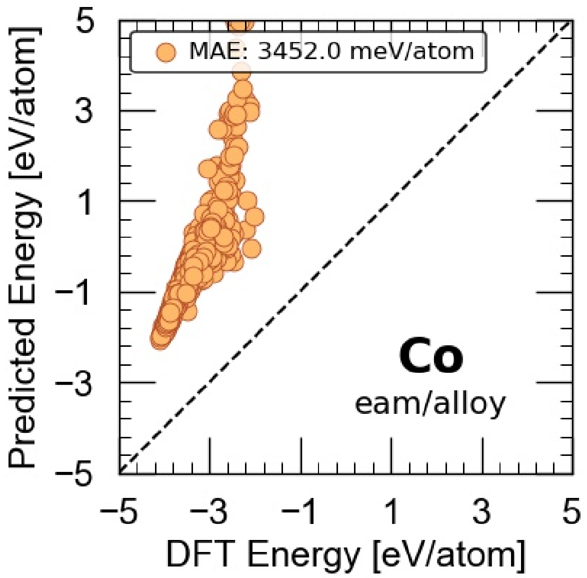

(a)

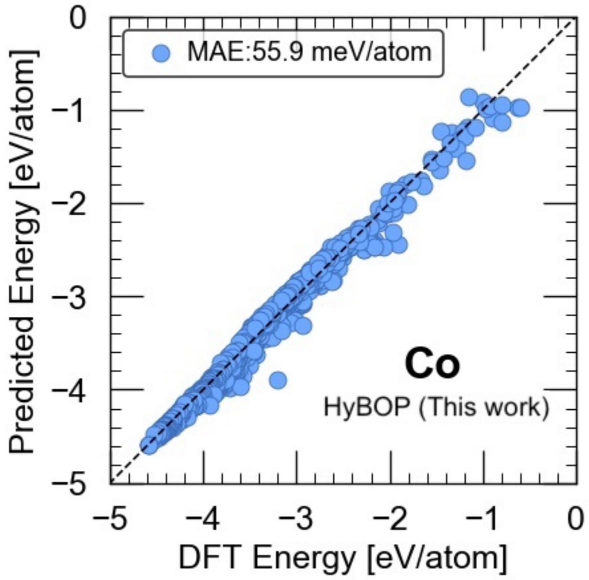

(b)

6.10.2 Force Correlation Plots

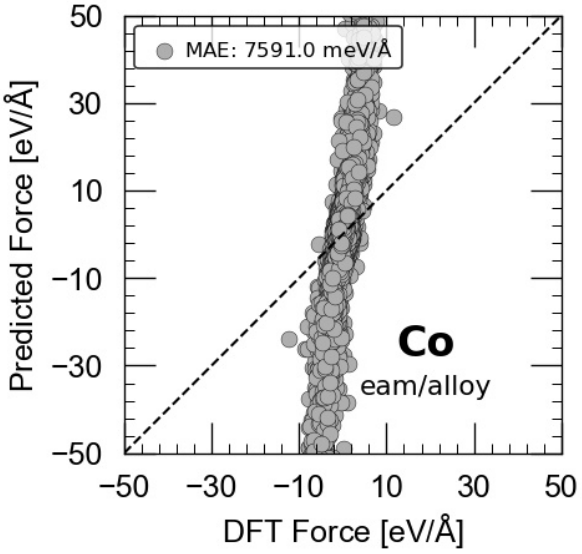

(a)

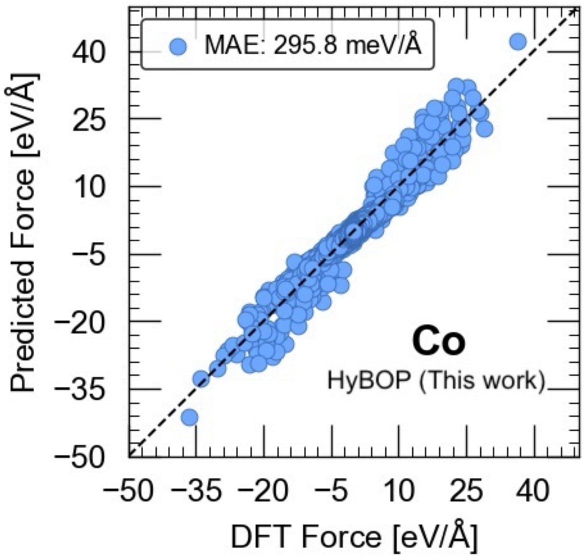

(b)

6.11 Cr

6.11.1 Energy Correlation Plots

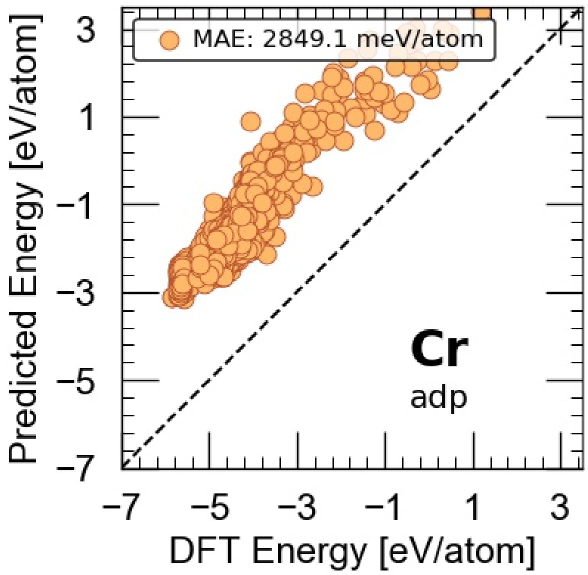

(a)

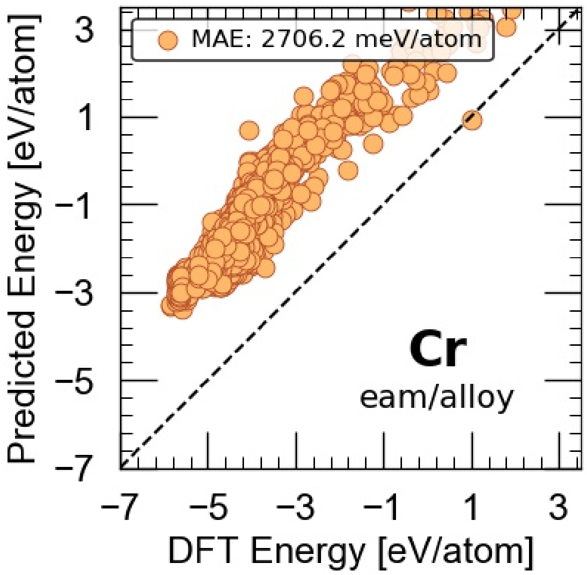

(b)

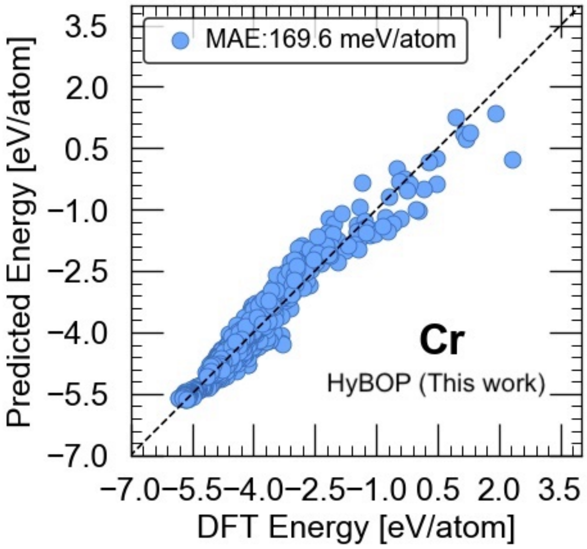

(c)

6.11.2 Force Correlation Plots

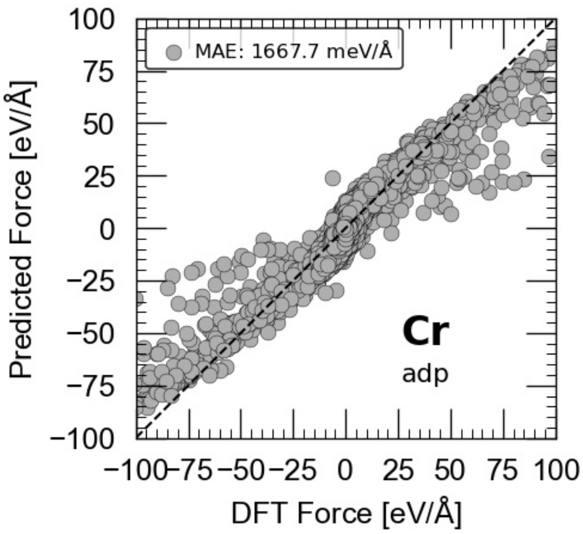

(a)

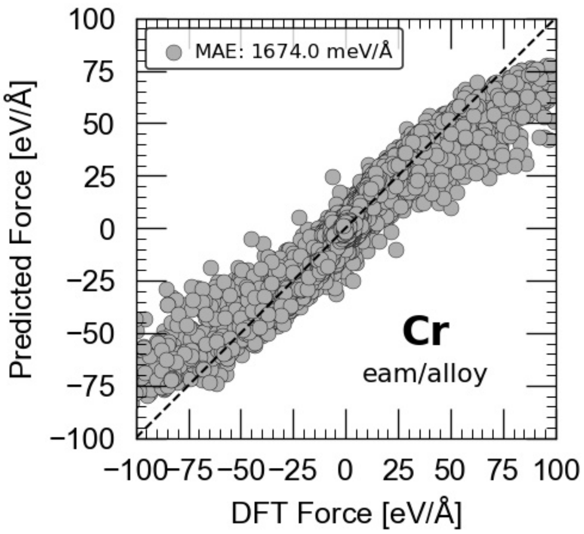

(b)

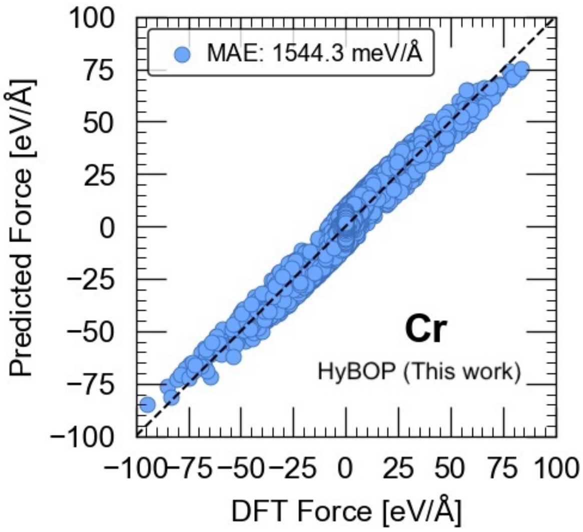

(c)

6.12 Cu

6.12.1 Energy Correlation Plots

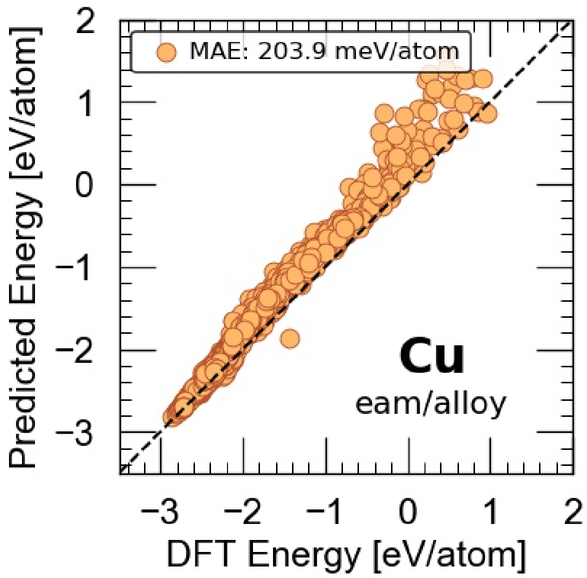

(a)

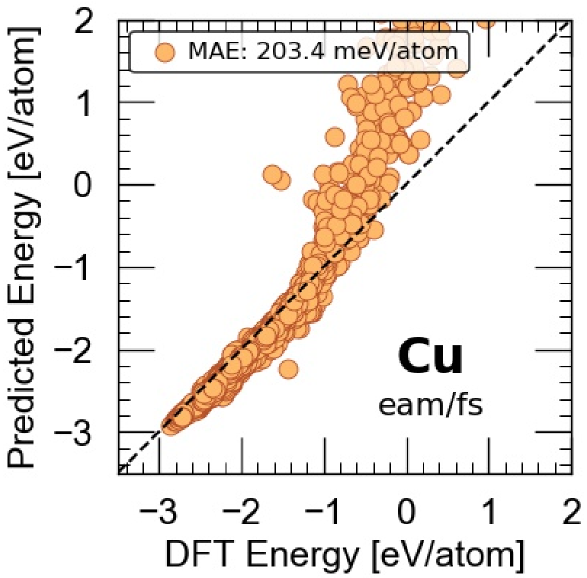

(b)

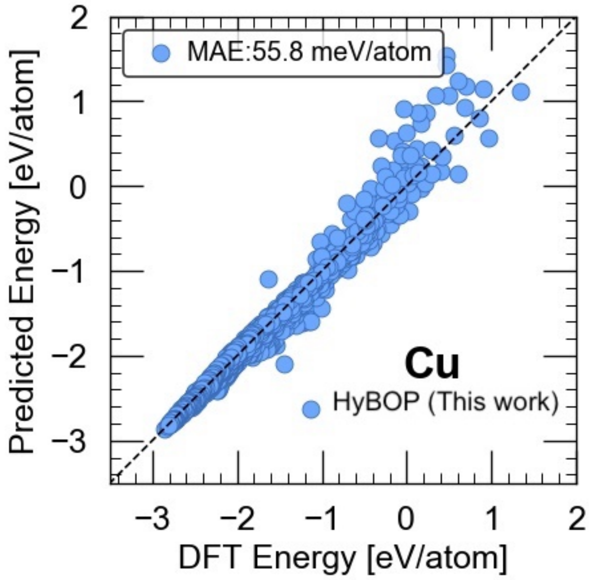

(c)

6.12.2 Force Correlation Plots

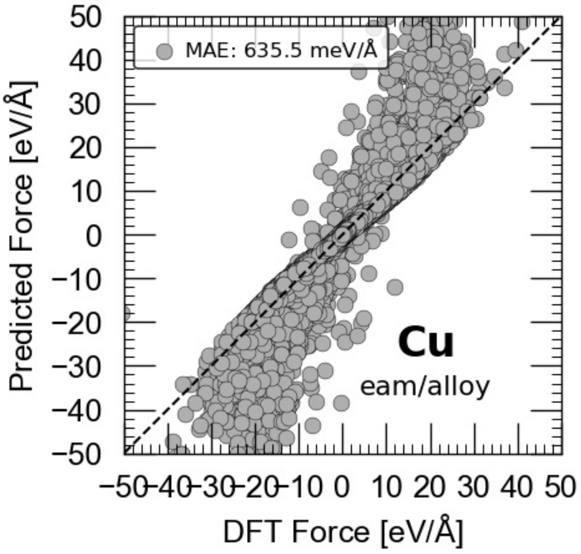

(a)

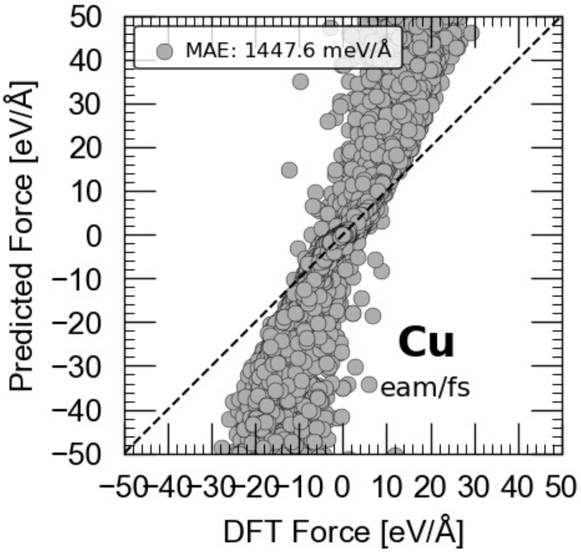

(b)

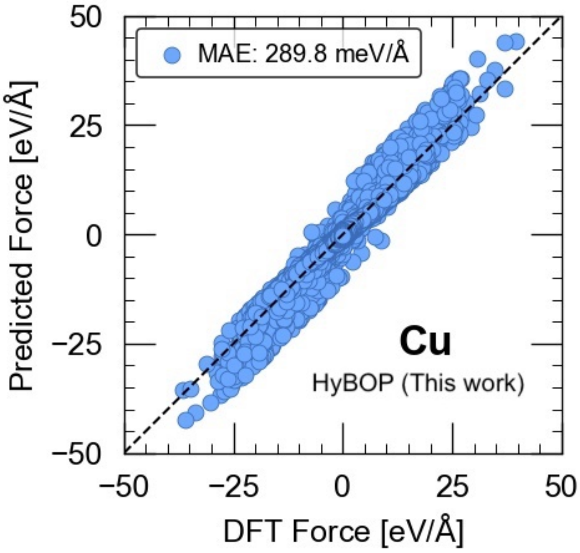

(c)

6.13 Fe

6.13.1 Energy Correlation Plots

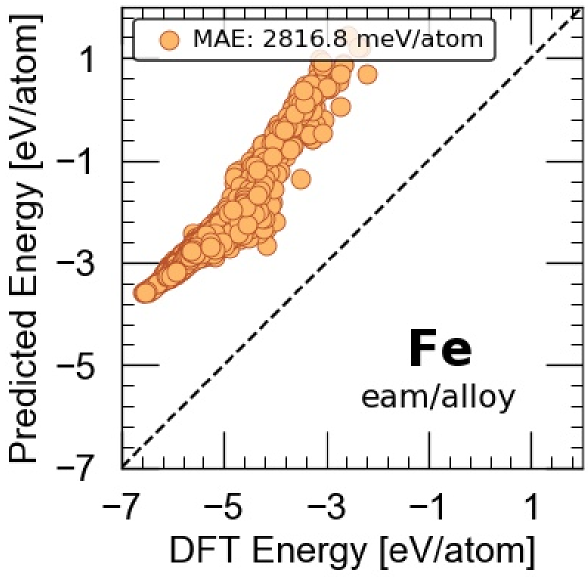

(a)

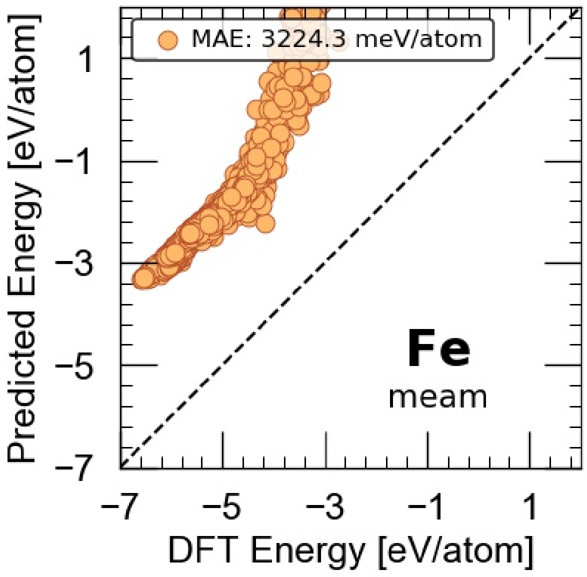

(b)

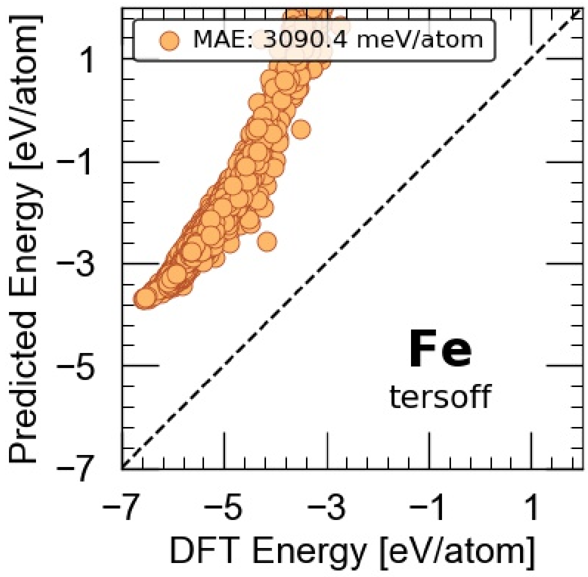

(c)

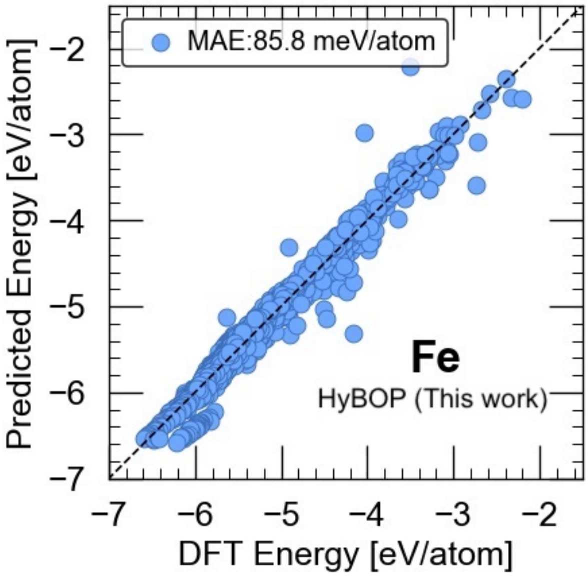

(d)

6.13.2 Force Correlation Plots

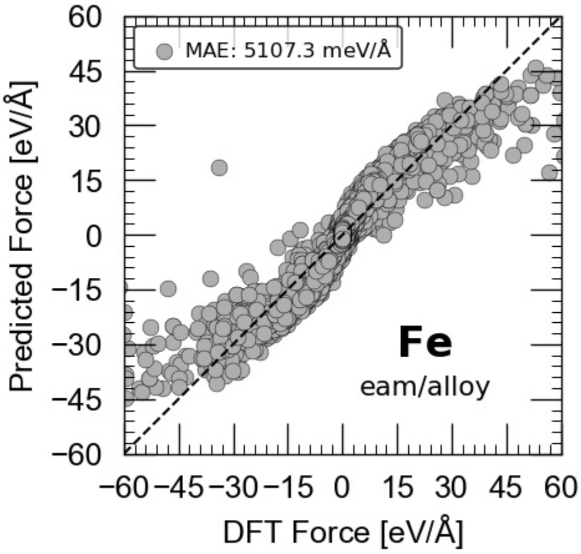

(a)

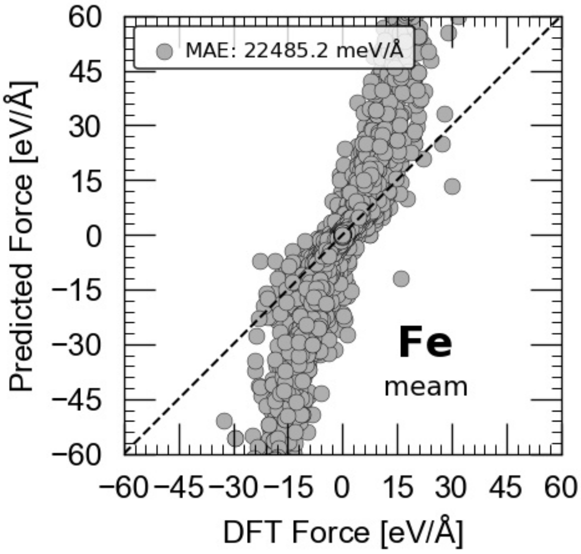

(b)

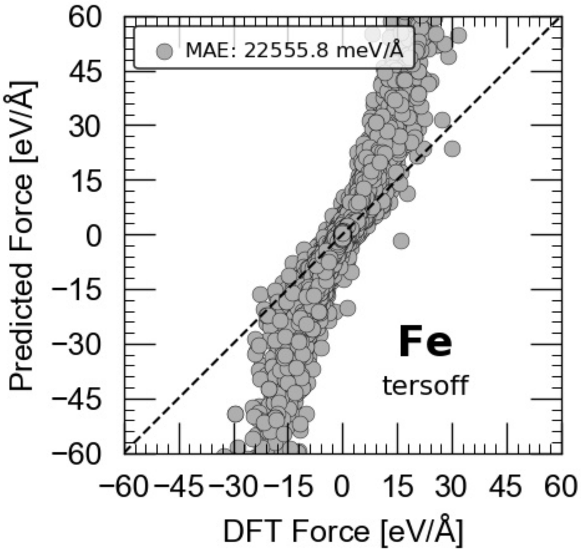

(c)

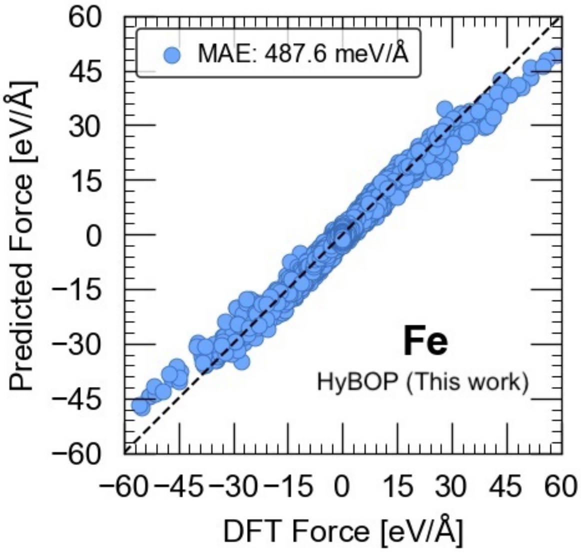

(d)

6.14 Ga

6.14.1 Energy Correlation Plots

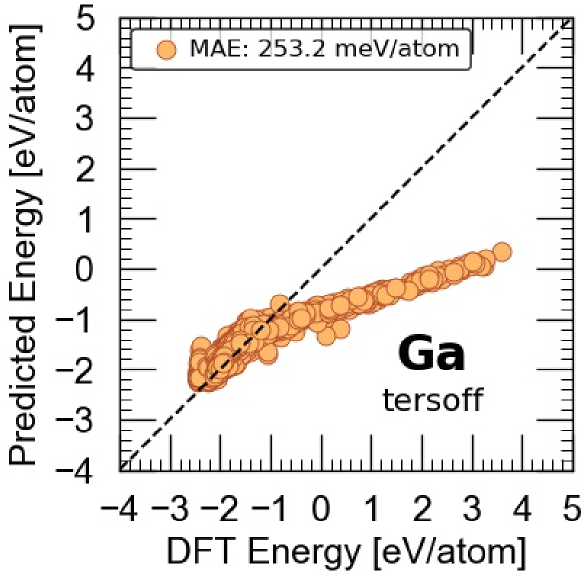

(a)

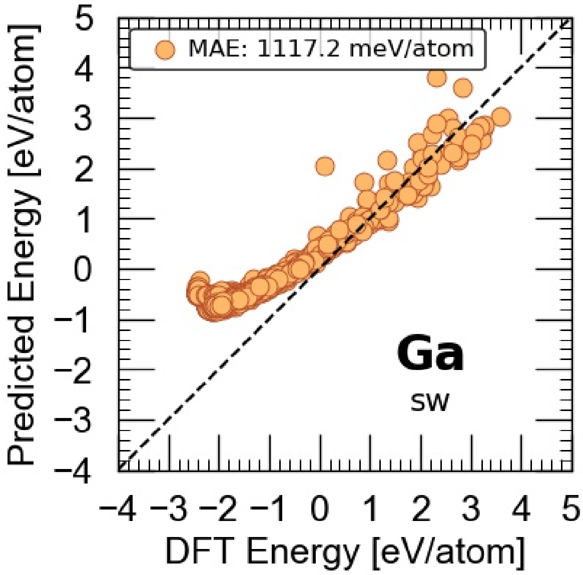

(b)

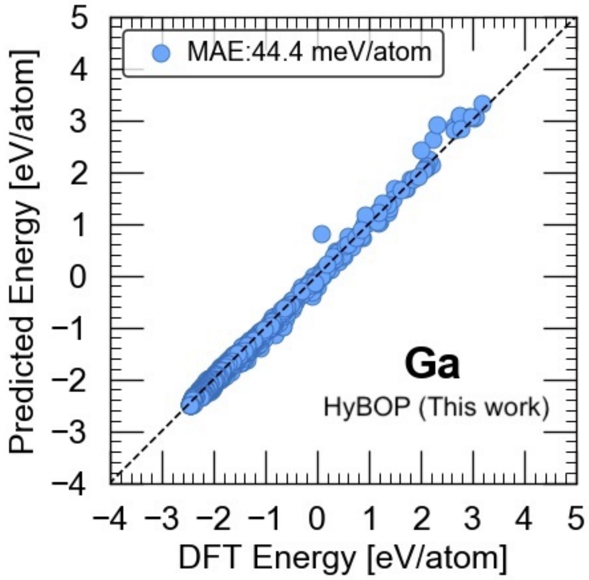

(c)

6.14.2 Force Correlation Plots

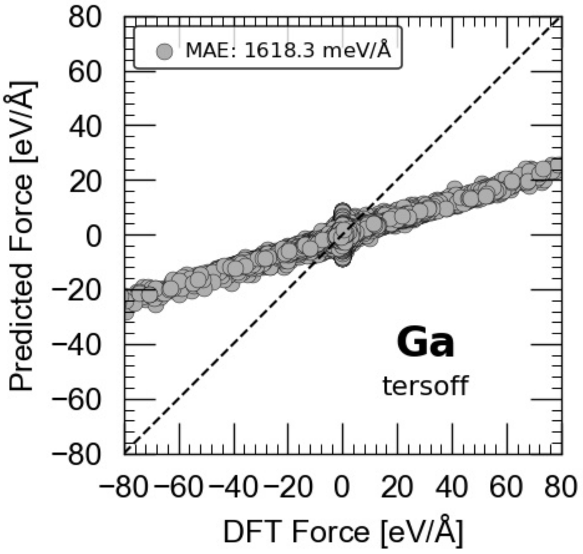

(a)

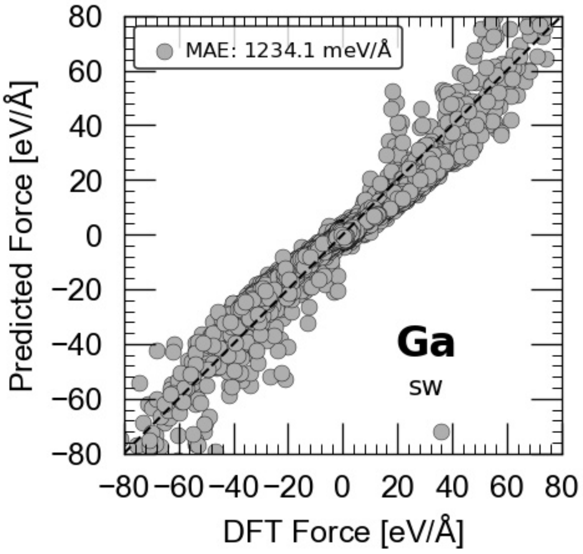

(b)

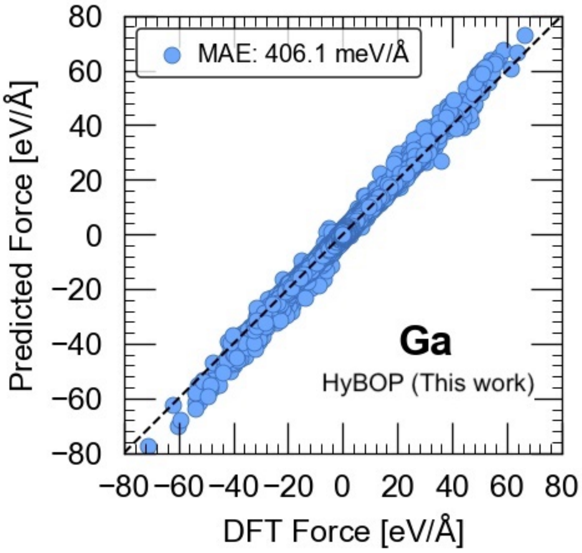

(c)

6.15 Ge

6.15.1 Energy Correlation Plots

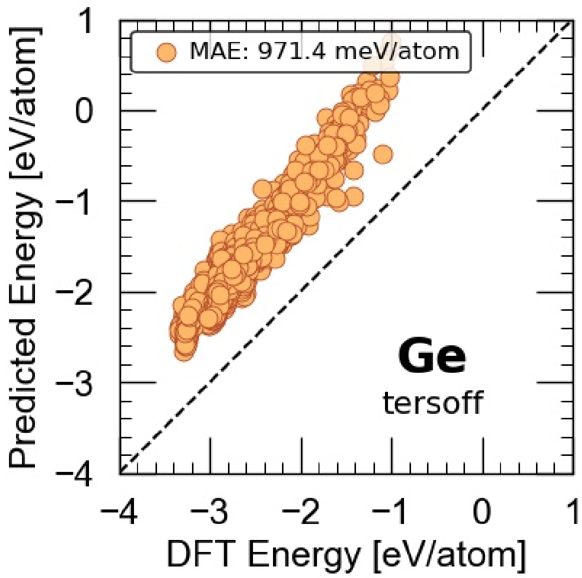

(a)

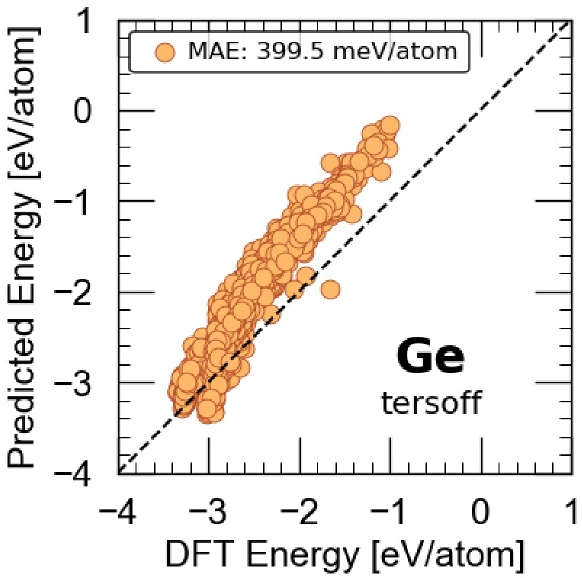

(b)

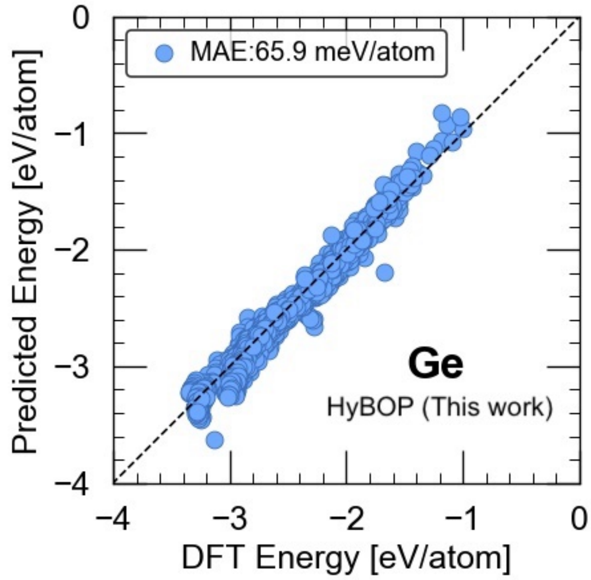

(c)

6.15.2 Force Correlation Plots

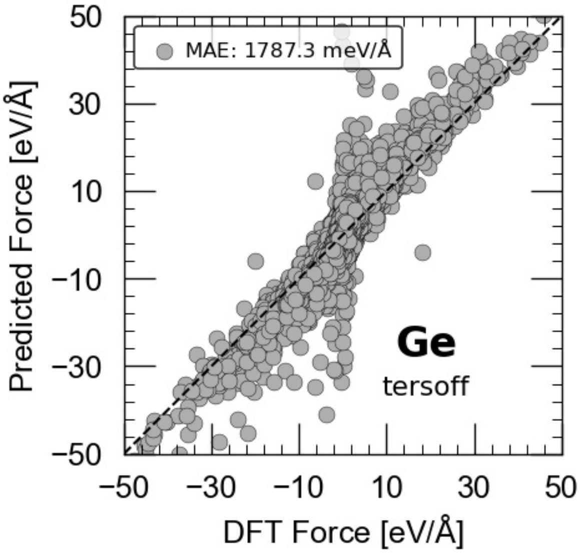

(a)

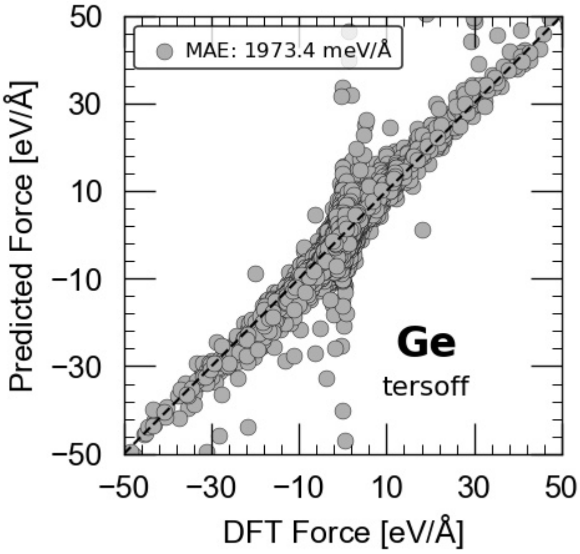

(b)

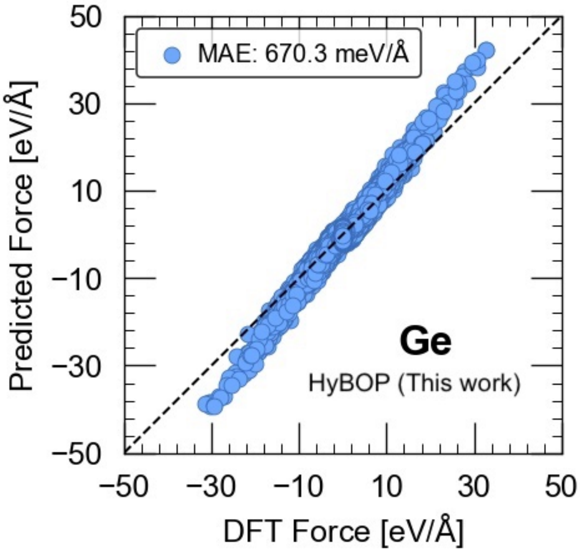

(c)

6.16 K

6.16.1 Energy Correlation Plots

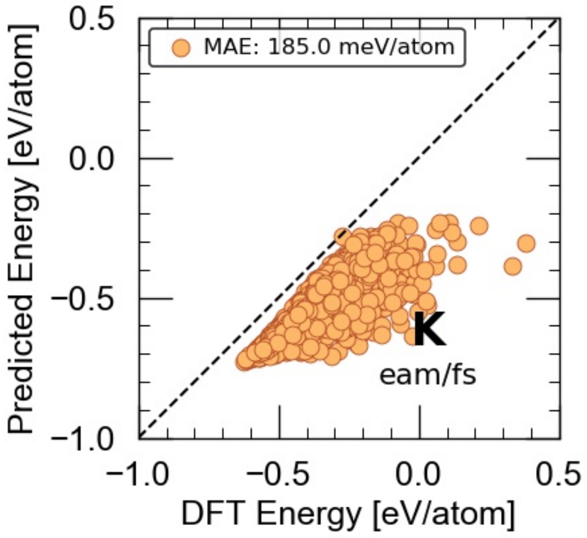

(a)

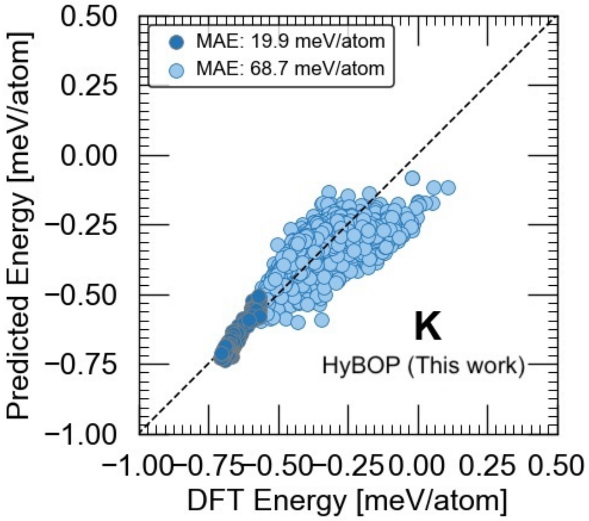

(b)

6.16.2 Force Correlation Plots

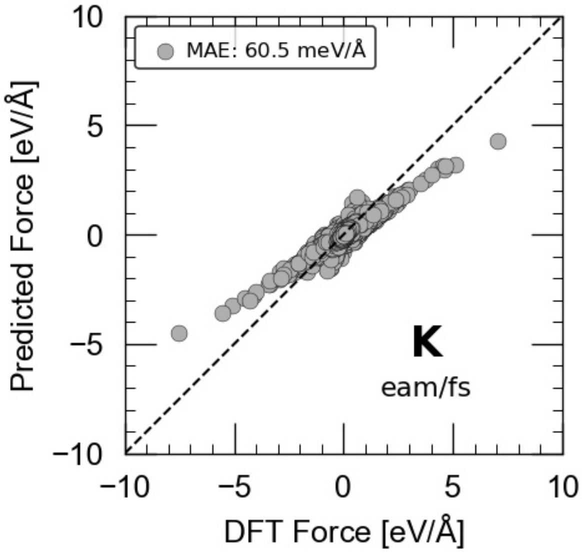

(a)

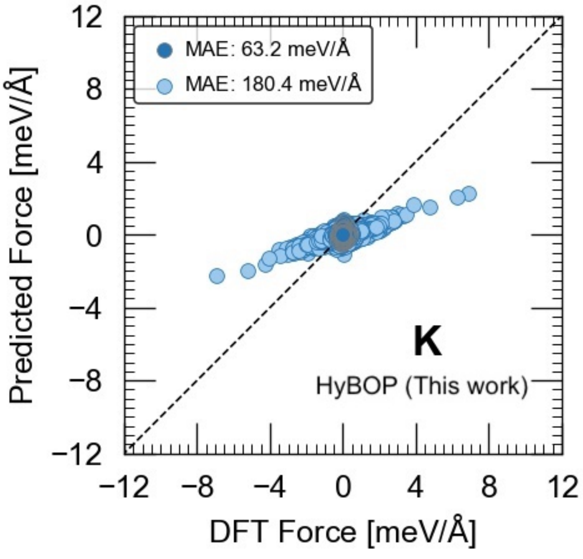

(b)

6.17 Li

6.17.1 Energy Correlation Plots

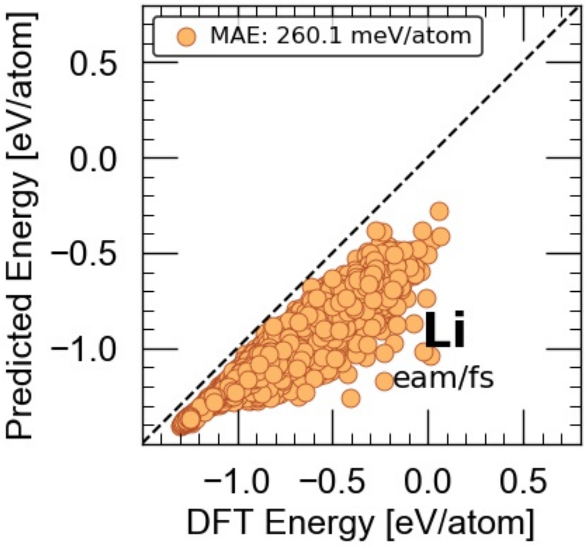

(a)

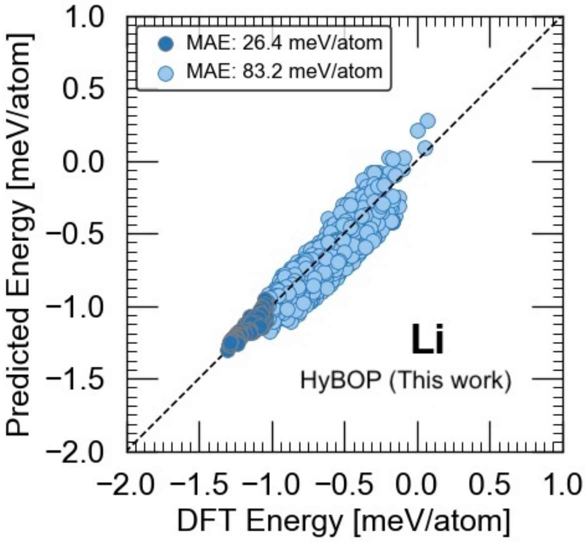

(b)

6.17.2 Energy Correlation Plots

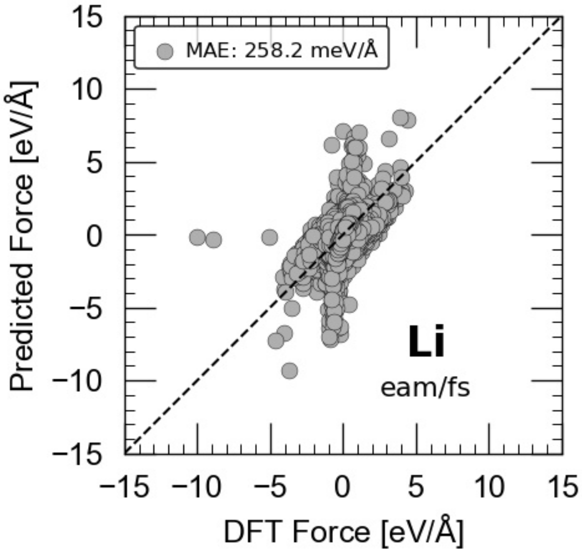

(a)

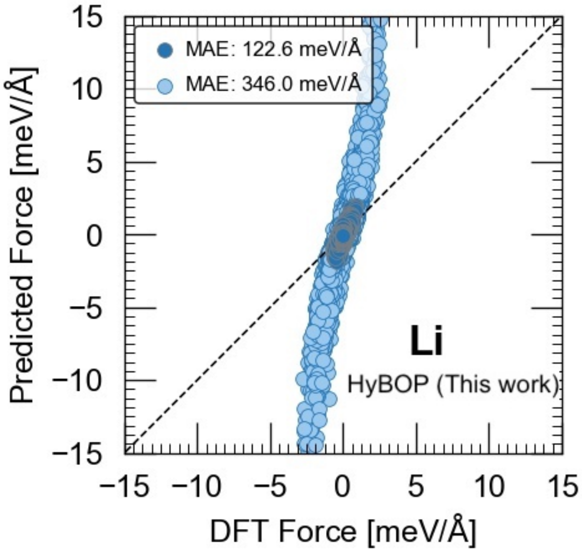

(b)

6.18 Mg

6.18.1 Energy Correlation Plots

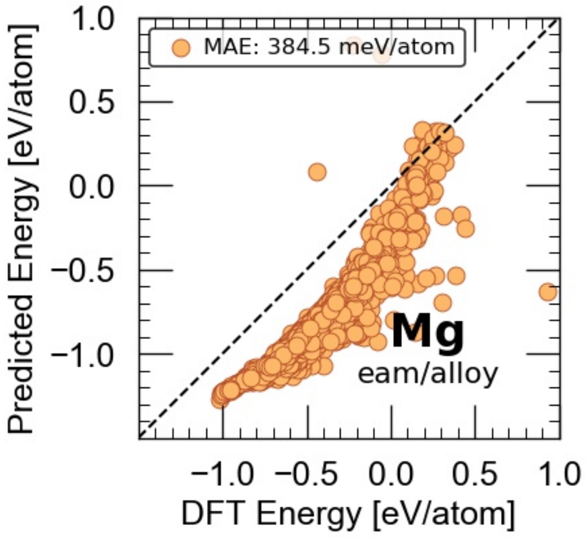

(a)

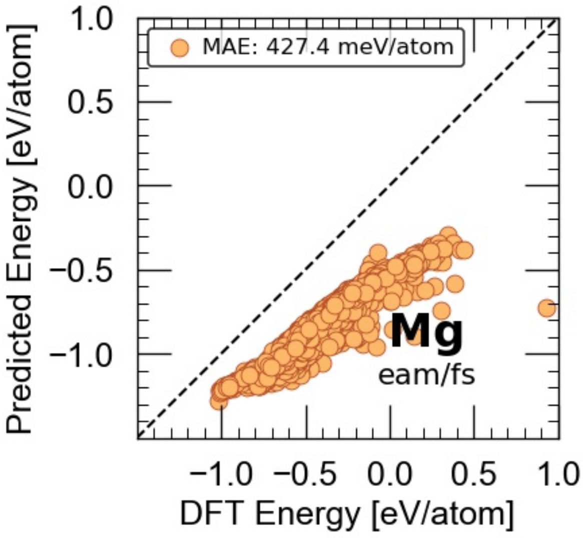

(b)

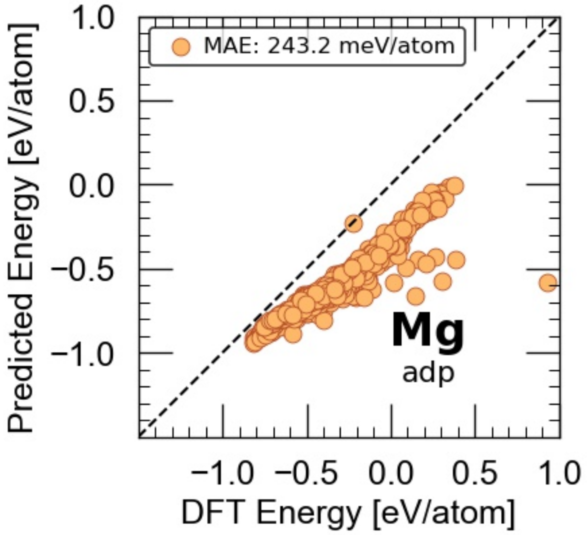

(c)

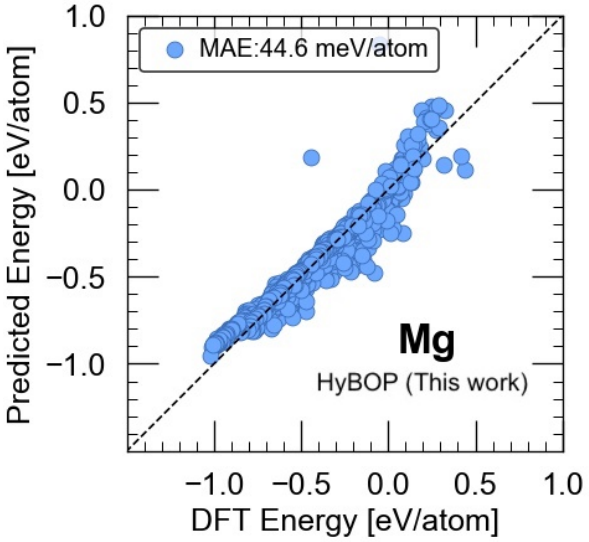

(d)

6.18.2 Force Correlation Plots

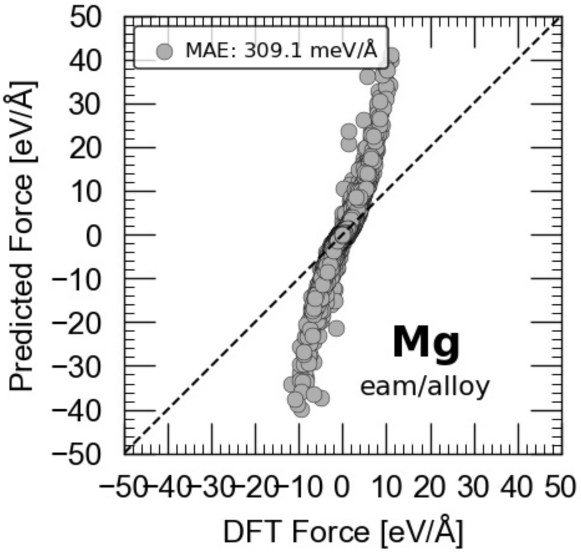

(a)

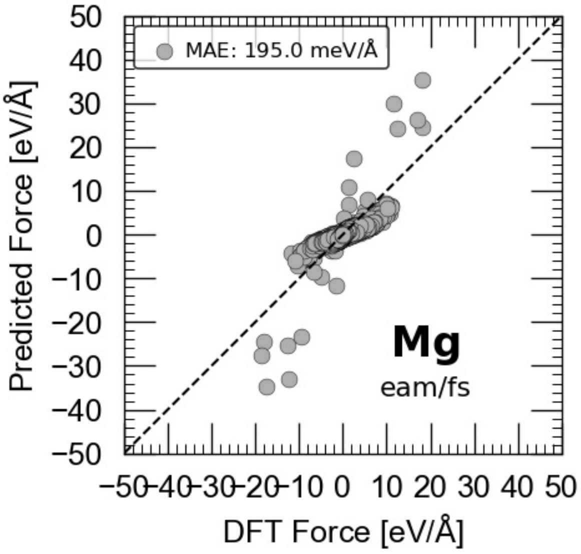

(b)

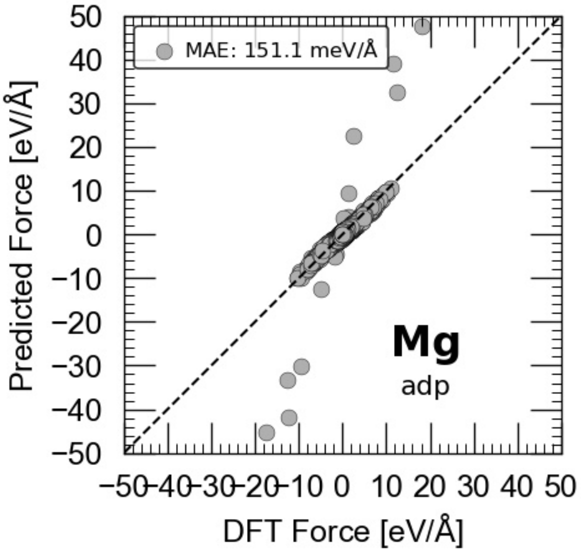

(c)

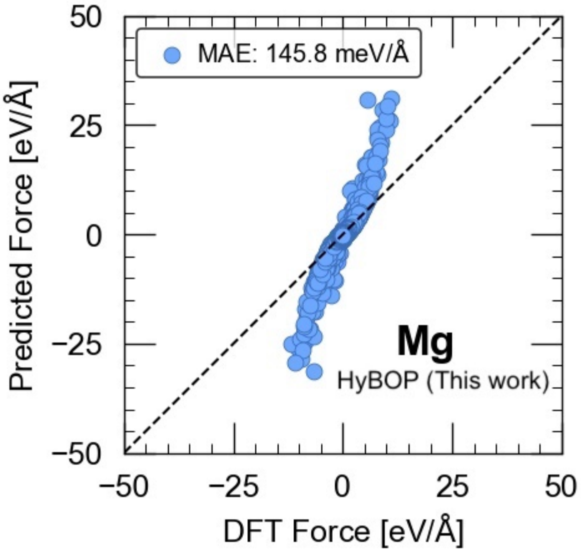

(d)

6.19 Mo

6.19.1 Energy Correlation Plots

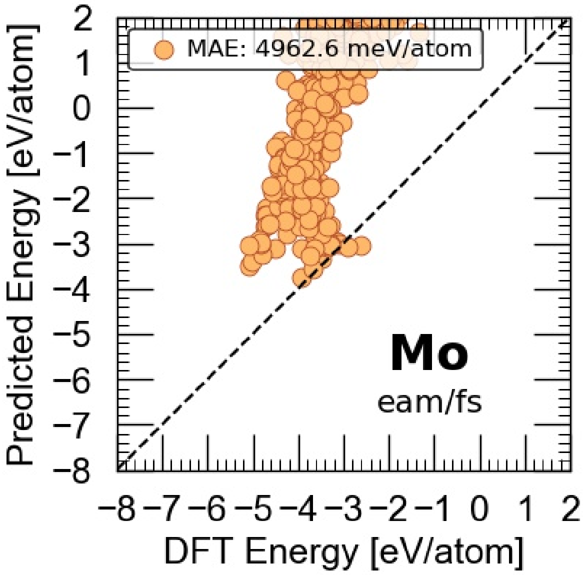

(a)

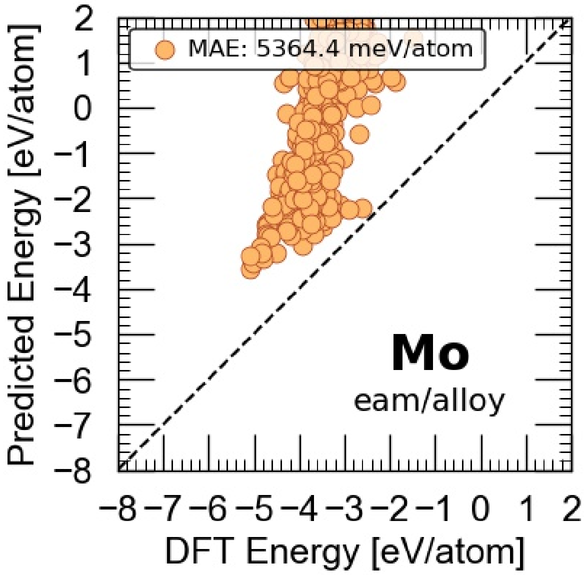

(b)

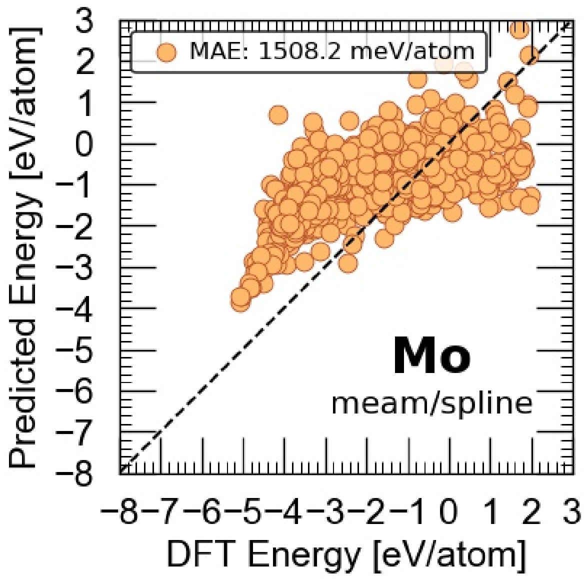

(c)

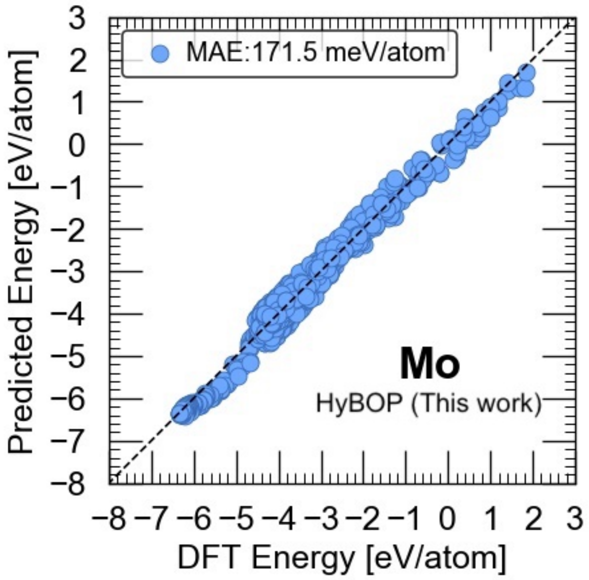

(d)

6.19.2 Force Correlation Plots

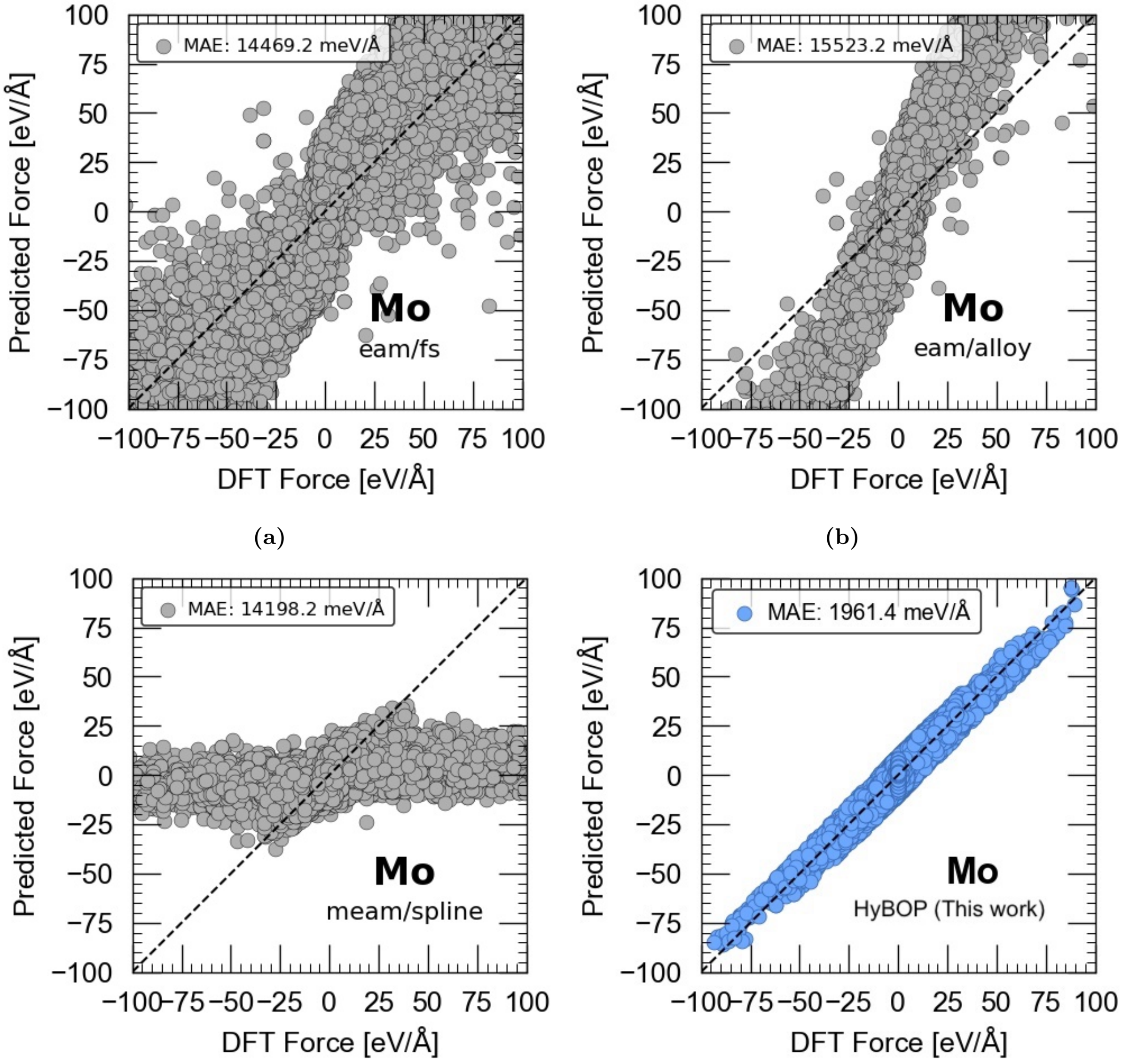

(a) (b)

(c) (d)

6.20 Na

6.20.1 Energy Correlation Plots

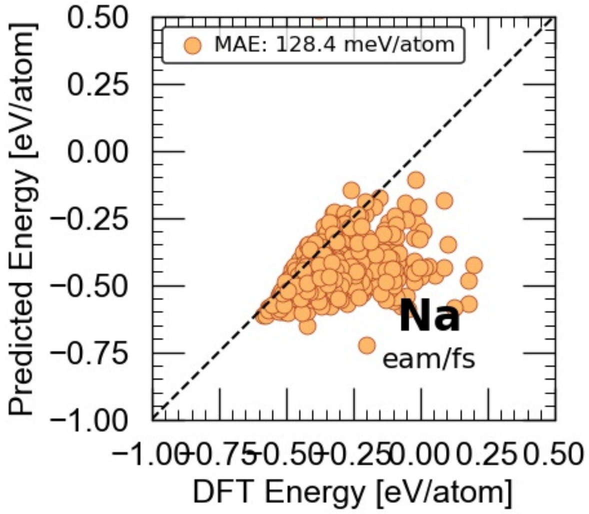

(a)

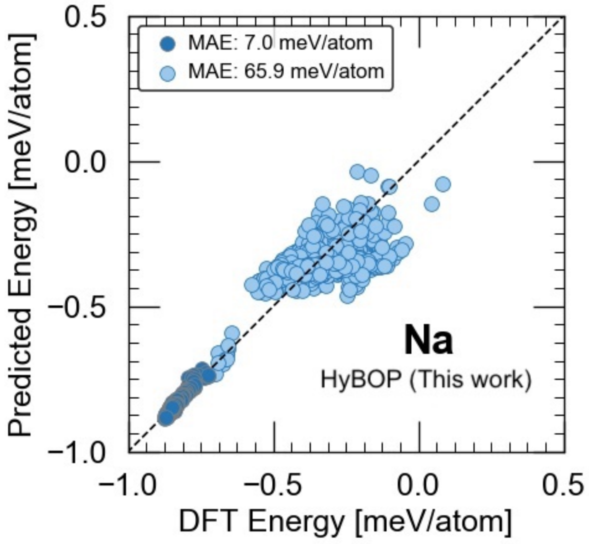

(b)

6.20.2 Force Correlation Plots

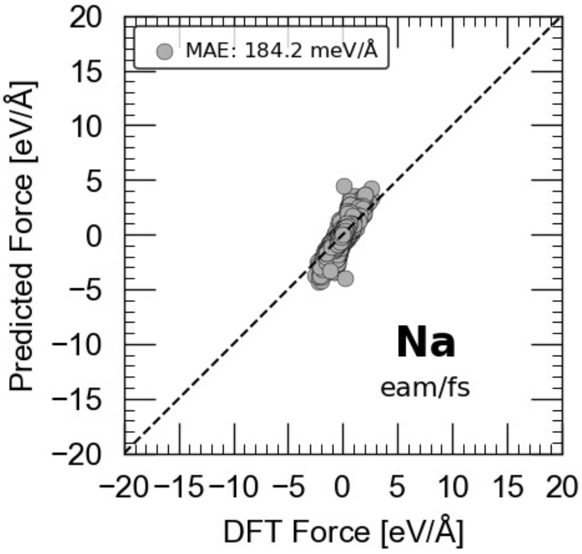

(a)

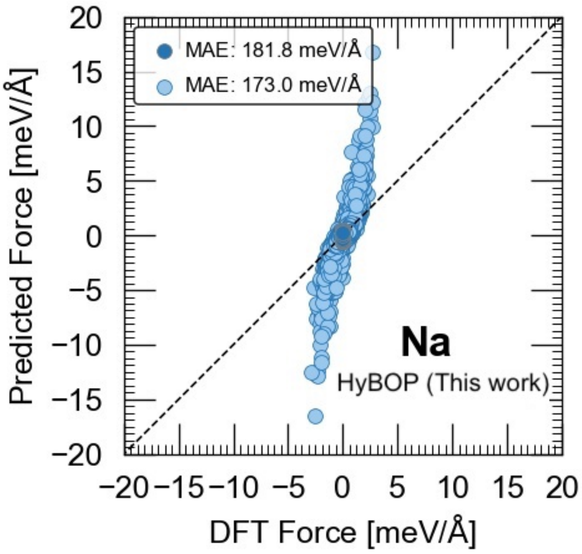

(b)

6.21 Nb

6.21.1 Energy Correlation Plots

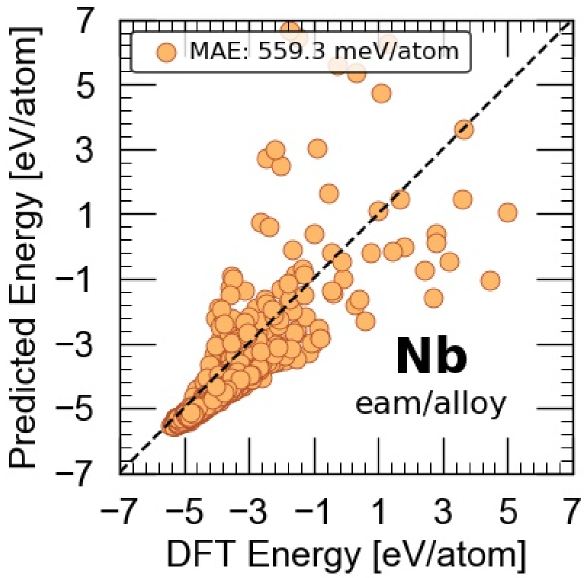

(a)

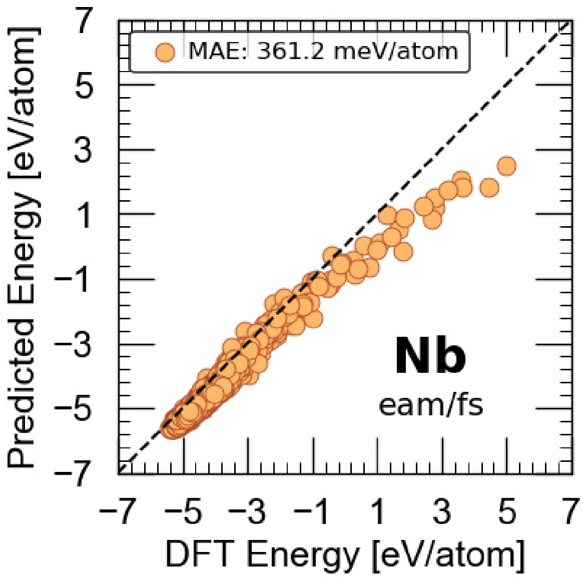

(b)

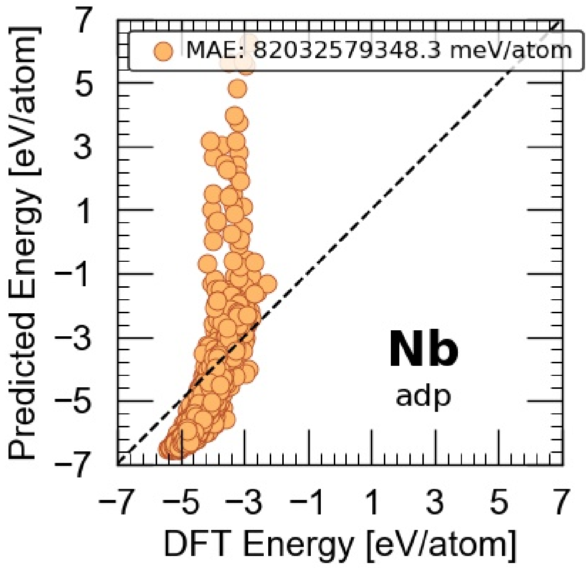

(c)

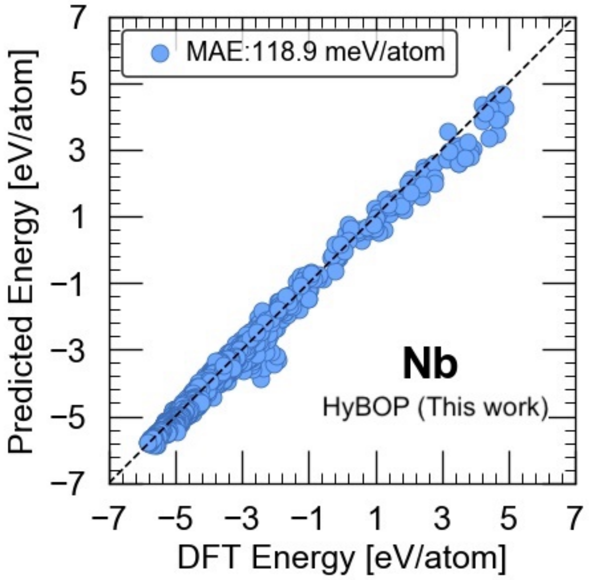

(d)

6.21.2 Force Correlation Plots

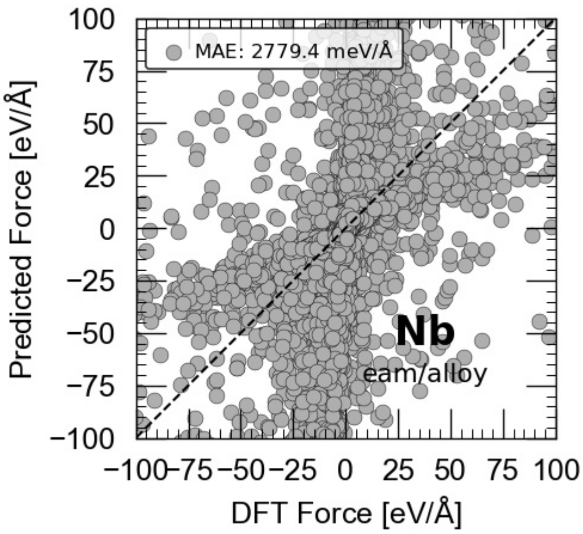

(a)

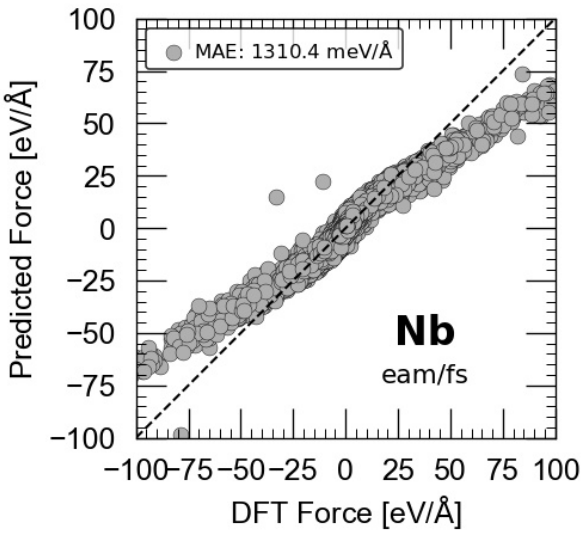

(b)

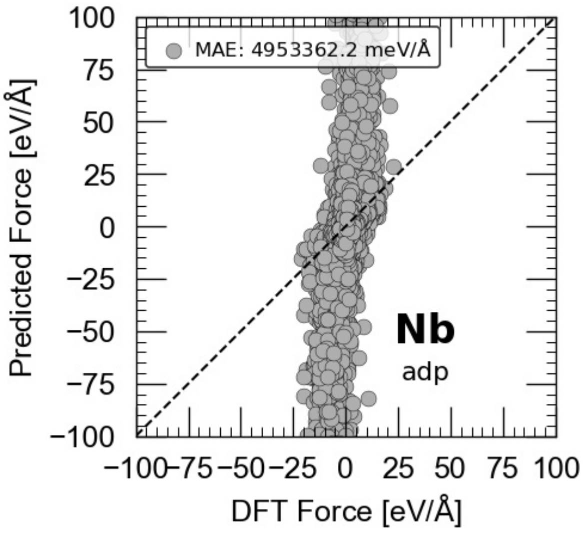

(c)

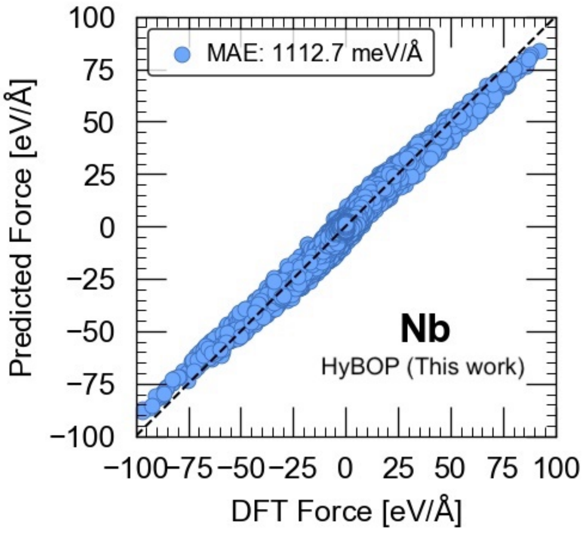

(d)

6.22 Ni

6.22.1 Energy Correlation Plots

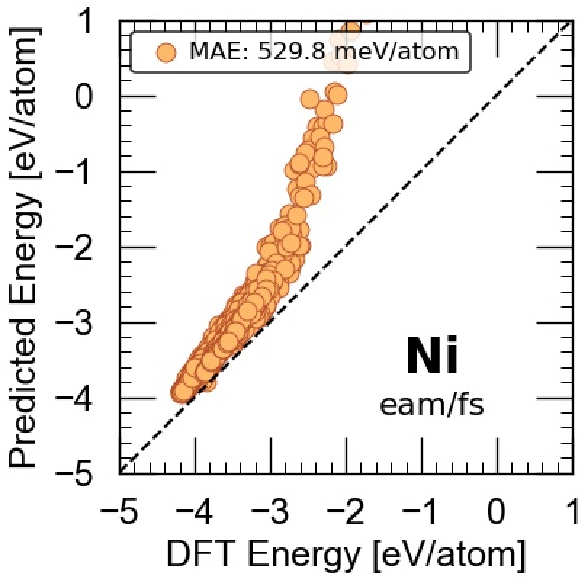

(a)

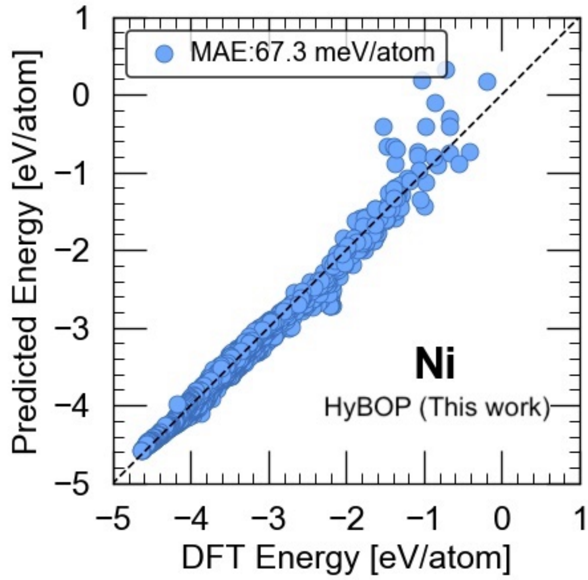

(b)

6.22.2 Force Correlation Plots

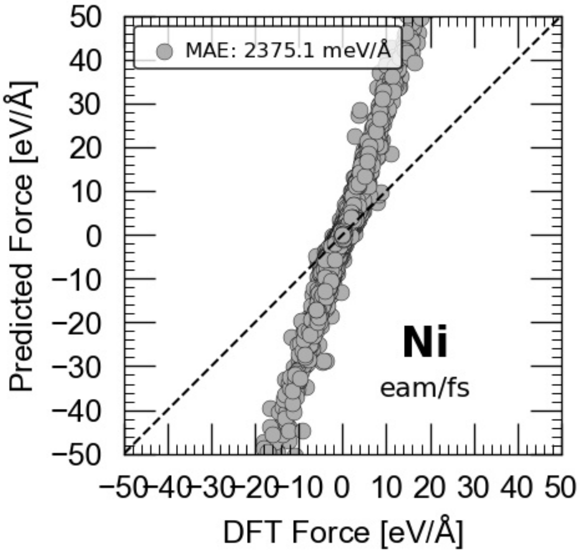

(a)

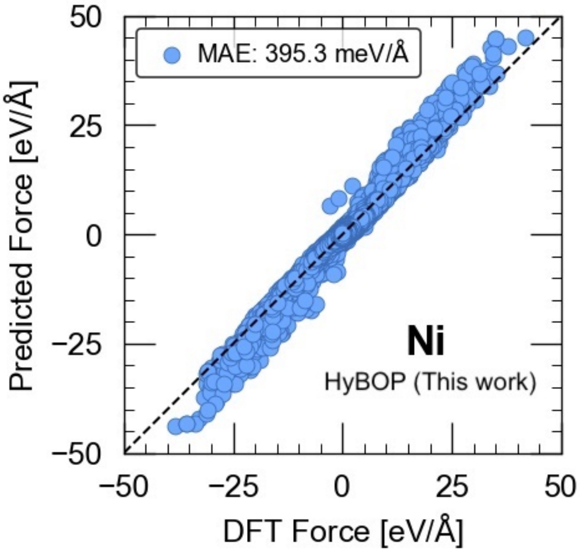

(b)

6.23 P

6.23.1 Energy Correlation Plots

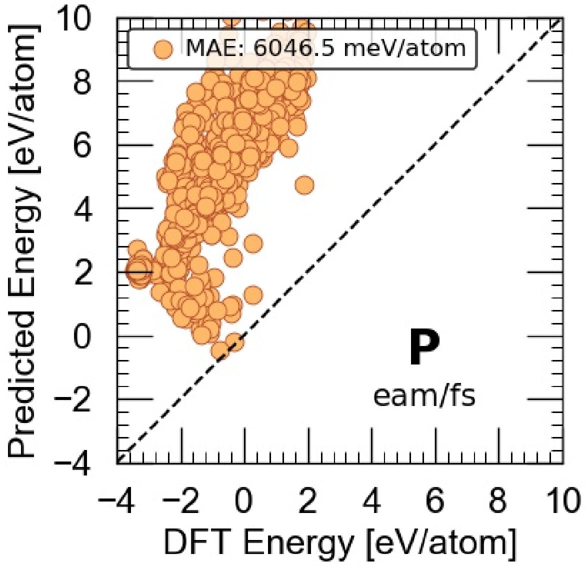

(a)

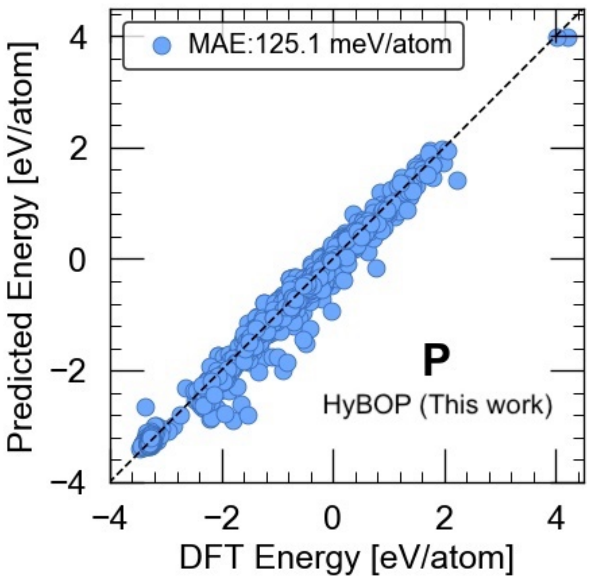

(b)

6.23.2 Force Correlation Plots

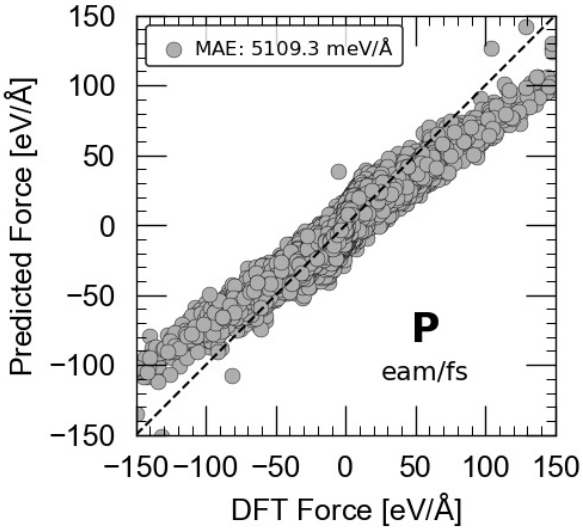

(a)

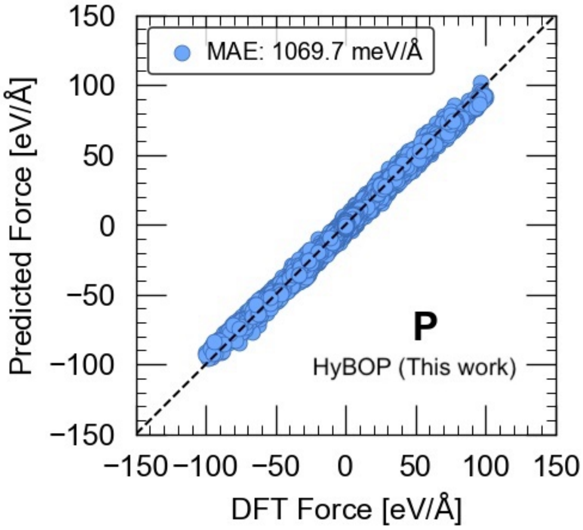

(b)

6.24 Pb

6.24.1 Energy Correlation Plots

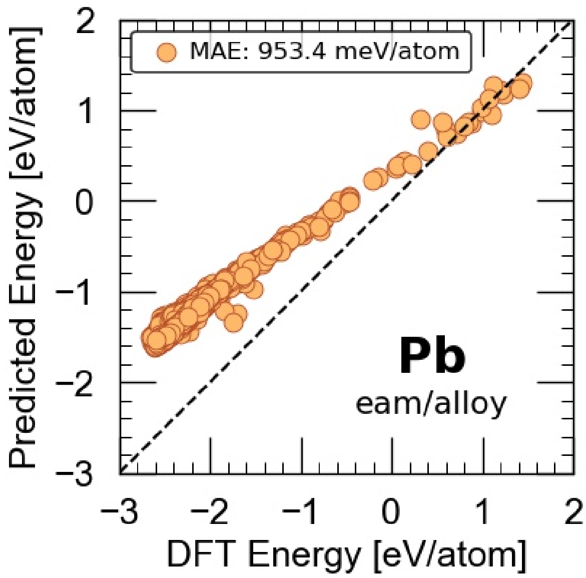

(a)

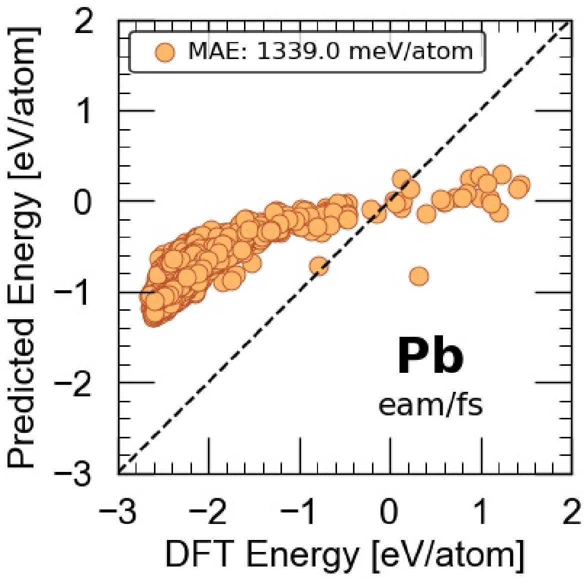

(b)

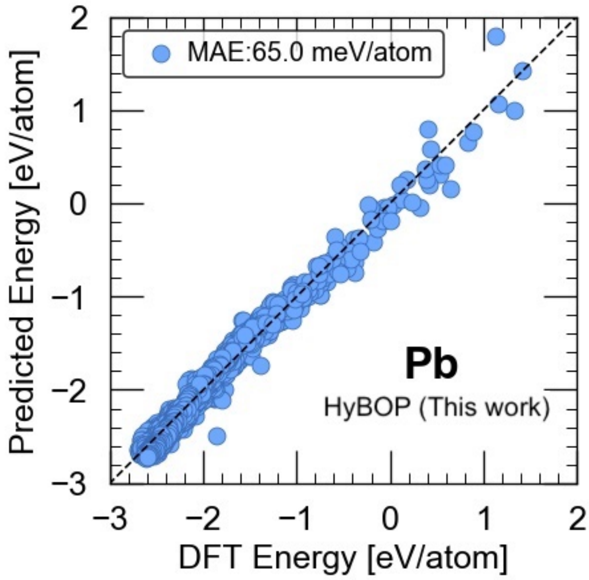

(c)

6.24.2 Force Correlation Plots

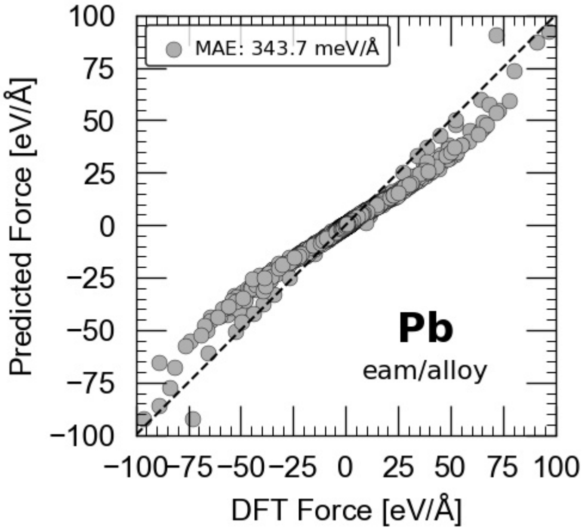

(a)

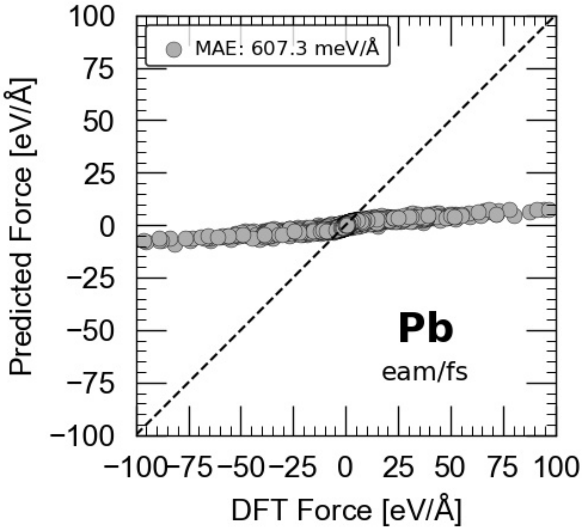

(b)

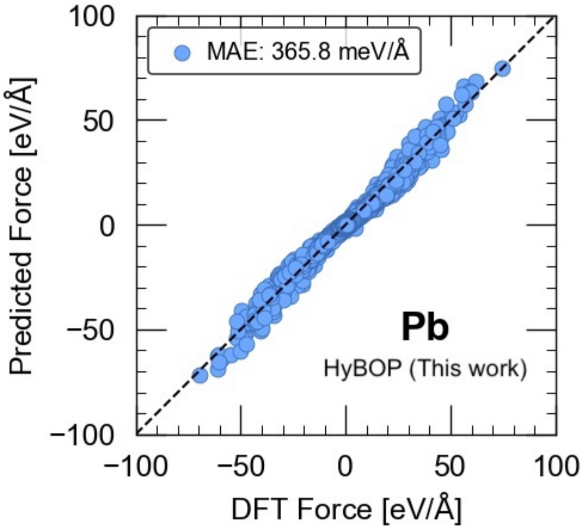

(c)

6.25 Pd

6.25.1 Energy Correlation Plots

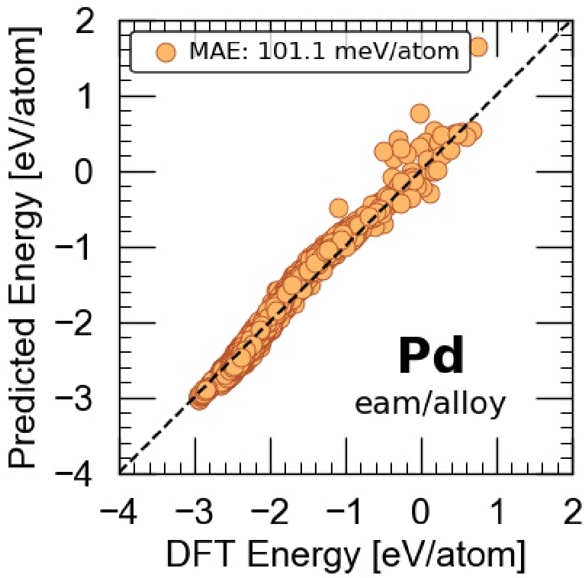

(a)

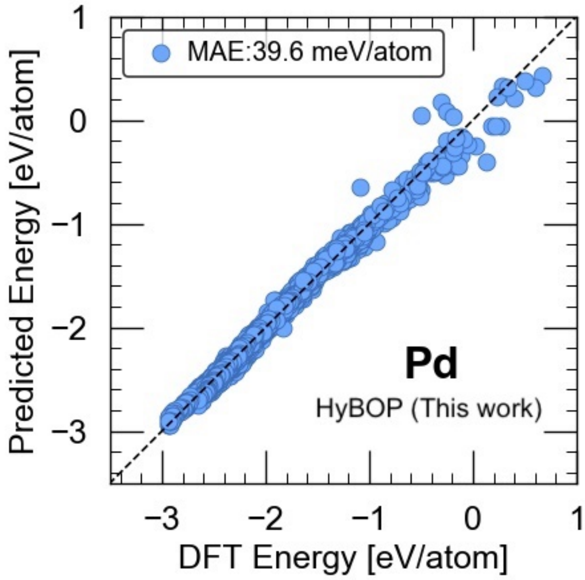

(b)

6.25.2 Force Correlation Plots

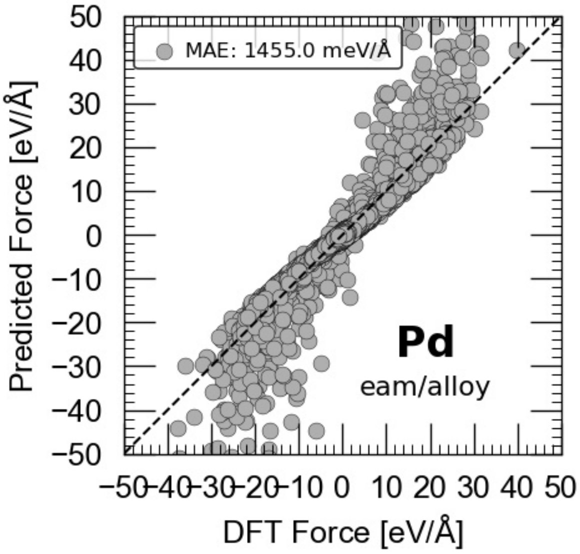

(a)

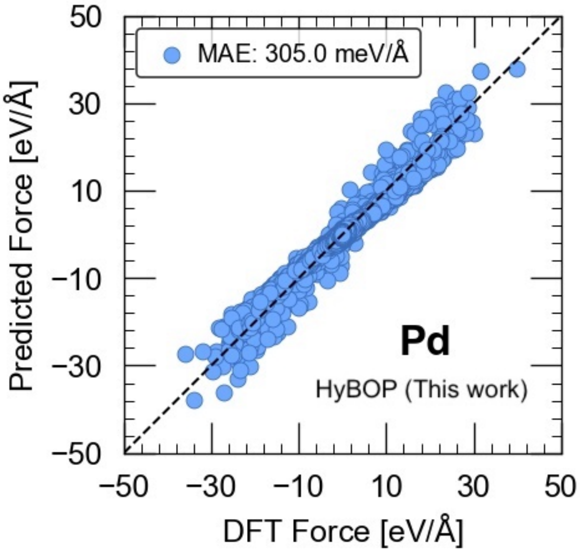

(b)

6.26 Pt

6.26.1 Energy Correlation Plots

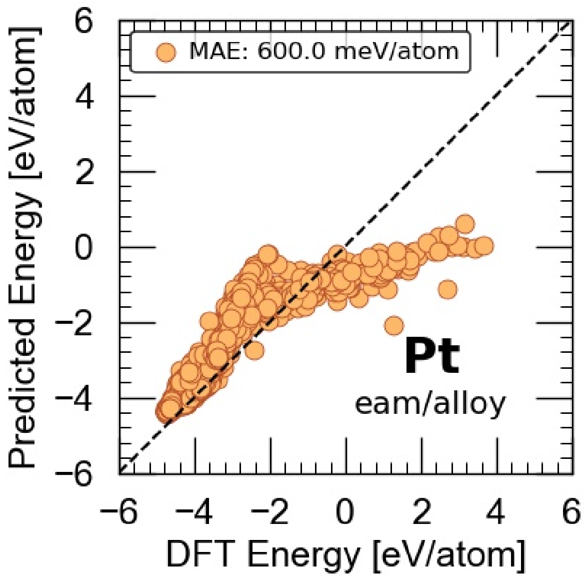

(a)

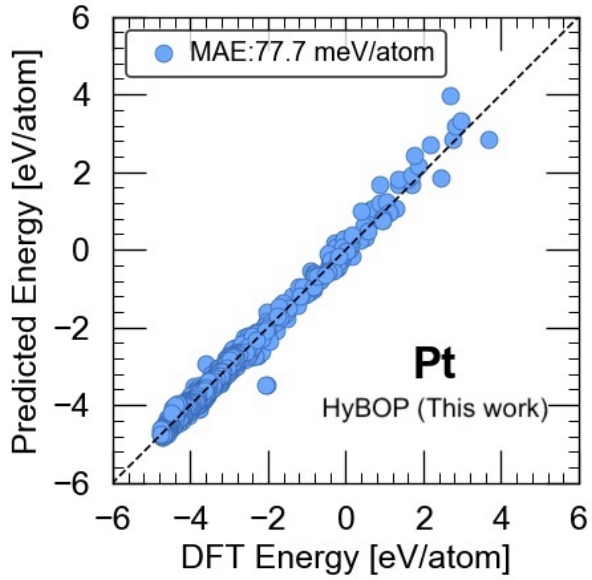

(b)

6.26.2 Force Correlation Plots

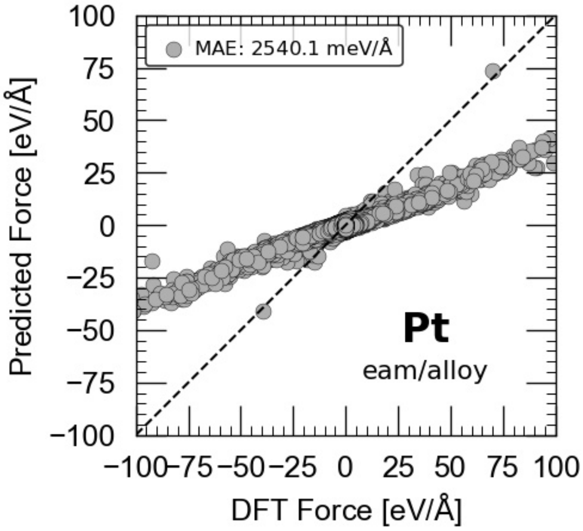

(a)

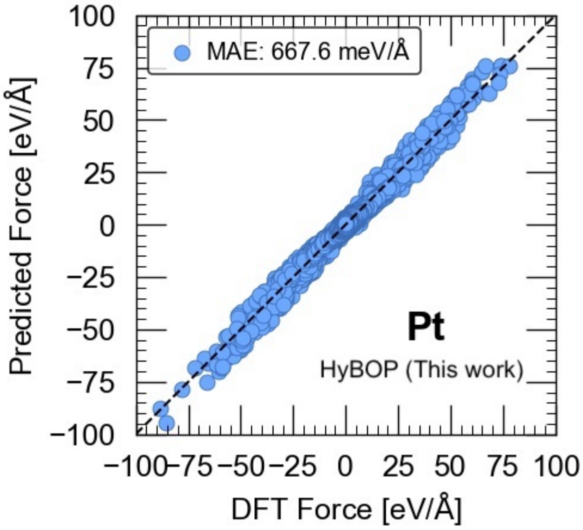

(b)

6.27 Rb

6.27.1 Energy Correlation Plots

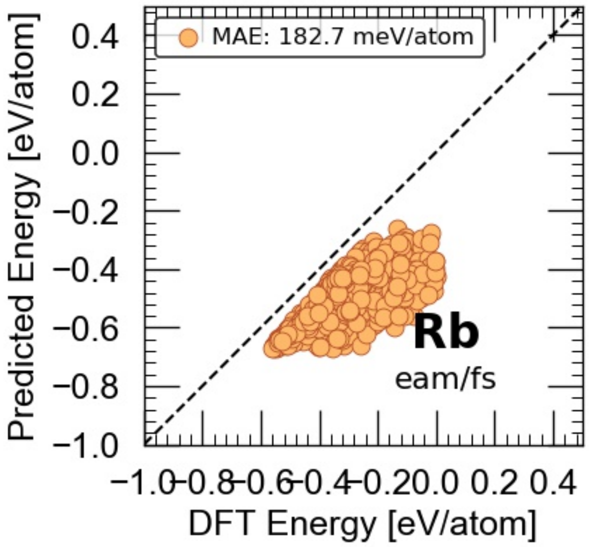

(a)

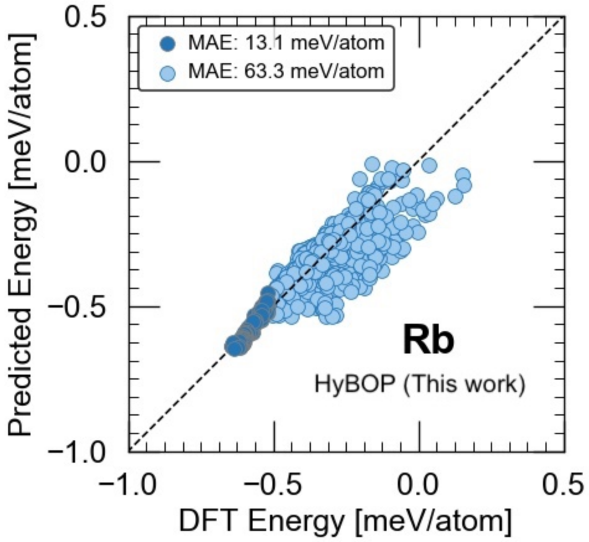

(b)

6.27.2 Force Correlation Plots

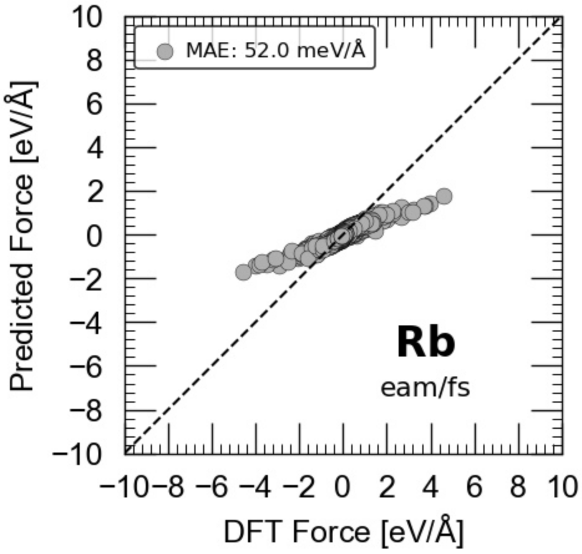

(a)

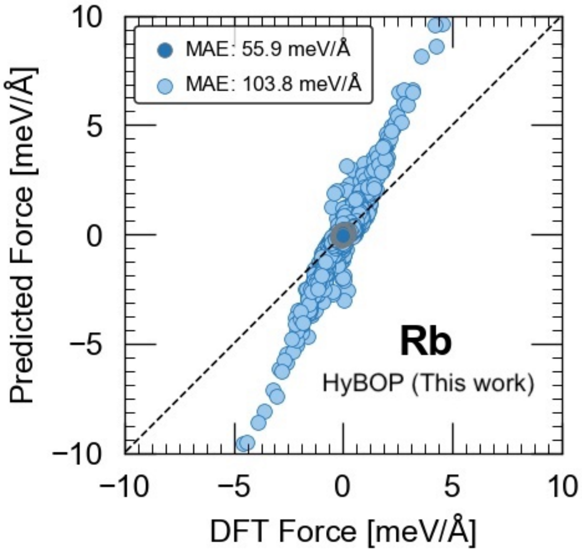

(b)

6.28 Ru

6.28.1 Energy Correlation Plots

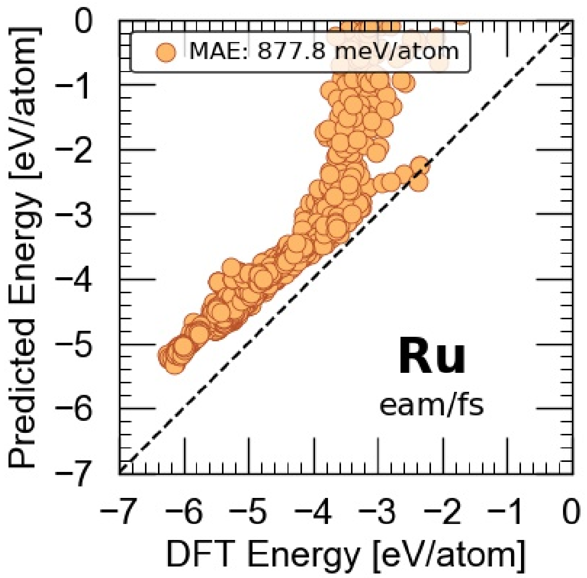

(a)

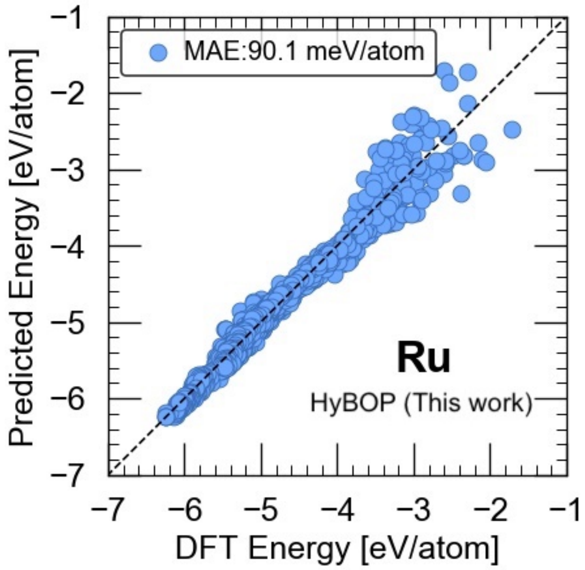

(b)

6.28.2 Force Correlation Plots

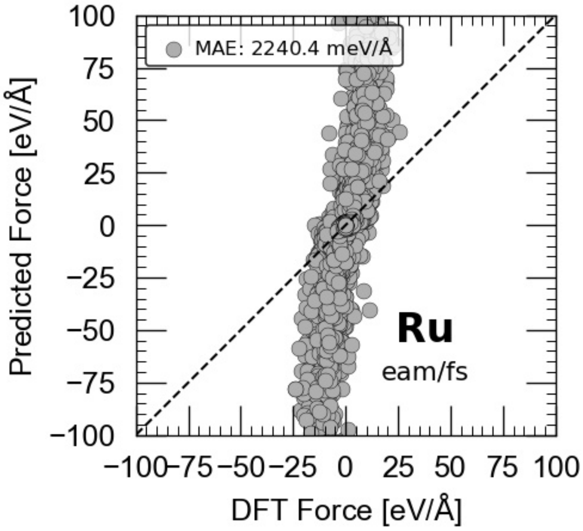

(a)

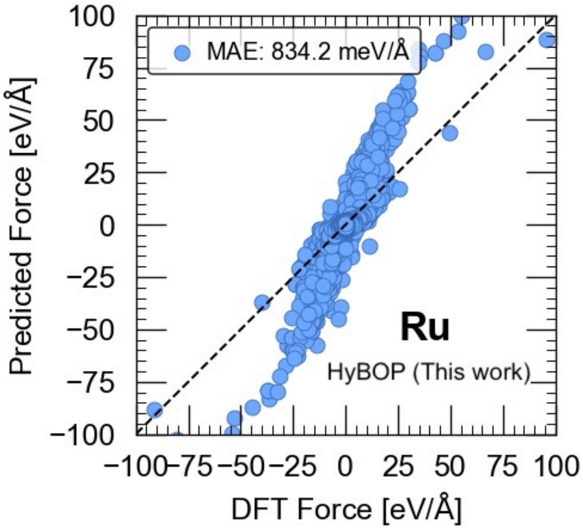

(b)

6.29 S

6.29.1 Energy Correlation Plots

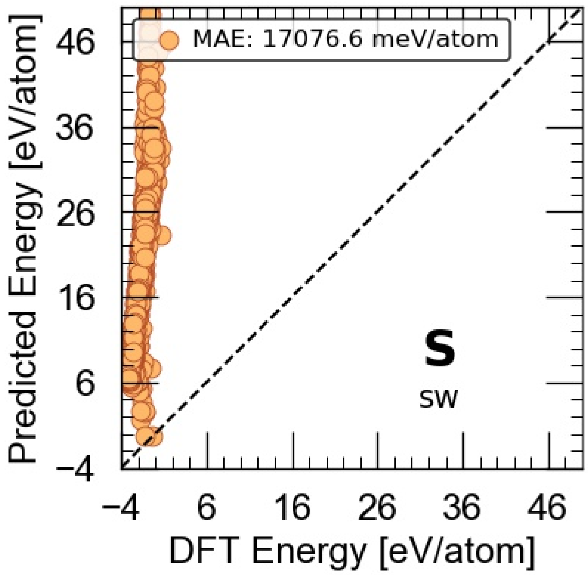

(a)

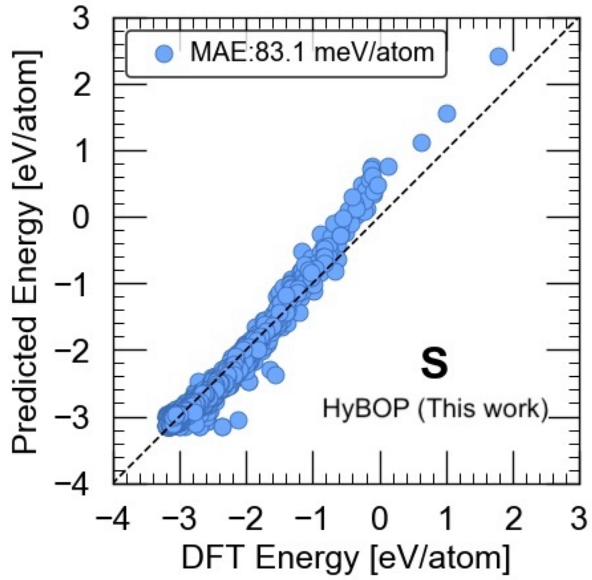

(b)

6.29.2 Force Correlation Plots

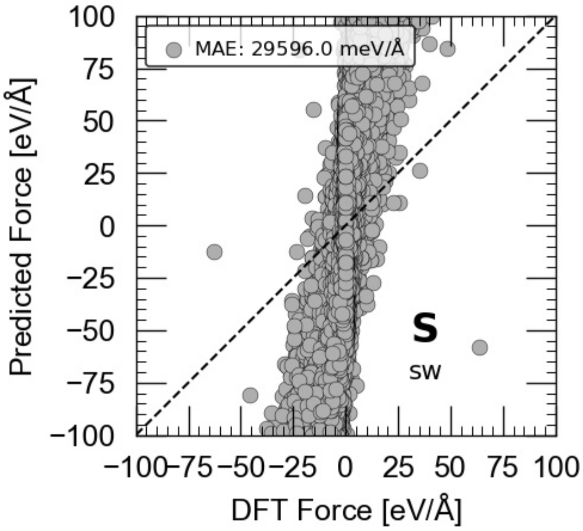

(a)

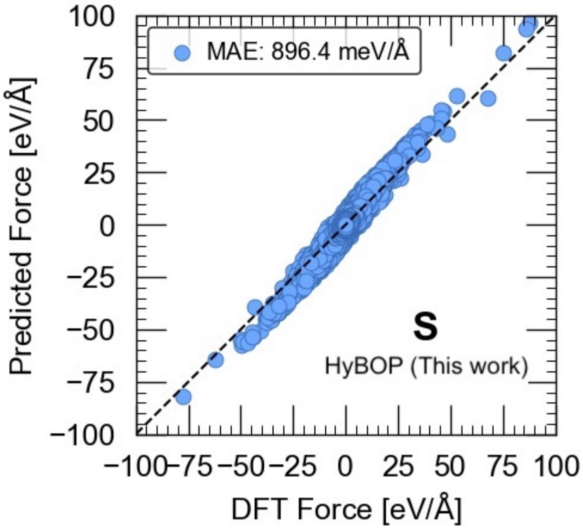

(b)

6.30 Se

6.30.1 Energy Correlation Plots

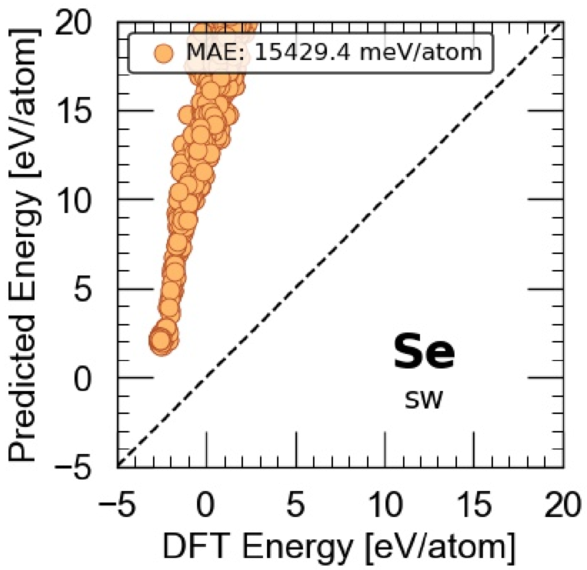

(a)

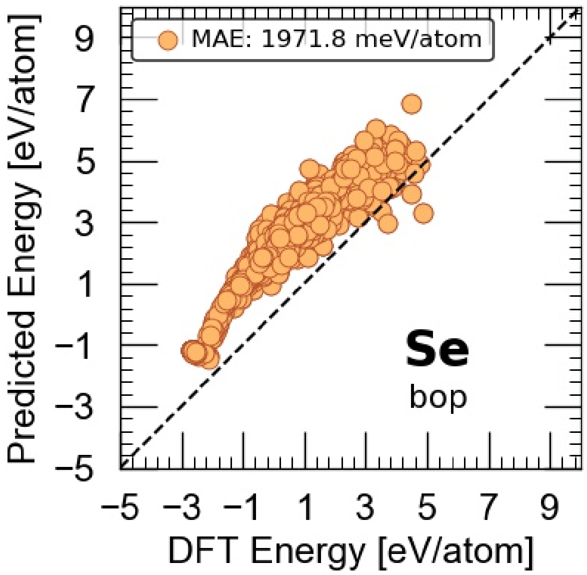

(b)

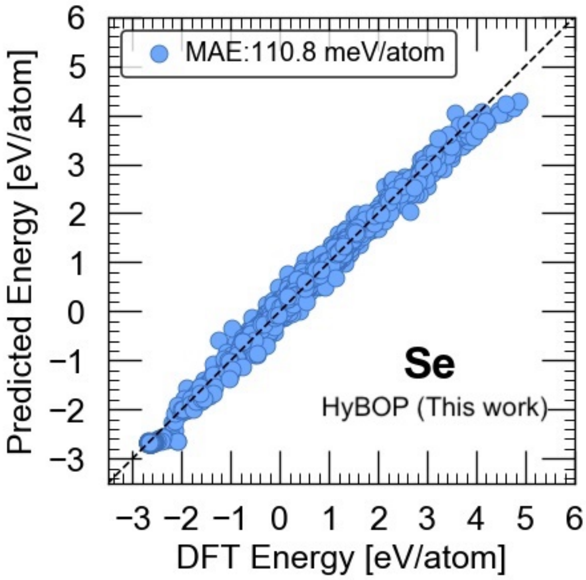

(c)

6.30.2 Force Correlation Plots

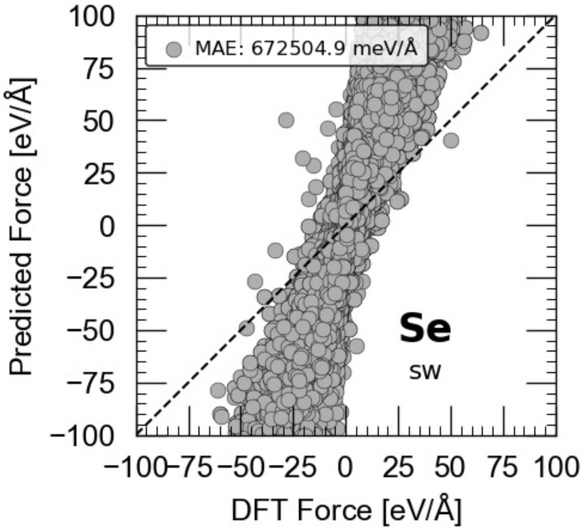

(a)

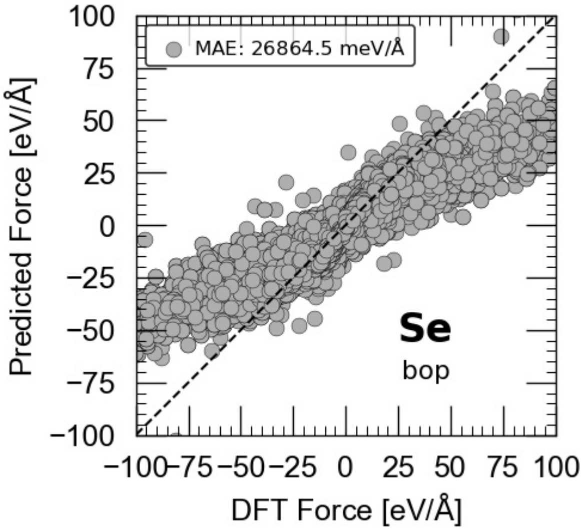

(b)

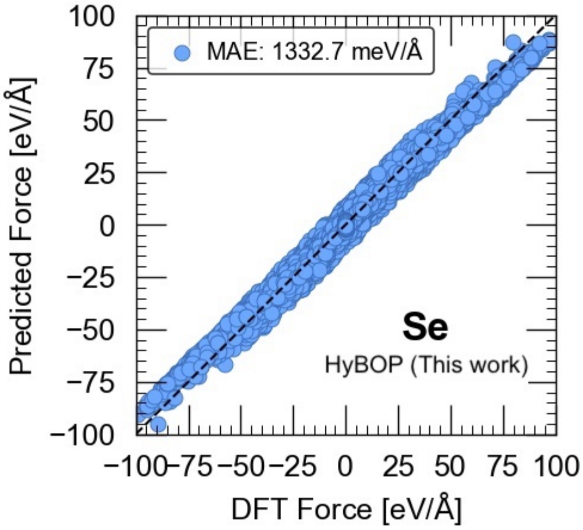

(c)

6.31 Si

6.31.1 Energy Correlation Plots

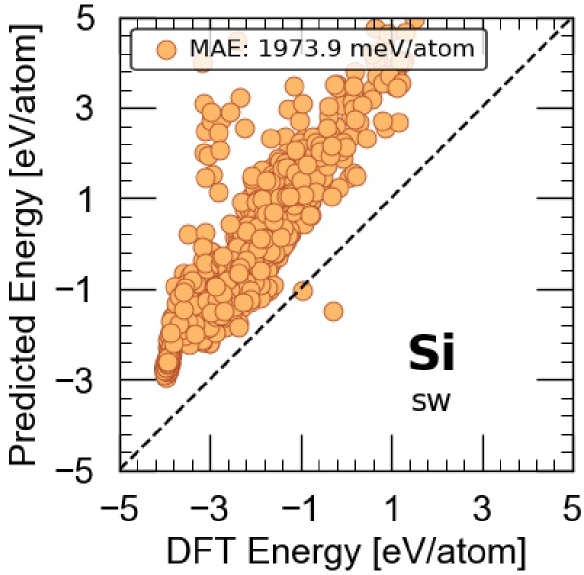

(a)

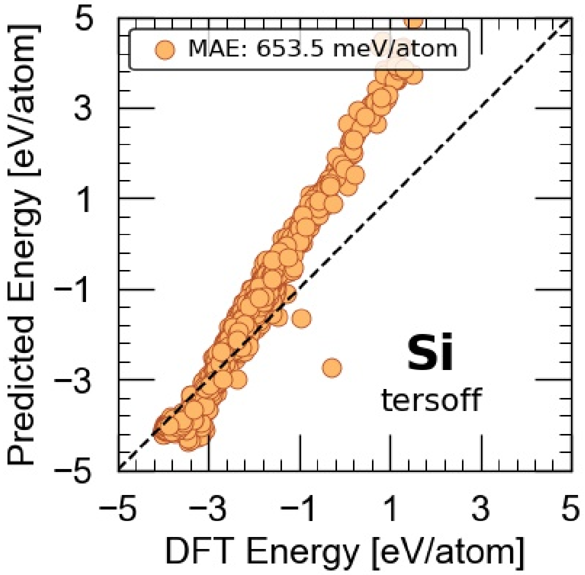

(b)

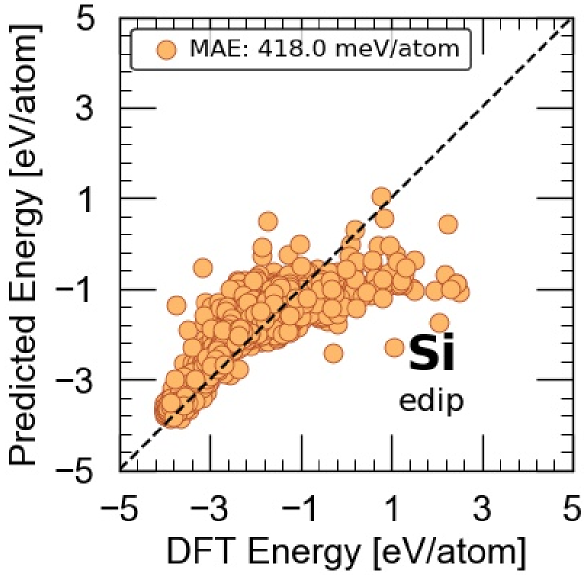

(c)

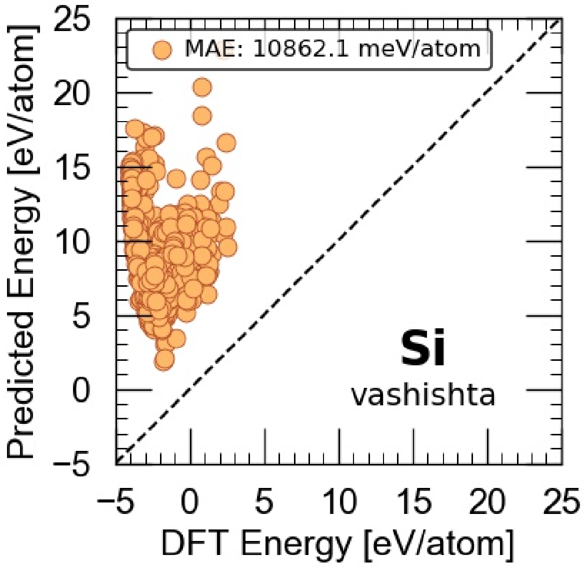

(d)

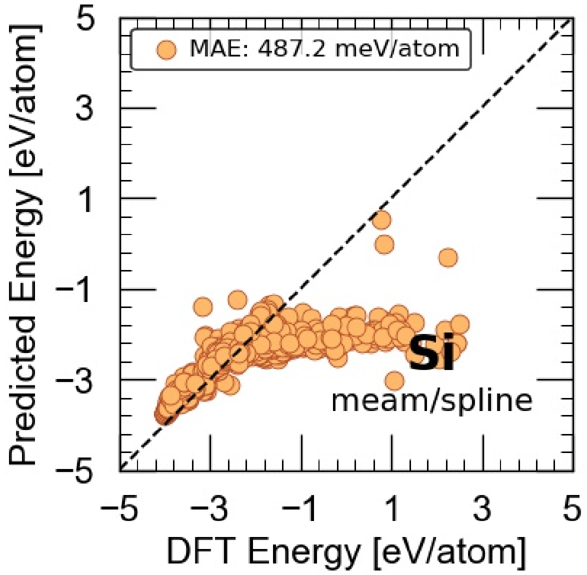
MAE: 487.2 meV/atom
Si
meam/spline
Predicted Energy [eV/atom]
DFT Energy [eV/atom]
5
3
1
−1
−3
−5
−5
−3
−1
1
3
5

(a)

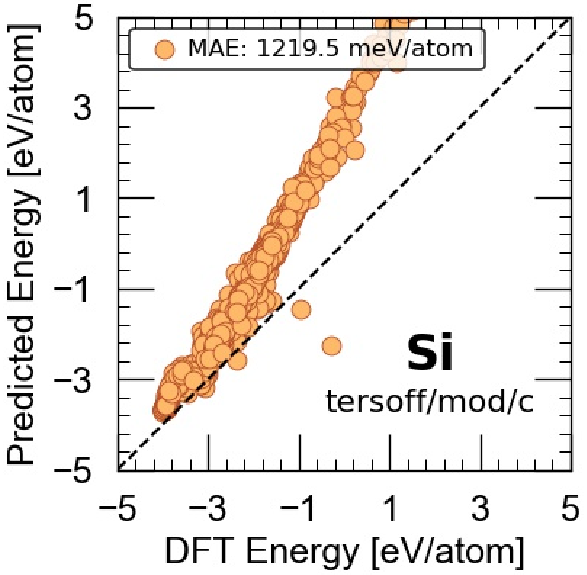
MAE: 1219.5 meV/atom
Si
tersoff/mod/c
Predicted Energy [eV/atom]
DFT Energy [eV/atom]
5
3
1
−1
−3
−5
−5
−3
−1
1
3
5

(b)

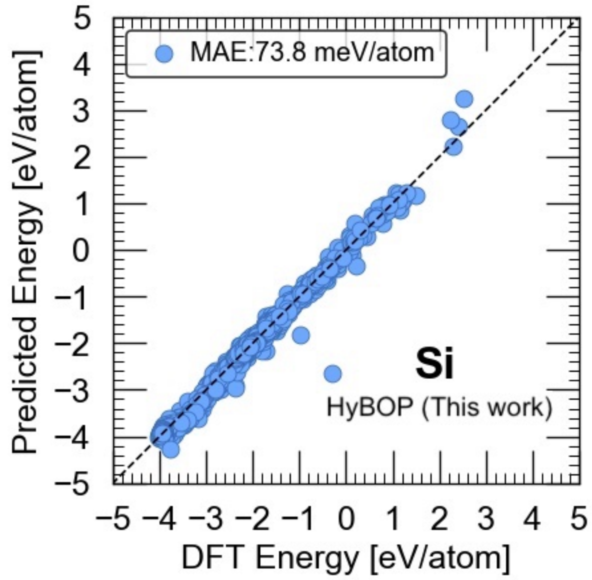
MAE:73.8 meV/atom
Si
HyBOP (This work)
Predicted Energy [eV/atom]
DFT Energy [eV/atom]
5
4
3
2
1
0
−1
−2
−3
−4
−5
−5
−4
−3
−2
−1
0
1
2
3
4
5

(c)

6.31.2 Force Correlation Plots

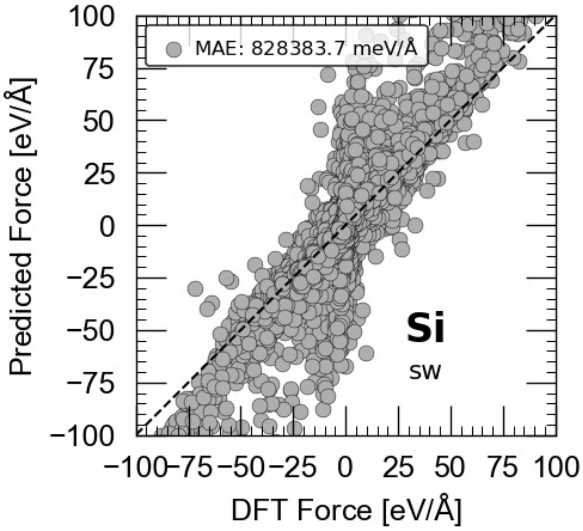

(a)

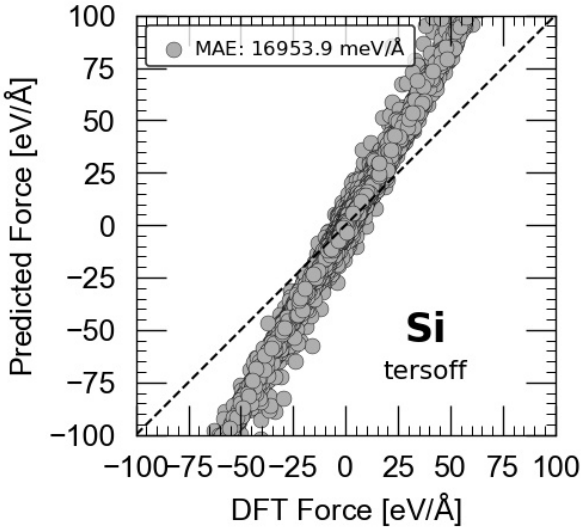

(b)

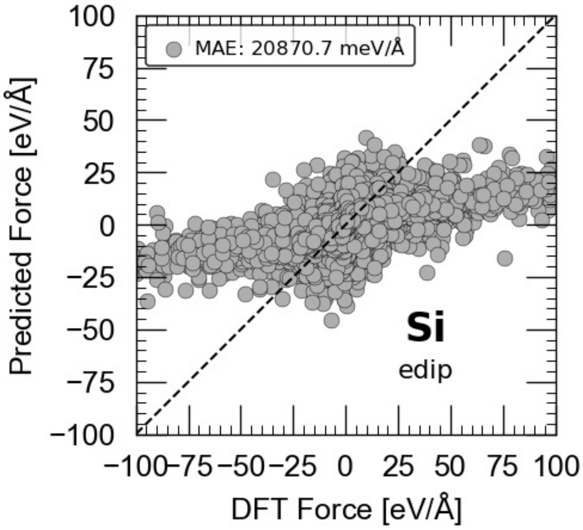

(c)

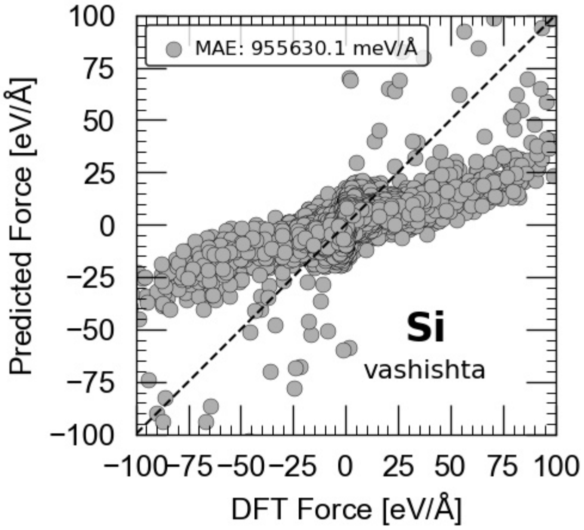

(d)

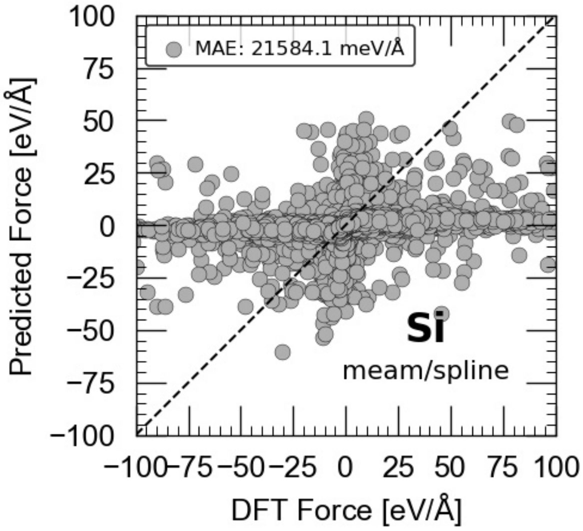
MAE: 21584.1 meV/Å
Si
meam/spline
Predicted Force [eV/Å]
DFT Force [eV/Å]
100
75
50
25
0
−25
−50
−75
−100

(a)

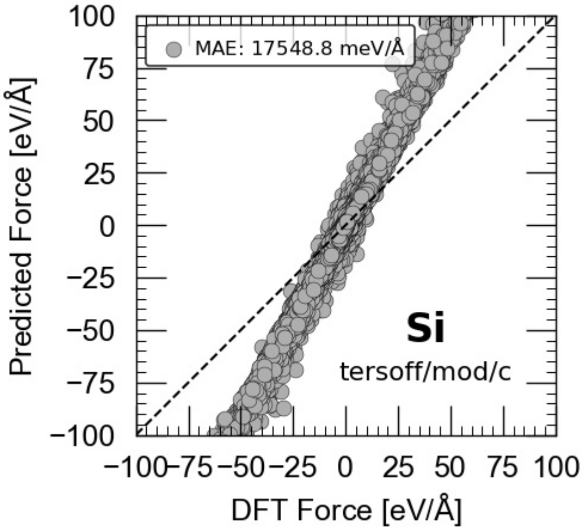
MAE: 17548.8 meV/Å
Si
tersoff/mod/c
Predicted Force [eV/Å]
DFT Force [eV/Å]
100
75
50
25
0
−25
−50
−75
−100

(b)

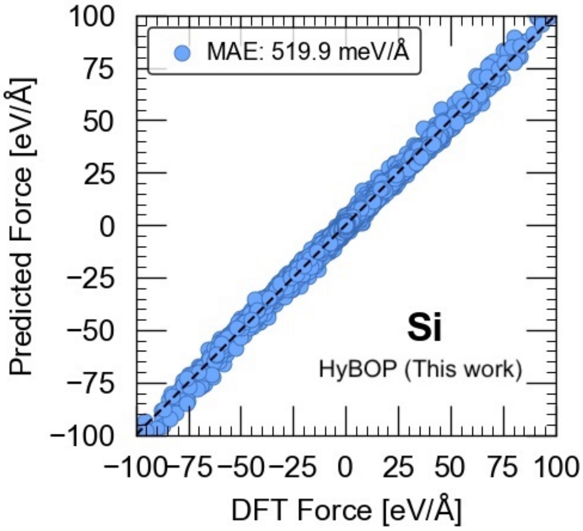
MAE: 519.9 meV/Å
Si
HyBOP (This work)
Predicted Force [eV/Å]
DFT Force [eV/Å]
100
75
50
25
0
−25
−50
−75
−100

(c)

6.32 Ta

6.32.1 Energy Correlation Plots

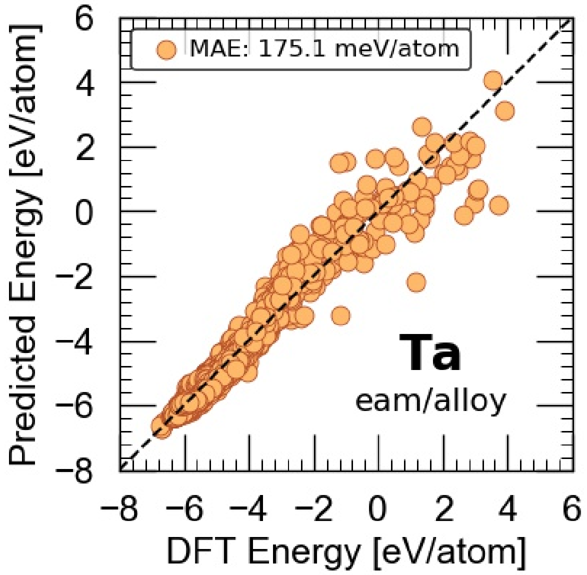

(a)

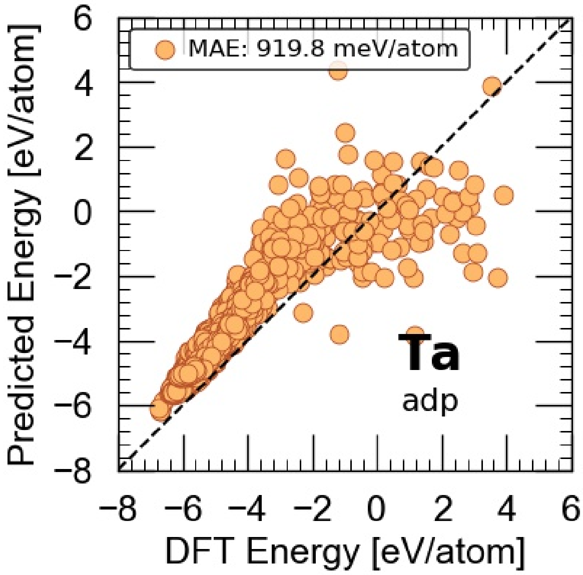

(b)

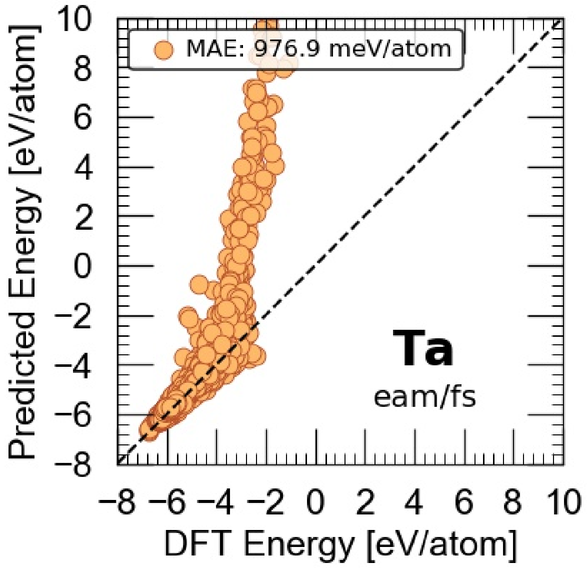

(c)

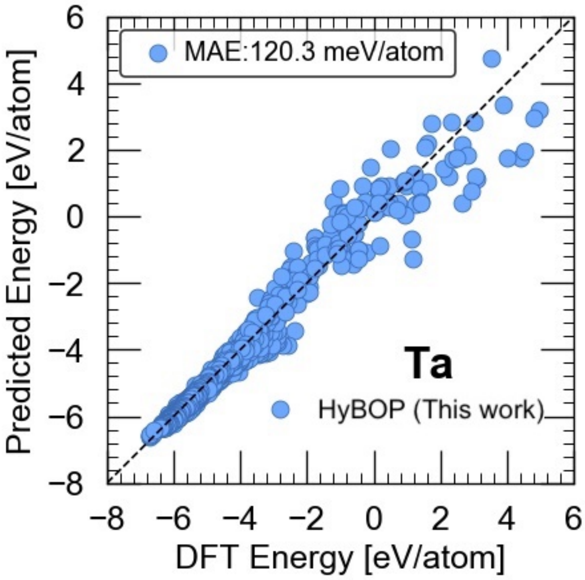

(d)

6.32.2 Force Correlation Plots

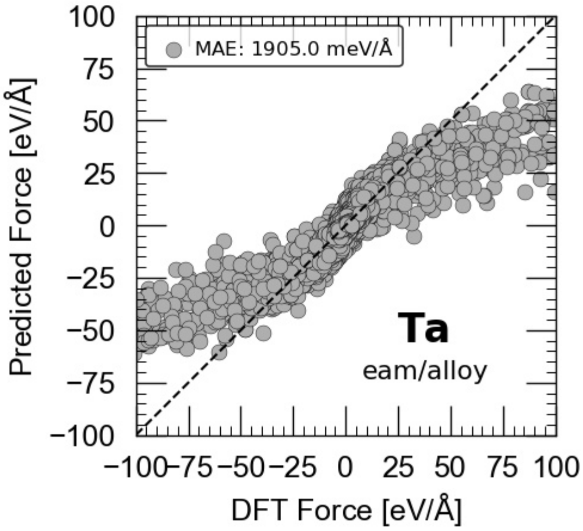

(a)

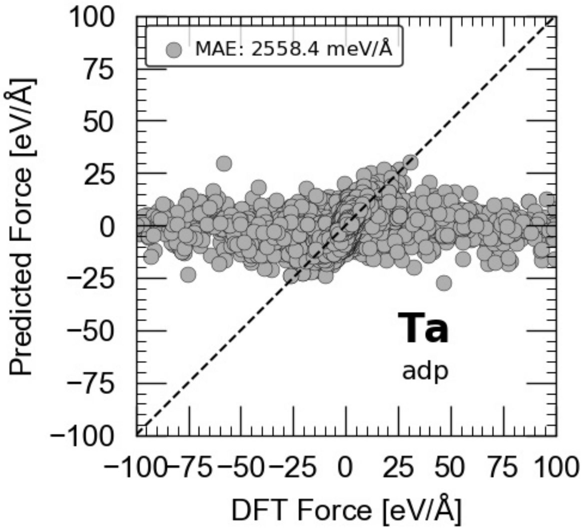

(b)

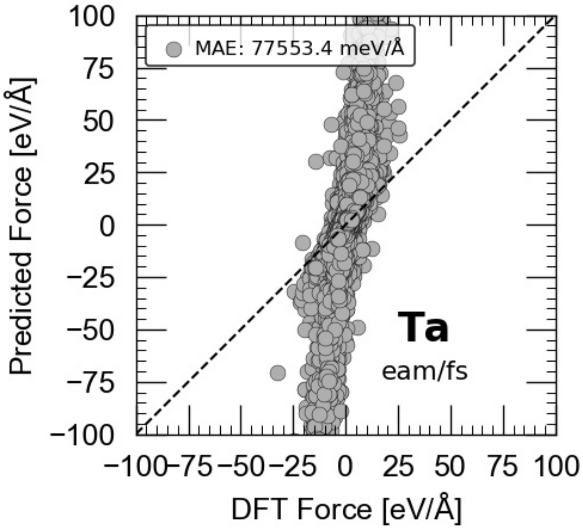

(c)

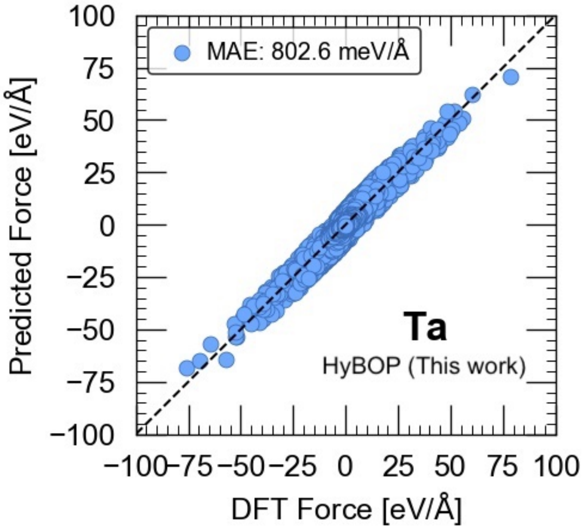

(d)

6.33 Te

6.33.1 Energy Correlation Plots

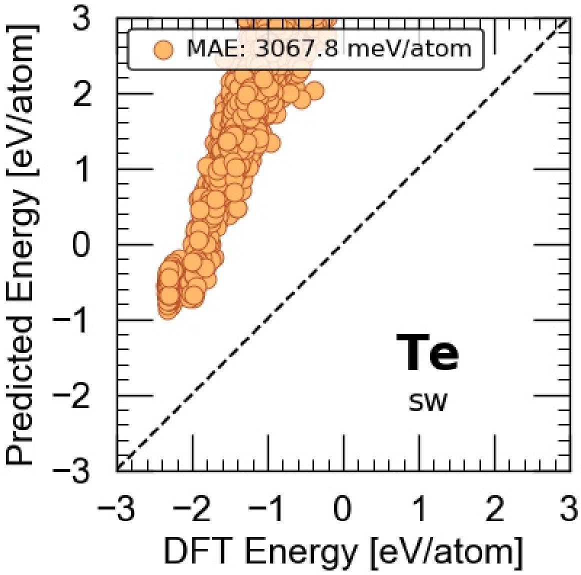

(a)

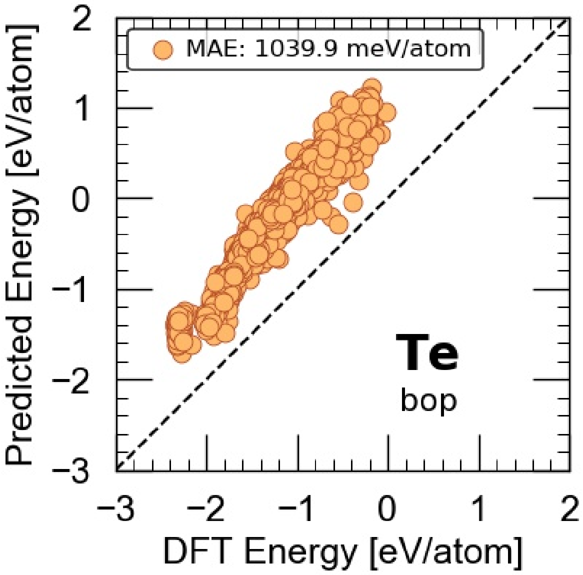

(b)

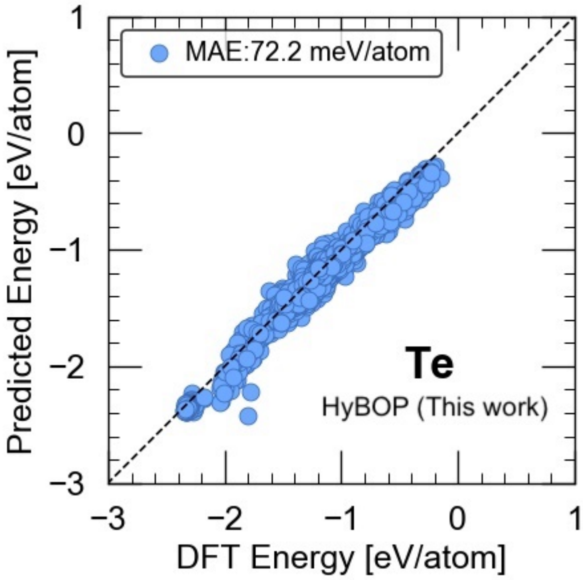

(c)

6.33.2 Force Correlation Plots

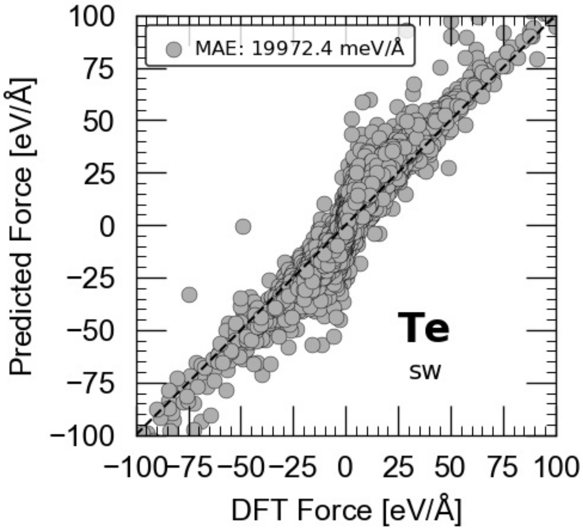

(a)

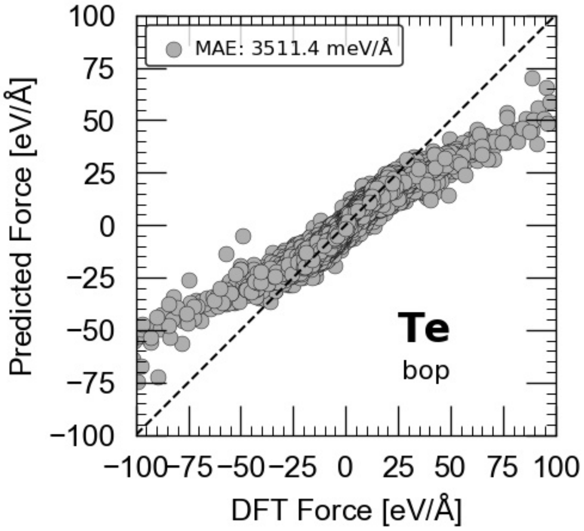

(b)

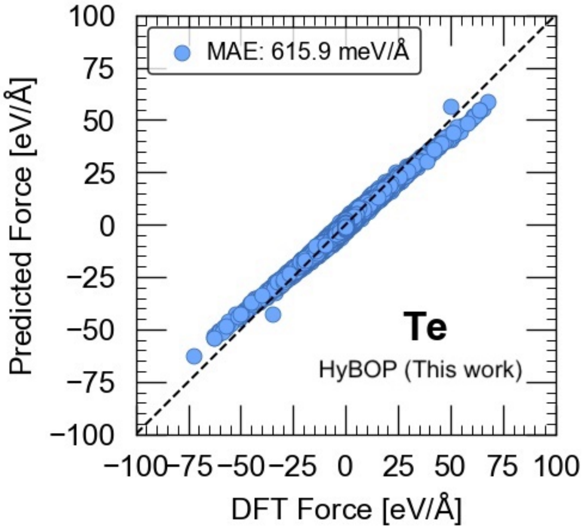

(c)

6.34 Ti

6.34.1 Energy Correlation Plots

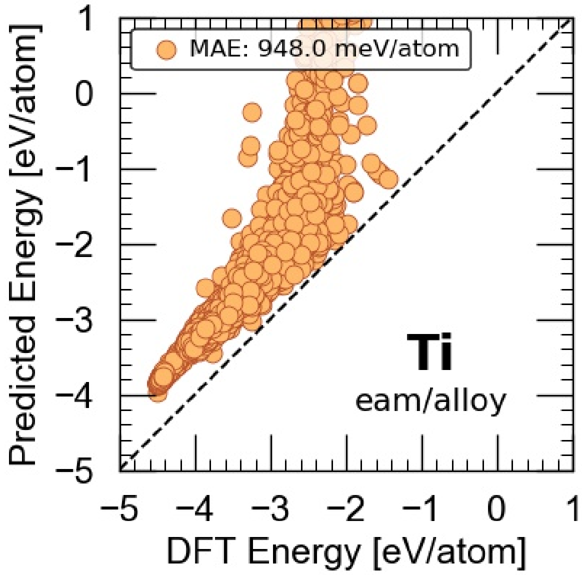

(a)

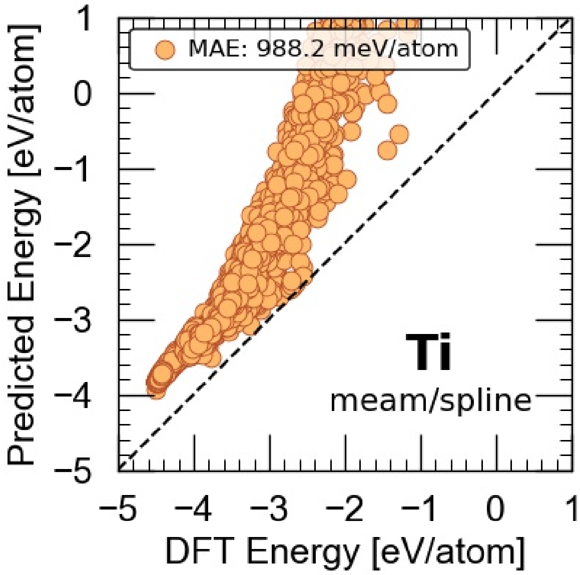

(b)

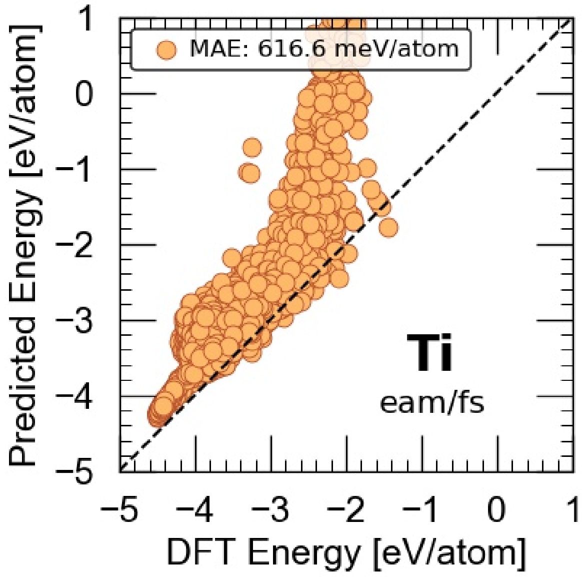

(c)

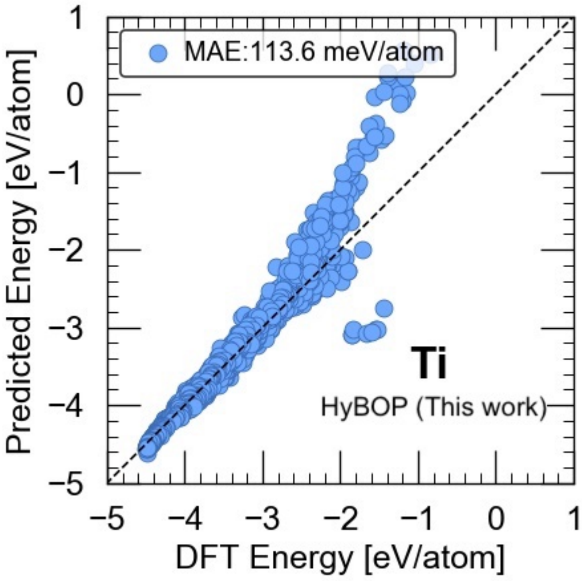

(d)

6.34.2 Force Correlation Plots

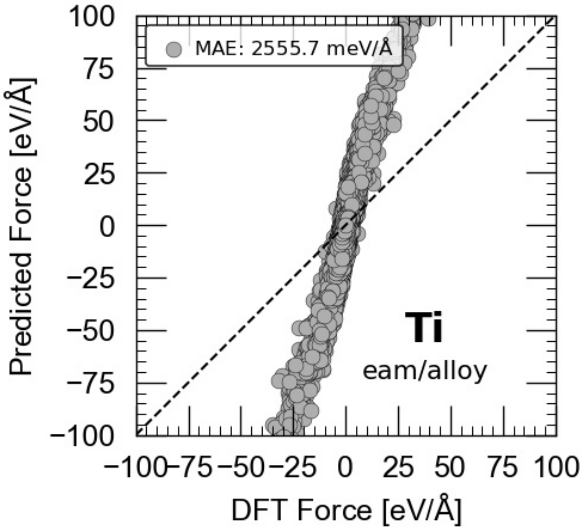

(a)

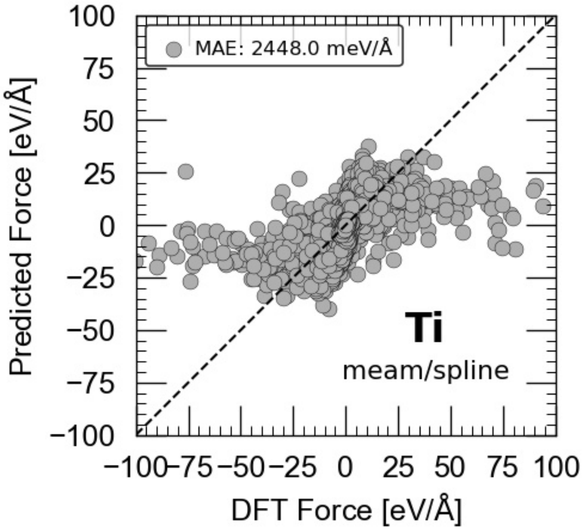

(b)

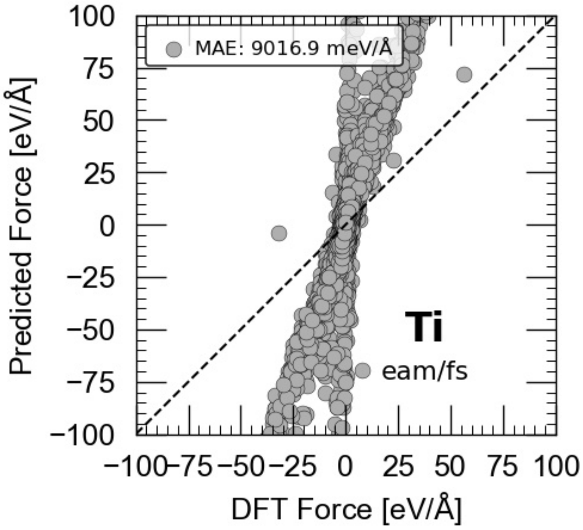

(c)

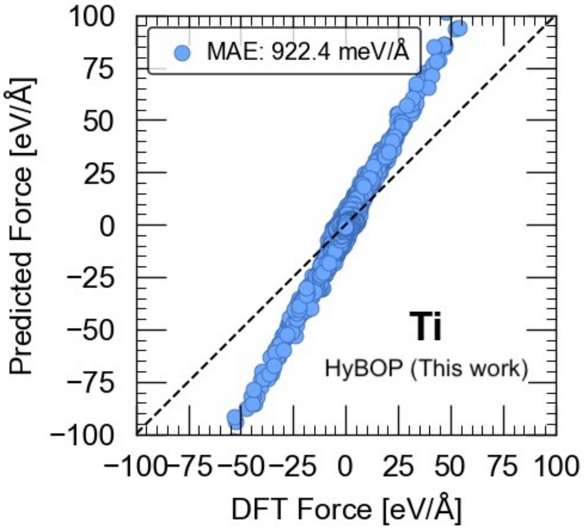

(d)

6.35 V

6.35.1 Energy Correlation Plots

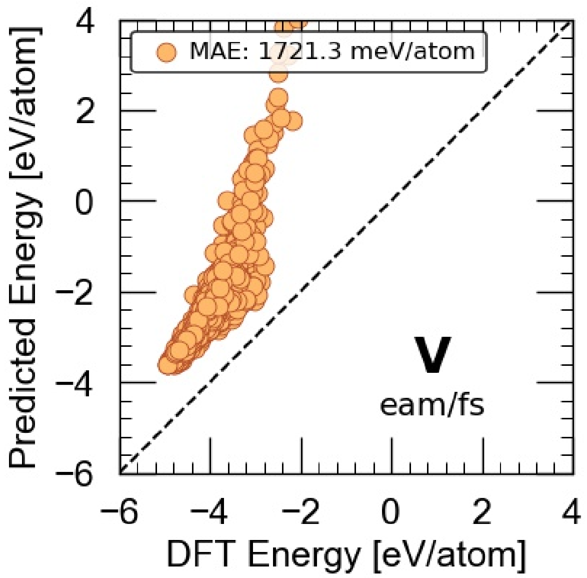

(a)

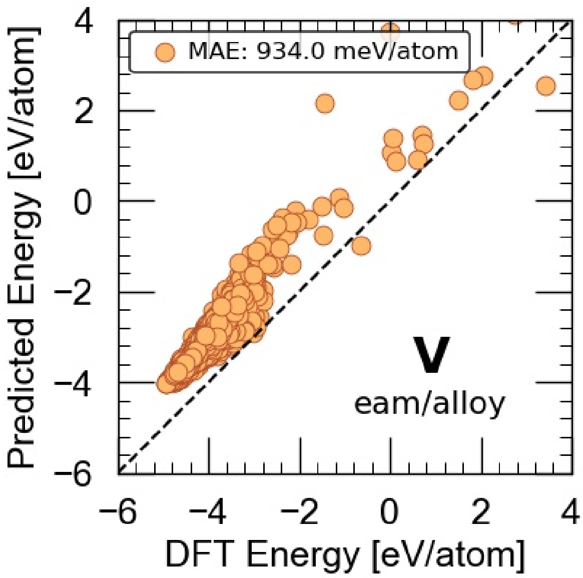

(b)

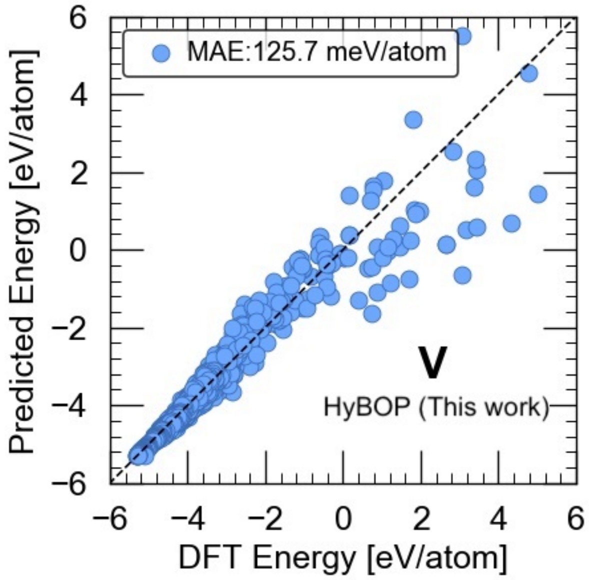

(c)

6.35.2 Force Correlation Plots

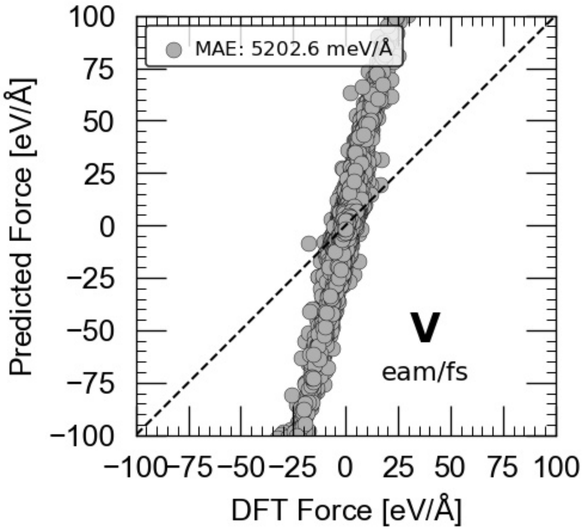

(a)

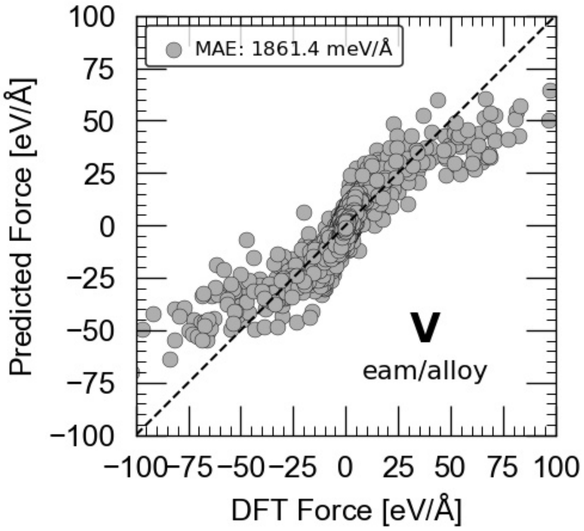

(b)

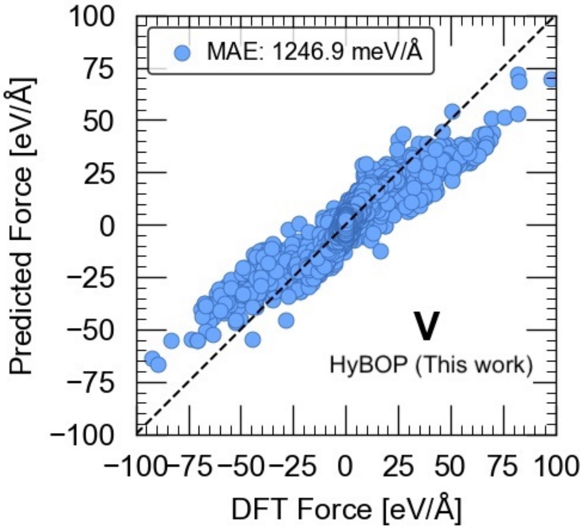

(c)

6.36 W

6.36.1 Energy Correlation Plots

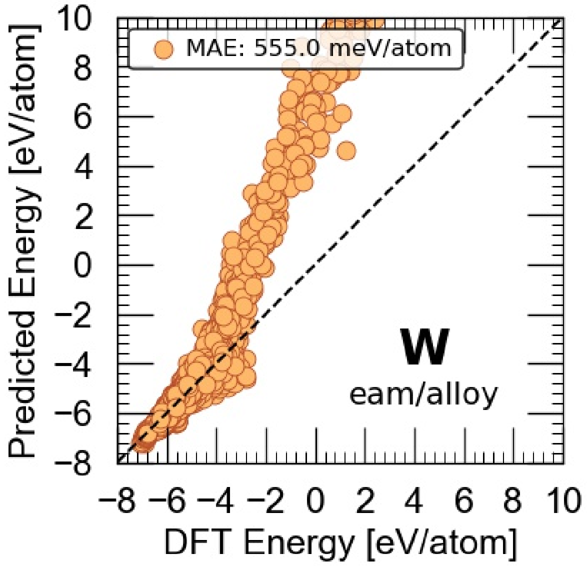

(a)

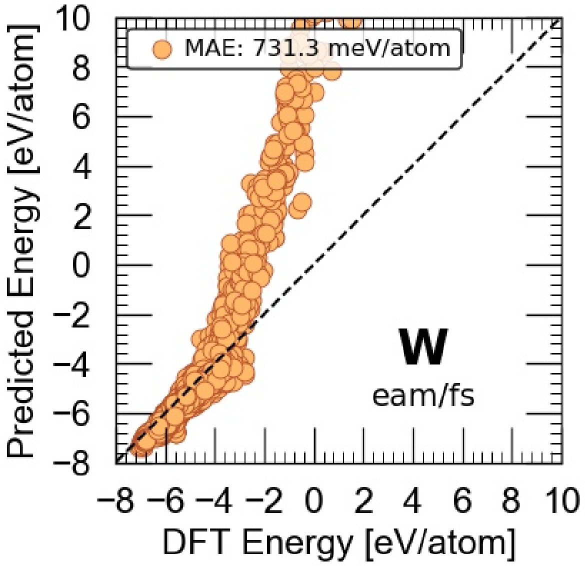

(b)

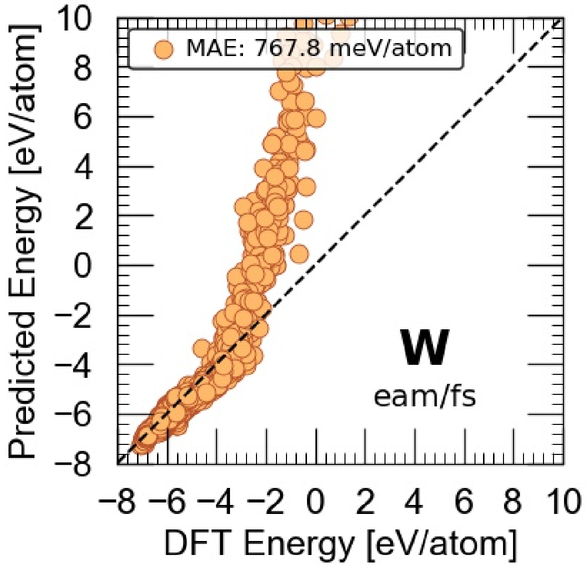

(c)

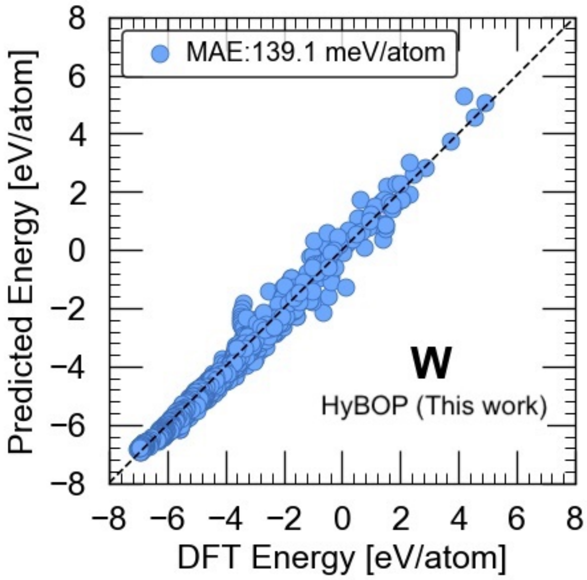

(d)

6.36.2 Force Correlation Plots

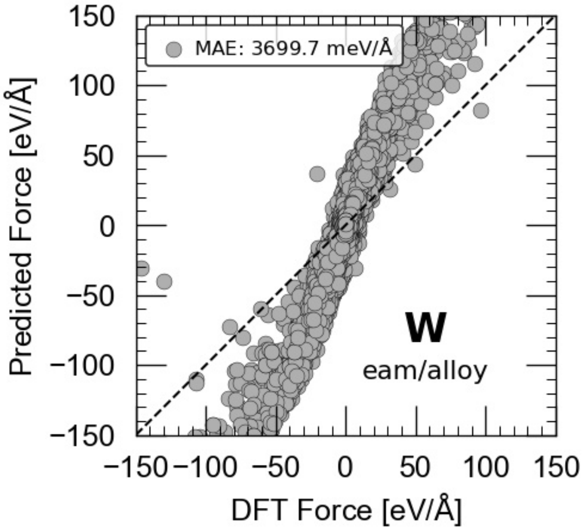

(a)

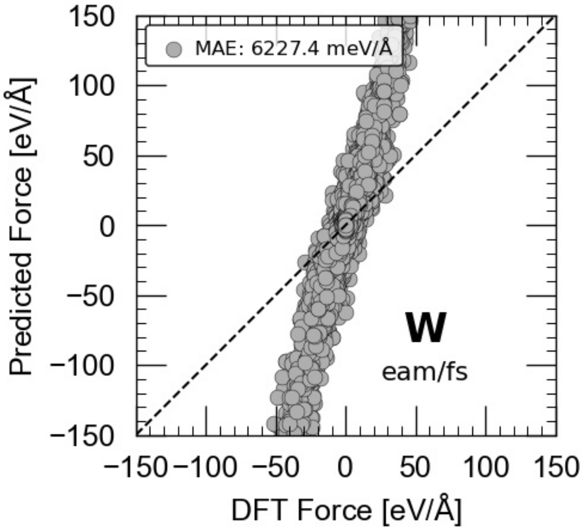

(b)

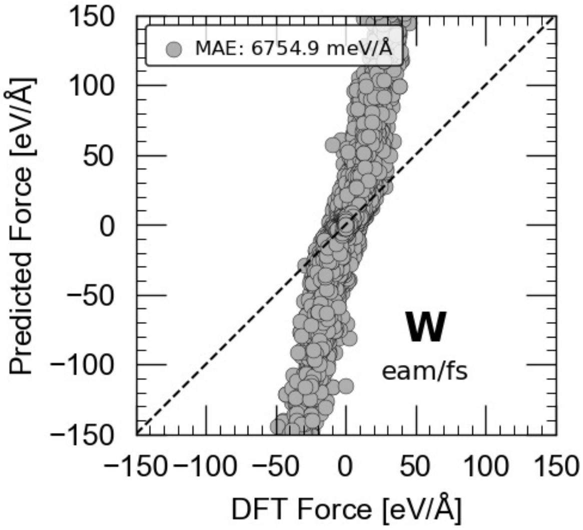

(c)

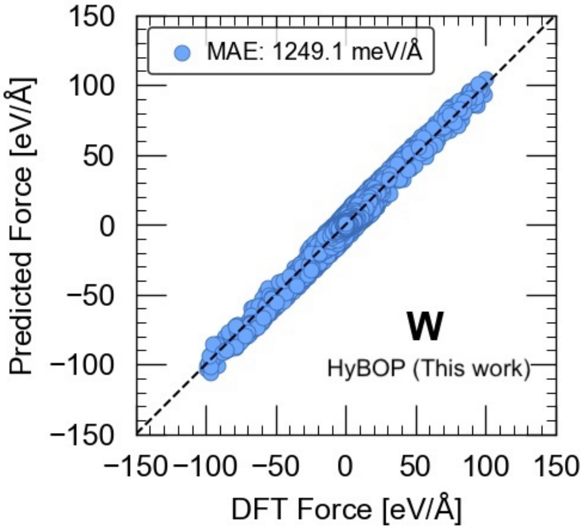

(d)

6.37 Zn

6.37.1 Energy Correlation Plots

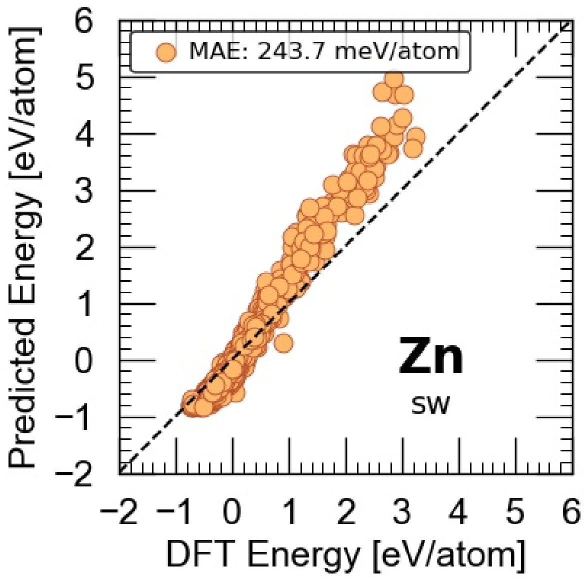

(a)

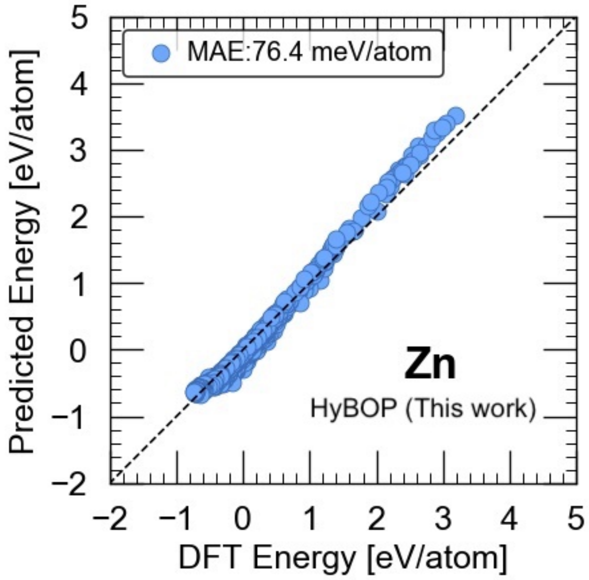

(b)

6.37.2 Force Correlation Plots

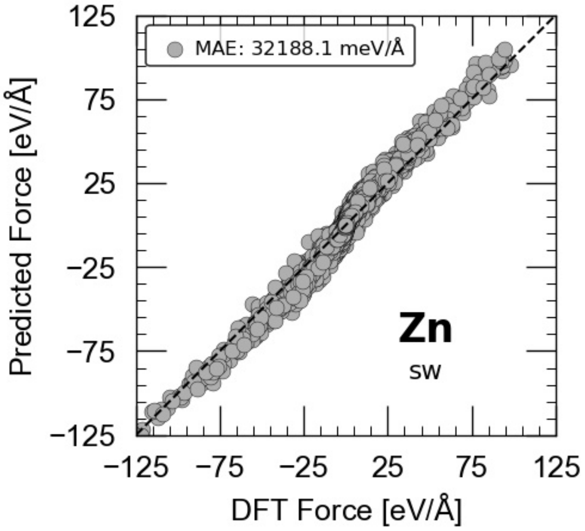

(a)

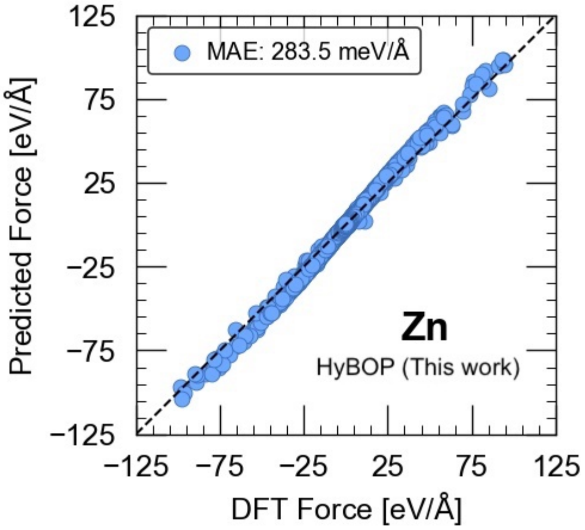

(b)

6.38 Zr

6.38.1 Energy Correlation Plots

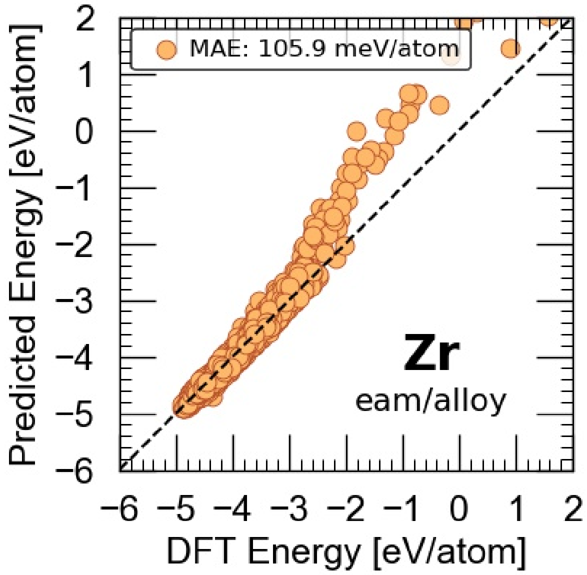

(a)

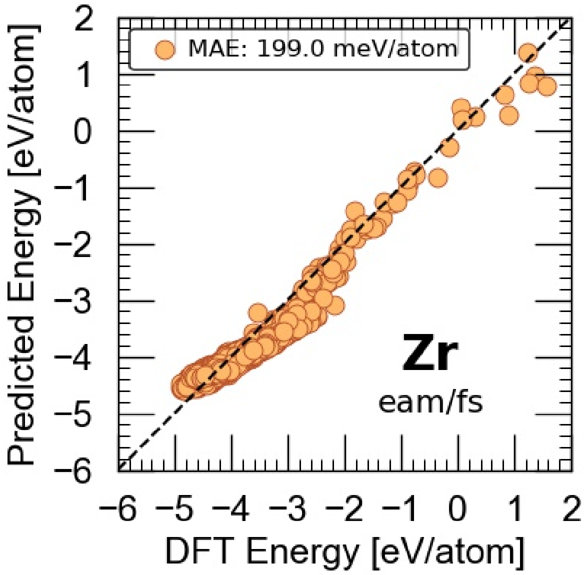

(b)

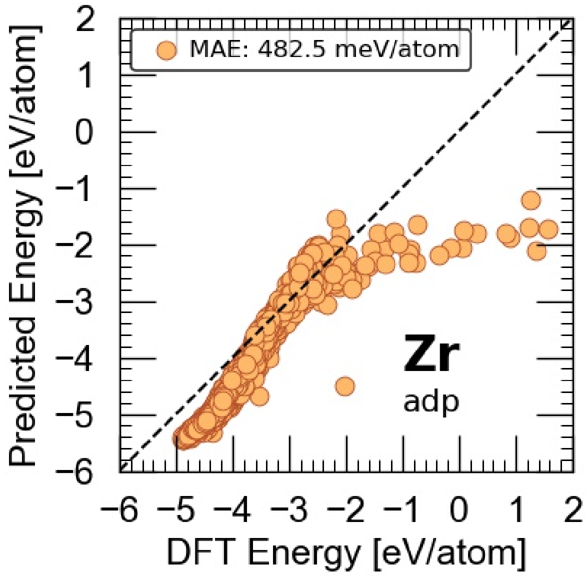

(c)

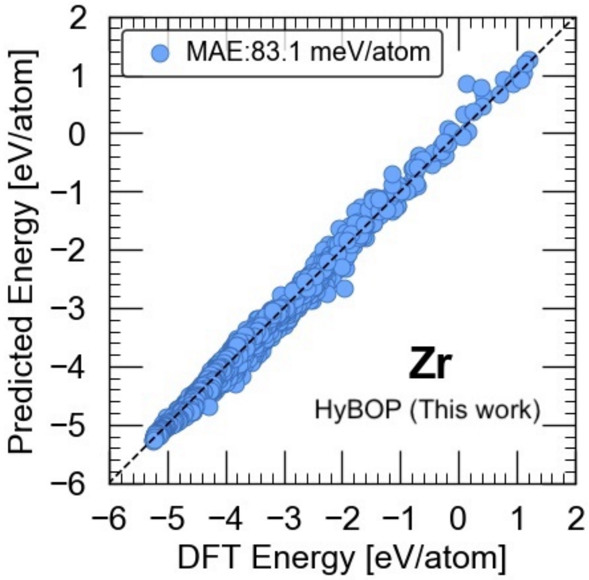

(d)

6.38.2 Force Correlation Plots

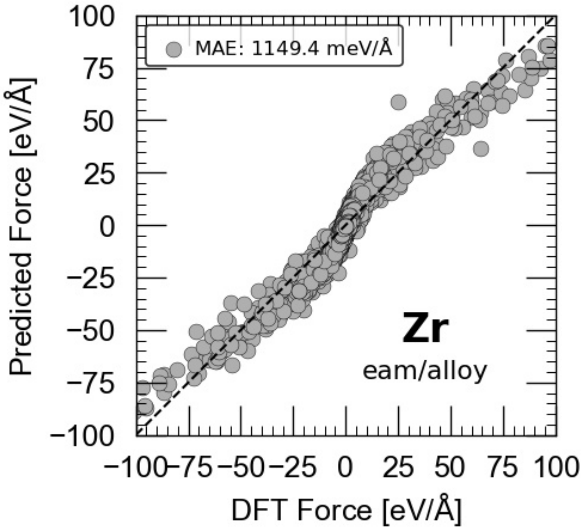

(a)

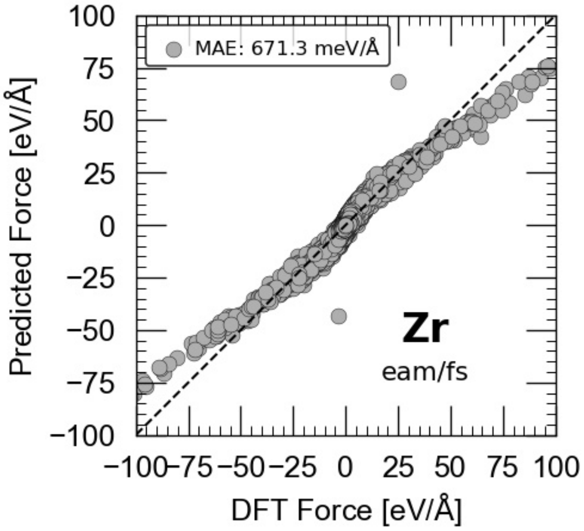

(b)

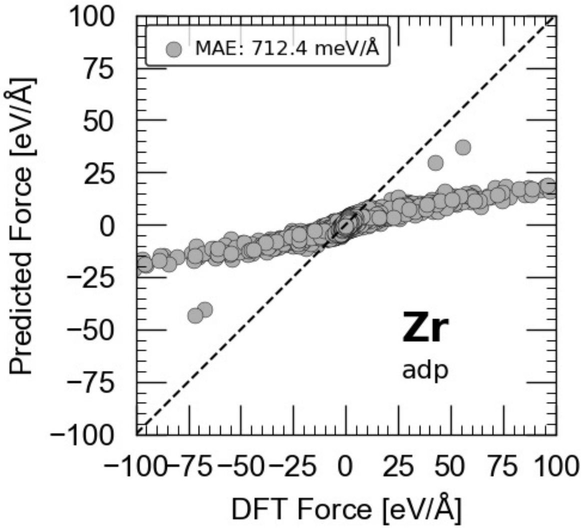

(c)

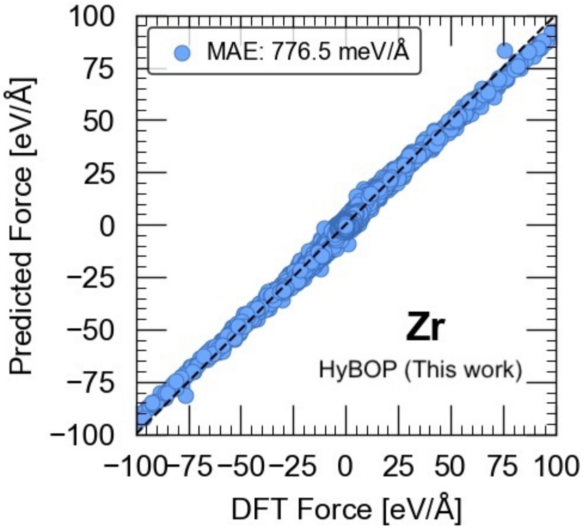

(d)

7 Supplementary Note 3

7.1 Functional Form of Hybrid Bond-Order Potential

The inter-atomic interaction in our hybrid bond order potentials (HyBOP) is described by the short range Tersoff–Brenner functional form (V_{SR}) [58] and a scaled Lennard-Jones (LJ) function for long range interaction (V_{LR}). Hence, the total potential energy in HYBOP is expressed as,

$$V = V_{SR} + V_{LR} \tag{9}$$

The Tersoff-Brenner total potential energy (V_{SR}), which describes the short range interaction consists of pairwise contributions from repulsive $f_R(r_{ij})$ and attractive $f_A(r_{ij})$ interactions between each distinct atomic pair $i-j$ at a distance r_{ij} from each other. The expression for V is written as [114]:

$$V_{SR} = \frac{1}{2}\sum_{i}\sum_{i\neq j} f_C(r_{ij})[f_R(r_{ij}) + b_{ij} f_A(r_{ij})] \tag{10}$$

where $f_C(r_{ij})$ is the cut-off function that restricts the range of interaction to nearest neighbors, and is expressed by,

$$f_C(r) = \begin{cases} 1, & r < R - D \\ \frac{1}{2} - \frac{1}{2}\sin\frac{\pi(r-R)}{2D} & R - D < r < R + D \\ 0, & r > R + D \end{cases} \tag{11}$$

where R and D specify the cut-off distance of the potential. Both the repulsive $f_R(r)$ and attractive contributions to the bond energy of $i-j$ pair decay exponentially with the separation distance r_{ij} and expressed as:

$$f_R(r) = A \cdot e^{-\lambda_1 \cdot r} \tag{12}$$

$$f_A(r) = -B \cdot e^{-\lambda_2 \cdot r} \tag{13}$$

where A, B, λ_1, and λ_2 are free parameters have units Å^{-1}, Å^{-1}, eV, and eV respectively. The term b_{ij} in eq describes the bond order around a pair of atoms $i-j$, which is described by three-body interactions expressed as,

$$b_{ij} = (1 + \beta^n \zeta_{ij}^n)^{\frac{-1}{2n}} \tag{14}$$

where, ζ_{ij} is described by,

$$\zeta_{ij} = \sum_{k\neq i,j} f_c(r_{ik}) g_{ik}(\theta_{ijk}) e^{[\lambda_3(r_{ij}-r_{ik}]} \tag{15}$$

where the $g(\theta)$ is expressed as,

$$g(\theta) = \gamma(1 + \frac{c^2}{d^2} - \frac{c^2}{d^2 + (cos\theta + h)^2}) \tag{16}$$

The scaled Lennard-Jones (LJ) function which that describes long range interaction (V_{LR}) is given by,

$$V_{LR} = \sum_{i}\sum_{j>i} 4\epsilon_{ij} f_s(M_i)\Big[\Big(\frac{\sigma_{ij}}{r_{ij}}\Big)^{12} - \Big(\frac{\sigma_{ij}}{r_{ij}}\Big)^{6}\Big] \tag{17}$$

where, ϵ_{ij} and σ_{ij} are LJ parameters for a pair of atoms i and j that are a distance r_{ij} apart. $f_s(M_i)$ is a scaling function that describes the dependence of LR contribution from a given atomic pair $i-j$ on the number of atoms within a prescribed radial distance R_c^{LR} from the atom i.

$$f_s(M) = \frac{1}{2}\Big[erf\Big(\frac{M}{\kappa_1} - \kappa_2\Big) + 1\Big] \tag{18}$$

where κ_1 and κ_2 are the scaling parameters. The magnitude of $f_s(M)$ is negligible for small clusters, and hence, their energetics is described by the Tersoff-type BOP terms only (i.e., V_{SR}). For bulk polymorphs, $f_s(M)$ reaches its maximum value of 1.0.

The 13 short range parameters ($A, B, R, D, \beta, n, \lambda_1, \lambda_2, \lambda_3, \gamma, c, d,$ and h) and 5 long range parameters ($\epsilon, \sigma, \kappa_1, \kappa_2$ and R_c^{LR}) optimized using c-MCTS for all 54 elements. The 18 optimized parameters for all 54 elements are provided in the Supplementary Data 1 in the main manuscript.

8 Supplementary Note 4

8.1 Workflow Overview

The ML workflow to train potentials for 54 different elements across the Periodic table involves three main aspects: (1) model selection, (2) training (and test) data generation, and (3) optimization of a reward (or objective) function using c-MCTS and the cluster training data. Details on model selection and training (and test) data generations can be found above in section 7.1 and 4.4, respectively. The definition of the reward/objective function are described below.

8.2 Reward Function Evaluation

During a reward evaluation of each element, we employed a batch sampling methodology wherein we randomly select 100 clusters from our training dataset such that it contains at least 10 low energy clusters with each constituting atoms that experience forces less than a cut-off (F_{cut}) value; F_{cut} was set to 0.05 eV/Å for this work. We note that the node selection during MCTS run is given by the complete Eq. 1 in the main manuscript. However, here, we focus on one of its key component, i.e., the reward function (or penalty imposed), given by following three parts:

(a) Clusters far from equilibrium: This forms the error in energy prediction, plus an additional penalty if the difference exceeds 25 meV/atom.

(b) Low energy clusters: In this scheme we additionally perform 10 minimization steps on the low energy clusters for the choice of parameter set using LAMMPS. If the energy difference between the structures before and after the minimization exceed 25 meV/atom, an additional penalty is added. This ensures the stability of nanoclusters. The reward/objective value described in previous section (a) is also included here.

(c) Energetic ordering among isomers: Apart from the criteria described in (a) and (b), we also assigned a penalty term if the energetic ordering among clusters of same sizes is not retained. This helps preserve relative ordering among the isomer clusters, which was previously not captured in other empirical force fields (for e.g. in Au nanoclusters [115, 62, 116, 117]).

The cumulative reward/objective is the sum of all the above three components, which is minimized using c-MCTS. The convergence of objective function with c-MCTS iteration steps is shown in Figure 97. We use the same workflow for developing force fields for all 54 elemental nanoclusters across the Periodic table as described above.

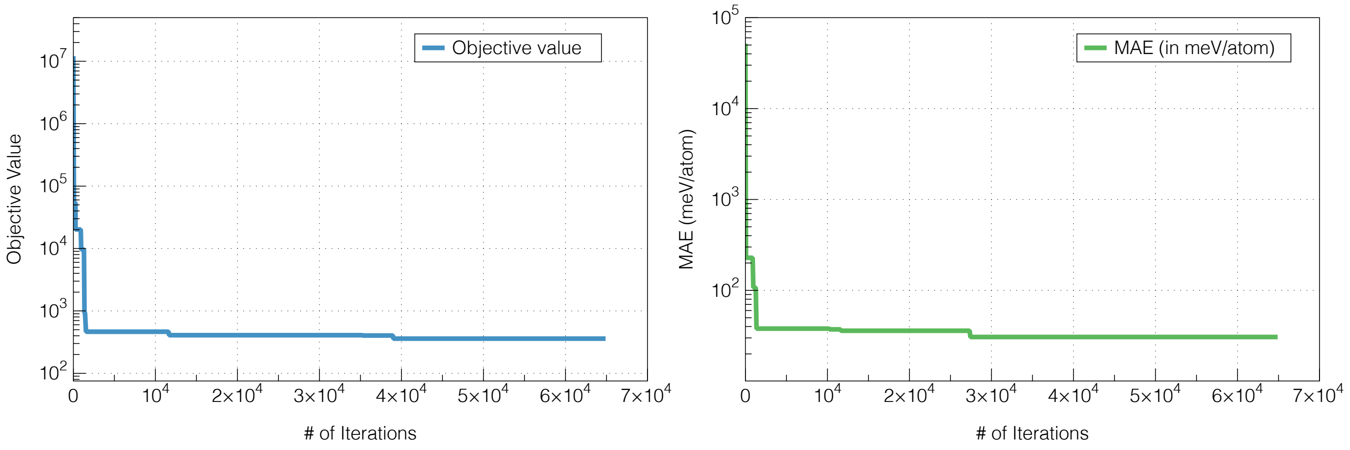

(a) (b)

Supplementary Figure 97: The (a) reward/objective value and (b) the calculated MAE as a function of c-MCTS iteration steps for element Ag.

9 Supplementary Note 5

9.1 Performance of c-MCTS Trained HyBOP Potentials: Energy and Force Predictions

In order to demonstrate the performance of our ML force-field, we include correlation plots of both the energy and the forces for each elements in an alphabetical manner (Figures 98 - 151). The mean absolute errors for energies (in meV/atom) and forces (in meV/Å) are included in the legend of the plot. Each plot has four panels; top row containing energy plots for the (a) training and (b) test data, while the bottom row covering force plots for the (c) training and (d) test data. For alkali elements (i.e. Li, Na, K, Rb, Cs) we particularly highlight the performance of low energy clusters using different colors.

9.2 Ag

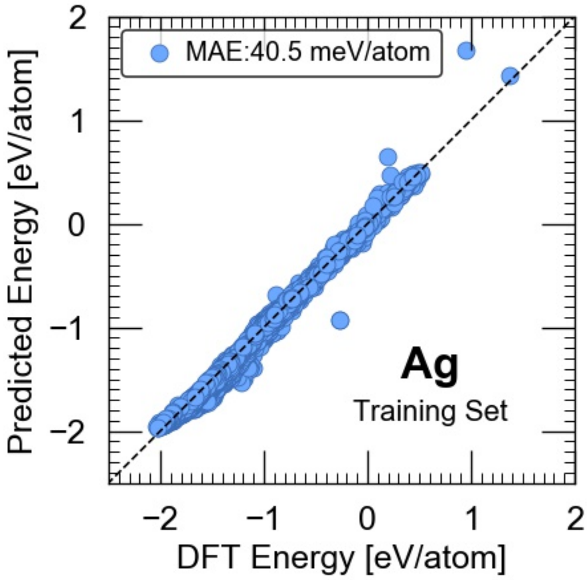

(a)

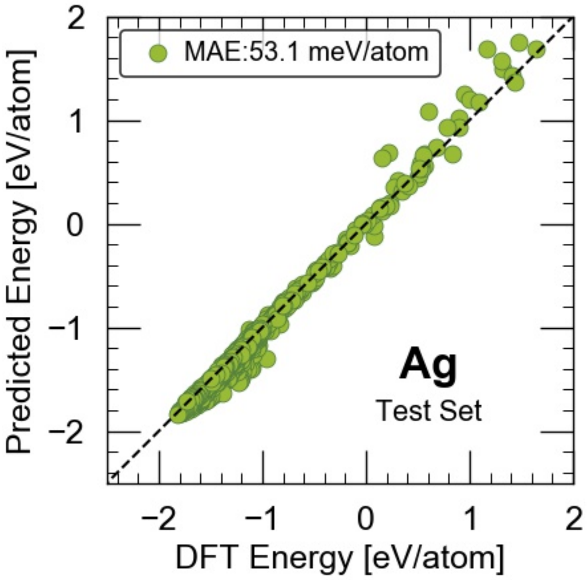

(b)

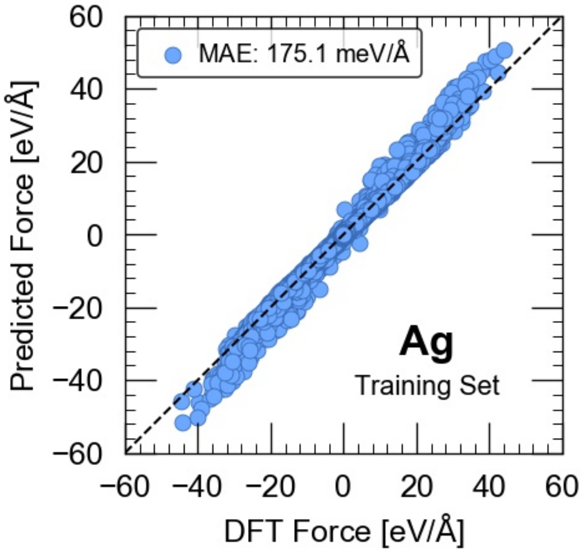

(c)

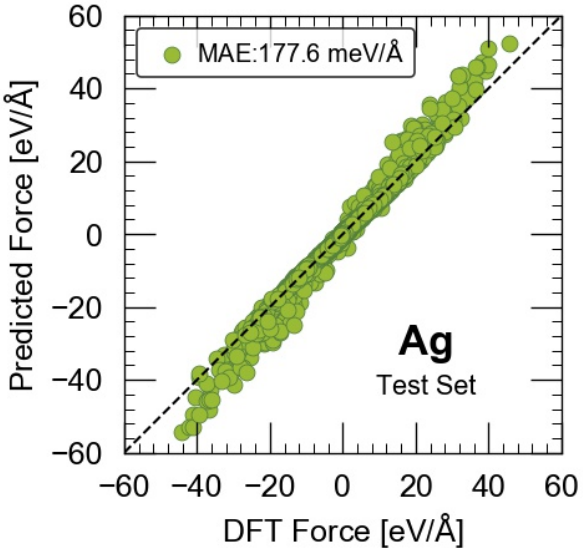

(d)

Supplementary Figure 98: Energy and force correlation plots for Ag nanoclusters

9.3 Al

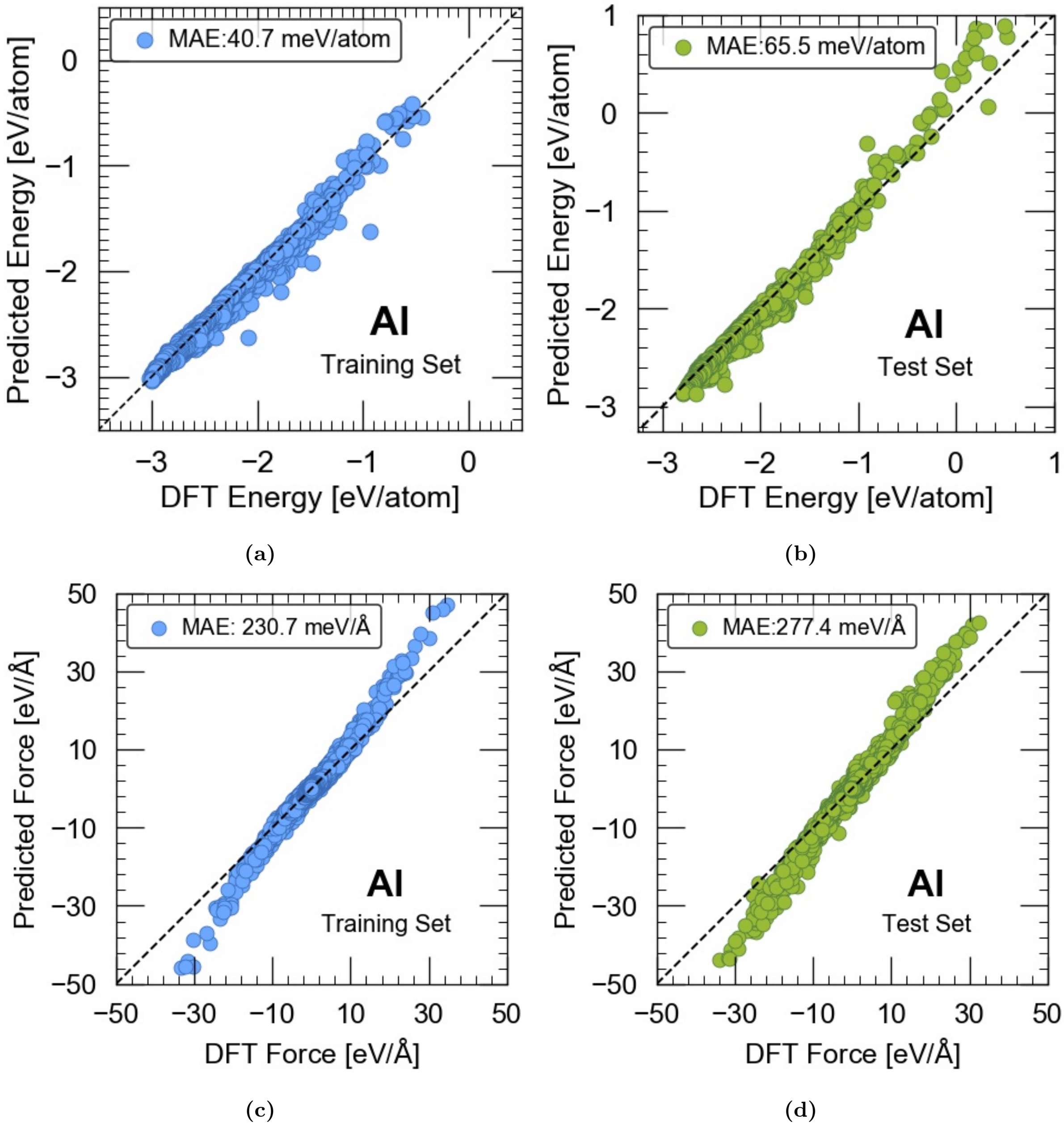

Supplementary Figure 99: Energy and force correlation plots for Al nanoclusters

9.4 As

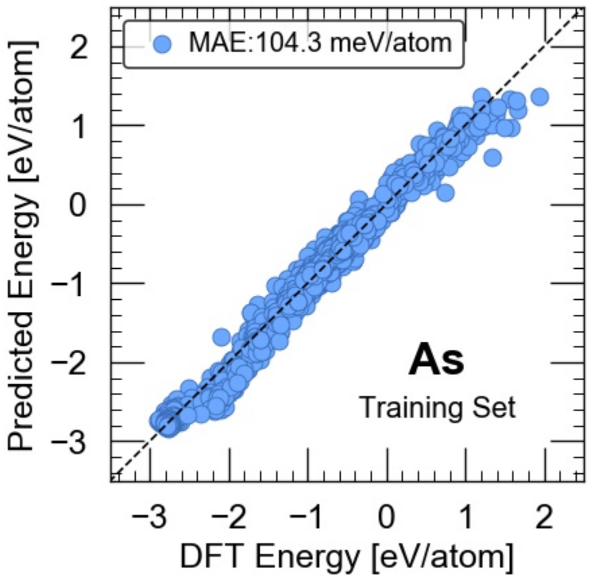

(a)

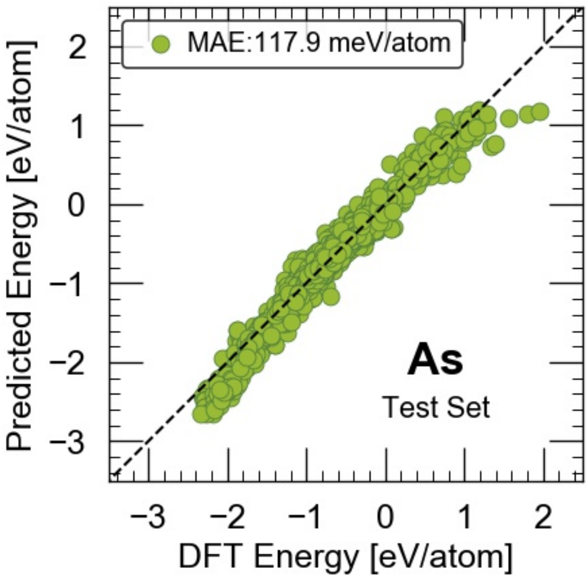

(b)

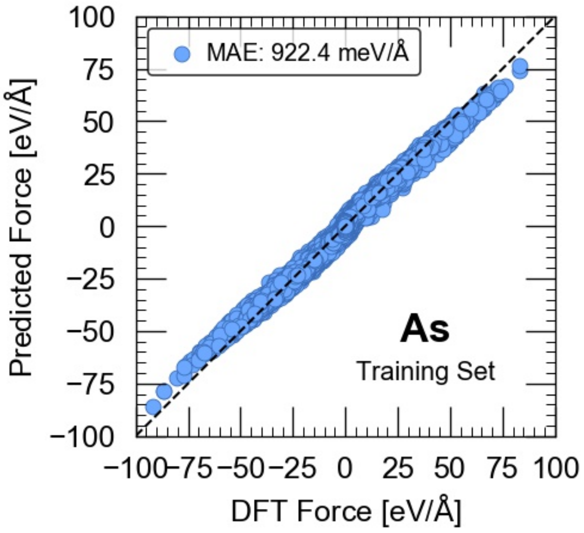

(c)

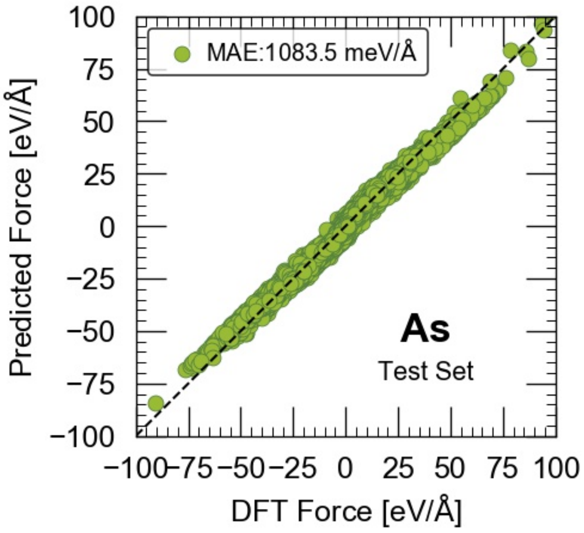

(d)

Supplementary Figure 100: Energy and force correlation plots for As nanoclusters

9.5 Au

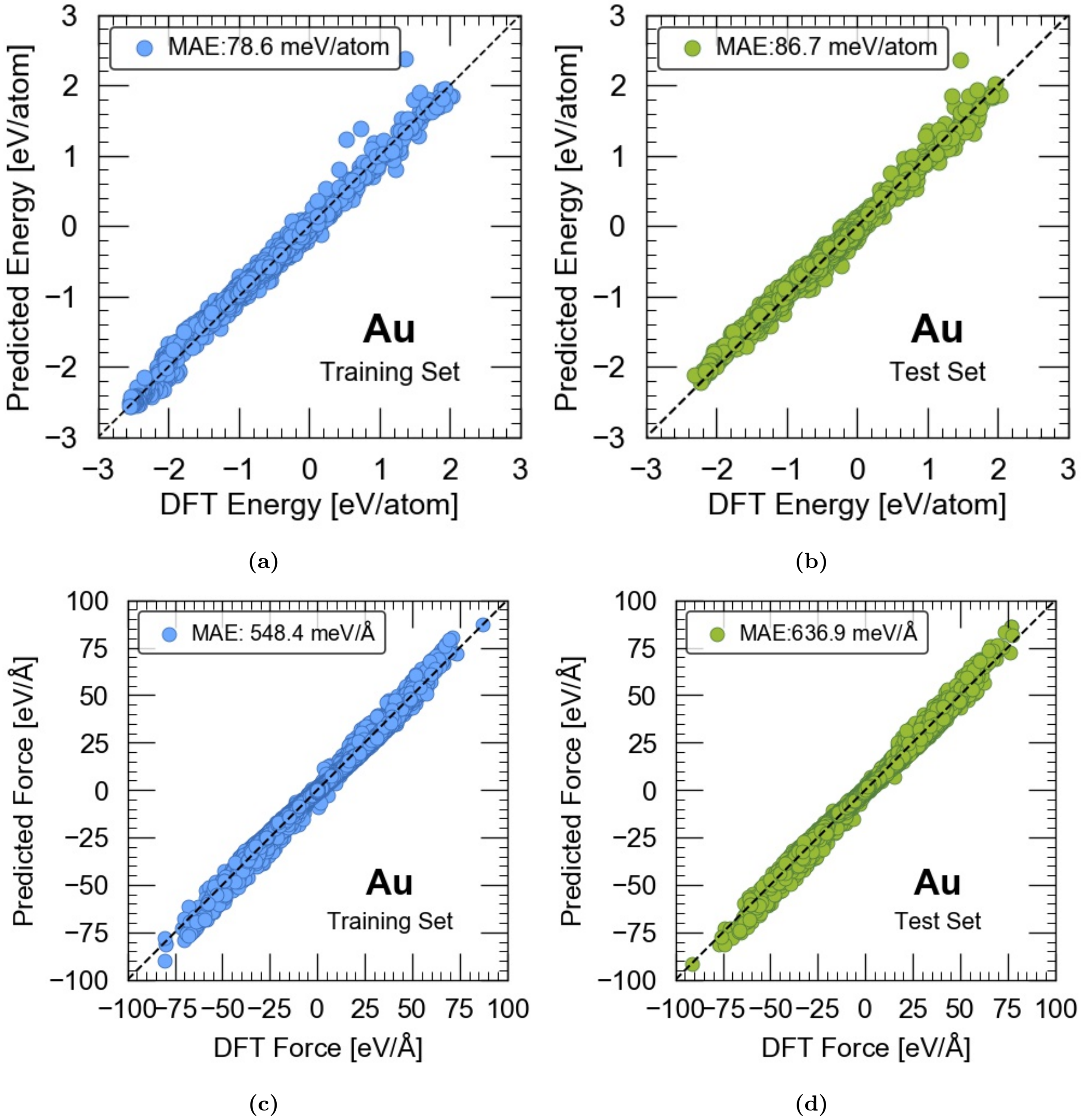

Supplementary Figure 101: Energy and force correlation plots for Au nanoclusters

9.6 B

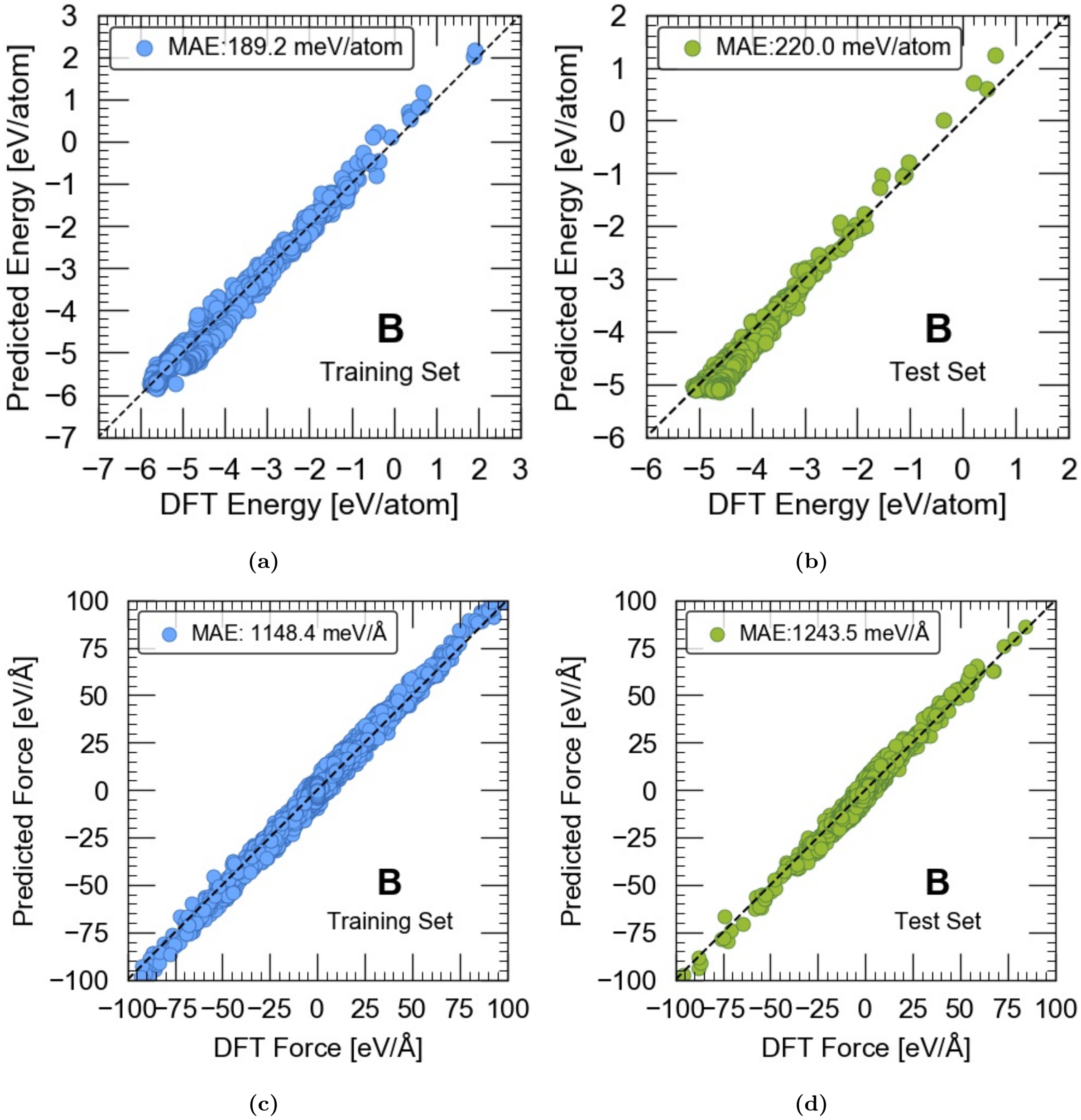

Supplementary Figure 102: Energy and force correlation plots for B nanoclusters

9.7 Ba

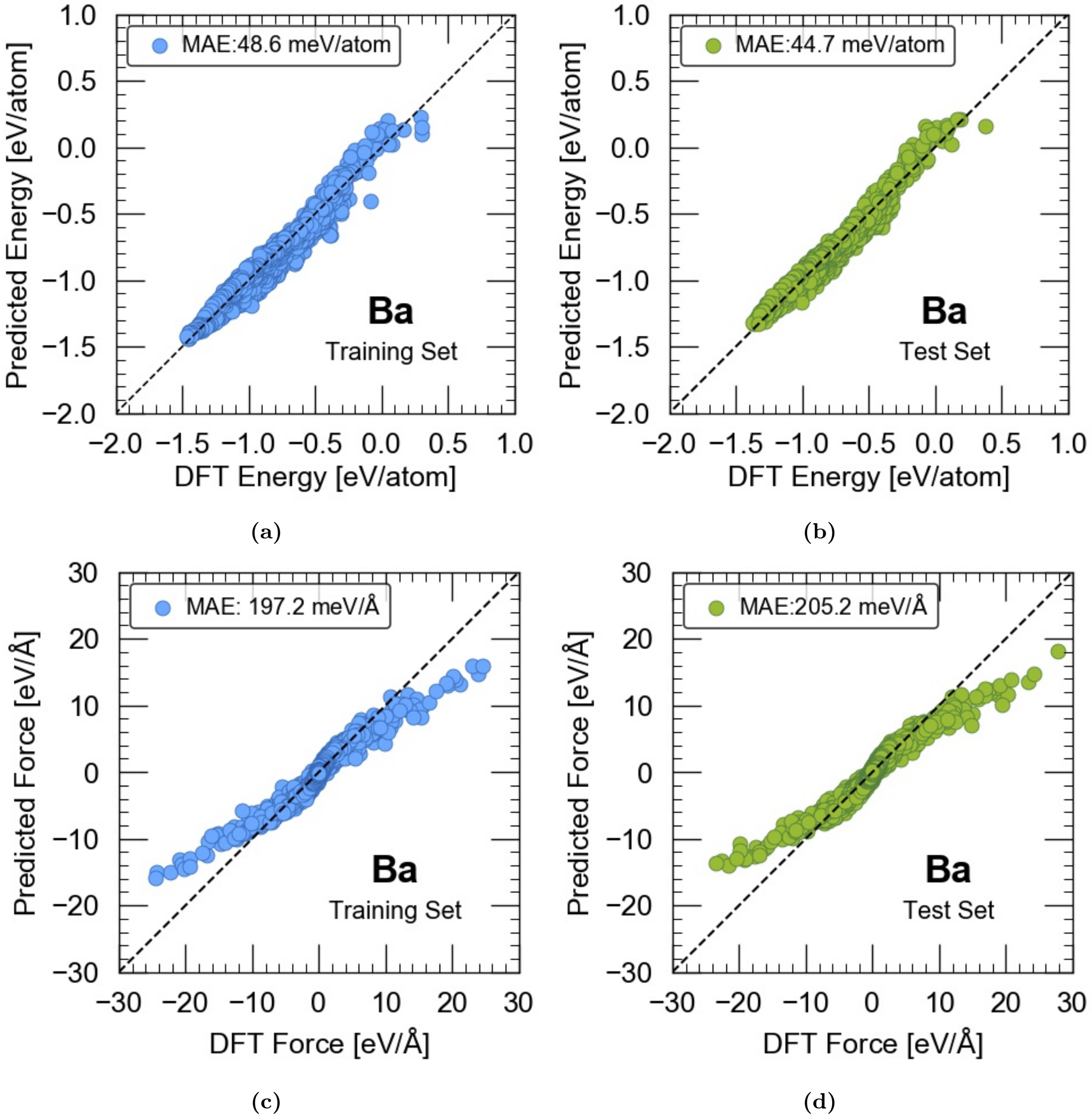

Supplementary Figure 103: Energy and force correlation plots for Ba nanoclusters

9.8 Be

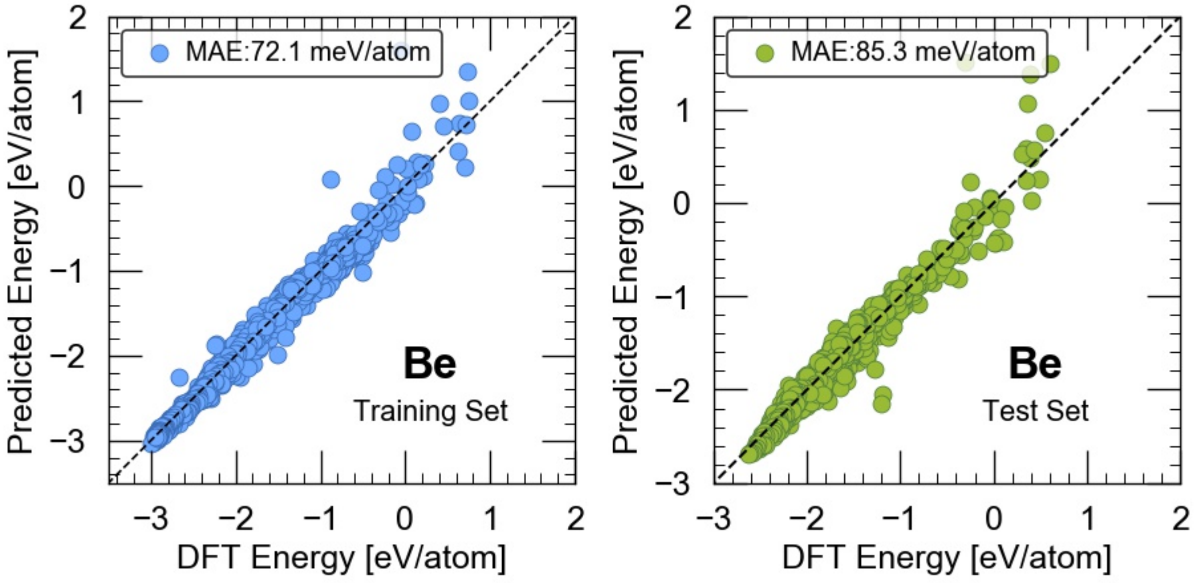

(a) (b)

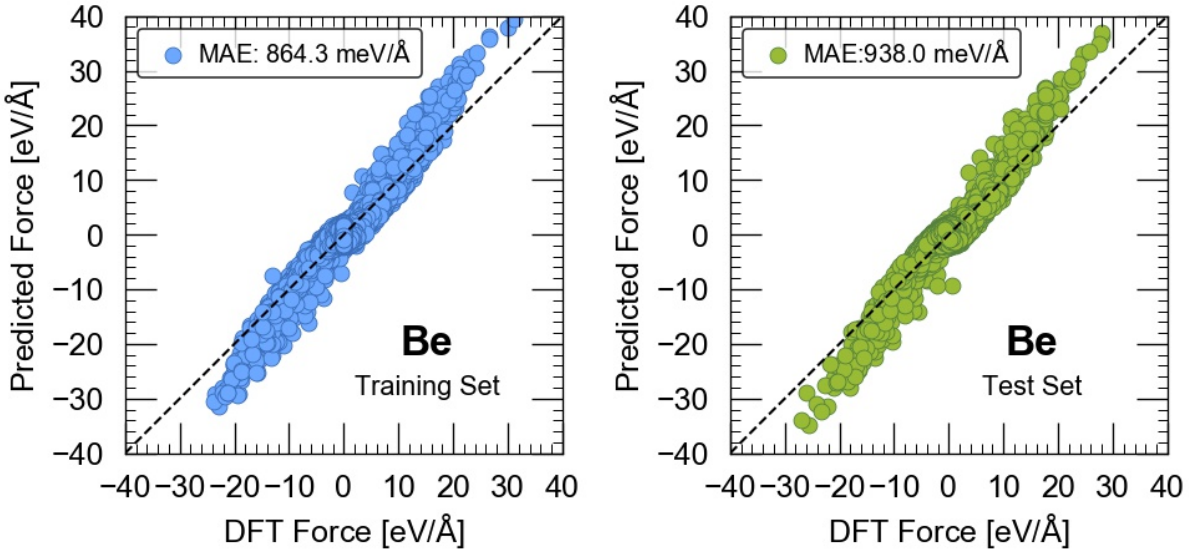

(c) (d)

Supplementary Figure 104: Energy and force correlation plots for Be nanoclusters

9.9 Bi

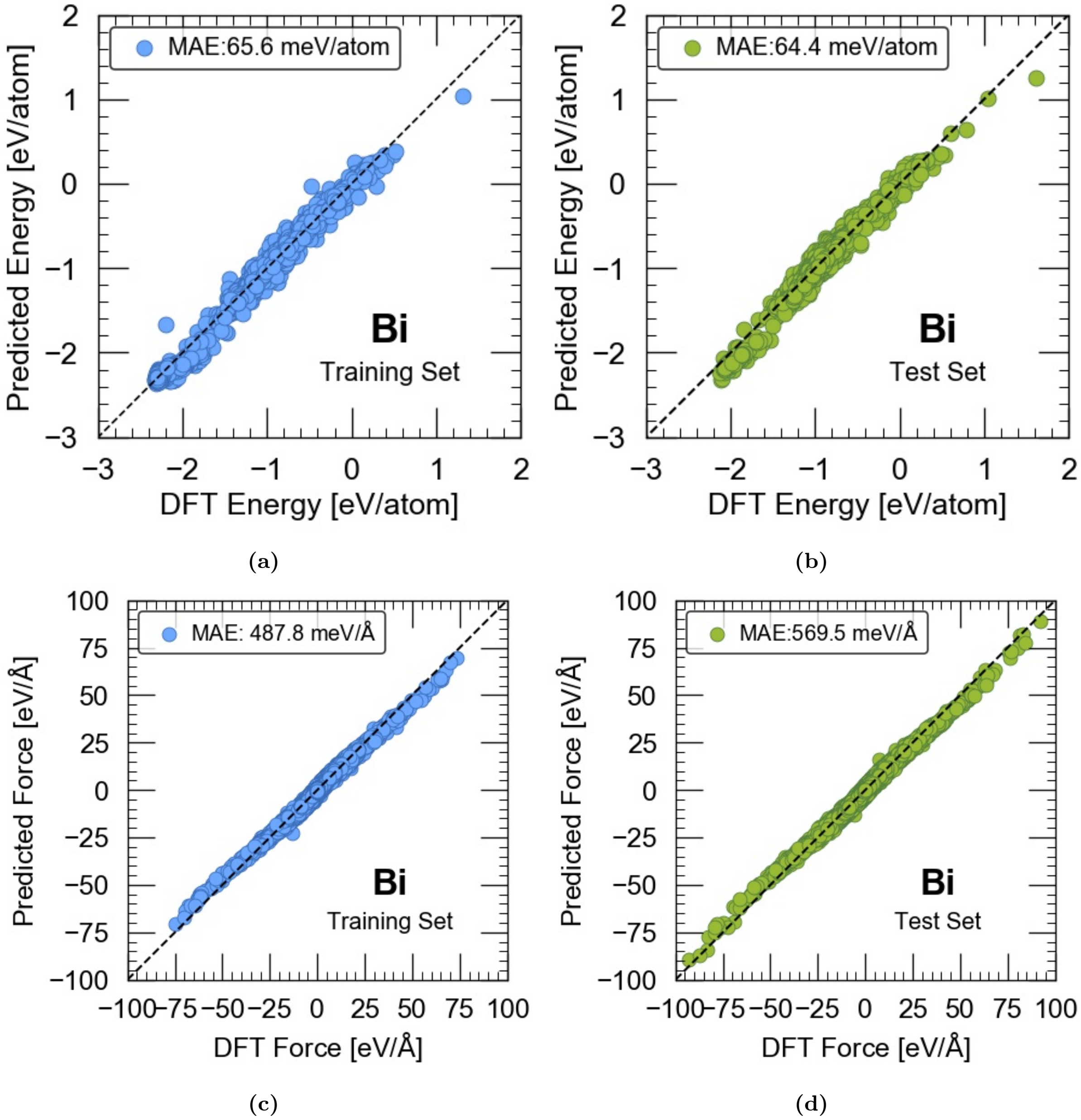

Supplementary Figure 105: Energy and force correlation plots for Bi nanoclusters

9.10 C

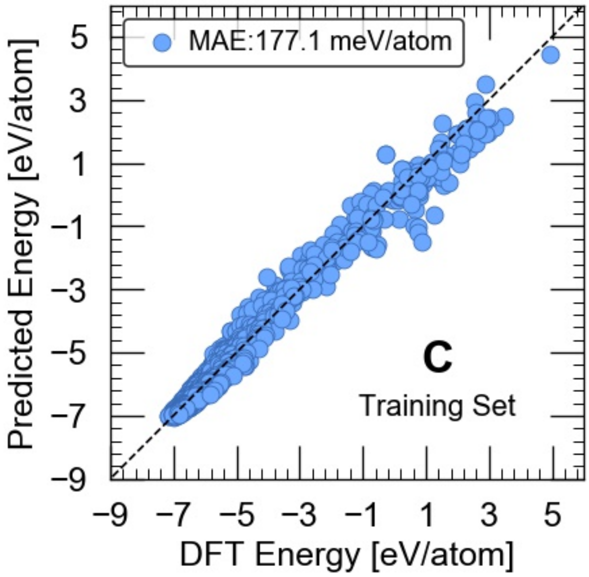

(a)

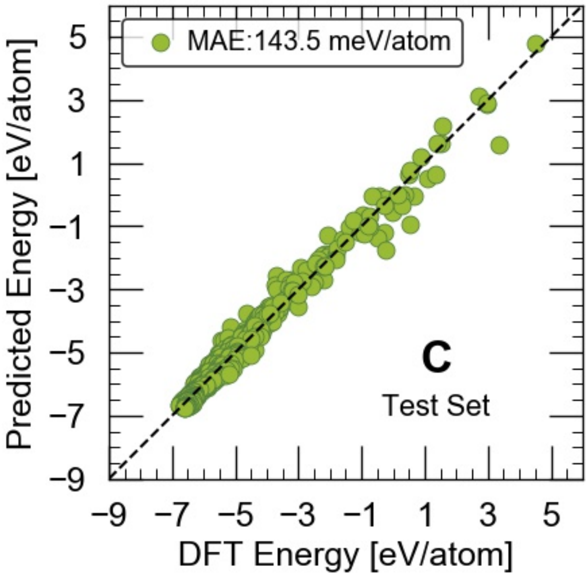

(b)

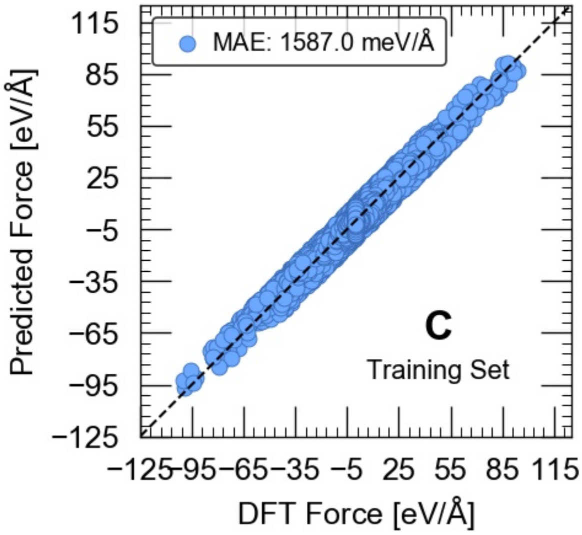

(c)

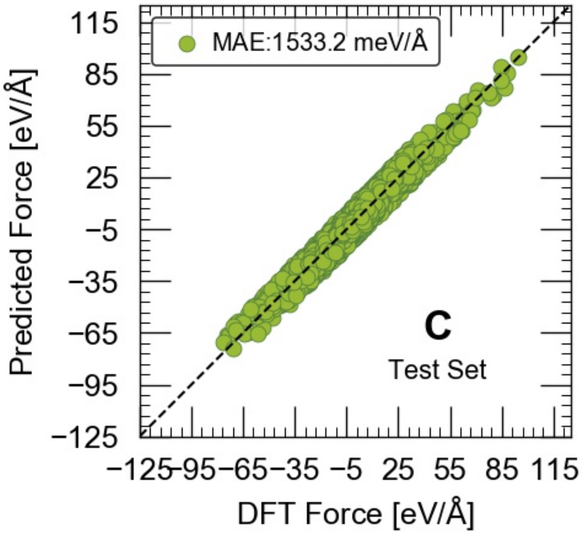

(d)

Supplementary Figure 106: Energy and force correlation plots for C nanoclusters

9.11 Ca

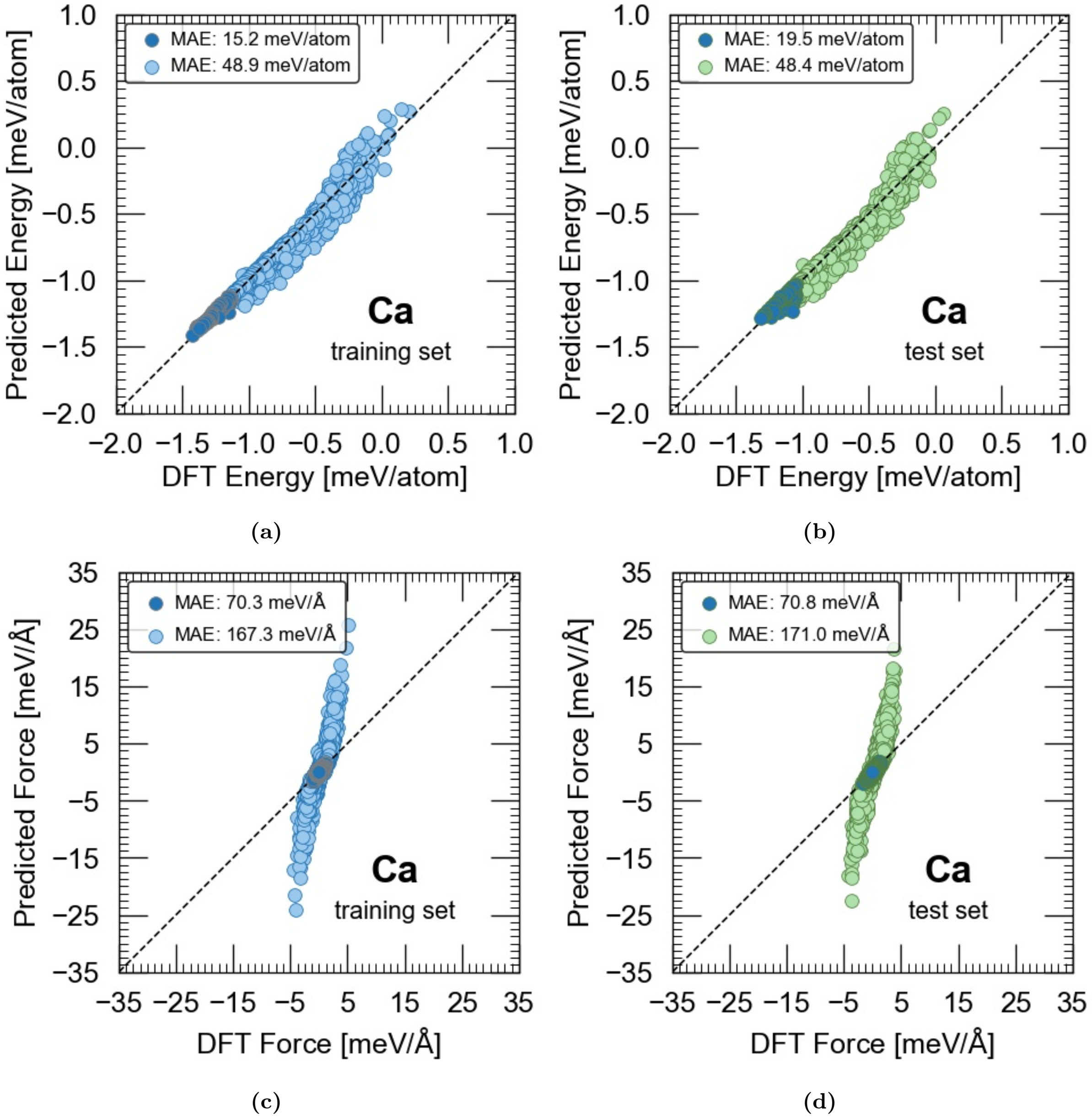

Supplementary Figure 107: Energy and force correlation plots for Ca nanoclusters

9.12 Cd

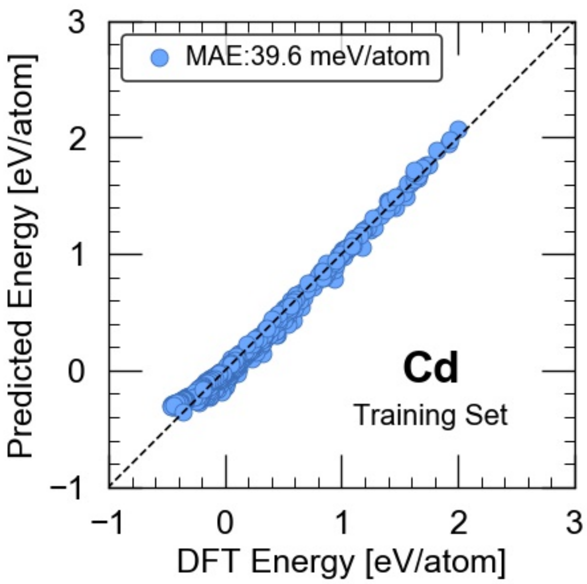

(a)

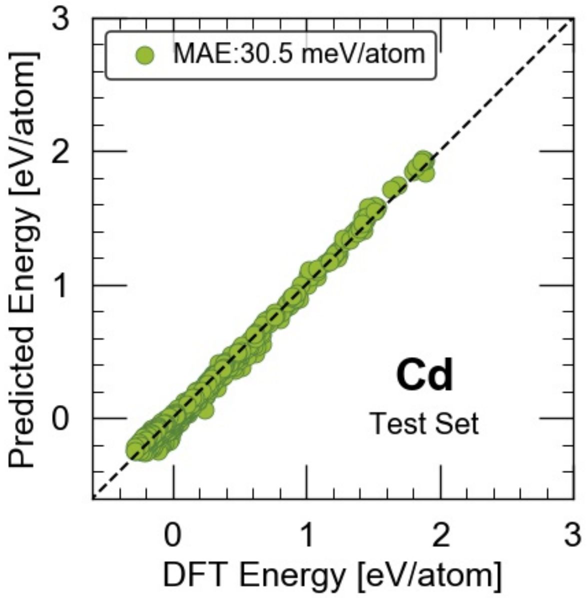

(b)

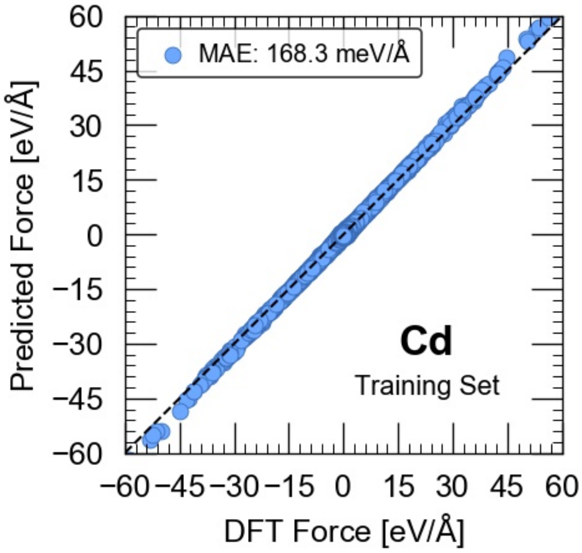

(c)

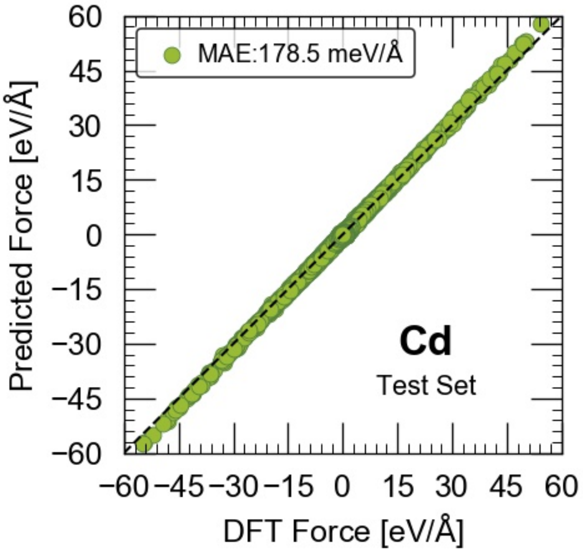

(d)

Supplementary Figure 108: Energy and force correlation plots for Cd nanoclusters

9.13 Co

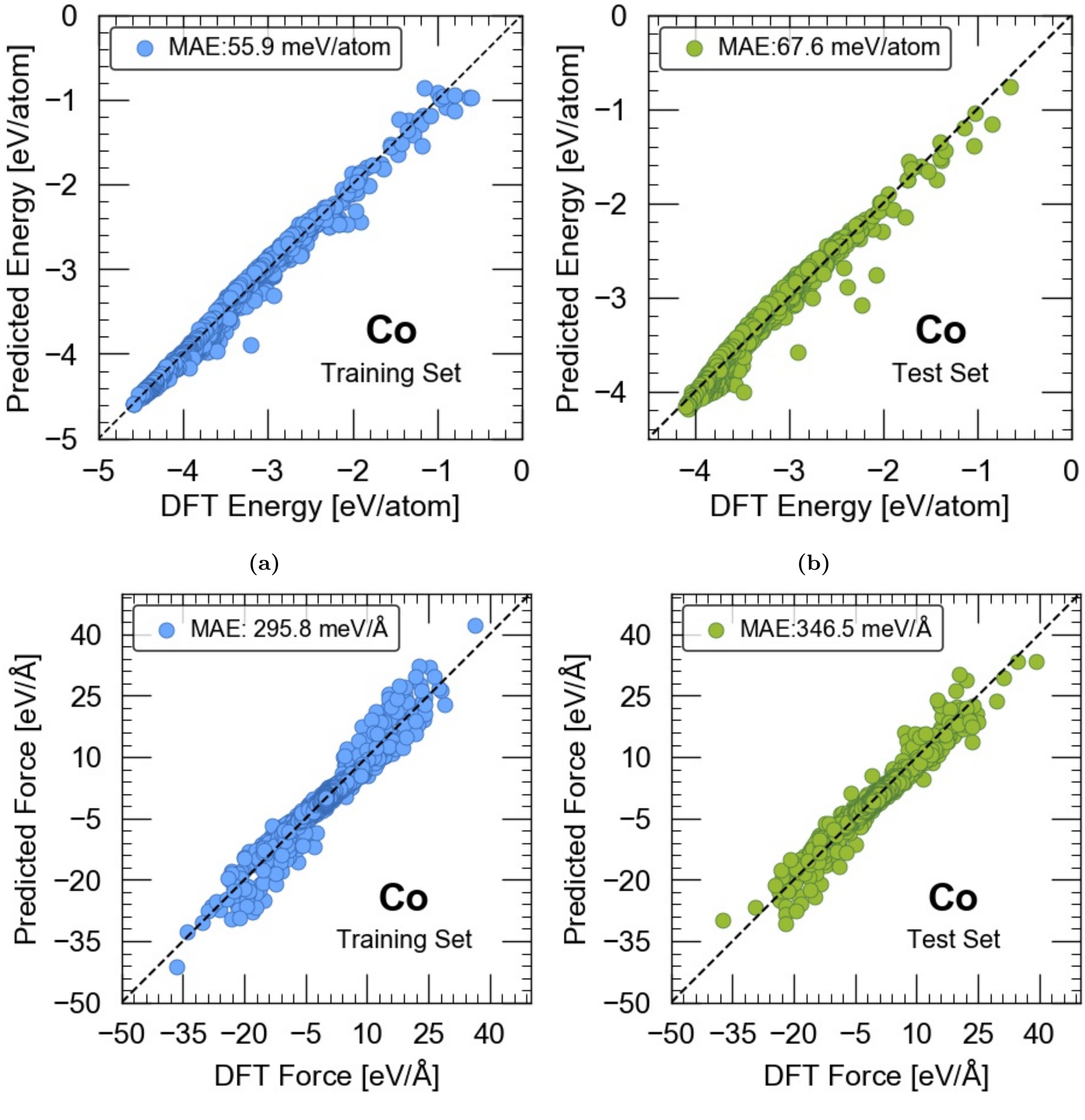

(a) (b) (c) (d)

Supplementary Figure 109: Energy and force correlation plots for Co nanoclusters

9.14 Cr

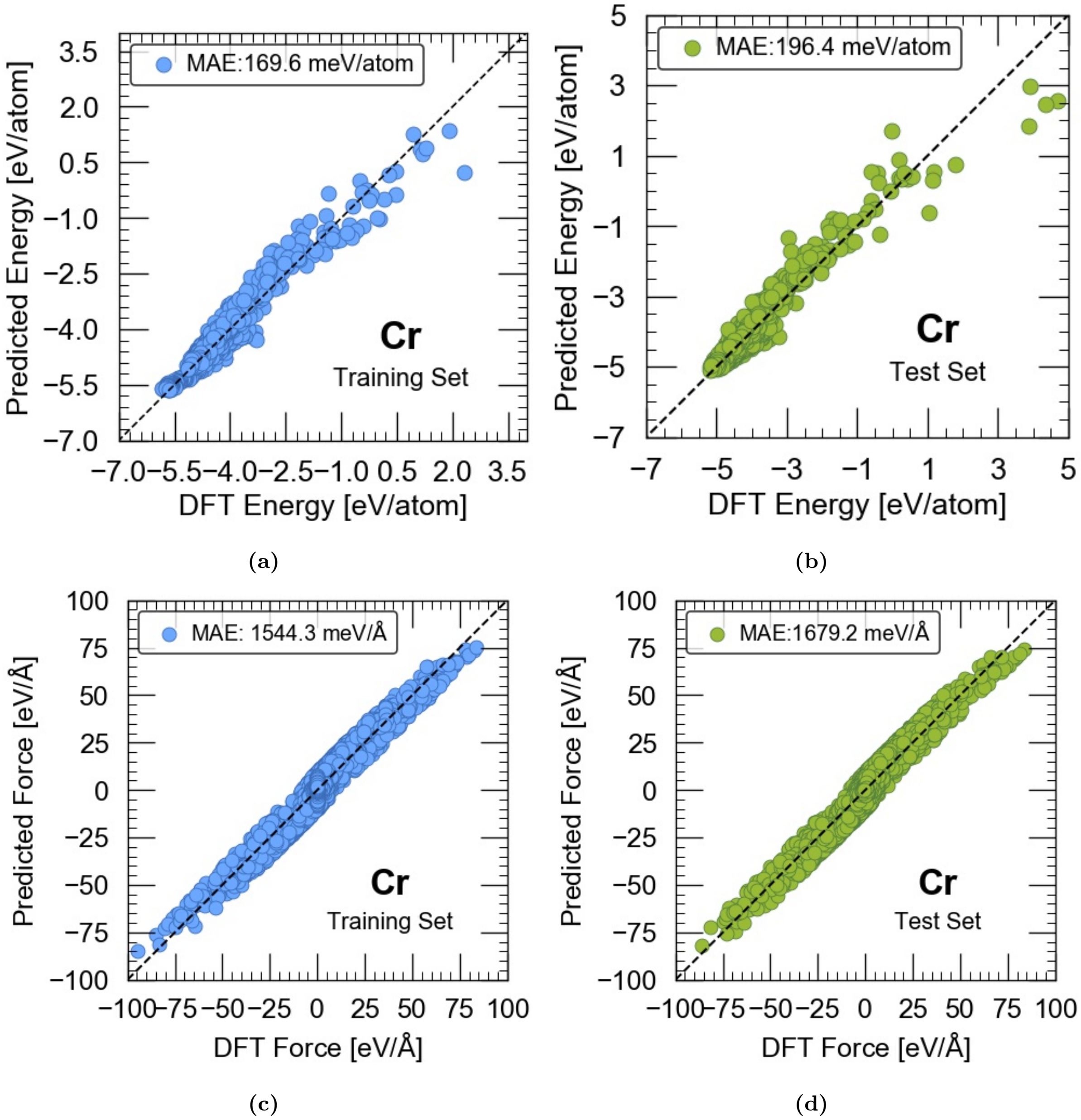

Supplementary Figure 110: Energy and force correlation plots for Cr nanoclusters

9.15 Cs

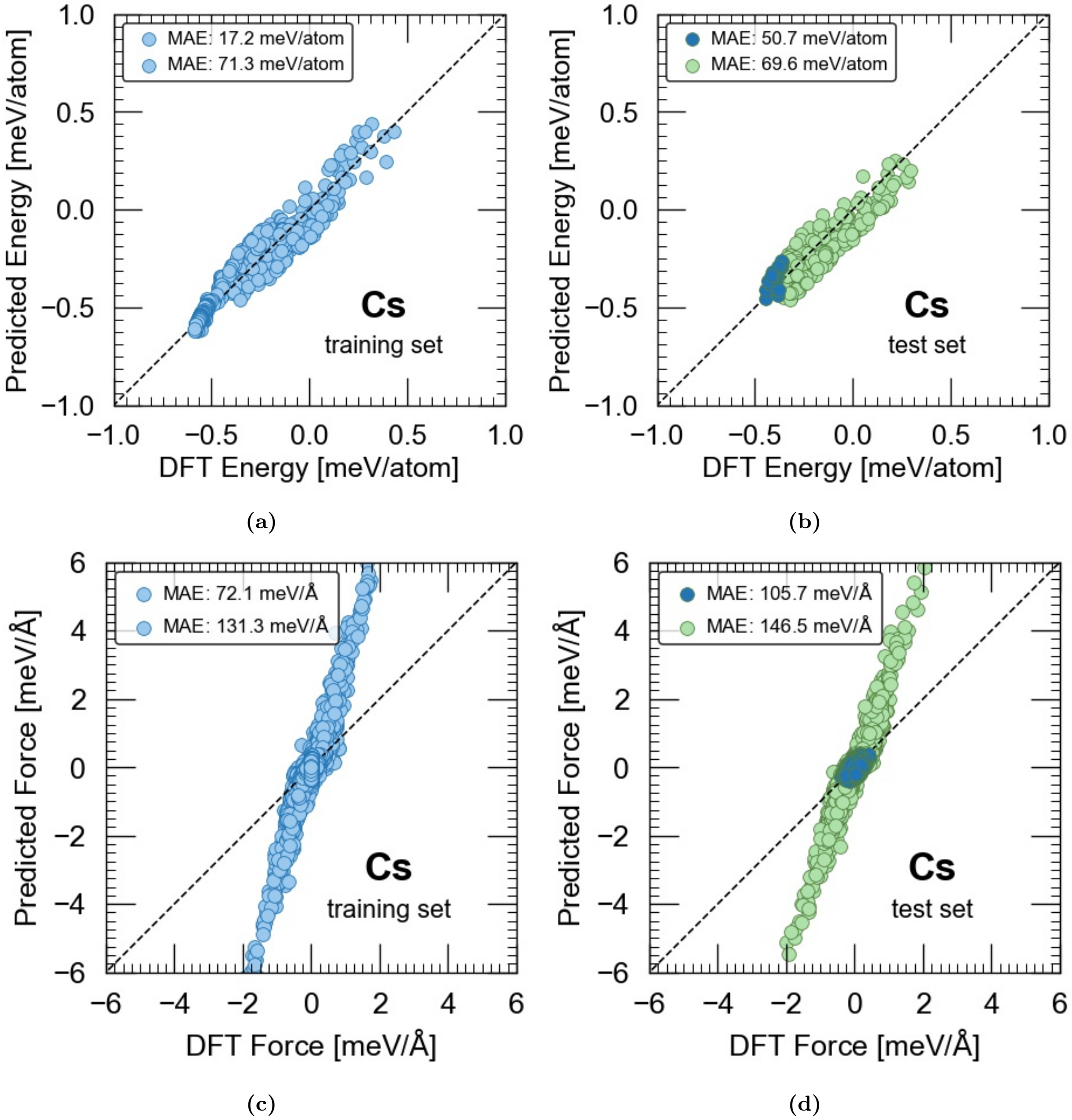

Supplementary Figure 111: Energy and force correlation plots for Cs nanoclusters

9.16 Cu

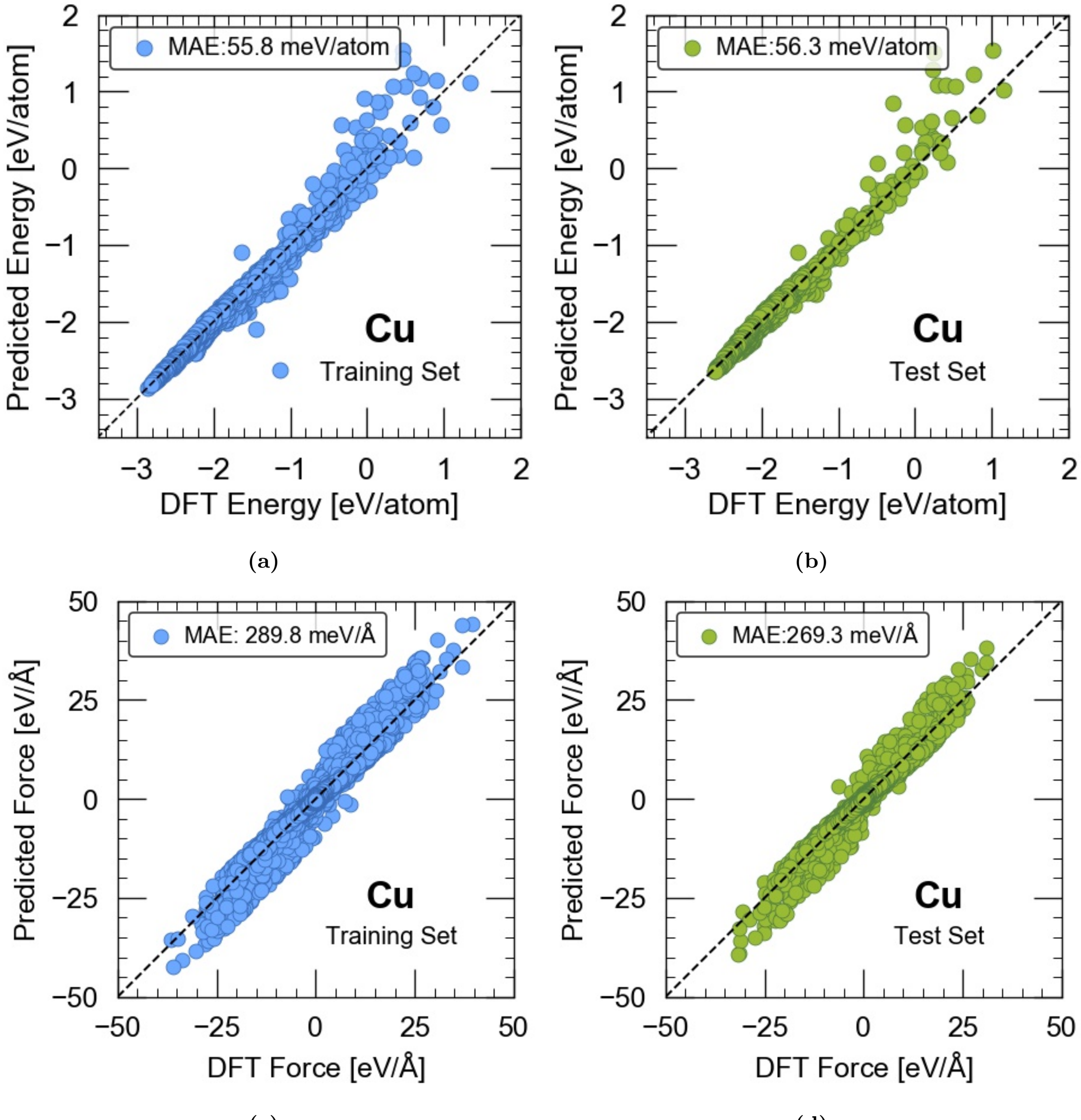

(a) (b) (c) (d)

Supplementary Figure 112: Energy and force correlation plots for Cu nanoclusters

9.17 Fe

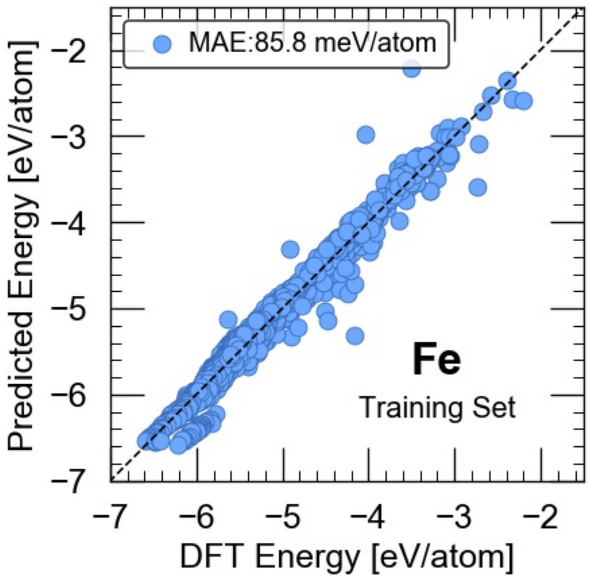

(a)

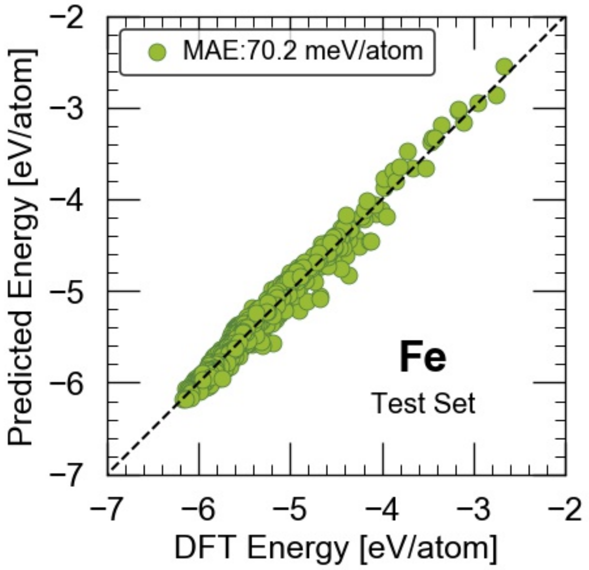

(b)

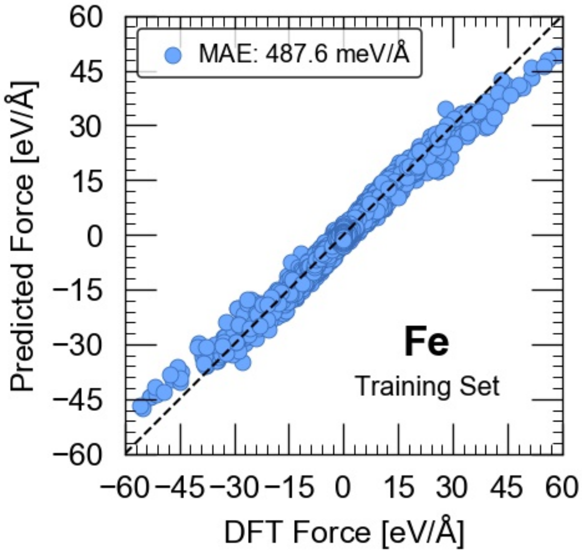

(c)

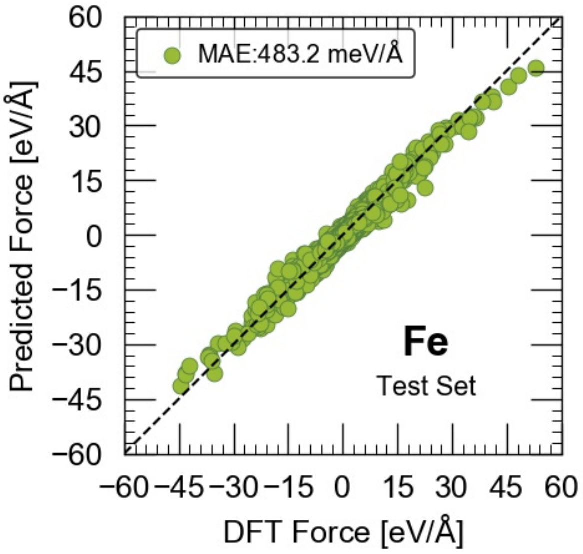

(d)

Supplementary Figure 113: Energy and force correlation plots for Fe nanoclusters

9.18 Ga

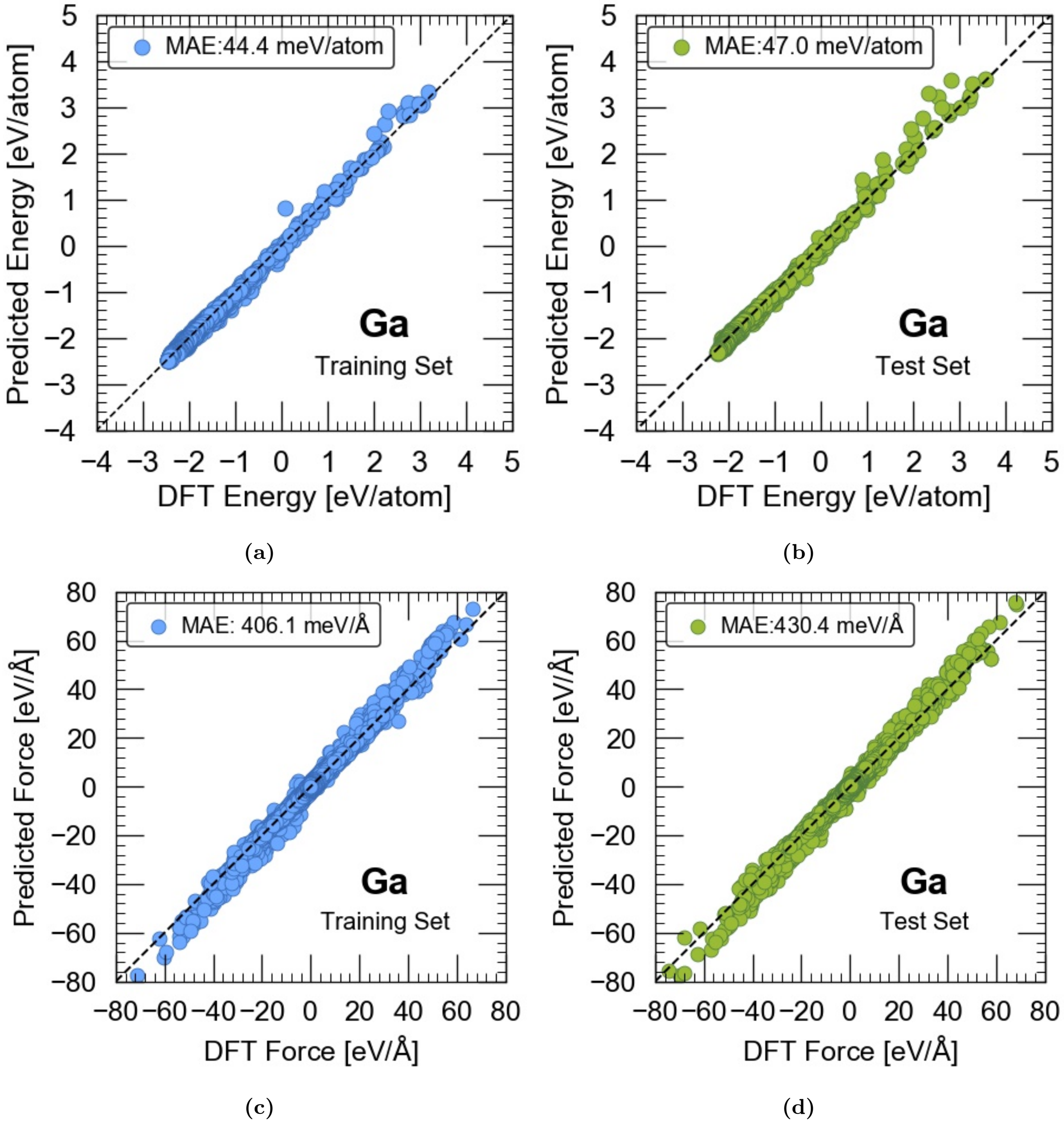

Supplementary Figure 114: Energy and force correlation plots for Ga nanoclusters

9.19 Ge

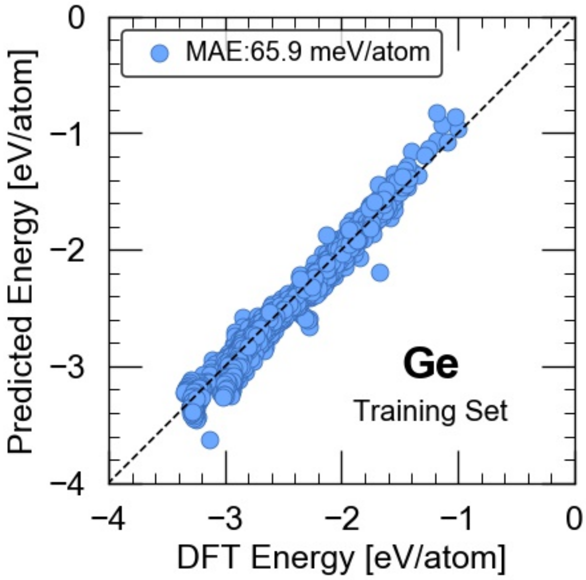

(a)

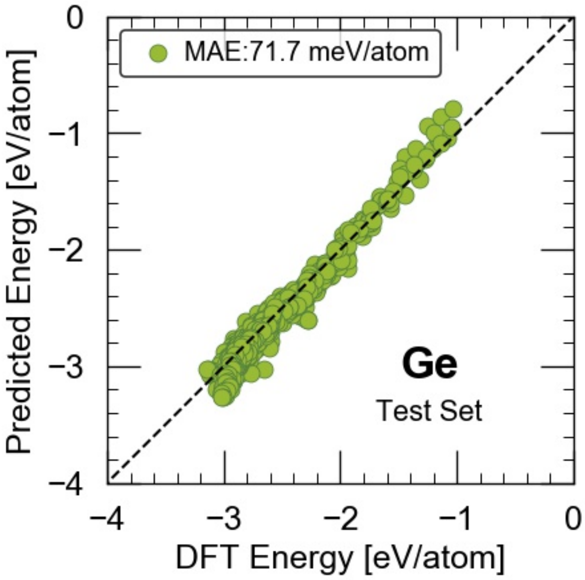

(b)

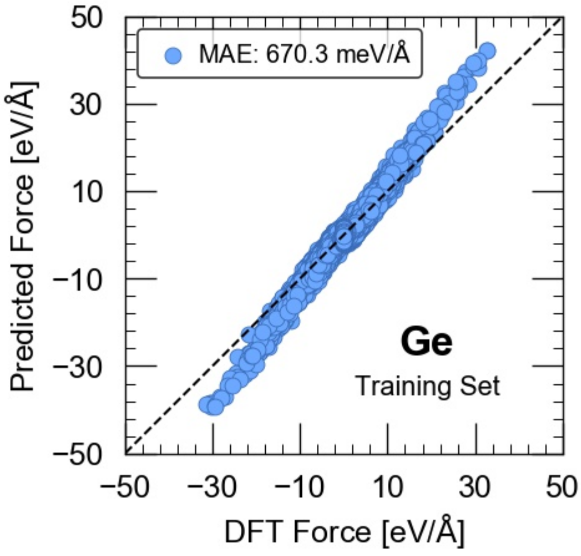

(c)

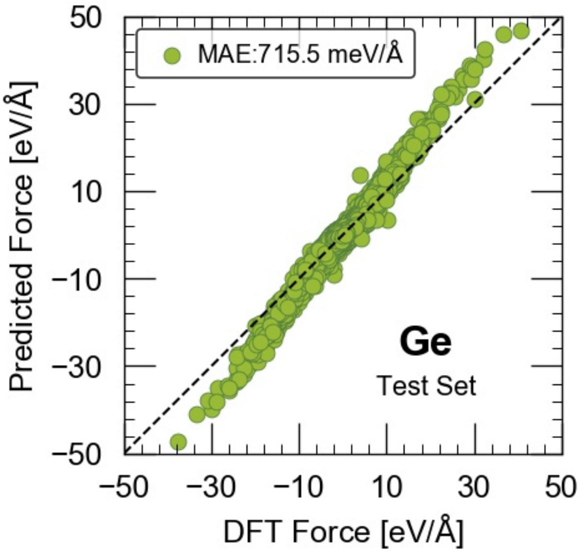

(d)

Supplementary Figure 115: Energy and force correlation plots for Ge nanoclusters

9.20 Hf

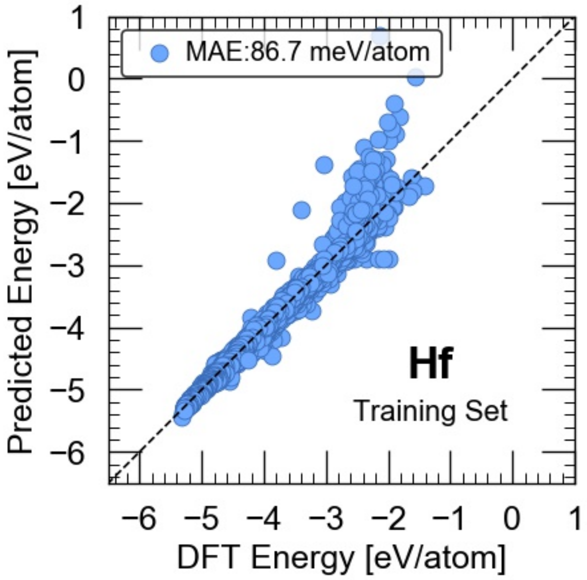

(a)

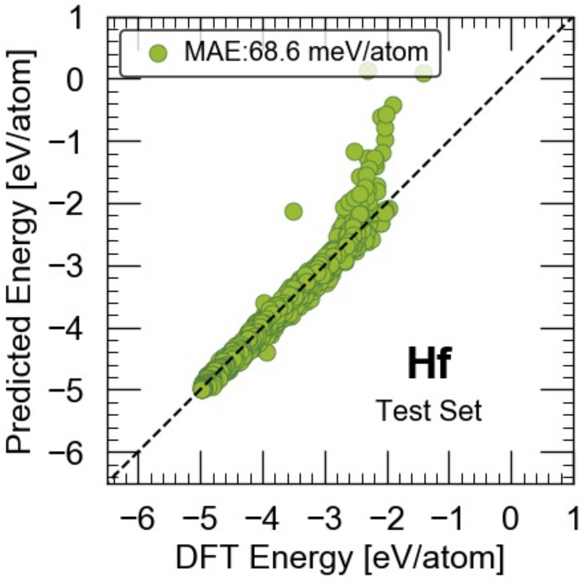

(b)

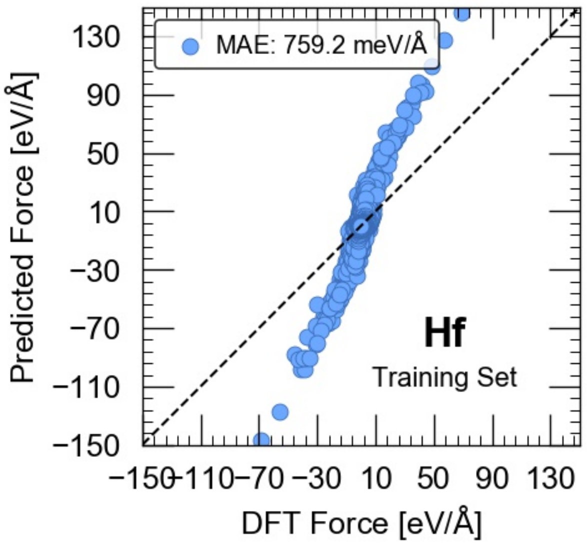

(c)

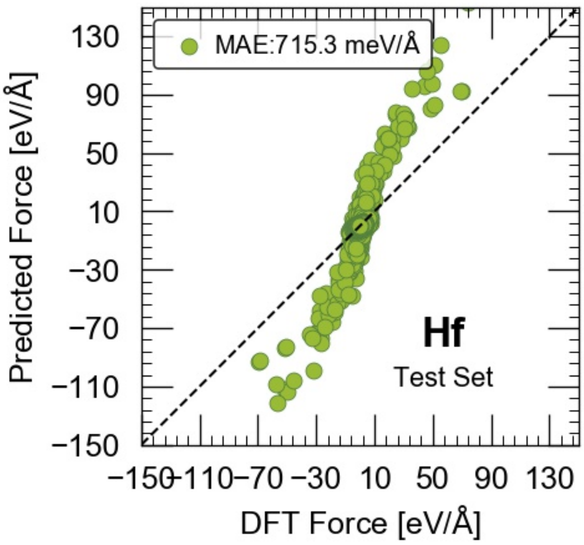

(d)

Supplementary Figure 116: Energy and force correlation plots for Hf nanoclusters

9.21 In

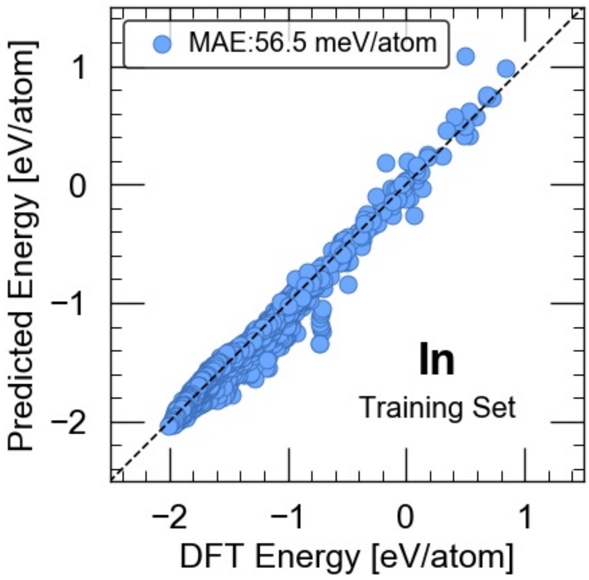

(a)

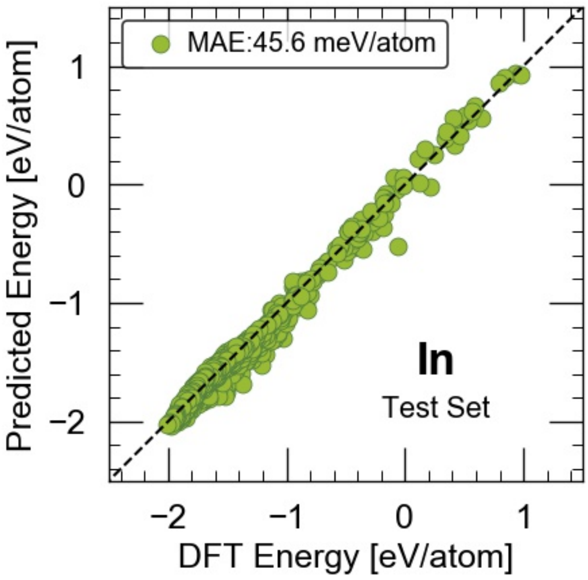

(b)

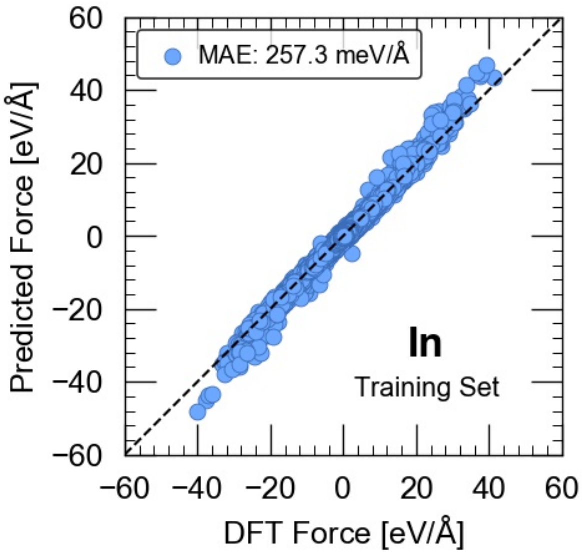

(c)

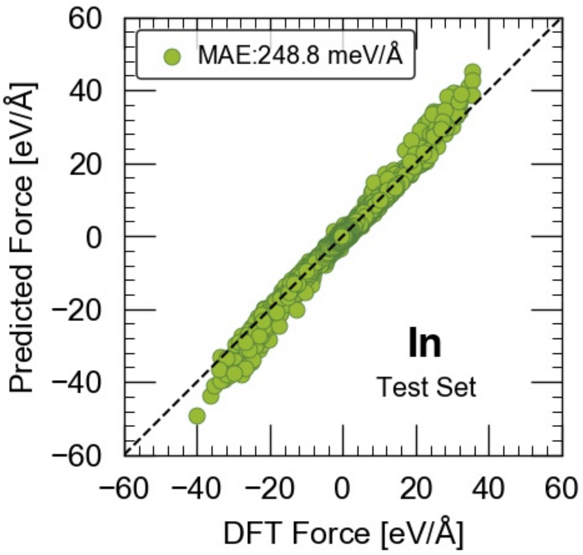

(d)

Supplementary Figure 117: Energy and force correlation plots for In nanoclusters

9.22 Ir

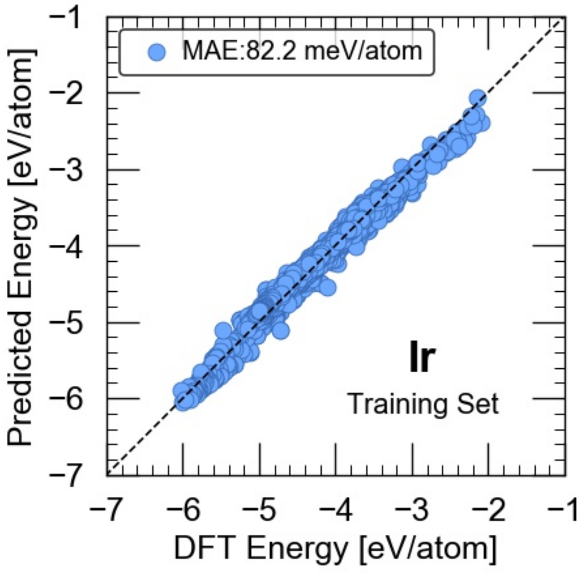

(a)

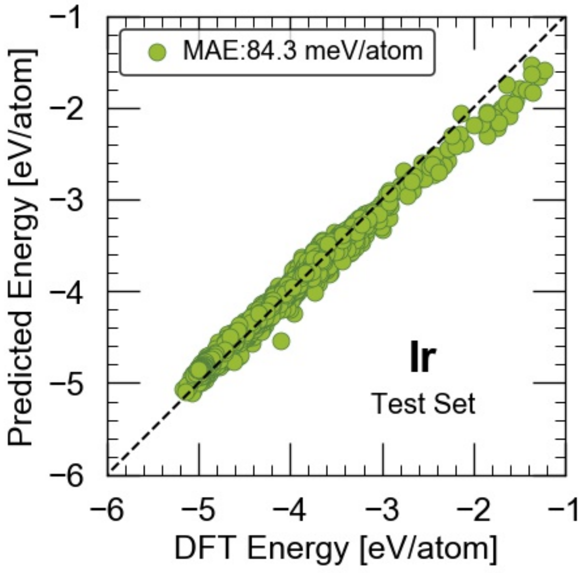

(b)

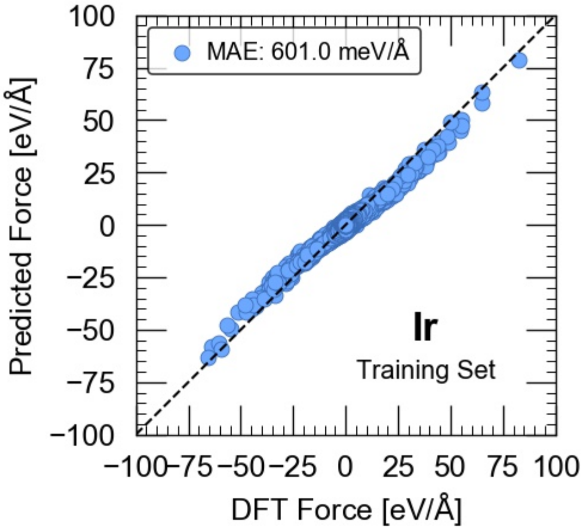

(c)

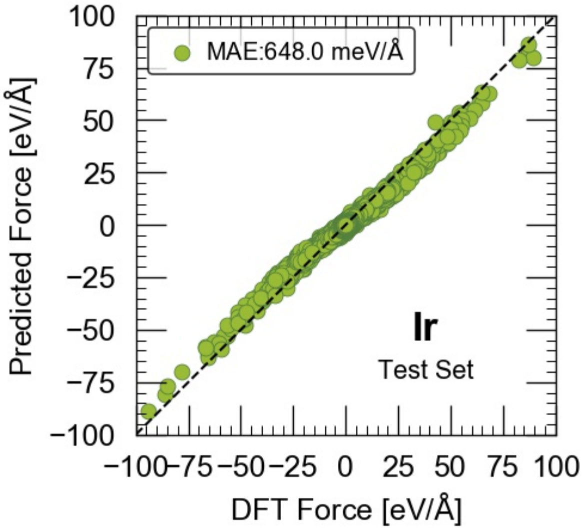

(d)

Supplementary Figure 118: Energy and force correlation plots for Ir nanoclusters

9.23 K

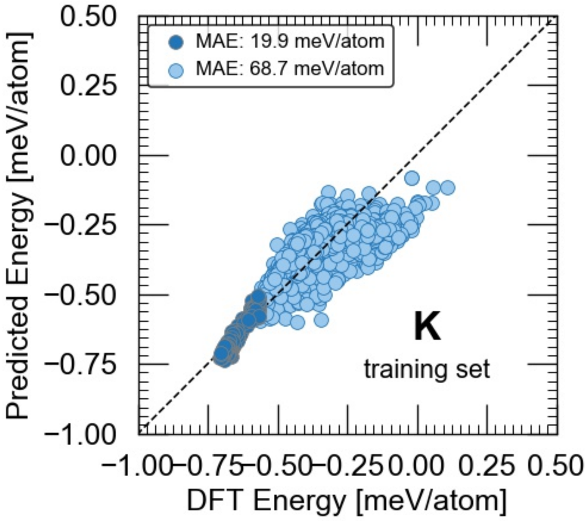

(a)

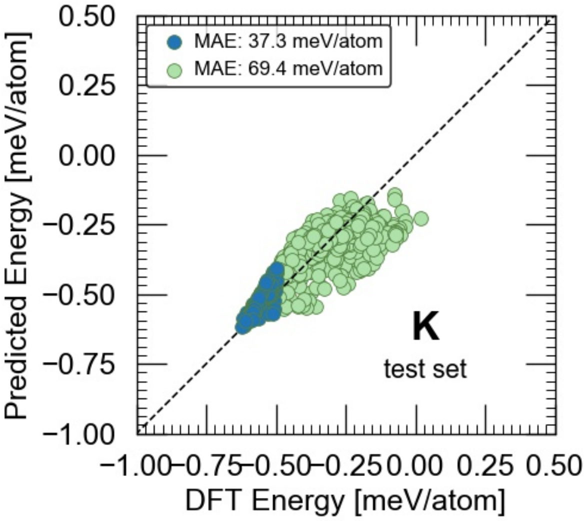

(b)

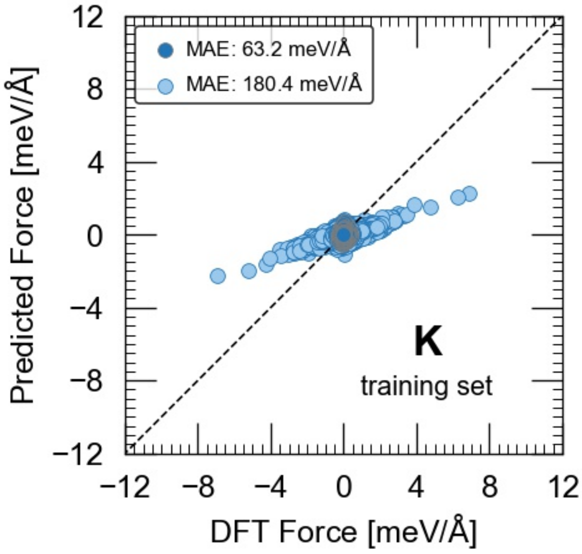

(c)

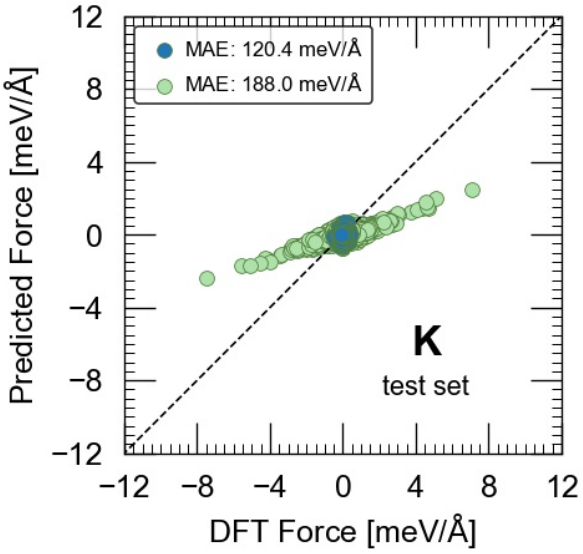

(d)

Supplementary Figure 119: Energy and force correlation plots for K nanoclusters

9.24 Li

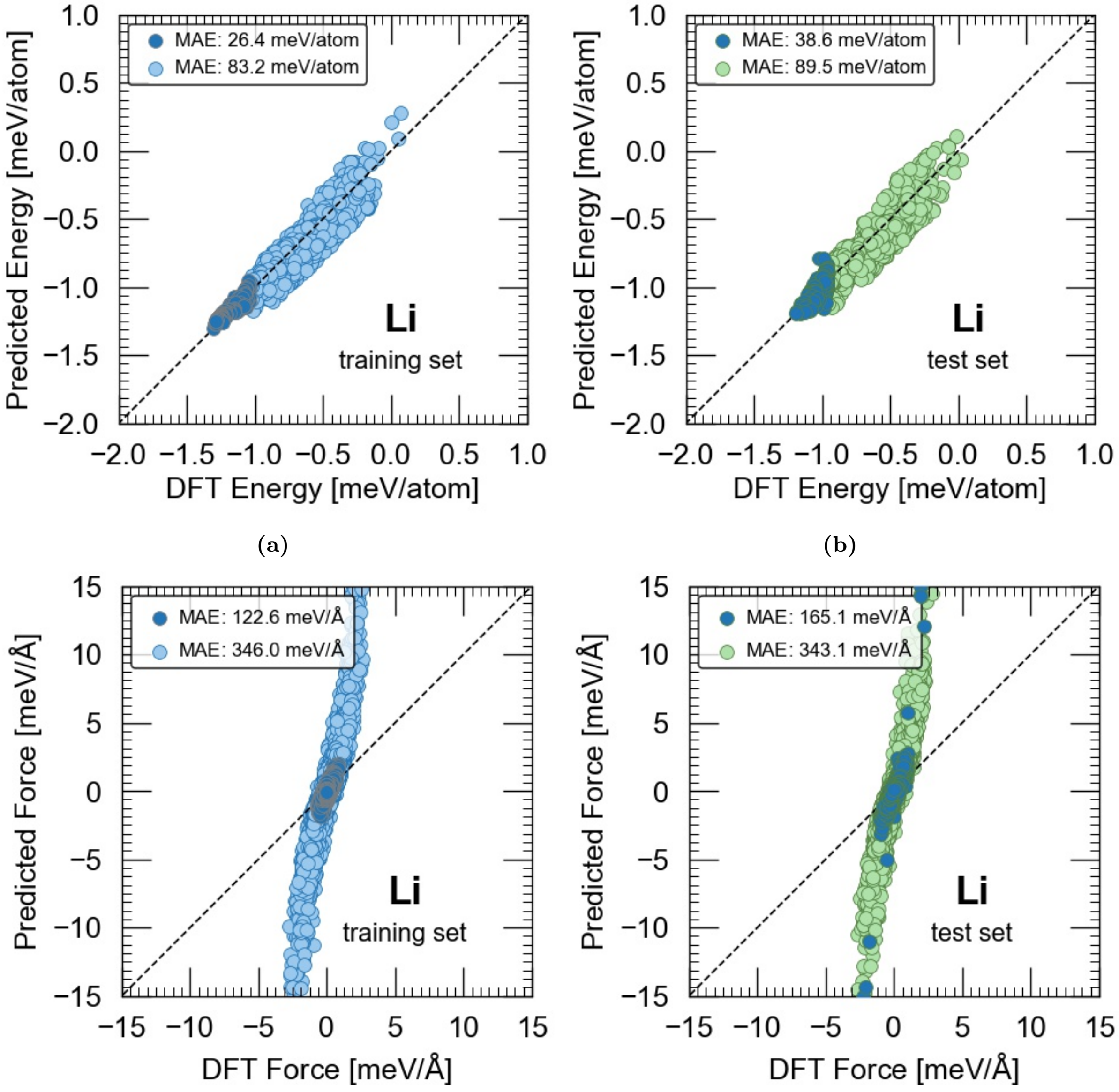

(a) **(b)**

(c) **(d)**

Supplementary Figure 120: Energy and force correlation plots for Li nanoclusters

9.25 Mg

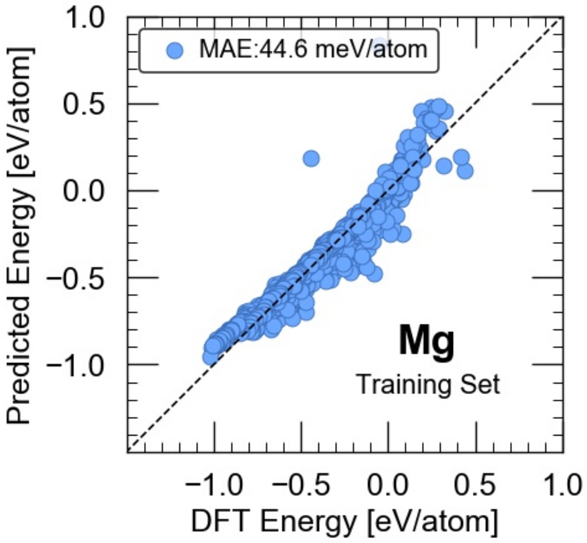

(a)

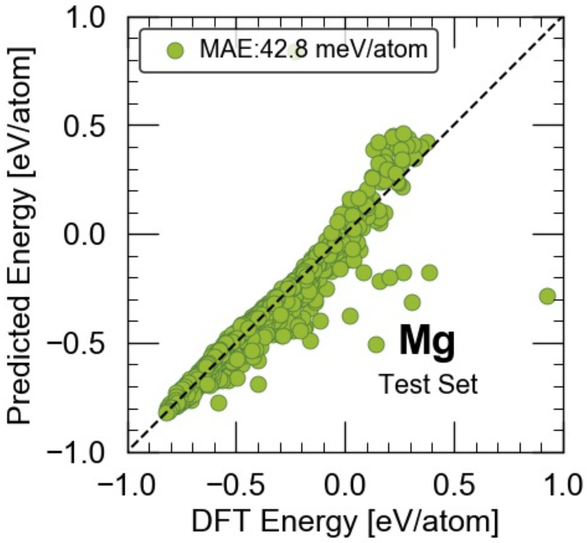

(b)

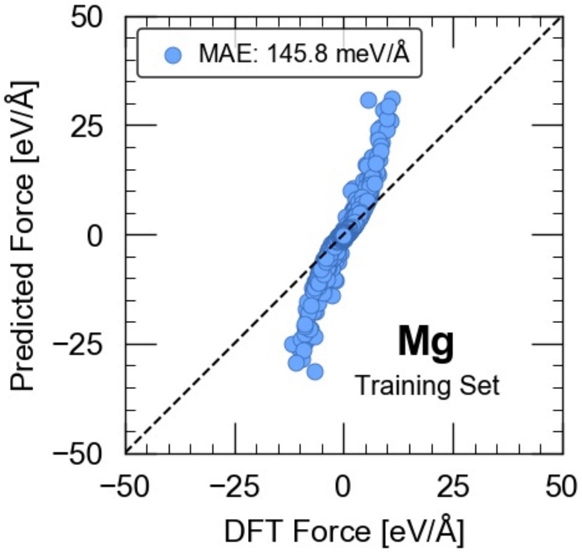

(c)

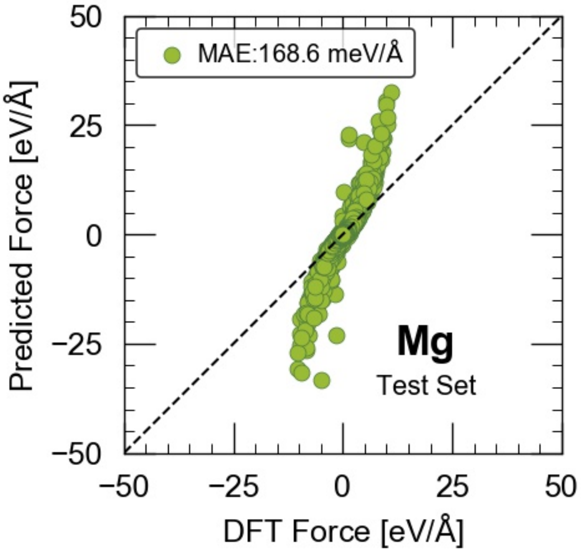

(d)

Supplementary Figure 121: Energy and force correlation plots for Mg nanoclusters

9.26 Mn

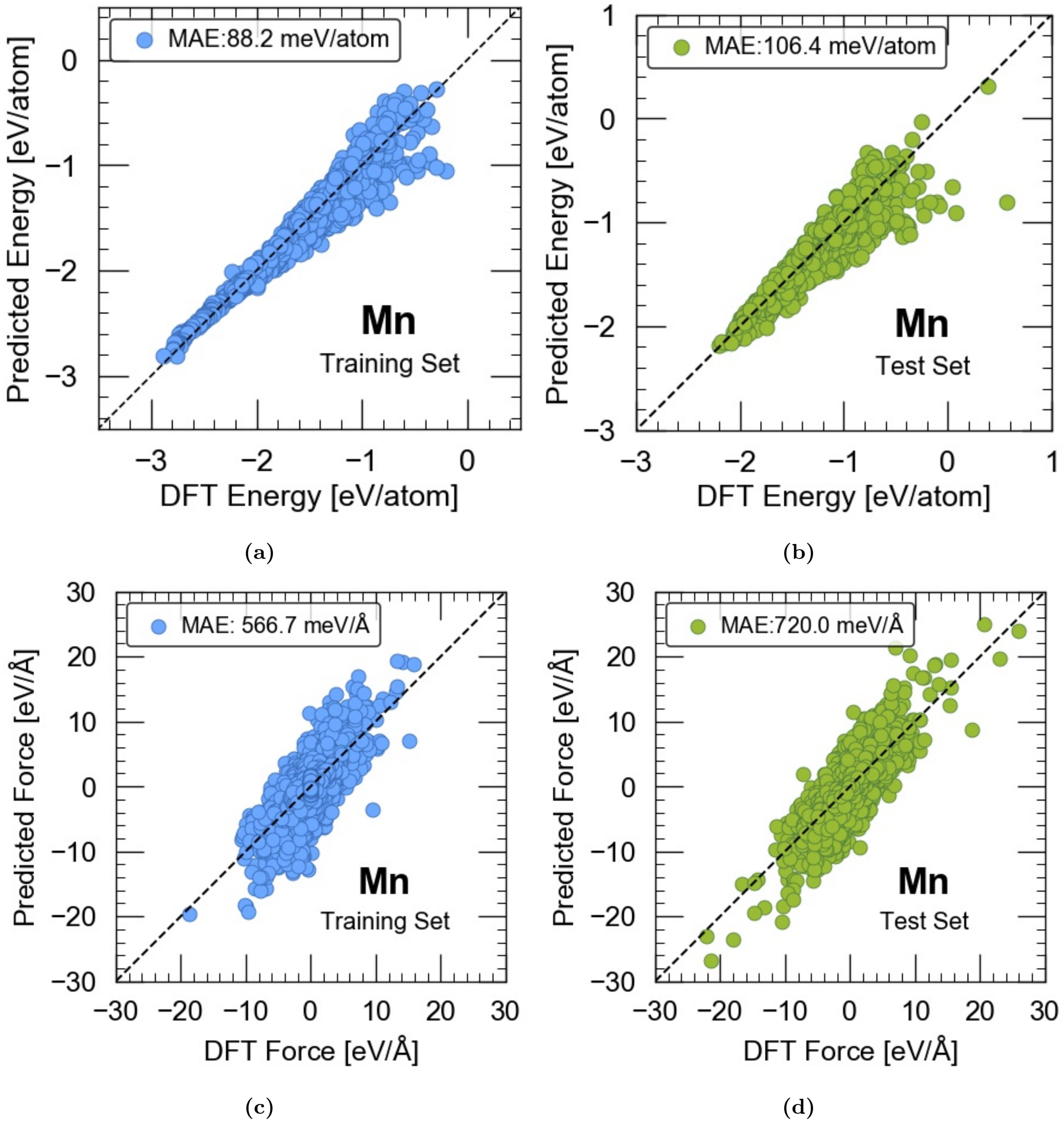

Supplementary Figure 122: Energy and force correlation plots for Mn nanoclusters

9.27 Mo

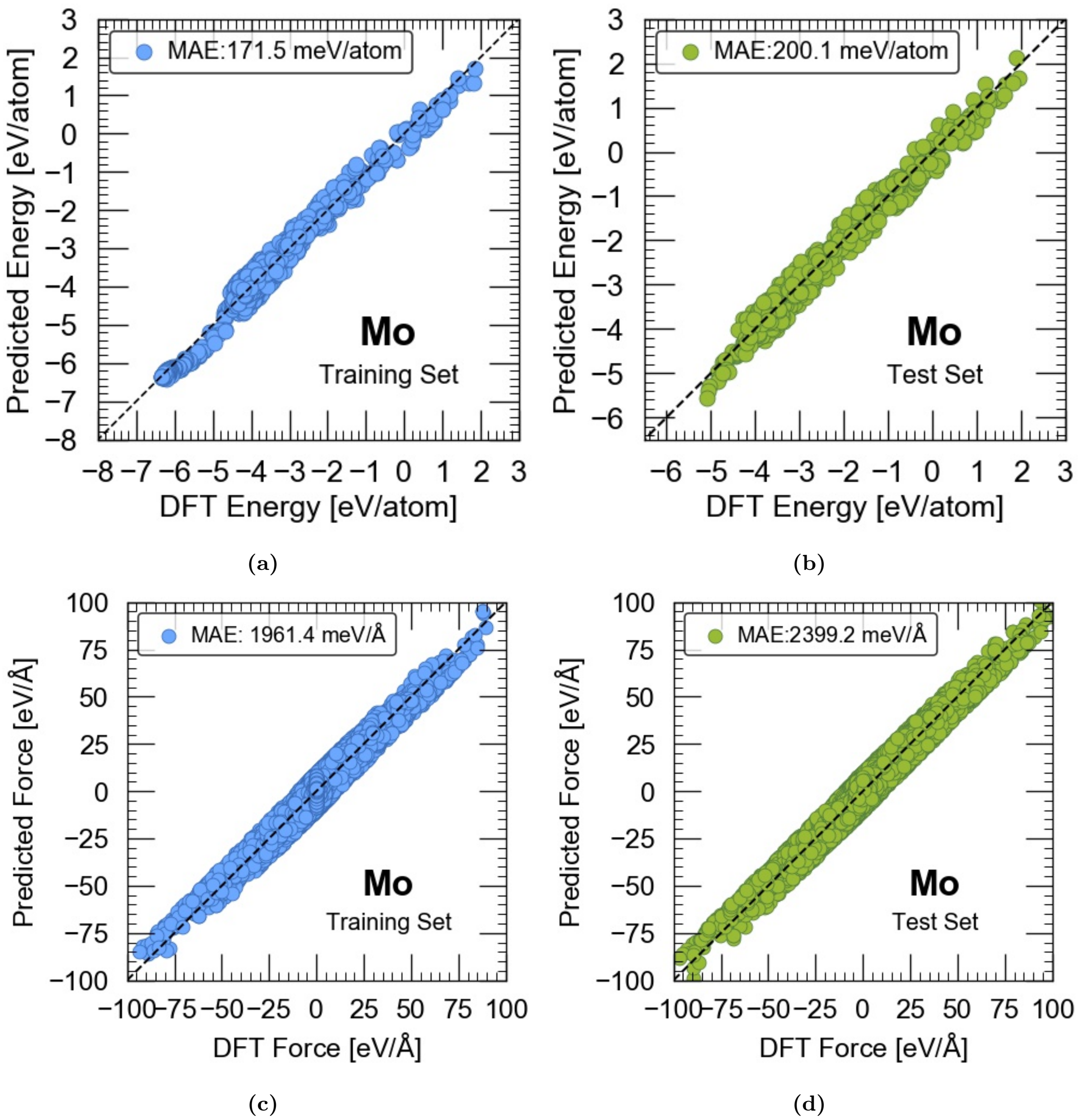

Supplementary Figure 123: Energy and force correlation plots for Mo nanoclusters

9.28 Na

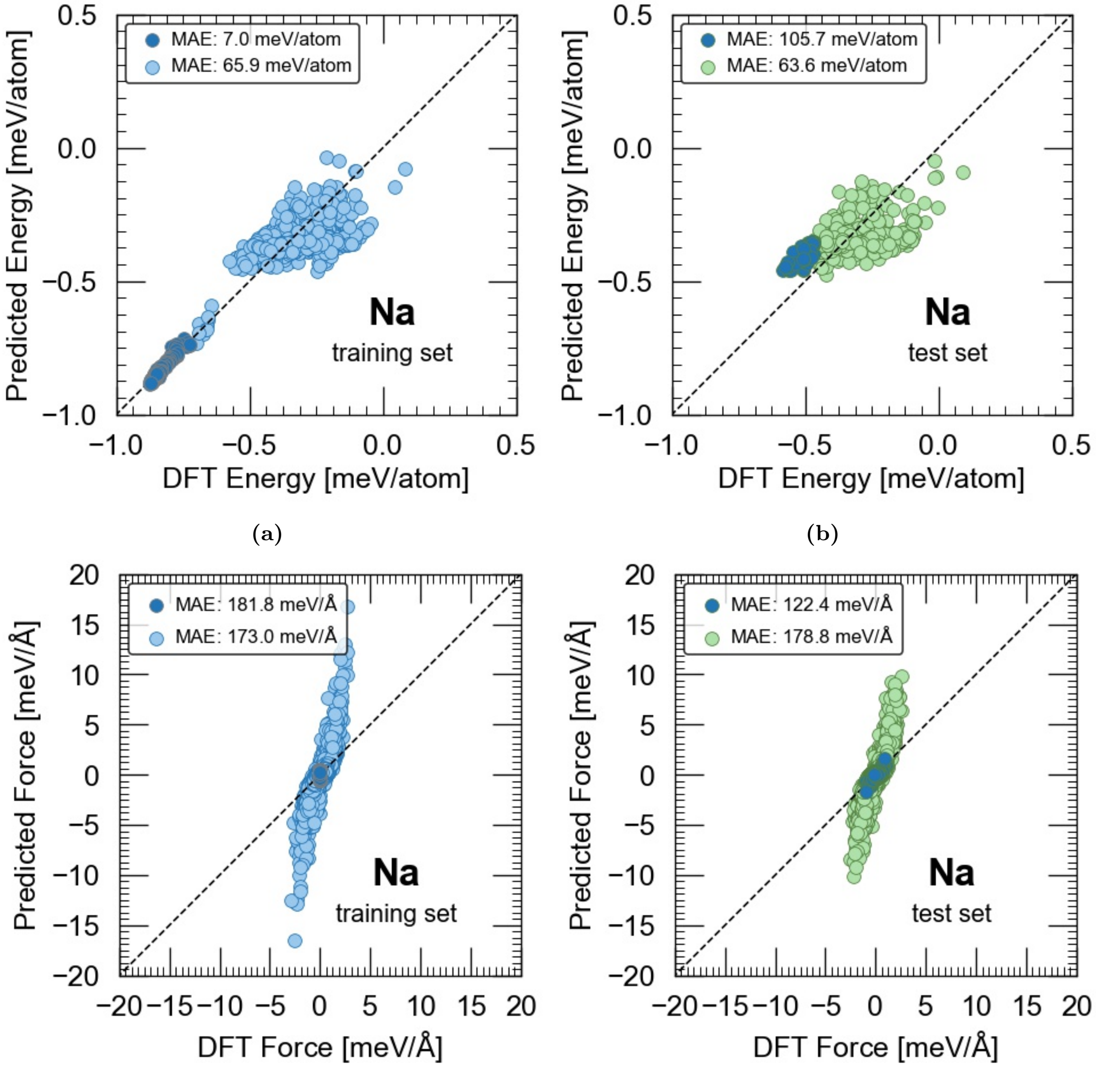

(a) (b) (c) (d)

Supplementary Figure 124: Energy and force correlation plots for Na nanoclusters

9.29 Nb

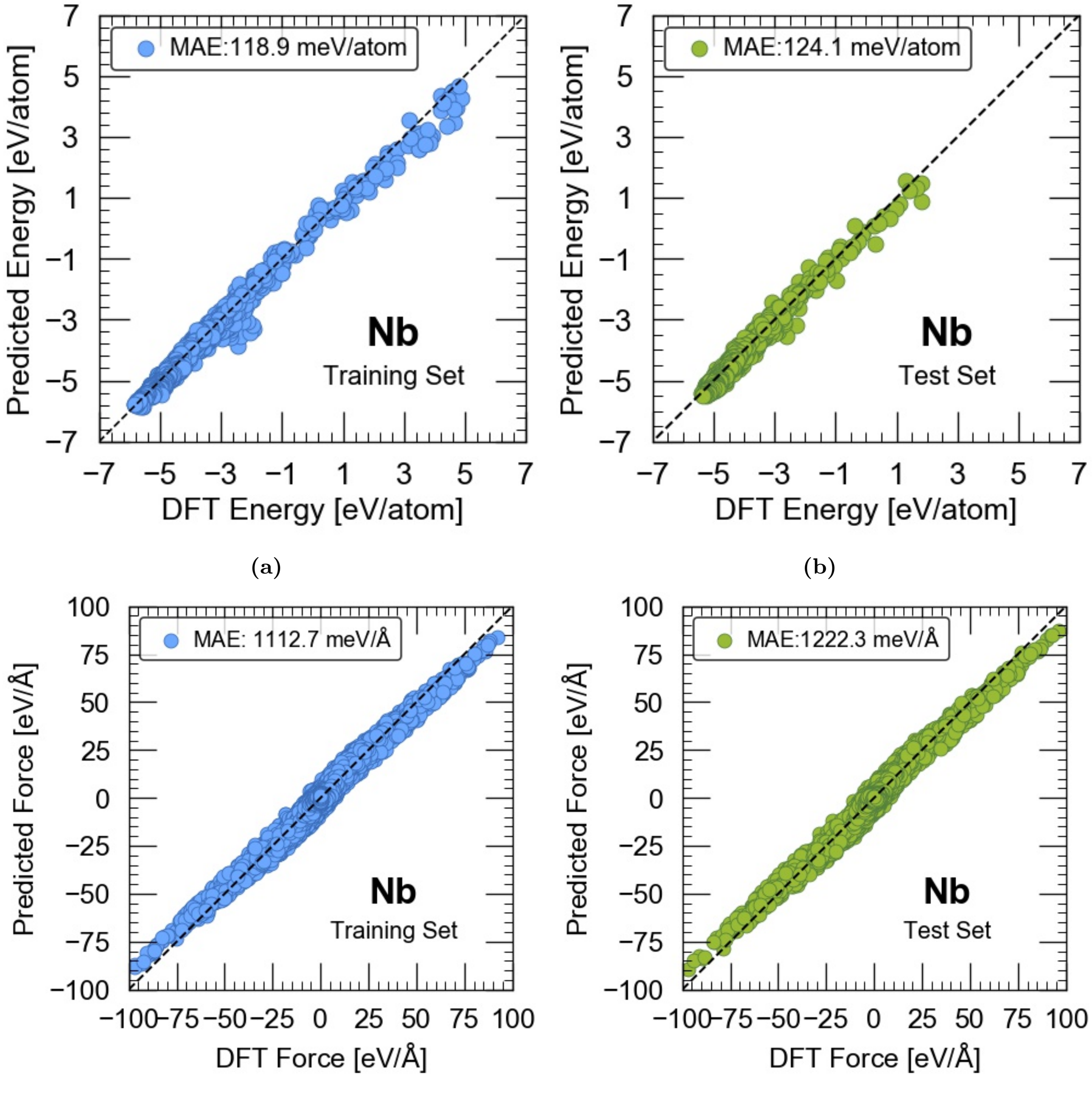

(a) (b) (c) (d)

Supplementary Figure 125: Energy and force correlation plots for Nb nanoclusters

9.30 Ni

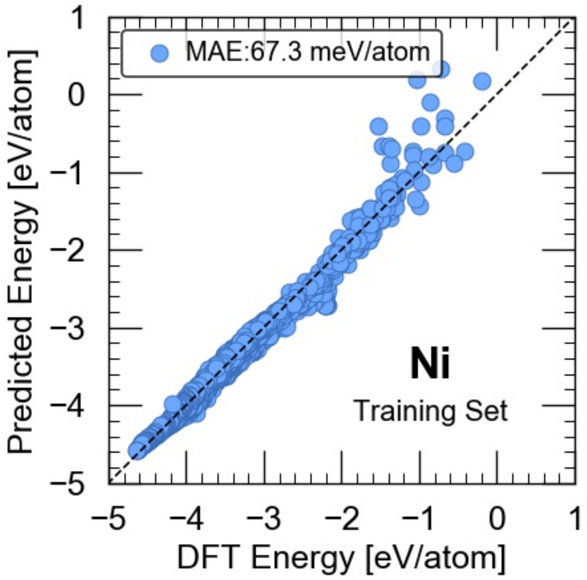

(a)

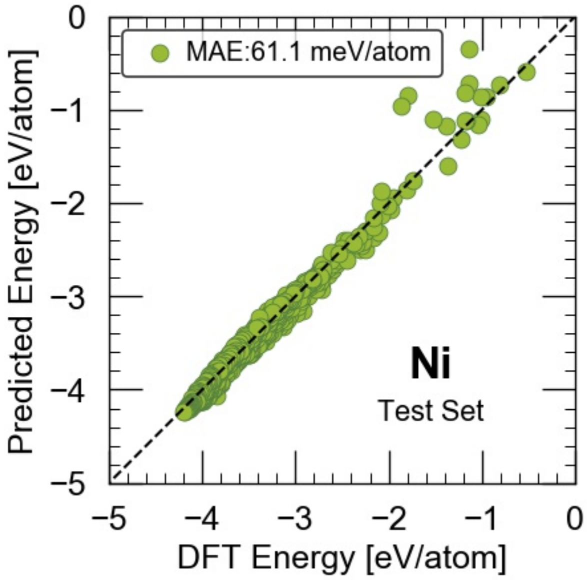

(b)

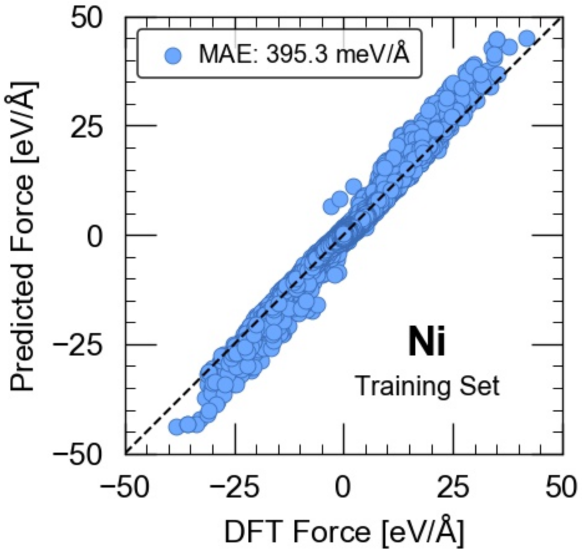

(c)

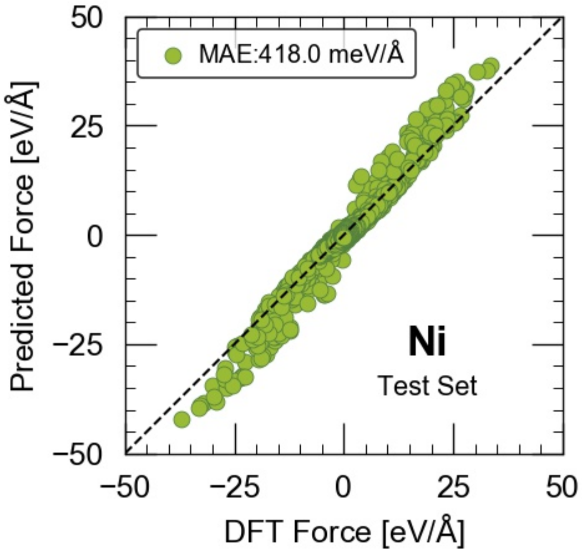

(d)

Supplementary Figure 126: Energy and force correlation plots for Ni nanoclusters

9.31 Os

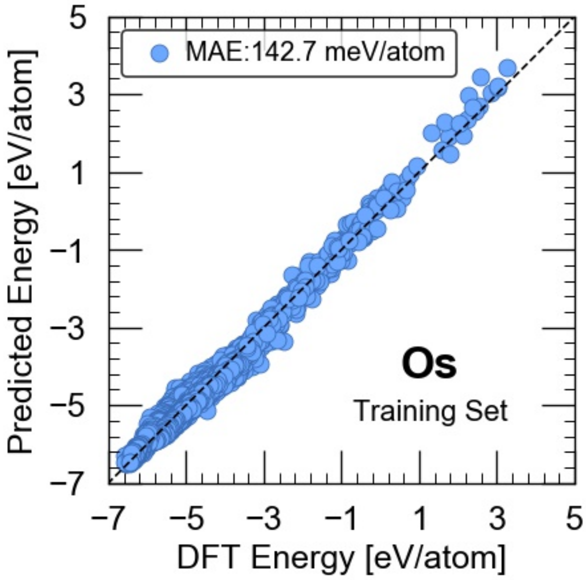

(a)

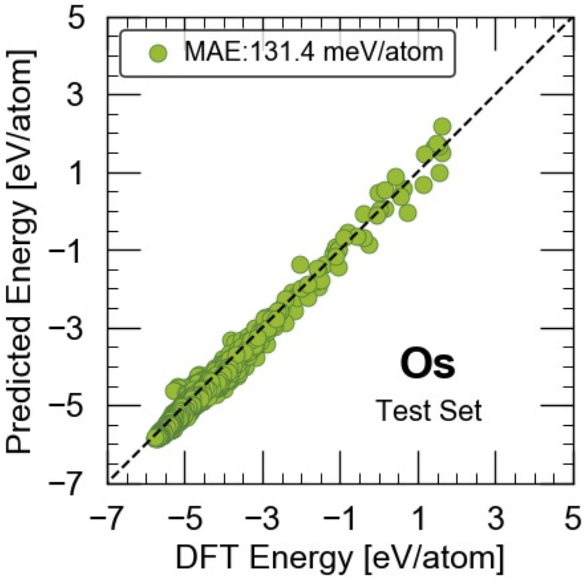

(b)

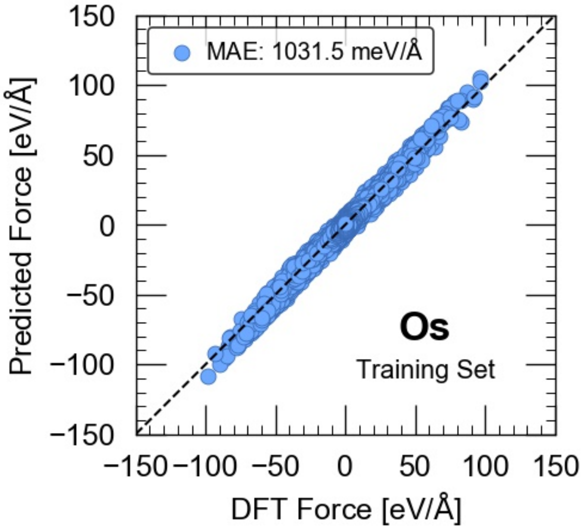

(c)

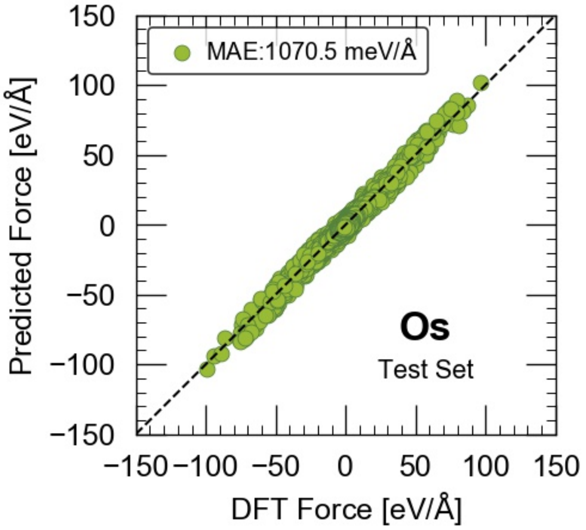

(d)

Supplementary Figure 127: Energy and force correlation plots for Os nanoclusters

9.32 P

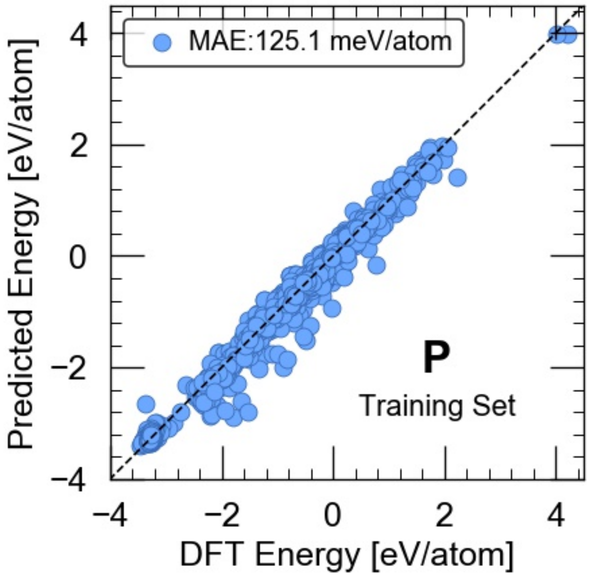

(a)

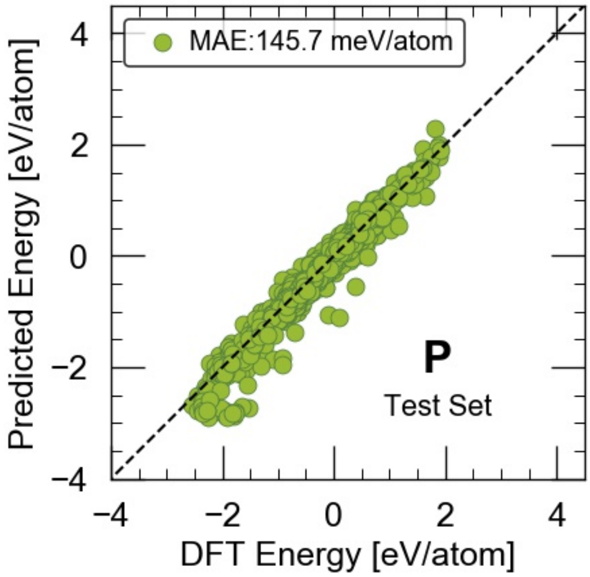

(b)

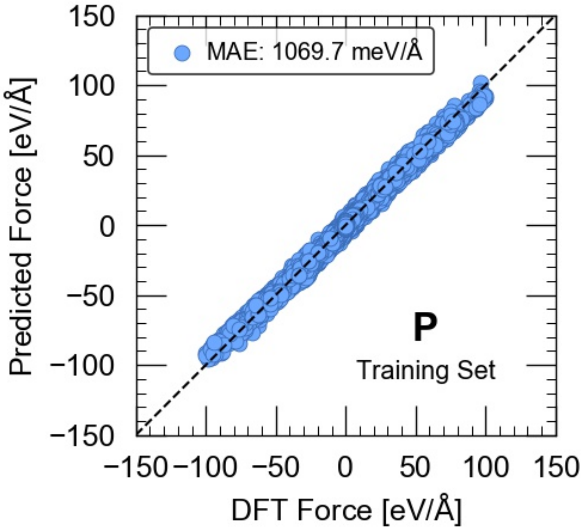

(c)

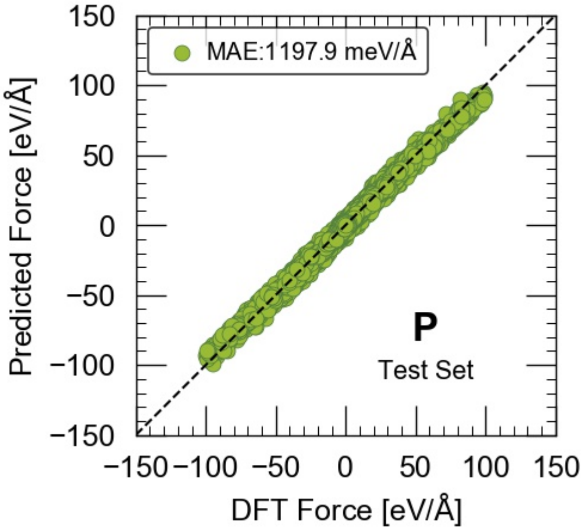

(d)

Supplementary Figure 128: Energy and force correlation plots for P nanoclusters

9.33 Pb

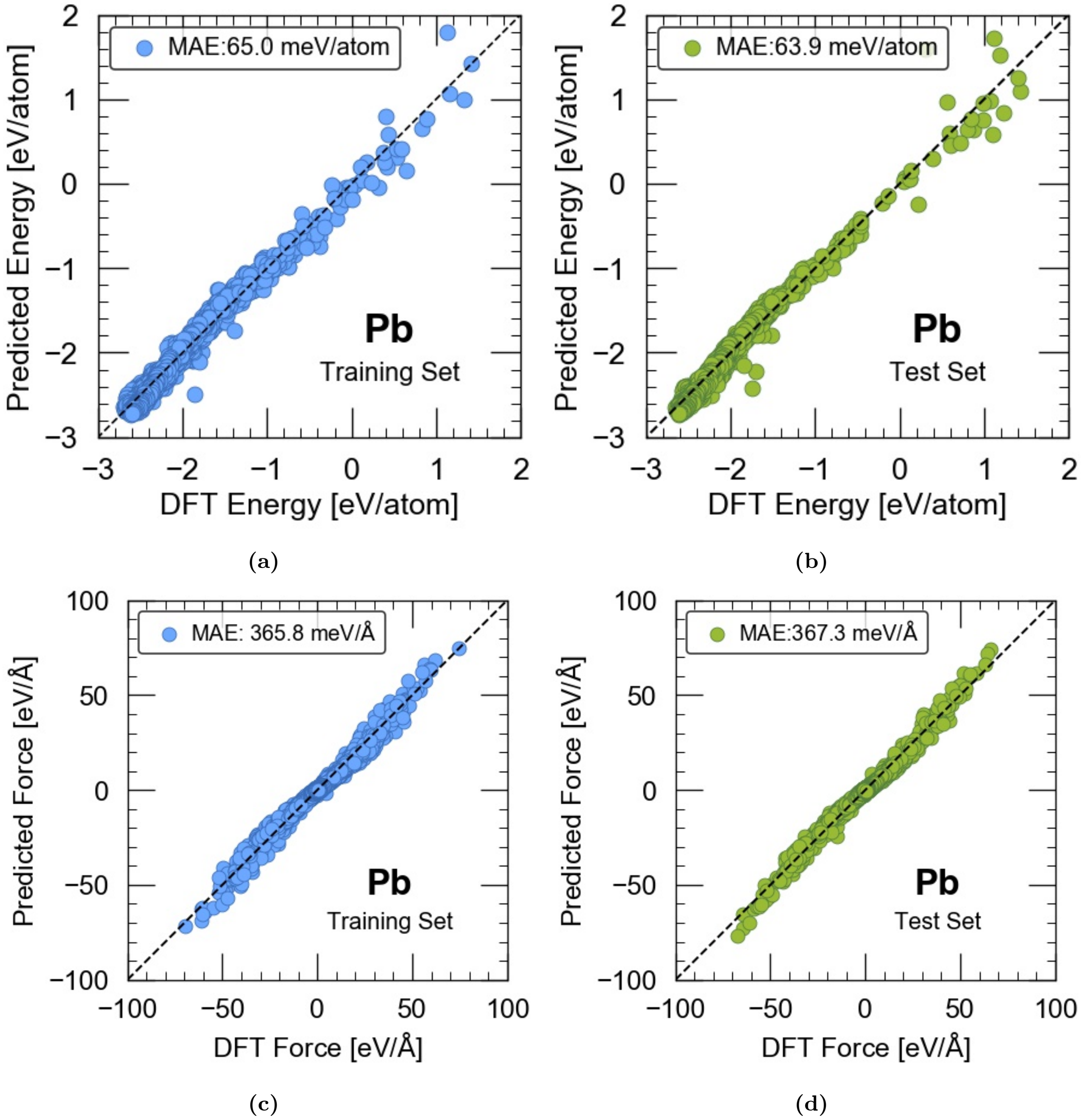

Supplementary Figure 129: Energy and force correlation plots for Pb nanoclusters

9.34 Pd

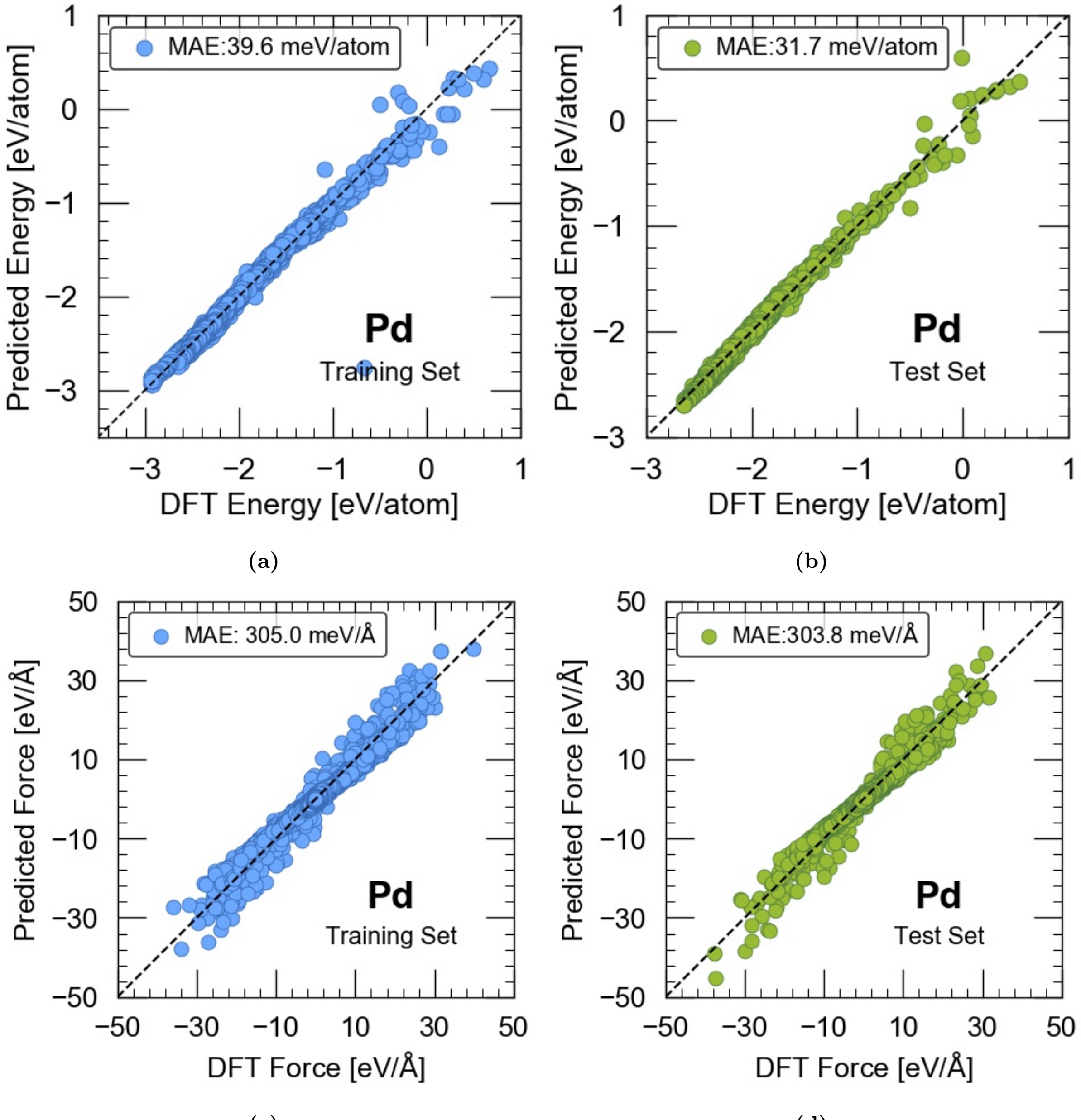

(a) (b) (c) (d)

Supplementary Figure 130: Energy and force correlation plots for Pd nanoclusters

9.35 Pt

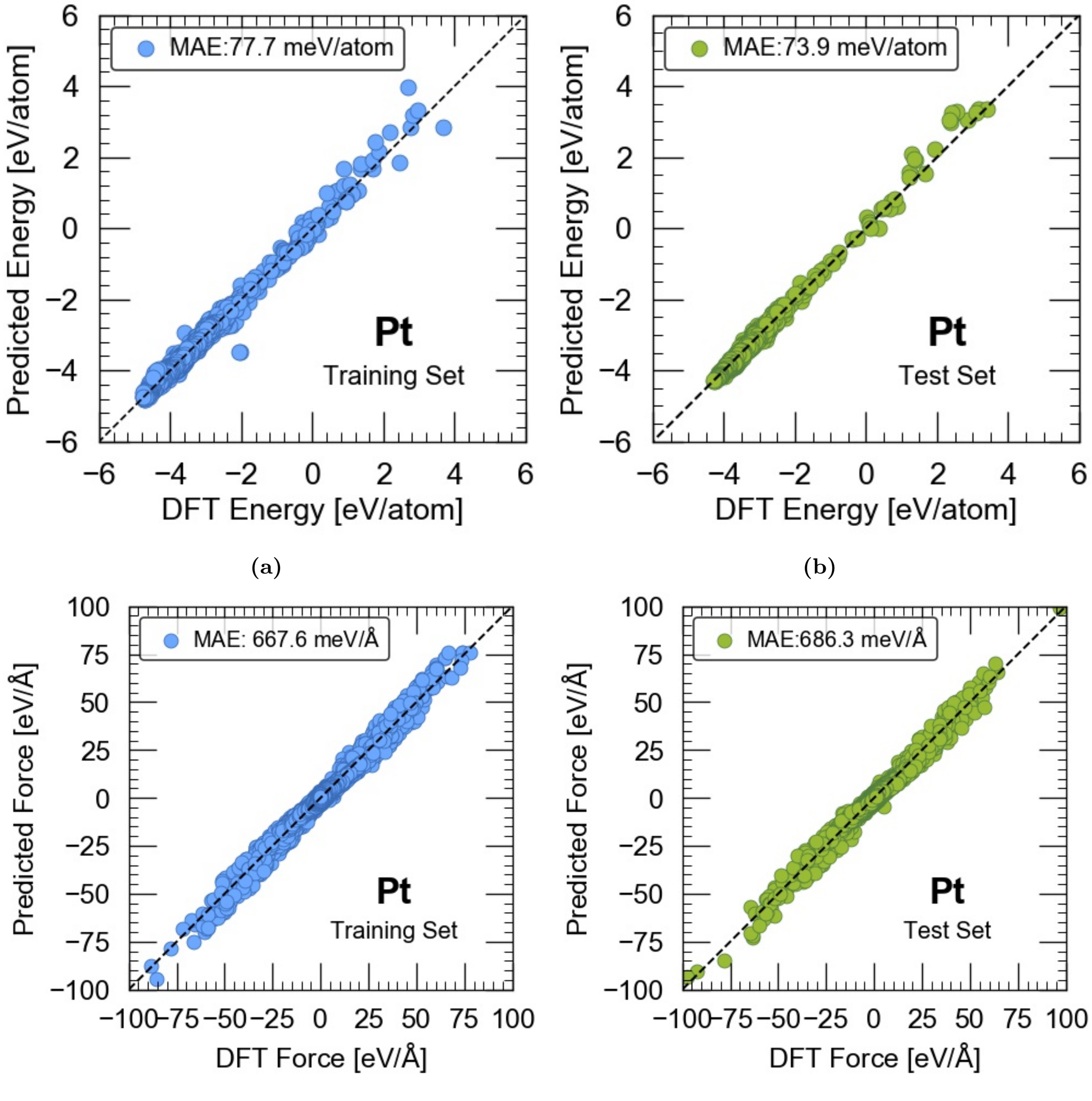

(a) (b) (c) (d)

Supplementary Figure 131: Energy and force correlation plots for Pt nanoclusters

9.36 Rb

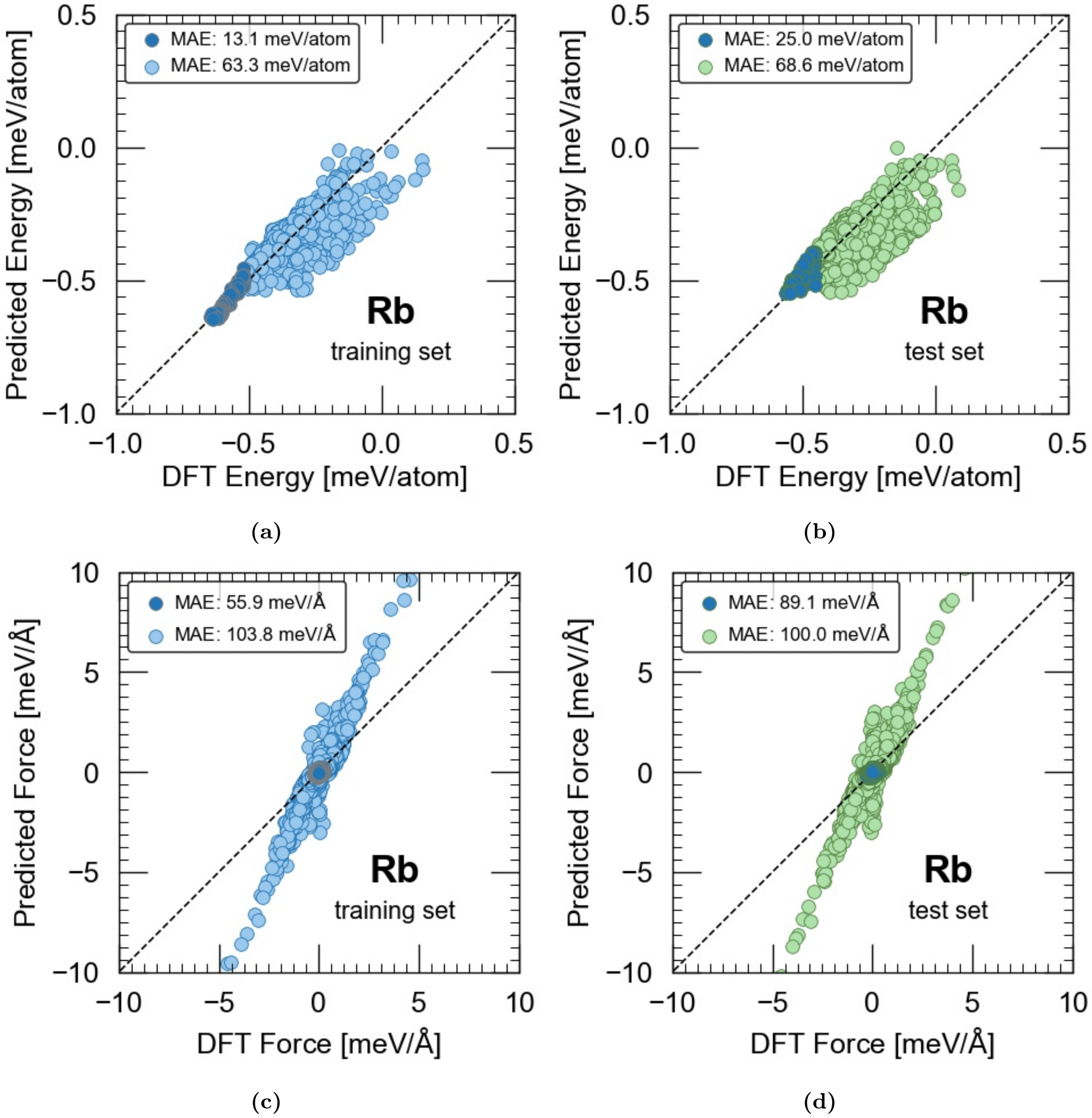

(a) (b) (c) (d)

Supplementary Figure 132: Energy and force correlation plots for Rb nanoclusters

9.37 Re

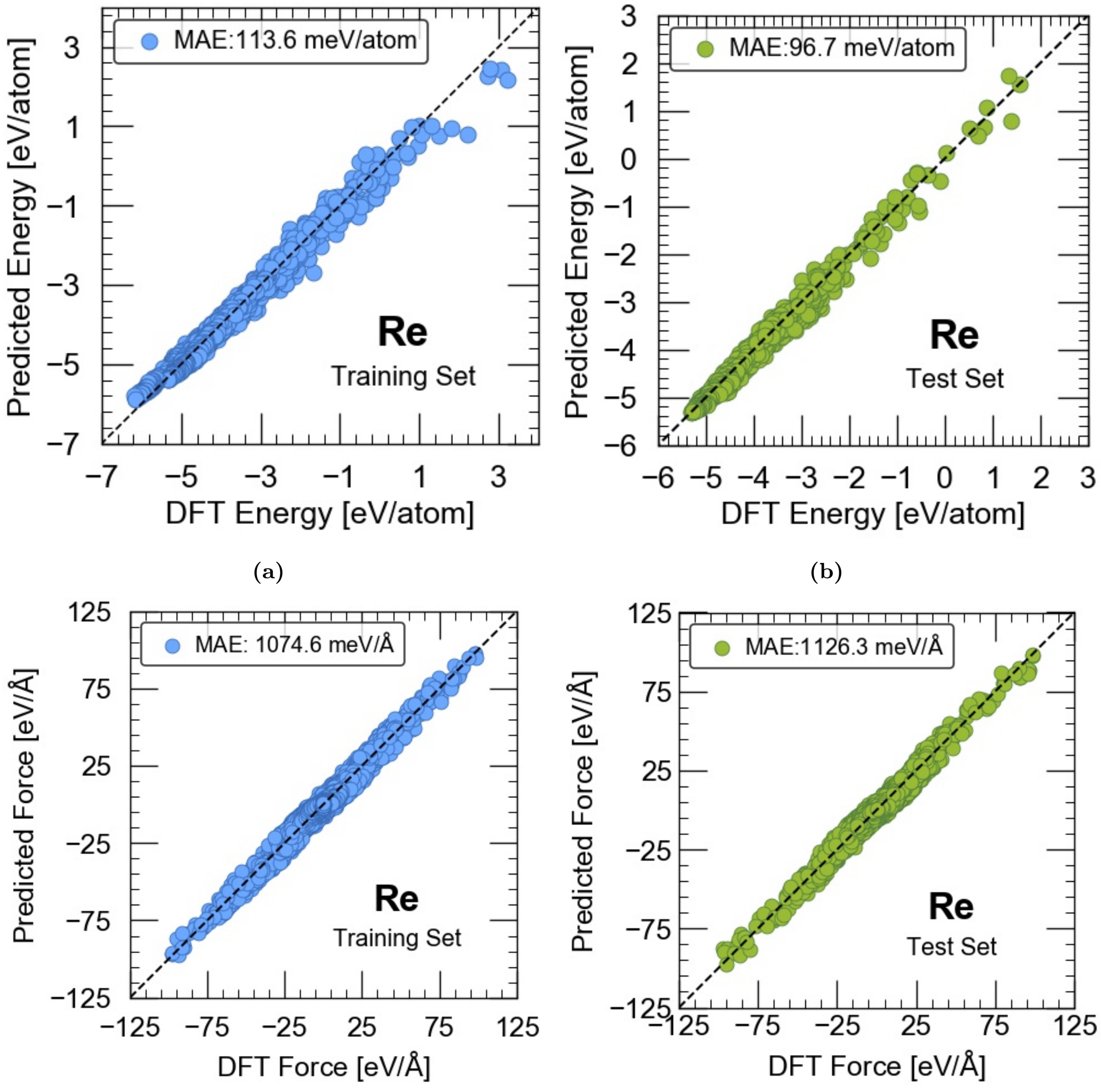

(a) **(b)**

(c) **(d)**

Supplementary Figure 133: Energy and force correlation plots for Re nanoclusters

9.38 Rh

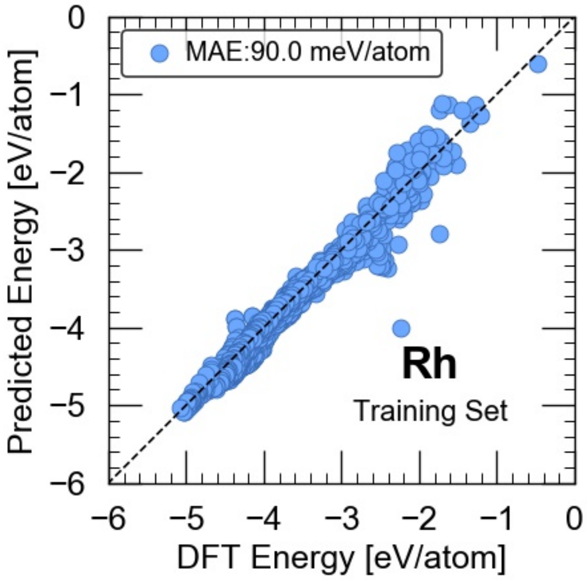

(a)

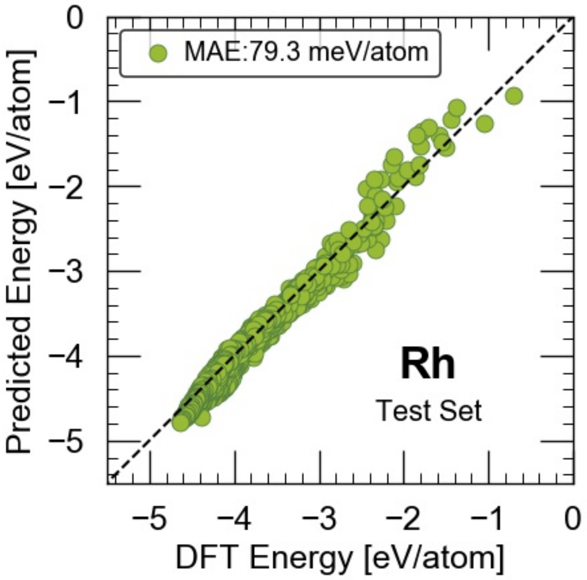

(b)

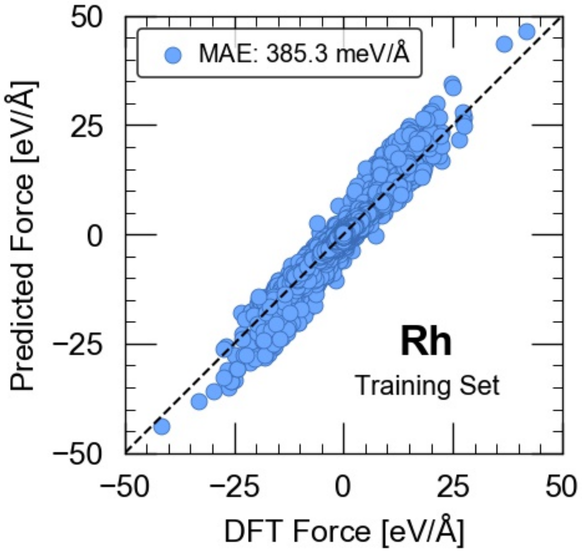

(c)

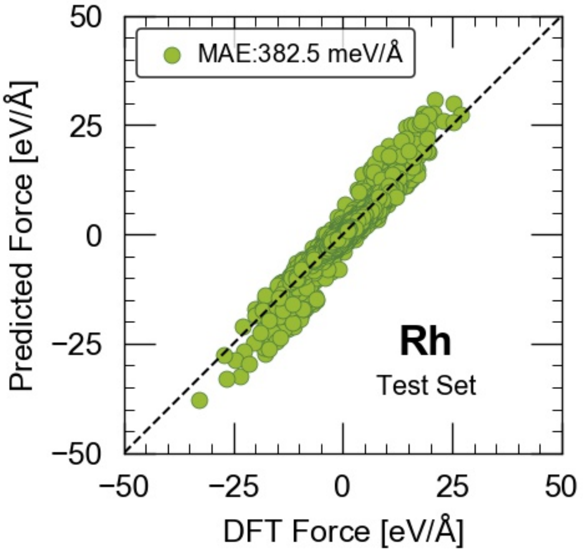

(d)

Supplementary Figure 134: Energy and force correlation plots for Rh nanoclusters

9.39 Ru

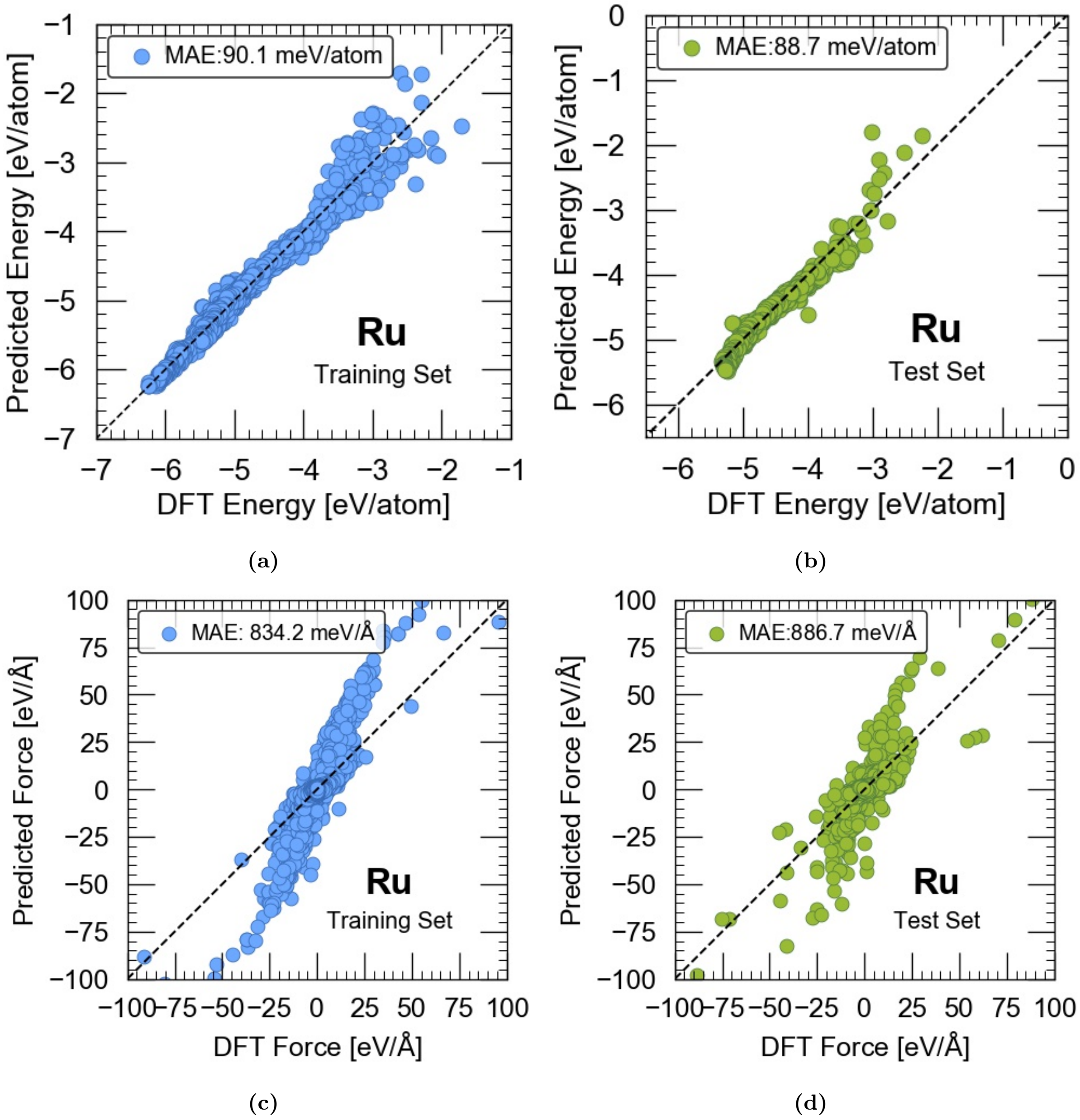

(a) **(b)** **(c)** **(d)**

Supplementary Figure 135: Energy and force correlation plots for Ru nanoclusters

9.40 S

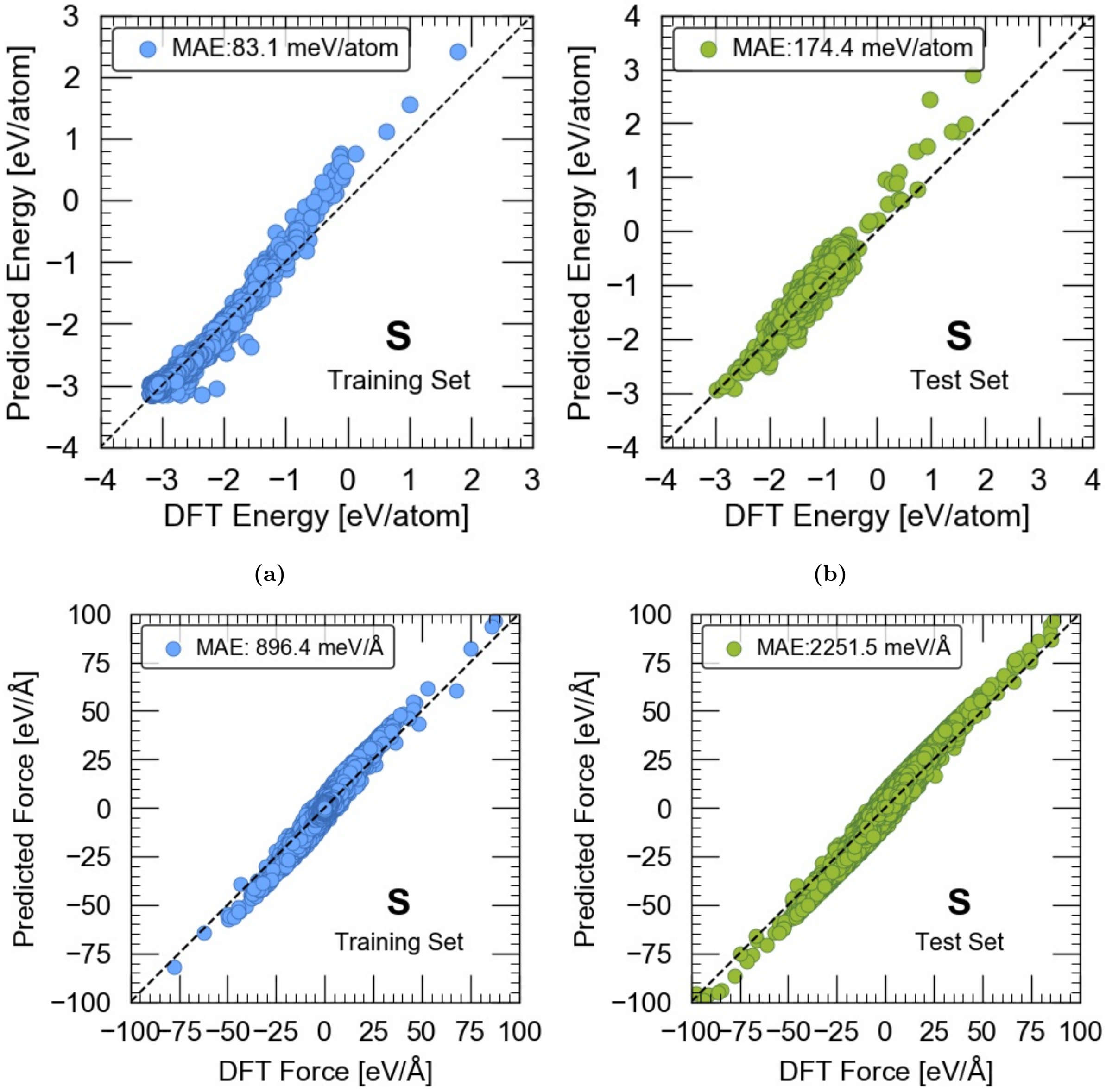

(a) **(b)** **(c)** **(d)**

Supplementary Figure 136: Energy and force correlation plots for S nanoclusters

9.41 Sb

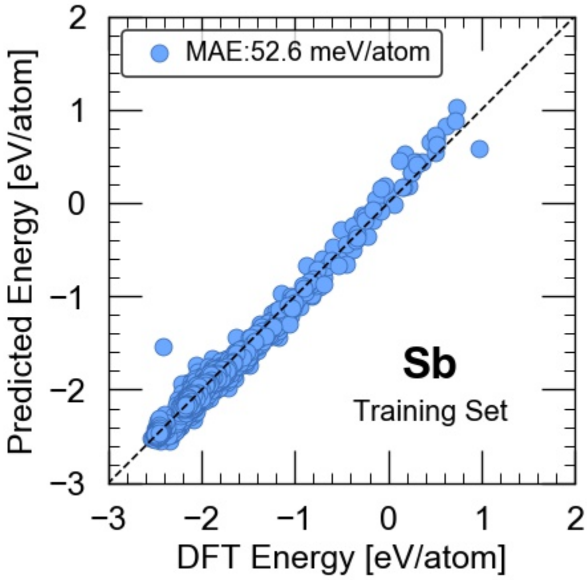

(a)

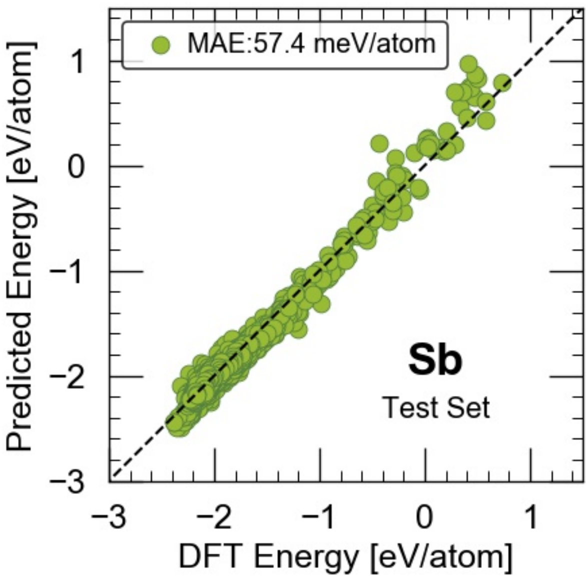

(b)

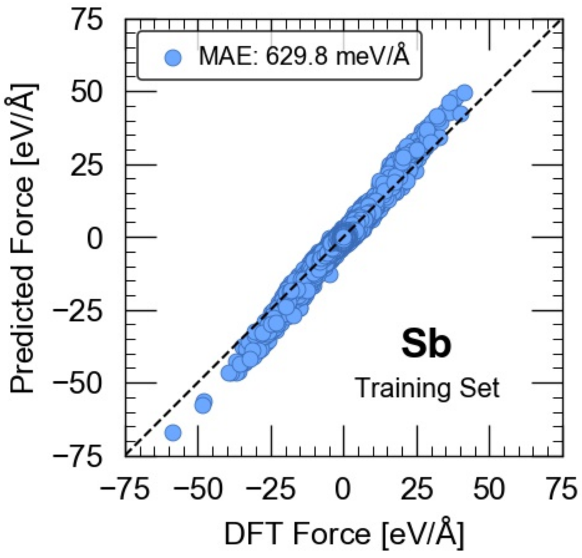

(c)

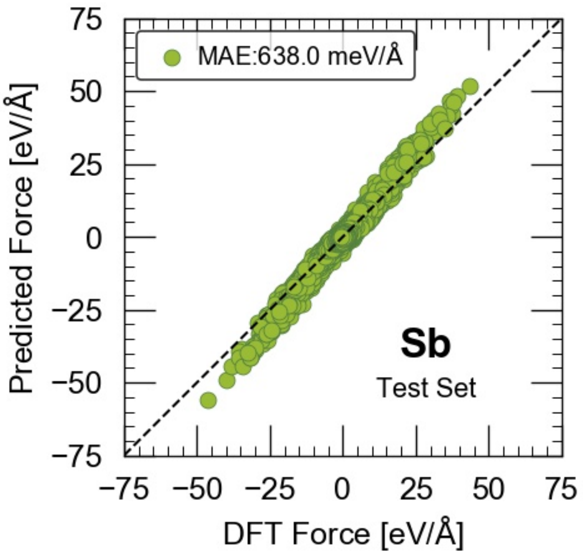

(d)

Supplementary Figure 137: Energy and force correlation plots for Sb nanoclusters

9.42 Sc

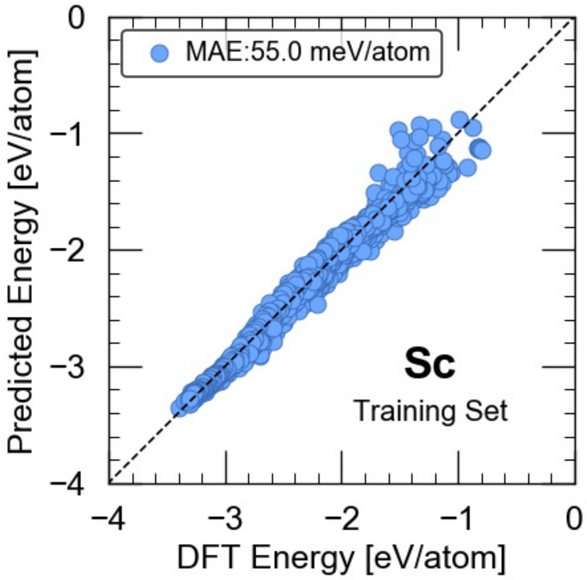

(a)

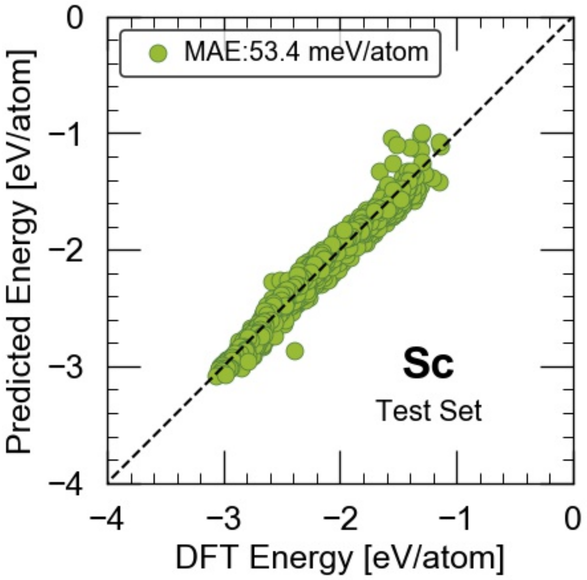

(b)

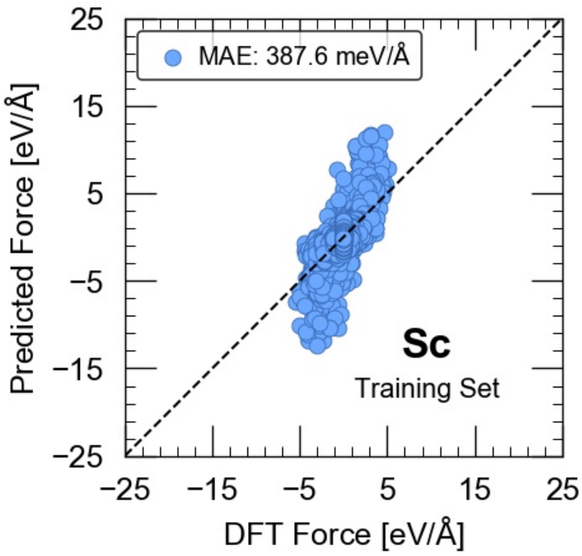

(c)

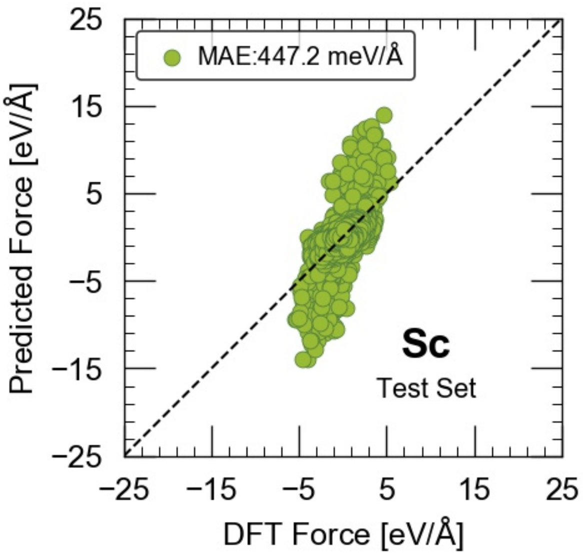

(d)

Supplementary Figure 138: Energy and force correlation plots for Sc nanoclusters

9.43 Se

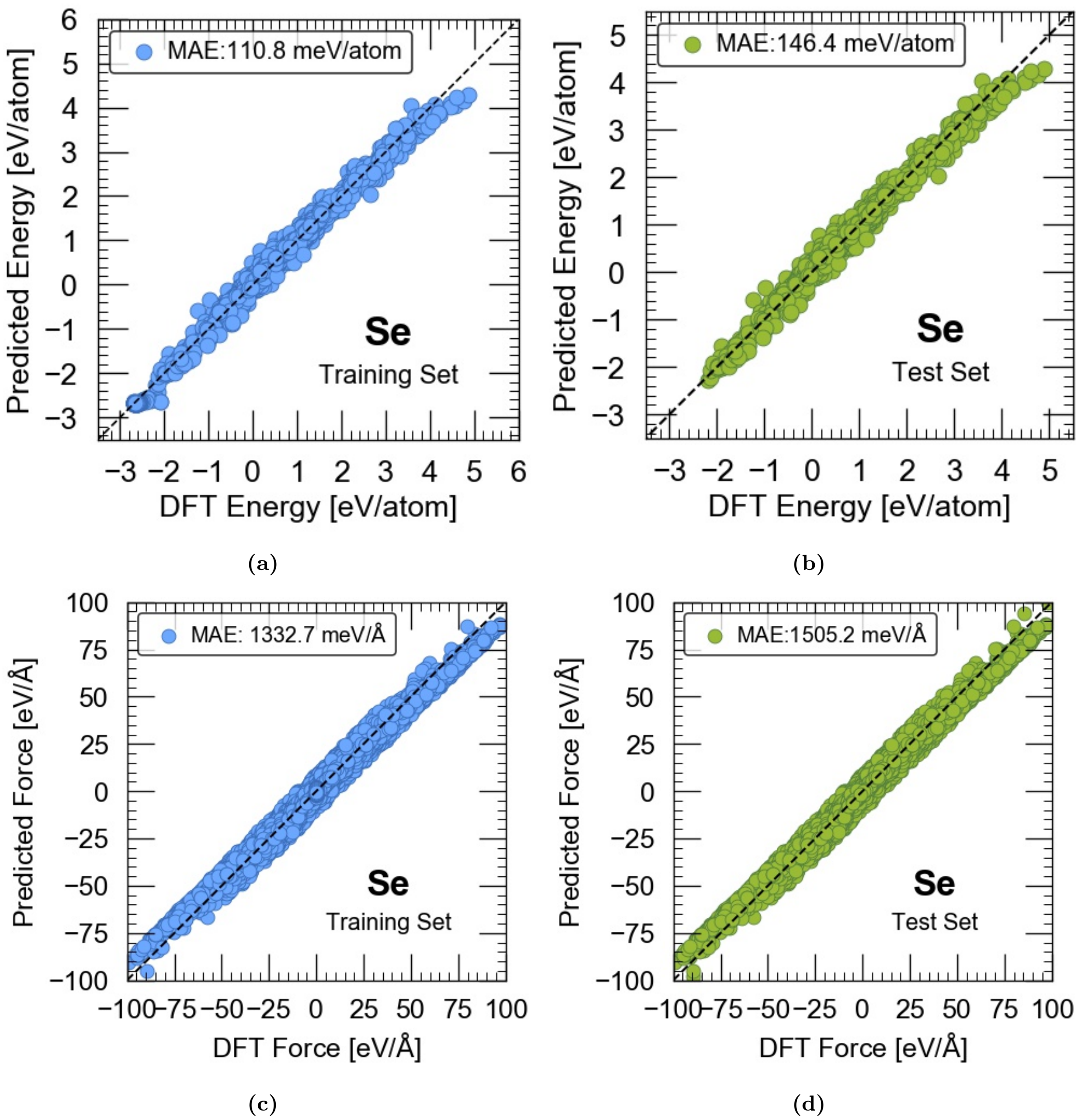

Supplementary Figure 139: Energy and force correlation plots for Se nanoclusters

9.44 Si

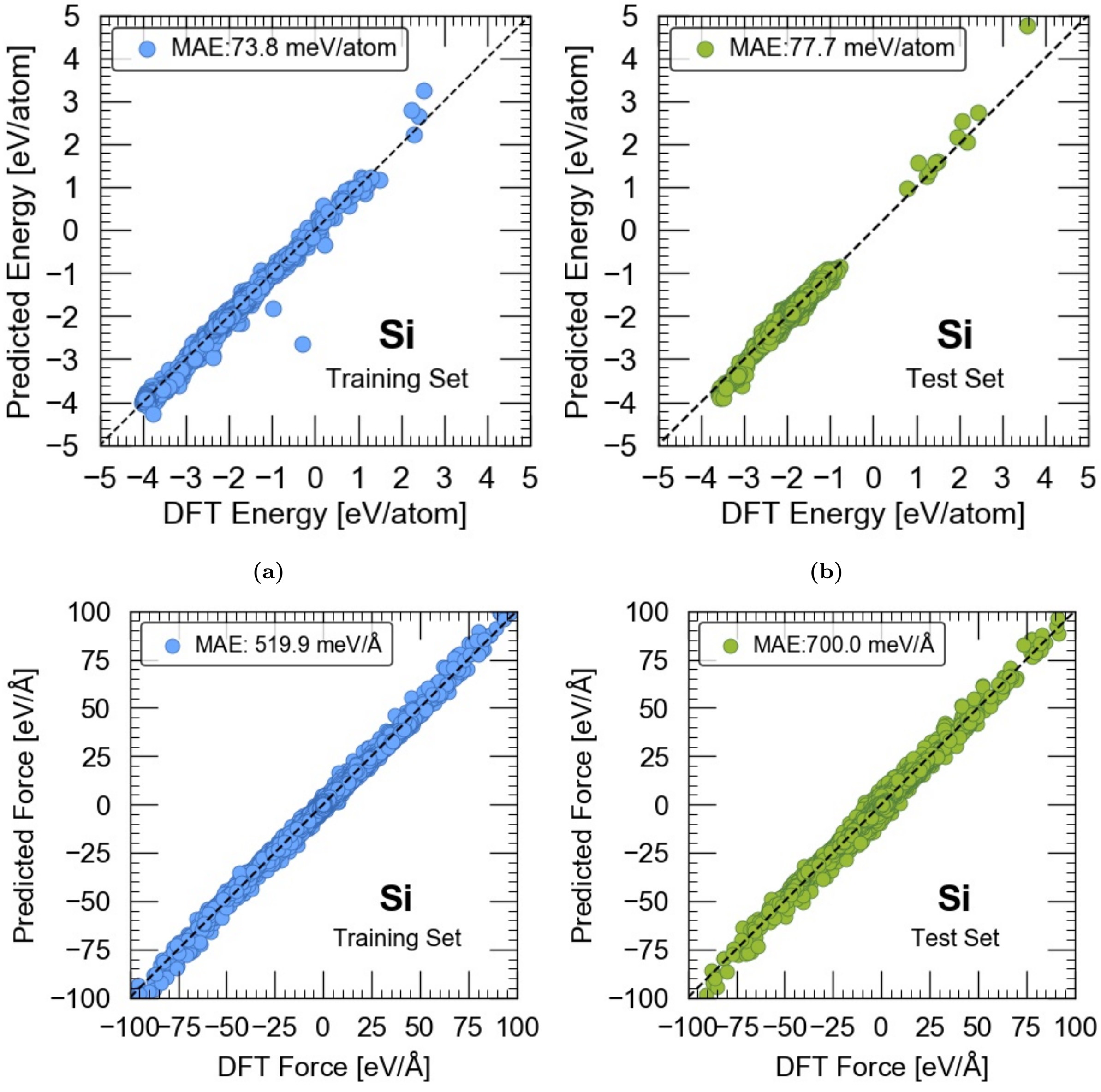

(c) (d)

Supplementary Figure 140: Energy and force correlation plots for Si nanoclusters

9.45 Sn

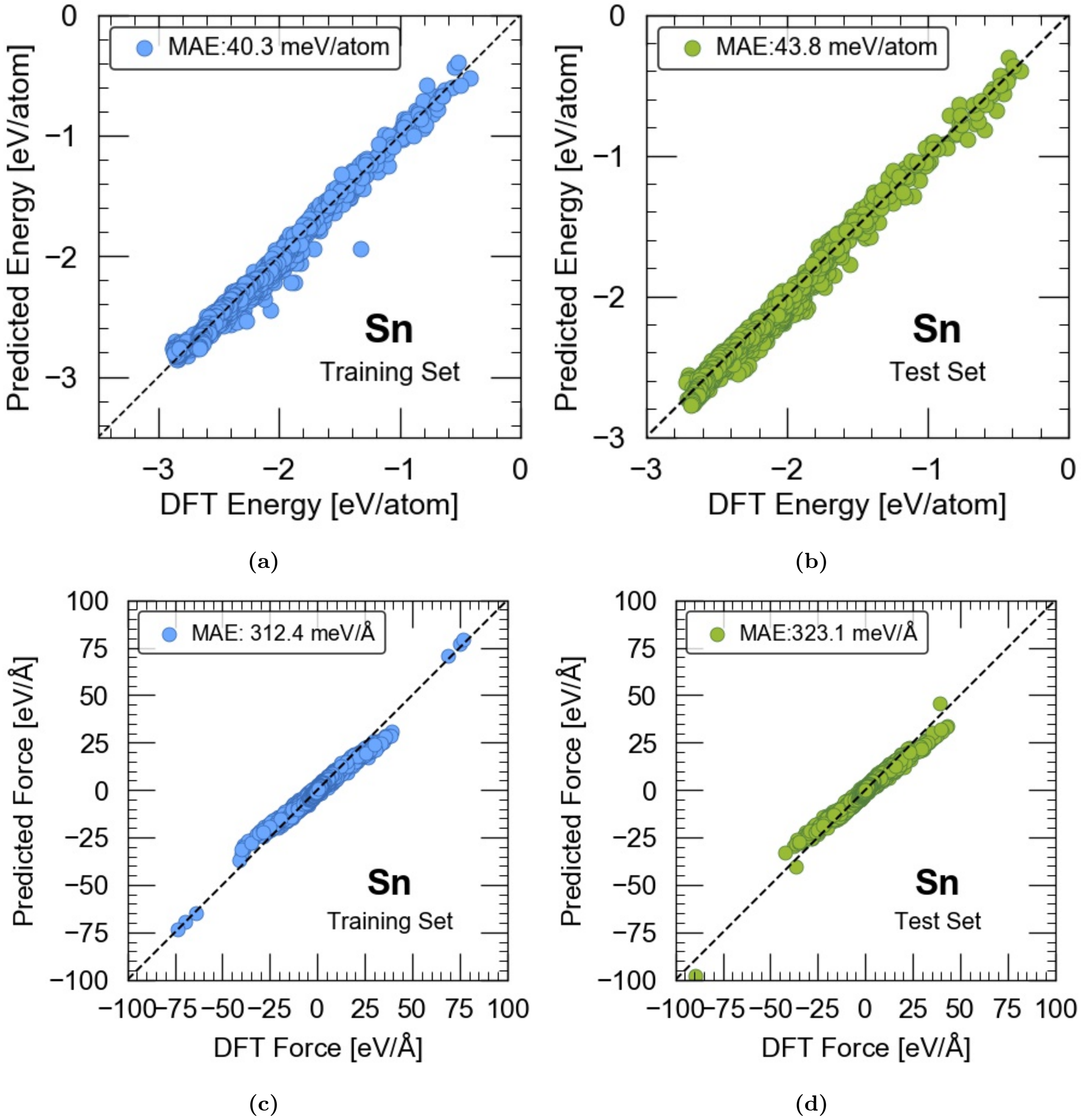

Supplementary Figure 141: Energy and force correlation plots for Sn nanoclusters

9.46 Sr

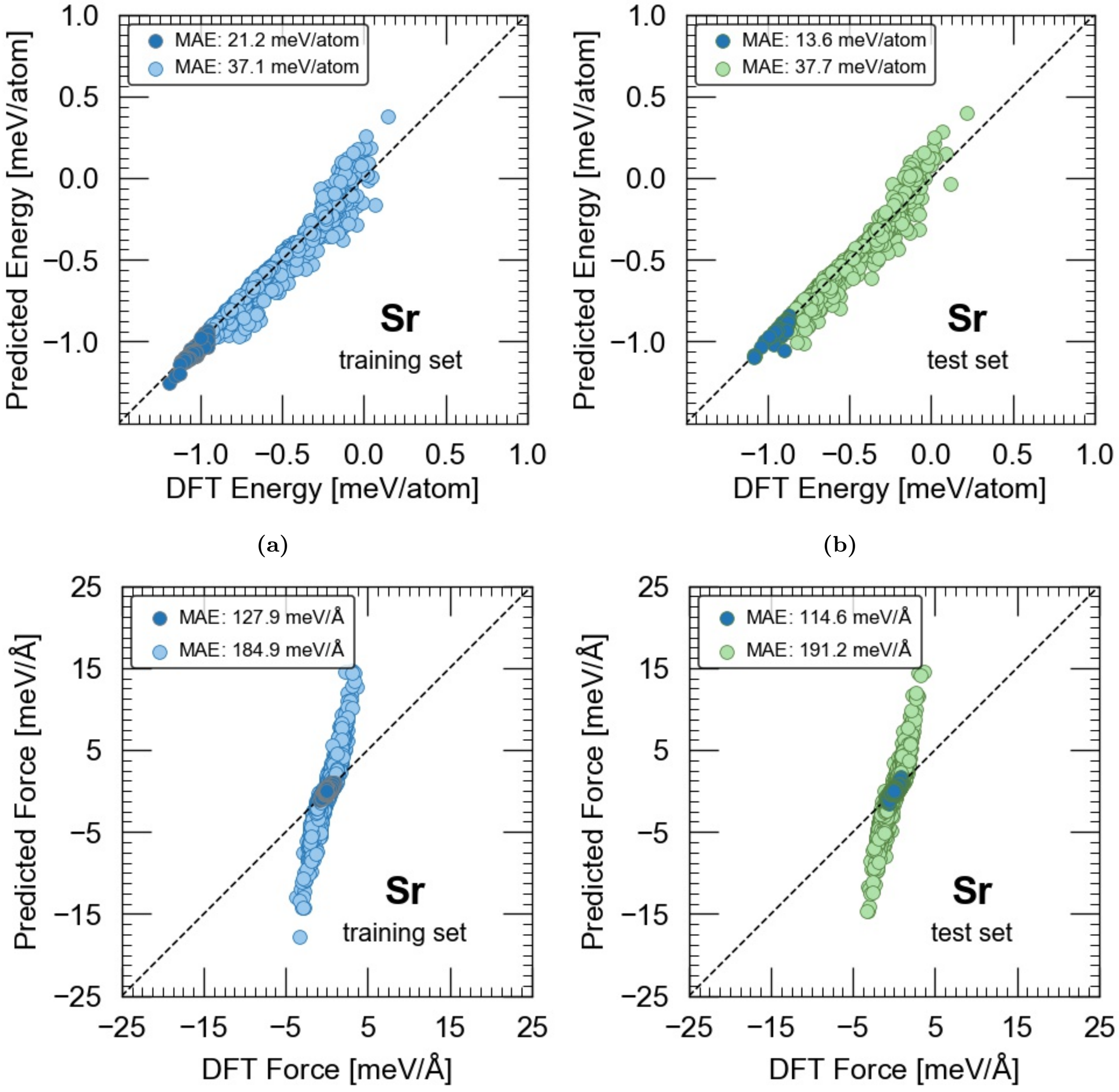

(a) (b) (c) (d)

Supplementary Figure 142: Energy and force correlation plots for Sr nanoclusters

9.47 Ta

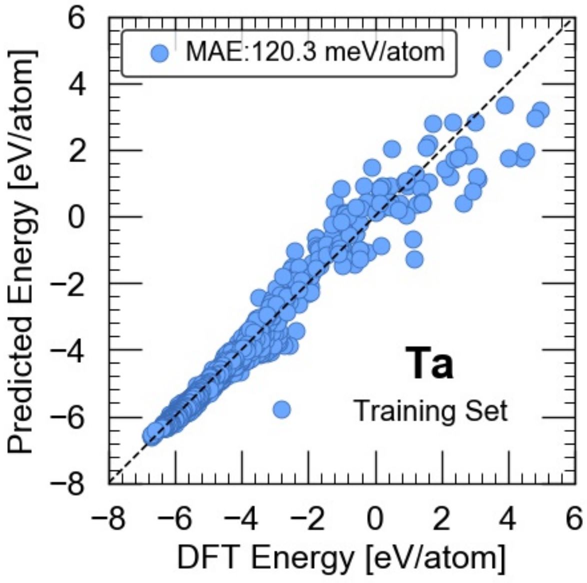

(a)

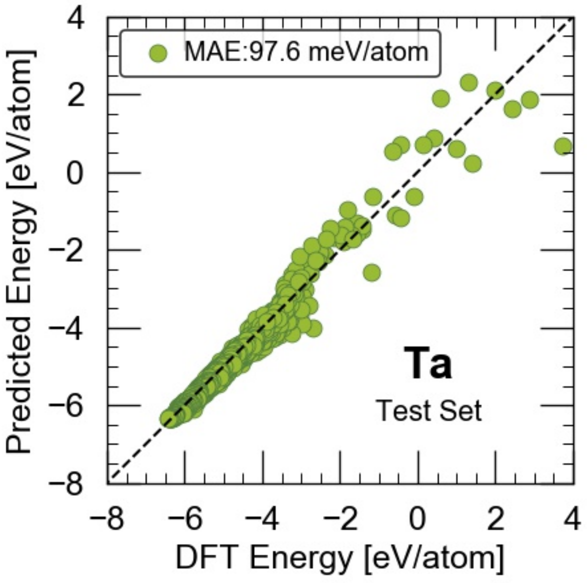

(b)

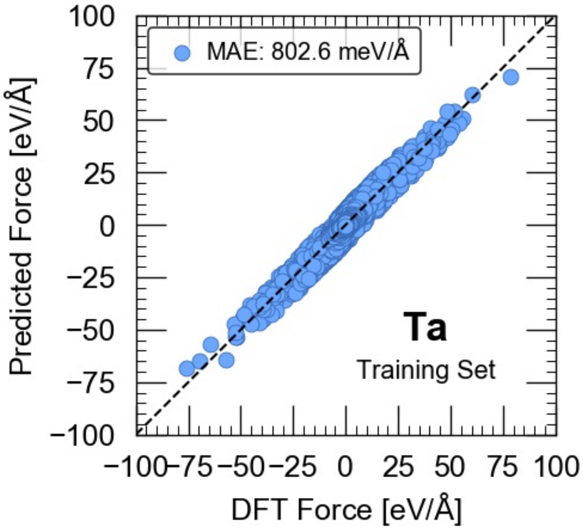

(c)

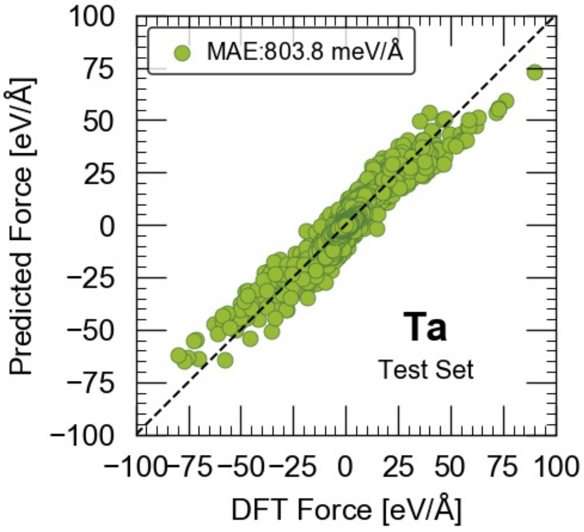

(d)

Supplementary Figure 143: Energy and force correlation plots for Ta nanoclusters

9.48 Te

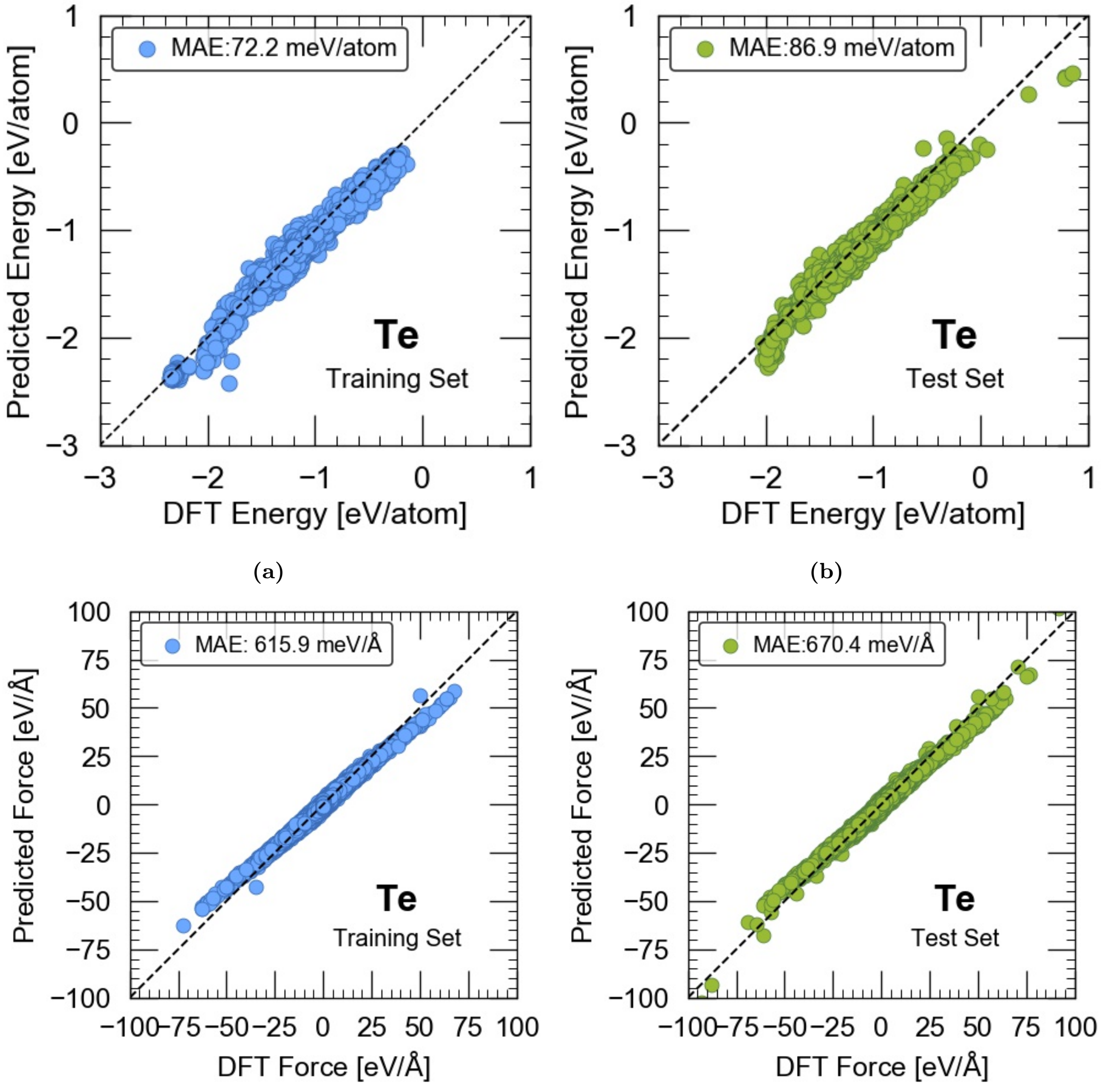

(a) **(b)** **(c)** **(d)**

Supplementary Figure 144: Energy and force correlation plots for Te nanoclusters

9.49 Ti

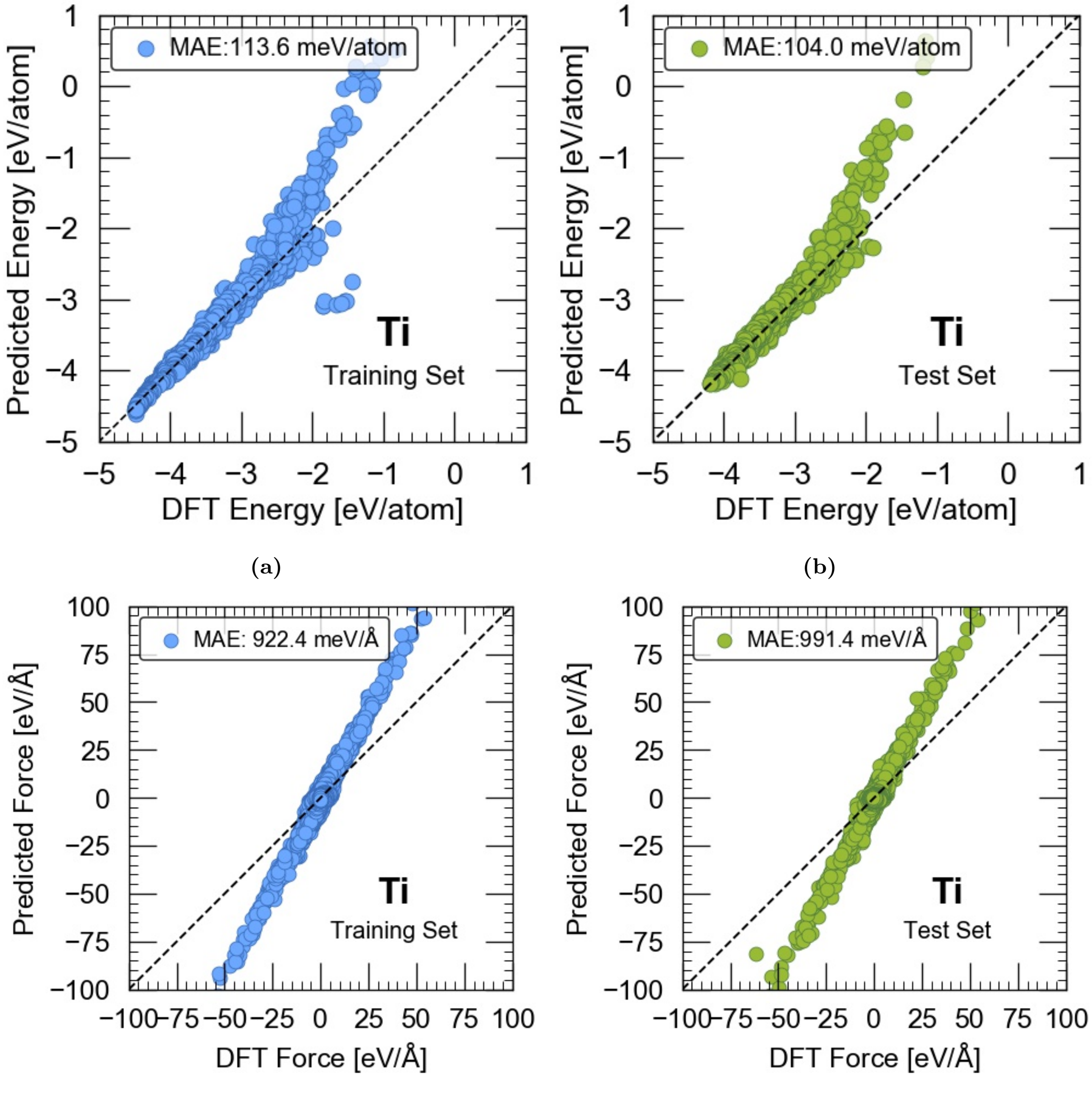

(a) (b) (c) (d)

Supplementary Figure 145: Energy and force correlation plots for Ti nanoclusters

9.50 Tl

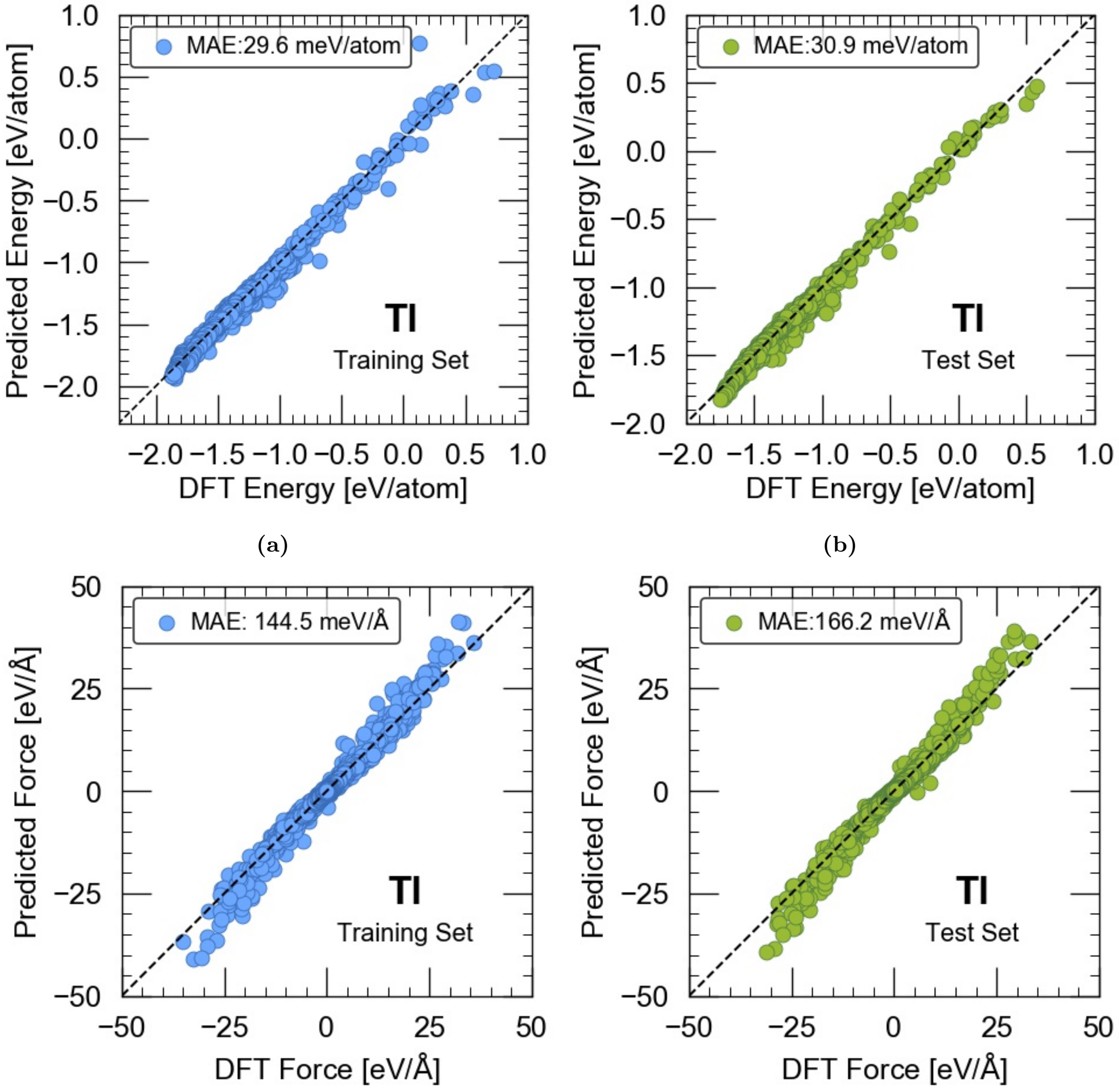

(a) (b) (c) (d)

Supplementary Figure 146: Energy and force correlation plots for Tl nanoclusters

9.51 V

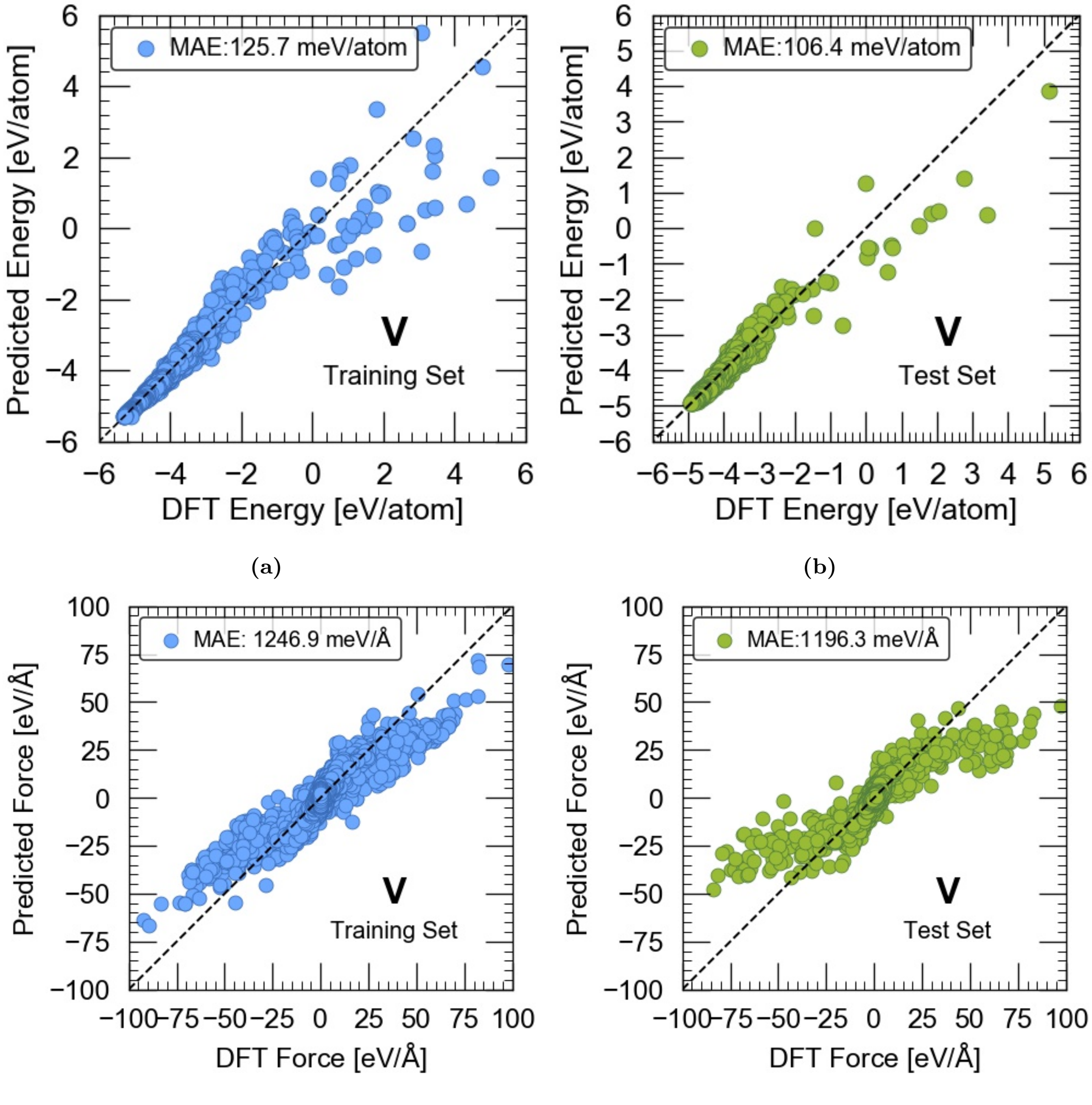

(a) (b) (c) (d)

Supplementary Figure 147: Energy and force correlation plots for V nanoclusters

9.52 W

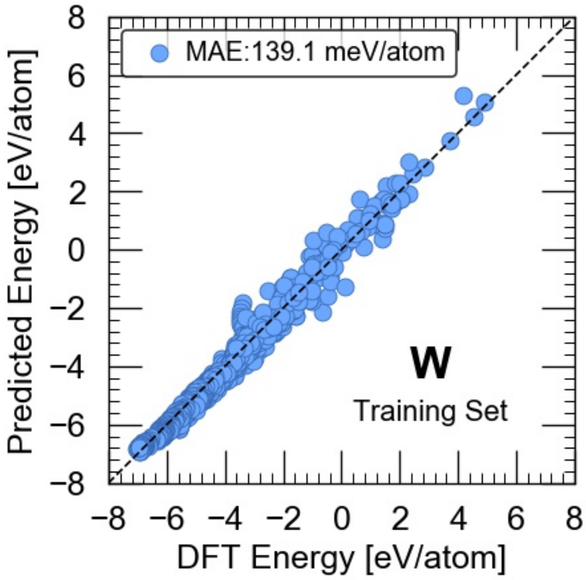

(a)

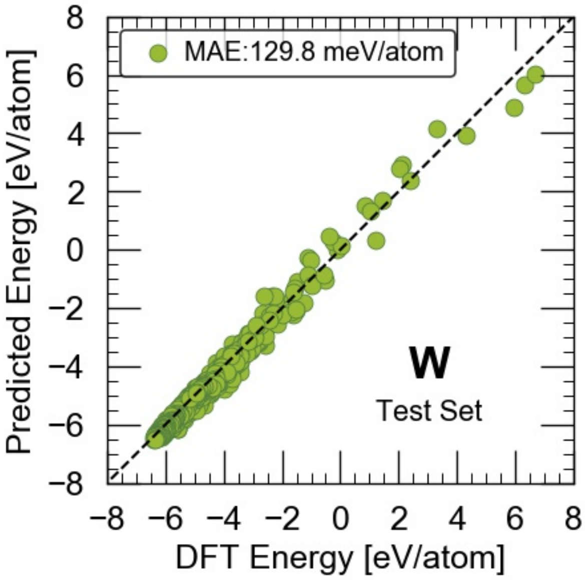

(b)

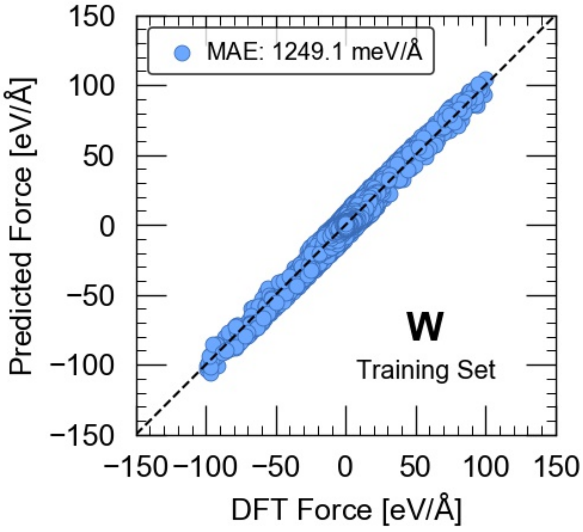

(c)

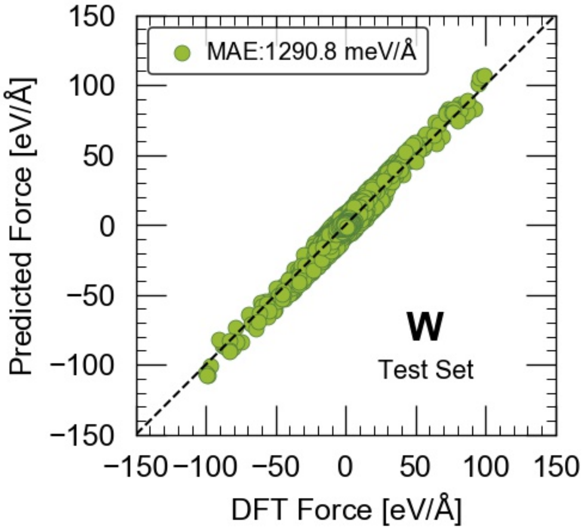

(d)

Supplementary Figure 148: Energy and force correlation plots for W nanoclusters

9.53 Y

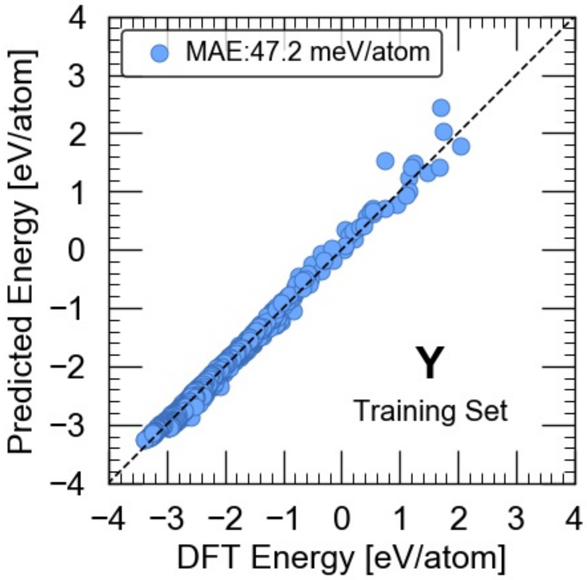

(a)

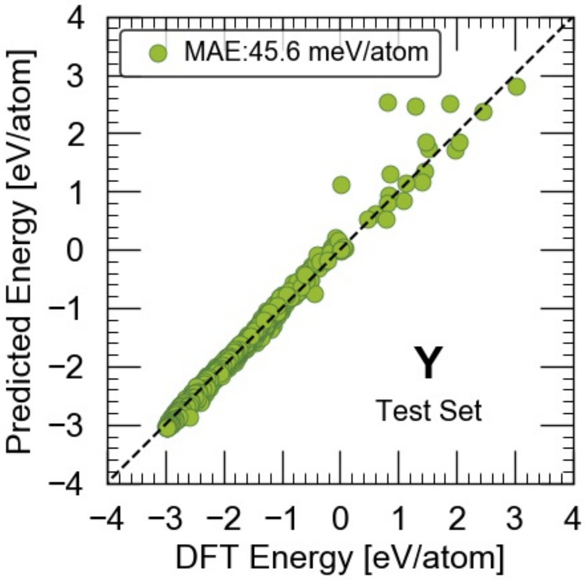

(b)

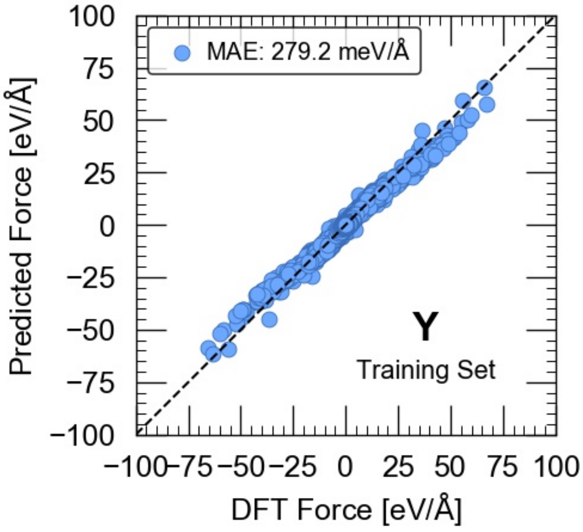

(c)

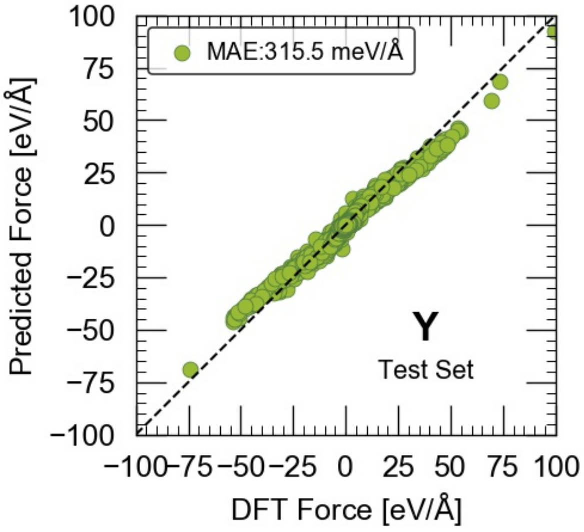

(d)

Supplementary Figure 149: Energy and force correlation plots for Y nanoclusters

9.54 Zn

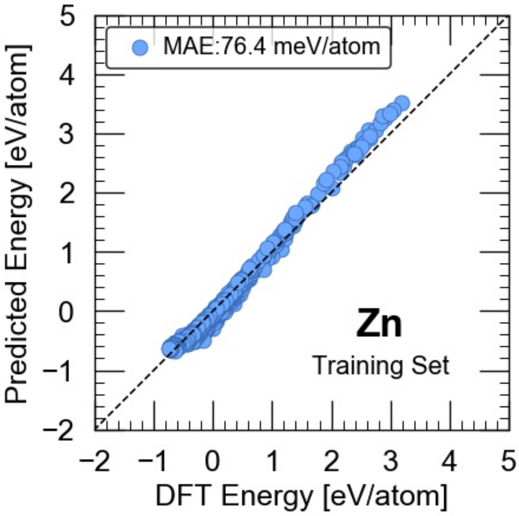

(a)

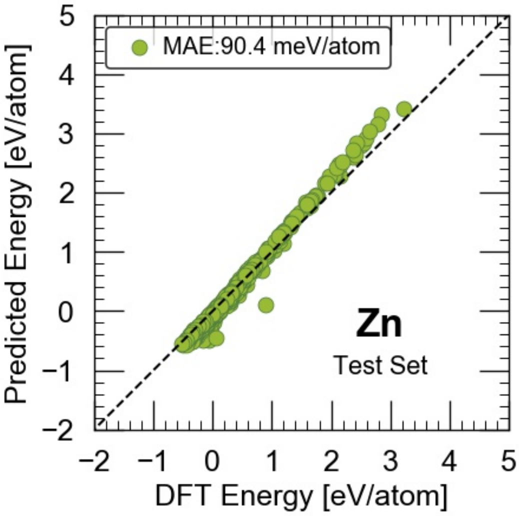

(b)

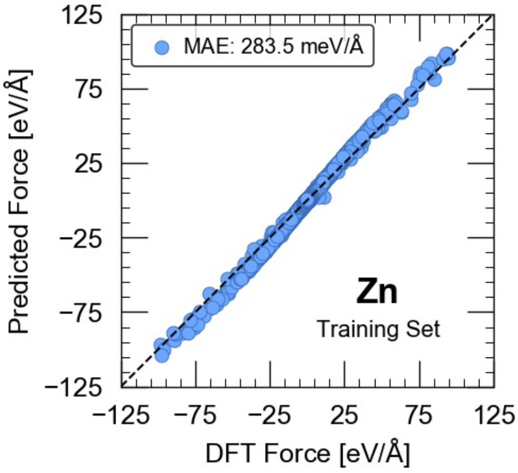

(c)

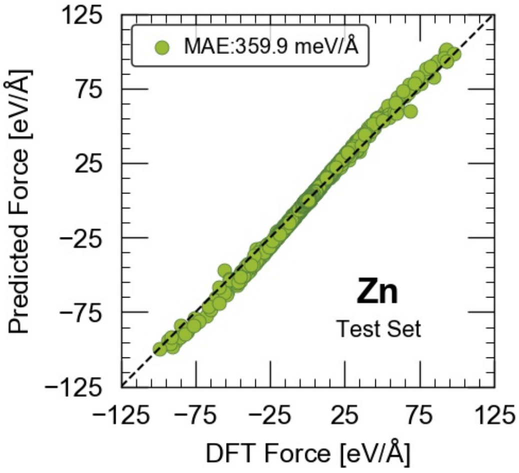

(d)

Supplementary Figure 150: Energy and force correlation plots for Zn nanoclusters

9.55 Zr

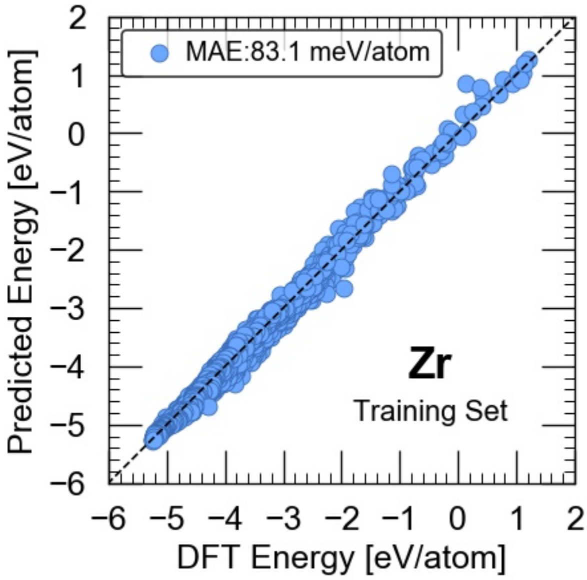

(a)

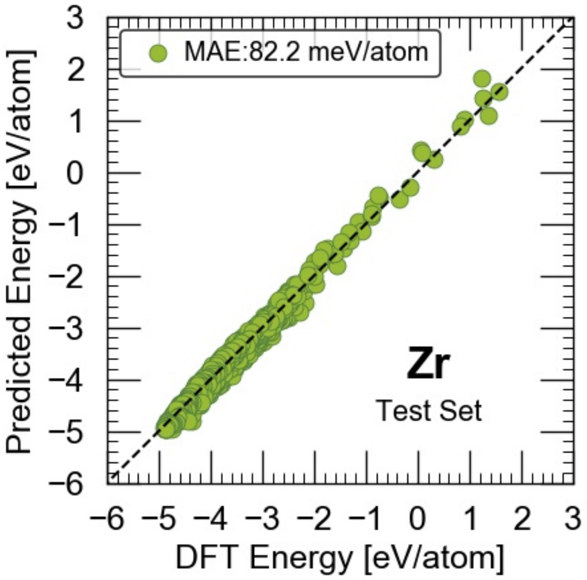

(b)

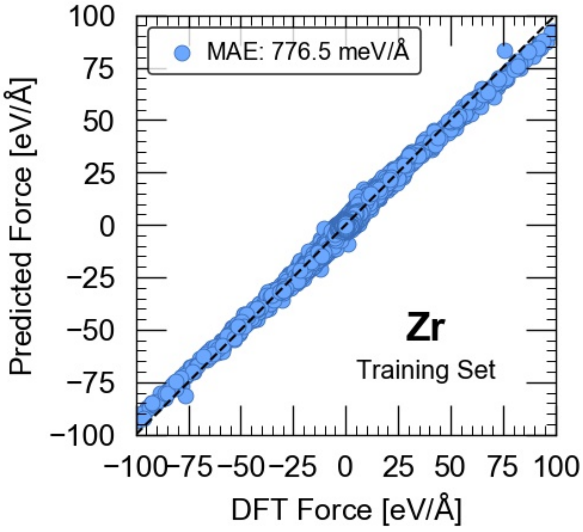

(c)

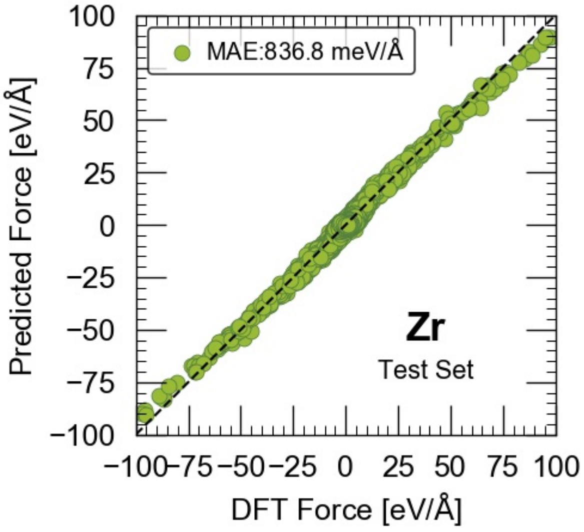

(d)

Supplementary Figure 151: Energy and force correlation plots for Zr nanoclusters

10 Supplementary Note 6

10.1 Performance of c-MCTS trained HyBOP Potentials: Bulk Properties

Supplementary Figure 3 show the performance of the c-MCTS trained HyBOP potentials in predicting bulk properties. HyBOP captures the cohesive energy of the several different polymorphs (including the ground state) of the 54 elements studied. Additionally, predictions for the six lattice parameters corresponding to each of these $\sim$ 200 polymorphs are shown in Supplementary Figure 4. These results clearly demonstrate the universality of our approach—being able to achieve accurate results across 54 different elements—as well as the generality of the developed potentials—being able to accurately predict properties outside of the potential training. Here, we have tabulated the bulk properties such as cohesive energies (Supplementary Table 6), lattice constants (Supplementary Table 7), and elastic properties (Supplementary Table 7) for each of the 54 different elements and their polymorphs.

Supplementary Table 6: Predicted cohesive energies of $\sim$ 200 different polymorphs of 54 different elements from c-MCTS optimized hybrid bond order potential parameters. The bulk structures have been collected from materials project database [31]. The calculated DFT predicted properties have been evaluated using same VASP settings as for evaluating energies for nanoclusters.

Element	Crystal Structure or Space Group	Cohesive Energy [DFT] (eV/atom)	Cohesive Energy [HyBOP] (eV/atom)	Element	Crystal Structure or Space Group	Cohesive Energy [DFT] (eV/atom)	Cohesive Energy [HyBOP] (eV/atom)
Ag	fcc	-2.511725	-2.678527	Ga	64	-2.709715	-2.77013
Ag	bcc	-2.486285	-2.662565	Ga	63	-2.70549	-2.737447
Ag	hcp	-2.466516	-2.641534	Ga	166	-2.700409	-0.923209
Ag	dc	-1.740683	-1.968567	Ge	dc	-3.727929	-3.161135
Al	fcc	-3.550967	-3.678654	Ge	hcp	-3.437855	-3.044477
Al	hcp	-3.482888	-3.665045	Ge	fcc	-3.408915	-2.949522
Al	bcc	-3.418648	-3.649364	Ge	bcc	-3.396594	-2.510756
Al	dc	-2.787166	-2.666609	Hf	hcp	-6.424324	-6.923274
As	166	-2.976068	-2.905794	Hf	fcc	-6.412572	-6.920017
As	64	-2.956048	-2.755563	Hf	bcc	-6.30179	-6.786385
Au	fcc	-3.039523	-3.110927	Hf	dc	-3.797874	-4.723101
Au	bcc	-3.016642	-3.077583	In	225	-2.398108	-2.451924
Au	hcp	-2.968084	-3.021436	In	139	-2.390626	-2.444852
Au	dc	-2.334834	-1.97954	In	194	-2.375204	-2.446356
B	166	-6.411857	-6.777673	Ir	fcc	-7.438913	-8.100696
B	58	-6.374528	-6.829759	Ir	hcp	-7.406411	-8.010652
Ba	bcc	-1.898709	-1.950853	Ir	bcc	-6.801922	-7.766919
Ba	fcc	-1.878124	-1.891708	Ir	dc	-6.403451	-5.695726
Ba	hcp	-1.835984	-1.889814	K	bcc	-0.815316	-1.448568
Ba	dc	-0.931932	-0.852879	K	hcp	-0.811026	-1.391862
Be	hcp	-3.678366	-3.457156	K	fcc	-0.808695	-1.333891
Be	fcc	-3.648624	-3.45592	K	dc	-0.568378	-1.108746
Be	bcc	-3.643434	-3.383279	Li	bcc	-1.602626	-1.460418
Be	dc	-2.0921	-1.508473	Li	hcp	-1.599063	-1.441768
Bi	166	-2.545872	-2.466887	Li	fcc	-1.594919	-1.342393
Bi	221	-2.501312	-2.443583	Li	dc	-1.087183	-1.005804
Bi	11	-2.499945	-2.440143	Mg	hcp	-1.508292	-1.431628
C	194	-8.085643	-7.691359	Mg	fcc	-1.488512	-1.422104
C	dc	-8.04232	-7.648072	Mg	bcc	-1.473176	-1.418283
Ca	fcc	-1.904007	-1.791543	Mg	dc	-0.742313	-0.717385
Ca	bcc	-1.865393	-1.77714	Mo	bcc	-7.868566	-7.299034
Ca	dc	-0.870288	-0.848194	Mo	fcc	-7.437533	-7.236264
Cd	hcp	-0.805063	-0.501228	Mo	hcp	-7.305558	-7.203089
Cd	fcc	-0.719304	-0.491894	Mo	dc	-5.690853	-5.601266
Cd	bcc	-0.660937	-0.454511	Mn	213	-3.825298	-4.13521
Cd	dc	-0.419069	-0.182275	Mn	dc	-2.509528	-2.802293
Co	hcp	-5.495889	-5.48542	Na	bcc	-1.081903	-1.087598
Co	bcc	-5.4624	-5.482344	Na	fcc	-1.065422	-1.043722
Co	fcc	-5.383827	-5.480283	Na	hcp	-0.943856	-0.900545
Co	dc	-4.289325	-4.286697	Na	dc	-0.743367	-0.547226
Cr	bcc	-7.146919	-6.451581	Nb	bcc	-7.117227	-6.800298
Cr	fcc	-6.736178	-6.387226	Nb	fcc	-6.780568	-6.667064
Cr	hcp	-6.646299	-6.340389	Nb	hcp	-6.7343	-6.503295
Cr	dc	-5.068723	-5.587453	Nb	dc	-4.566032	-4.392304
Cu	fcc	-3.484558	-3.51709	Ni	fcc	-5.561355	-5.191993
Cu	hcp	-3.481414	-3.514532	Ni	hcp	-5.540381	-5.113854
Cu	bcc	-3.455087	-3.492064	Ni	bcc	-5.481357	-5.074967
Cu	dc	-2.48237	-2.034938	Ni	dc	-4.3766	-4.104433
Fe	bcc	-7.635909	-7.555372	Os	hcp	-8.222873	-9.319432
Fe	hcp	-7.539061	-7.555037	Os	fcc	-8.178825	-9.319391
Fe	fcc	-7.504575	-7.543839	Os	bcc	-7.407289	-9.258629
Fe	dc	-6.432157	-6.05063	Os	dc	-7.084982	-6.590547

Element	Crystal Structure or Space Group	Cohesive Energy [DFT] (eV/atom)	Cohesive Energy [HyBOP] (eV/atom)	Element	Crystal Structure or Space Group	Cohesive Energy [DFT] (eV/atom)	Cohesive Energy [HyBOP] (eV/atom)
P	2	-3.523668	-3.175458	Sr	fcc	-1.614341	-1.693771
P	13	-3.518213	-3.141145	Sr	bcc	-1.613174	-1.68943
P	53	-2.603751	-3.064313	Sr	hcp	-1.604187	-1.688577
Pb	fcc	-3.002566	-3.258758	Sr	dc	-0.654009	-0.600016
Pb	hcp	-2.958443	-3.201885	Ta	bcc	-8.188406	-7.261214
Pb	bcc	-2.943543	-3.198847	Ta	fcc	-7.938669	-7.038726
Pb	dc	-2.709097	-2.087622	Ta	hcp	-7.721448	-6.802617
Pd	fcc	-3.756993	-3.851315	Ta	dc	-5.334876	-5.337801
Pd	bcc	-3.713697	-3.824644	Te	152	-2.487637	-2.323882
Pd	hcp	-3.708835	-3.817314	Te	51	-2.454671	-2.309573
Pd	dc	-2.637883	-2.446031	Te	26	-2.453546	-2.306971
Pt	fcc	-5.683154	-7.248088	Te	12	-2.450184	-2.303554
Pt	bcc	-5.557434	-7.213583	Te	221	-2.448225	-2.297885
Pt	hcp	-5.507283	-7.020244	Te	166	-2.44718	-2.289804
Pt	dc	-4.627776	-4.567044	Ti	hcp	-5.46973	-5.659291
Rb	hcp	-0.779024	-0.847858	Ti	fcc	-5.458149	-5.656921
Rb	fcc	-0.778202	-0.846446	Ti	bcc	-5.414838	-5.601304
Rb	bcc	-0.776903	-0.844636	Ti	dc	-3.405656	-2.8036
Rb	dc	-0.513786	-0.373211	Tl	hcp	-2.087312	-2.756243
Re	hcp	-7.773664	-8.893102	Tl	bcc	-2.085762	-2.674979
Re	fcc	-7.717929	-8.892743	Tl	fcc	-2.058703	-2.618666
Re	bcc	-7.471534	-8.766693	Tl	dc	-1.709524	-1.735913
Re	dc	-6.249314	-5.837517	V	bcc	-6.466928	-6.837281
Rh	fcc	-6.259331	-6.274332	V	fcc	-6.190417	-6.563272
Rh	hcp	-6.204678	-6.244817	V	hcp	-6.072358	-6.552652
Rh	bcc	-5.897166	-6.21429	V	dc	-4.220457	-5.587481
Rh	dc	-5.174197	-4.734084	W	bcc	-8.894151	-6.709545
Ru	hcp	-7.834005	-9.589588	W	fcc	-8.402234	-6.683261
Ru	fcc	-7.591943	-9.578795	W	hcp	-8.311161	-6.548693
Ru	bcc	-7.059656	-9.405646	W	dc	-6.279032	-5.344618
Ru	dc	-6.581176	-6.205775	Y	hcp	-4.168051	-4.00342
S	148	-3.179345	-3.034035	Y	fcc	-4.155554	-4.001346
Sb	166	-2.707282	-2.770084	Y	bcc	-4.049993	-4.075835
Sb	221	-2.673574	-2.734482	Y	dc	-2.227476	-2.628001
Sb	11	-2.658342	-2.721367	Zn	hcp	-1.101668	-0.800384
Sc	hcp	-4.194866	-4.080402	Zn	fcc	-1.062862	-0.760212
Sc	fcc	-4.176286	-4.079651	Zn	bcc	-1.025388	-0.587054
Sc	bcc	-4.12053	-4.077934	Zn	dc	-0.652028	-0.405924
Sc	dc	-2.277429	-2.273836	Zr	hcp	-6.289619	-5.861875
Se	152	-2.722448	-2.734832	Zr	fcc	-6.240104	-5.857419
Se	148	-2.702012	-2.362418	Zr	bcc	-6.180042	-5.823846
Si	dc	-4.592794	-4.1469	Zr	dc	-3.917309	-3.412517
Si	hcp	-4.088662	-3.97333				
Si	bcc	-4.072694	-3.744391				
Si	fcc	-4.061759	-3.430897				
Sn	139	-3.13455	-2.864157				

Supplementary Table 7: Predicted lattice parameters of ∼ 200 different polymorphs of 54 different elements from c-MCTS optimized hybrid bond order potential parameters.

Element	Crystal Structure or Space Group	a [DFT] (Å)	a [HyBOP] (Å)	b [DFT] (Å)	b [HyBOP] (Å)	c [DFT] (Å)	c[HyBOP] (Å)	α [DFT] (degree)	α [HyBOP] (degree)	β [DFT] (degree)	β[HyBOP] (degree)	γ [DFT] (degree)	γ [HyBOP] (degree)
Ag	fcc	4.106	4.109	4.106	4.11	4.106	4.109	90	90	90	90	90	90
Ag	bcc	3.262	3.262	3.262	3.262	3.262	3.262	90	90	90	90	90	90
Ag	hcp	2.941	2.935	2.941	2.933	9.313	9.434	90	90.2	90	89.8	120	120
Ag	dc	6.153	6.173	6.153	6.179	6.153	6.174	90	90	90	90	90	90
Al	fcc	4.049	4.077	4.049	4.077	4.049	4.077	90	90	90	90	90	90
Al	hcp	2.998	2.95	2.998	2.95	8.716	9.096	90	90	90	90	120	120
Al	bcc	3.241	3.251	3.241	3.251	3.241	3.251	90	90	90	90	90	90
Al	dc	6.048	6.002	6.048	6.002	6.048	6.002	90	90	90	90	90	90
As	166	3.803	3.836	3.803	3.828	10.781	10.522	90	90	90	90	120	119.8
As	64	5.966	6.671	5.966	5.636	4.464	4.341	90	77.7	90	93	143.191	130.3
Au	fcc	4.136	4.154	4.136	4.154	4.136	4.154	90	90	90	90	90	90
Au	bcc	3.279	3.302	3.279	3.302	3.279	3.302	90	90	90	90	90	90
Au	hcp	3.081	3.062	3.081	3.067	8.652	8.898	90	90	90	90	120	120.2
Au	dc	6.1	6.198	6.1	6.202	6.1	6.198	90	90	90	90	90	90
B	166	4.884	4.787	4.884	4.788	12.519	12.594	90	90	90	90	120	120
B	58	5.021	4.994	5.585	5.637	6.933	6.647	90	90	90	90	90	90
Ba	bcc	4.996	4.943	4.996	4.943	4.996	4.944	90	90	90	90	90	90
Ba	fcc	6.338	6.355	6.338	6.356	6.338	6.355	90	90	90	90	90	90
Ba	hcp	4.357	4.359	4.357	4.358	14.32	14.324	90	90	90	90	120	120
Ba	dc	7.773	7.924	7.773	7.925	7.773	7.924	90	90	90	90	90	90
Be	hcp	2.241	2.237	2.241	2.237	7.227	7.249	90	90	90	90	120	120
Be	fcc	3.165	3.161	3.165	3.161	3.165	3.161	90	90	90	90	90	90
Be	bcc	2.5	2.506	2.5	2.506	2.5	2.506	90	90	90	90	90	90
Be	dc	4.912	4.912	4.912	4.912	4.912	4.912	90	90	90	90	90	90
Bi	166	4.58	4.53	4.58	4.53	12.067	11.845	90	90	90	90	120	120
Bi	221	3.271	3.222	3.271	3.222	3.271	3.222	90	90	90	90	90	90
Bi	11	4.618	4.56	4.769	4.573	6.472	6.299	87.179	85.9	90	90	90	90
C	194	2.462	2.487	2.462	2.486	6.443	6.443	90	90	90	90	120	120
C	dc	3.554	3.556	3.554	3.556	3.554	3.556	90	90	90	90	90	90
Ca	fcc	5.453	5.402	5.453	5.402	5.453	5.402	90	90	90	90	90	90
Ca	bcc	4.281	4.272	4.281	4.272	4.281	4.272	90	90	90	90	90	90
Ca	dc	8.426	8.372	8.426	8.372	8.426	8.372	90	90	90	90	90	90
Cd	hcp	3.261	3.174	3.261	3.182	9.392	9.71	90	90	90	90	120	120.2
Cd	fcc	4.443	4.379	4.443	4.378	4.443	4.379	90	90	90	90	90	90
Cd	bcc	3.508	3.581	3.508	3.516	3.508	3.378	90	90	90	90	90	90
Cd	dc	6.614	6.766	6.614	6.766	6.614	6.766	90	90	90	90	90	90
Co	hcp	2.441	2.43	2.441	2.43	3.948	3.942	90	90	90	90	120	120
Co	bcc	2.778	2.742	2.778	2.742	2.778	2.742	90	90	90	90	90	90
Co	fcc	3.424	3.42	3.424	3.417	3.424	3.422	90	90	90	90	90	90
Co	dc	5.027	4.842	5.027	5.096	5.027	4.876	90	90	90	90	90	90
Cr	bcc	2.826	2.744	2.826	2.744	2.826	2.744	90	90	90	90	90	90
Cr	fcc	3.58	3.472	3.58	3.472	3.58	3.472	90	90	90	90	90	90
Cr	hcp	2.425	2.434	2.425	2.426	8.835	8.239	90	89.9	90	90	120	119.8
Cr	dc	5.032	4.902	5.032	4.902	5.032	4.902	90	90	90	90	90	90
Cu	fcc	3.612	3.673	3.612	3.668	3.612	3.676	90	90	90	90	90	90
Cu	hcp	2.569	2.61	2.569	2.61	8.251	8.42	90	90	90	90	120	120
Cu	bcc	2.87	2.903	2.87	3.063	2.87	2.902	90	90	90	90.3	90	90.1
Cu	dc	5.328	5.462	5.328	5.462	5.328	5.462	90	90	90	90	90	90

Element	Crystal Structure or Space Group	a [DFT] (Å)	a [HyBOP] (Å)	b [DFT] (Å)	b [HyBOP] (Å)	c [DFT] (Å)	c[HyBOP] (Å)	α [DFT] (degree)	α [HyBOP] (degree)	β [DFT] (degree)	β[HyBOP] (degree)	γ [DFT] (degree)	γ [HyBOP] (degree)
Fe	bcc	2.795	2.792	2.795	2.792	2.795	2.792	90	90	90	90	90	90
Fe	hcp	2.439	2.452	2.439	2.452	7.895	7.994	90	90	90	90	120	120
Fe	fcc	3.428	3.464	3.428	3.459	3.428	3.466	90	90	90	90	90	90
Fe	dc	4.837	4.826	4.837	4.826	4.837	4.826	90	90	90	90	90	90
Ga	64	4.498	4.388	4.498	4.566	4.613	4.442	90	89.6	90	90	119.311	118.3
Ga	63	4.561	4.504	4.561	4.616	4.613	4.572	90	90	90	90	118.229	119.6
Ga	166	7.835	10.833	7.835	8.518	7.835	8.321	72.227	95.7	72.227	70	72.227	78.1
Ge	dc	5.784	5.86	5.784	5.855	5.784	5.86	90	90.2	90	90.2	90	90
Ge	hcp	3.02	3.03	3.02	3.032	9.792	9.89	90	90	90	90.1	120	120
Ge	fcc	4.272	4.257	4.272	4.258	4.272	4.262	90	90.2	90	90.3	90	90.2
Ge	bcc	3.402	3.404	3.402	3.671	3.402	3.125	90	90	90	90.4	90	89.9
Hf	hcp	2.373	2.314	2.373	2.314	7.688	7.47	90	90	90	90	120	120
Hf	fcc	3.348	3.267	3.348	3.266	3.348	3.267	90	90	90	90	90	90
Hf	bcc	2.642	2.607	2.642	2.607	2.642	2.607	90	90	90	90	90	90
Hf	dc	6.438	6.242	6.438	6.243	6.438	6.243	90	90.2	90	90	90	90
In	225	3.383	3.367	3.383	3.367	3.383	3.367	60	60	60	60	60	60
In	139	3.482	3.395	3.482	3.396	3.482	3.414	124.831	122	124.831	122.1	81.817	86.4
In	194	3.347	3.349	3.347	3.349	5.692	5.582	90	90	90	90	120	120
Ir	fcc	3.86	3.86	3.86	3.86	3.86	3.86	90	90	90	90	90	90
Ir	hcp	2.742	2.734	2.742	2.735	8.913	8.914	90	90	90	90	120	120
Ir	bcc	3.102	3.1	3.102	3.101	3.102	3.098	90	90	90	90	90	90
Ir	dc	5.553	5.56	5.553	5.56	5.553	5.56	90	90	90	90	90	90
K	bcc	4.962	4.33	4.962	4.331	4.962	4.33	90	90	90	90	90	90
K	hcp	4.494	4.106	4.494	4.193	7.042	6.48	90	90	90	90	120	122
K	fcc	4.179	3.808	4.179	3.808	4.179	3.807	90	90	90	90	90	90
K	dc	9.12	7.793	9.12	7.793	9.12	7.793	90	90	90	90	90	90
Li	bcc	3.433	3.506	3.433	3.495	3.433	3.658	90	90.1	90	90.3	90	89.6
Li	hcp	3.082	3.145	3.082	3.129	4.94	5.058	90	90	90	90	120	119.6
Li	fcc	4.311	4.457	4.311	4.454	4.311	4.449	90	90.1	90	90	90	90
Li	dc	6.038	5.876	6.038	6.788	6.038	5.908	90	90.1	90	90.2	90	90
Mg	hcp	3.181	3.234	3.181	3.234	5.223	5.306	90	90	90	90.1	120	120
Mg	fcc	4.516	4.607	4.516	4.6	4.516	4.611	90	90.1	90	90.2	90	90.1
Mg	bcc	3.575	3.655	3.575	3.656	3.575	3.652	90	90	90	90	90	90
Mg	dc	6.822	6.669	6.822	6.964	6.822	6.69	90	90.1	90	90.2	90	90
Mo	bcc	3.142	3.042	3.142	3.042	3.142	3.042	90	90	90	90	90	90
Mo	fcc	3.983	3.842	3.983	3.841	3.983	3.842	90	90	90	90	90	90
Mo	hcp	2.693	2.681	2.693	2.675	9.834	9.179	90	90	90	90	120	119.9
Mo	dc	5.632	5.48	5.632	5.48	5.632	5.48	90	90	90	90	90	90
Mn	213	5.987	5.79	5.987	5.79	5.987	5.79	90	90	90	90	90	90
Mn	dc	4.905	4.916	4.905	4.916	4.905	4.916	90	90	90	90	90	90
Na	bcc	4.182	3.897	4.182	3.897	4.182	3.897	90	90	90	90	90	90
Na	fcc	5.28	5.062	5.28	5.062	5.28	5.061	90	90	90	90	90	90
Na	hcp	5.315	4.877	5.315	4.931	6.914	6.48	90	90	90	90	120	121.3
Na	dc	7.449	7.183	7.449	7.183	7.449	7.183	90	90	90	90	90	90
Nb	bcc	3.314	3.19	3.314	3.19	3.314	3.19	90	90	90	90	90	90
Nb	fcc	4.227	4.083	4.227	4.083	4.227	4.083	90	90	90	90	90	90
Nb	hcp	2.811	2.779	2.811	2.786	10.69	10.444	90	90	90	90	120	120.2
Nb	dc	5.93	5.914	5.93	5.914	5.93	5.914	90	90	90	90	90	90
Ni	fcc	3.493	3.484	3.493	3.479	3.493	3.486	90	90.1	90	90.1	90	90
Ni	hcp	2.494	2.48	2.494	2.481	7.922	7.993	90	90	90	90.1	120	120
Ni	bcc	2.786	2.786	2.786	2.786	2.786	2.786	90	90	90	90	90	90
Ni	dc	5.069	4.878	5.069	5.044	5.069	5.227	90	90	90	90.1	90	90

Element	Crystal Structure or Space Group	a [DFT] (Å)	a [HyBOP] (Å)	b [DFT] (Å)	b [HyBOP] (Å)	c [DFT] (Å)	c[HyBOP] (Å)	α [DFT] (degree)	α [HyBOP] (degree)	β [DFT] (degree)	β[HyBOP] (degree)	γ [DFT] (degree)	γ [HyBOP] (degree)
Os	hcp	2.735	2.722	2.735	2.722	8.78	8.817	90	90	90	90	120	120
Os	fcc	3.842	3.836	3.842	3.836	3.842	3.836	90	90	90	90	90	90
Os	bcc	3.081	3.066	3.081	3.066	3.081	3.066	90	90	90	90	90	90
Os	dc	5.53	5.523	5.53	5.776	5.53	5.518	90	90	90	90	90	90
P	2	7.365	7.302	11.921	11.977	12.545	12.456	104.485	104.6	106.151	106.2	98.875	98.6
P	13	9.202	9.224	9.25	9.252	23.366	23.196	74.841	74.5	90	90	90	90
P	53	3.262	3.31	4.743	4.677	7.407	8.458	90	90	90	90	90	90.1
Pb	fcc	3.327	3.233	3.327	3.242	3.327	3.038	90	90	90	89.9	90	90
Pb	hcp	2.378	2.237	2.378	2.244	7.657	7.271	90	90	90	89.7	120	120.2
Pb	bcc	2.66	2.586	2.66	2.587	2.66	2.58	90	90	90	90	90	90
Pb	dc	7.025	7.147	7.025	7.147	7.025	7.147	90	90	90	90	90	90
Pd	fcc	3.917	3.928	3.917	3.926	3.917	3.93	90	90	90	90	90	90
Pd	bcc	3.12	2.959	3.12	3.547	3.12	2.932	90	90	90	90.1	90	89.9
Pd	hcp	2.81	2.794	2.81	2.794	8.846	8.98	90	90	90	90	120	120
Pd	dc	5.745	5.752	5.745	5.752	5.745	5.752	90	90	90	90	90	90
Pt	fcc	3.956	3.97	3.956	3.97	3.956	3.97	90	90	90	90	90	90
Pt	bcc	3.142	3.152	3.142	3.152	3.142	3.152	90	90	90	90	90	90
Pt	hcp	2.88	2.862	2.88	2.866	8.532	8.779	90	90	90	90	120	120.1
Pt	dc	5.746	5.876	5.746	5.926	5.746	5.88	90	90	90	90	90	90
Rb	hcp	5.099	4.548	5.099	4.594	16.192	14.668	90	90	90	90	120	120.9
Rb	fcc	7.144	6.529	7.144	6.529	7.144	6.529	90	90	90	90	90	90
Rb	bcc	5.662	5.264	5.662	5.264	5.662	5.264	90	90	90	90	90	90
Rb	dc	10.348	10.243	10.348	10.243	10.348	10.243	90	90	90	90	90	90
Re	hcp	2.757	2.752	2.757	2.753	8.983	8.978	90	90	90	90	120	120
Re	fcc	3.905	3.896	3.905	3.896	3.905	3.896	90	90	90	90	90	90
Re	bcc	3.107	3.094	3.107	3.094	3.107	3.094	90	90	90	90	90	90
Re	dc	5.622	5.776	5.622	5.776	5.622	5.776	90	90	90	90	90	90
Rh	fcc	3.802	3.755	3.802	3.756	3.802	3.755	90	90	90	90	90	90
Rh	hcp	2.707	2.659	2.707	2.659	8.714	8.749	90	90	90	90	120	120
Rh	bcc	3.05	3.05	3.05	3.05	3.05	3.05	90	90	90	90	90	90
Rh	dc	5.47	5.458	5.47	5.458	5.47	5.458	90	90	90	90	90	90
Ru	hcp	2.706	2.646	2.706	2.647	4.246	4.228	90	90	90	90	120	120
Ru	fcc	3.783	3.72	3.783	3.72	3.783	3.72	90	90	90	90	90	90
Ru	bcc	3.03	2.971	3.03	2.971	3.03	2.971	90	90	90	90	90	90
Ru	dc	5.402	5.532	5.402	5.532	5.402	5.532	90	90	90	90	90	90
S	148	6.387	6.238	6.387	6.462	6.387	6.316	113.668	112.9	113.668	115.8	113.668	113.7
Sb	166	4.37	4.382	4.37	4.377	11.588	11.715	90	90	90	90	120	120
Sb	221	3.104	3.142	3.104	3.196	3.104	3.14	90	90	90	90	90	90.1
Sb	11	4.362	4.452	3.161	3.128	4.359	4.329	88.936	89.9	90	89.9	90	88.4
Sc	hcp	3.273	3.303	3.273	3.3	5.175	5.299	90	90	90	90	120	119.8
Sc	fcc	4.586	4.631	4.586	4.631	4.586	4.632	90	90	90	90	90	90
Sc	bcc	3.653	3.605	3.653	3.605	3.653	3.605	90	90	90	90	90	90
Sc	dc	6.402	7.281	6.402	7.281	6.402	7.281	90	90	90	90	90	90
Se	152	4.349	4.153	4.349	4.18	5.075	5.324	90	91.8	90	87.8	120	120.8
Se	148	6.57	7.322	6.57	7.053	6.57	7.006	113.509	130.1	113.509	98.6	113.509	114.2
Si	dc	5.465	5.531	5.465	5.53	5.465	5.53	90	90	90	90	90	90
Si	hcp	2.712	2.721	2.712	2.722	9.047	9.006	90	90	90	90.2	120	120
Si	bcc	3.075	3.243	3.075	2.986	3.075	2.902	90	90	90	90.1	90	90.1
Si	fcc	3.863	3.831	3.863	3.828	3.863	3.833	90	90.1	90	90.2	90	90
Sn	139	3.309	3.359	3.309	3.359	3.309	3.357	105.538	105.8	105.538	105.6	117.661	117.4

Element	Crystal Structure or Space Group	a [DFT] (Å)	a [HyBOP] (Å)	b [DFT] (Å)	b [HyBOP] (Å)	c [DFT] (Å)	c[HyBOP] (Å)	α [DFT] (degree)	α [HyBOP] (degree)	β [DFT] (degree)	β[HyBOP] (degree)	γ [DFT] (degree)	γ [HyBOP] (degree)
Sr	fcc	4.485	4.384	4.485	4.384	4.485	4.38	90	90	90	90	90	90
Sr	bcc	3.538	3.499	3.538	3.499	3.538	3.499	90	90	90	90	90	90
Sr	hcp	3.196	3.11	3.196	3.11	10.291	10.109	90	90	90	90	120	120
Sr	dc	9.463	9.302	9.463	9.302	9.463	9.302	90	90	90	90	90	90
Ta	bcc	3.304	3.3	3.304	3.3	3.304	3.299	90	90	90	90.1	90	90
Ta	fcc	4.2	4.201	4.2	4.196	4.2	4.206	90	90.2	90	90.2	90	90.1
Ta	hcp	2.816	2.924	2.816	2.913	10.391	9.932	90	90	90	90.1	120	119.8
Ta	dc	6.052	6.079	6.052	6.079	6.052	6.079	90	90	90	90	90	90
Te	152	4.481	4.463	4.481	4.464	5.971	5.963	90	90	90	90	120	120
Te	51	3.159	3.122	4.647	4.502	8.893	8.816	90	90	90	90	90	90
Te	26	3.173	3.129	4.63	4.494	8.891	8.815	90	90	90	90	90	90
Te	12	4.452	4.392	4.452	4.398	5.744	5.612	89.521	89.8	89.521	89.7	60.119	60
Te	221	3.196	3.141	3.196	3.141	3.196	3.141	90	90	90	90	90	90
Te	166	3.196	3.141	3.196	3.141	3.196	3.141	88.646	89	88.646	89	88.646	89
Ti	hcp	2.902	2.91	2.902	2.91	9.366	9.446	90	90	90	90	120	120
Ti	fcc	4.082	4.105	4.082	4.105	4.082	4.105	90	90	90	90	90	90
Ti	bcc	3.229	3.238	3.229	3.238	3.229	3.238	90	90	90	90	90	90
Ti	dc	5.894	6.289	5.894	6.289	5.894	6.289	90	90.1	90	90.1	90	90.1
Tl	hcp	3.608	3.346	3.608	3.37	5.49	5.299	90	90	90	90	120	120.6
Tl	bcc	3.961	3.914	3.961	3.914	3.961	3.914	90	90	90	90	90	90
Tl	fcc	5.002	4.954	5.002	4.955	5.002	4.954	90	90	90	90	90	90
Tl	dc	7.034	7.109	7.034	7.111	7.034	7.108	90	89.9	90	89.8	90	90
V	bcc	2.969	2.956	2.969	2.961	2.969	2.952	90	90	90	89.8	90	89.8
V	fcc	3.768	3.76	3.768	3.8	3.768	3.754	90	89.8	90	89.6	90	89.8
V	hcp	2.496	2.54	2.496	2.532	9.523	9.285	90	90	90	90	120	119.6
V	dc	5.388	5.269	5.388	5.269	5.388	5.269	90	90	90	90	90	90
W	bcc	3.165	3.218	3.165	3.198	3.165	3.217	90	90	90	90.2	90	90.1
W	fcc	4.011	4.003	4.011	4	4.011	4.01	90	90.2	90	90.4	90	90.2
W	hcp	2.743	2.874	2.743	2.876	9.787	9.712	90	89.9	90	90.2	120	120
W	dc	5.788	5.664	5.788	5.838	5.788	5.611	90	90.2	90	90.4	90	90.2
Y	hcp	3.57	3.449	3.57	3.449	5.727	5.582	90	90	90	90	120	120
Y	fcc	5.038	4.871	5.038	4.874	5.038	4.872	90	90	90	90	90	90
Y	bcc	4.032	3.931	4.032	3.931	4.032	3.931	90	90	90	90	90	90
Y	dc	7.061	6.444	7.061	6.444	7.061	6.444	90	90	90	90	90	90
Zn	hcp	2.627	2.677	2.627	2.669	5.251	4.891	90	90	90	90	120	119.7
Zn	fcc	3.899	3.931	3.899	3.927	3.899	3.934	90	90.2	90	90.2	90	90.2
Zn	bcc	3.089	3.089	3.089	3.088	3.089	3.087	90	90	90	90.1	90	90
Zn	dc	5.779	5.623	5.779	5.907	5.779	6.54	90	90.1	90	90.1	90	90.1
Zr	hcp	3.215	3.223	3.215	3.223	5.147	5.212	90	90	90	90	120	120
Zr	fcc	4.477	4.523	4.477	4.523	4.477	4.523	90	90	90	90	90	90
Zr	bcc	3.531	3.524	3.531	3.524	3.531	3.524	90	90	90	90	90	90
Zr	dc	6.235	6.67	6.235	6.86	6.235	6.864	90	90.6	90	90.4	90	90.2

Supplementary Table 8: The stiffness modulus ($C_{11}, C_{22}, C_{33}, C_{44}, C_{12}$ and C_{13} for the considered 54 elements, as predicted by the c-MCTS optimized Hybrid Bond Order potentials. The relevant structures in each case were collected from the Materials Project database [31].

Element	Crystal Structure or Space Group	C_{11} [DFT] (GPA)	C_{11} [HyBOP] (GPA)	C_{22} [DFT] (GPA)	C_{22} [HyBOP] (GPA)	C_{33} [DFT] (GPA)	C_{33} [HyBOP] (GPA)	C_{44} [DFT] (GPA)	C_{44} [HyBOP] (GPA)	C_{12} [DFT] (GPA)	C_{12} [HyBOP] (GPA)	C_{13} [DFT] (GPA)	C_{13} [HyBOP] (GPA)
Ag	fcc	100	124.4	100	120.4	100	123.5	41	38.2	82	102	82	103.2
Al	fcc	104	122	104	121.9	104	121.6	32	47.3	73	94	73	93.6
As	166	127	64	127	64	30	82.8	32	24	40	39.9	25	58.7
Au	fcc	144	142.4	144	142.4	144	142.3	29	62	134	107.1	134	107.2
B	166	453	381.4	453	379.5	602	395.9	208	35.3	113	265.6	43	309.5
Ba	bcc	12	9.8	12	9.7	12	9.7	10	17.4	7	5.9	7	5.9
Be	hcp	322	620.5	322	620.5	378	728.4	162	147	21	328	8	205.8
Bi	166	68	42.9	68	46.4	30	53.0	10	2.6	24	36.9	19	36.3
C	194	904	991.1	904	992.2	8	0	2	0	159	422.8	-1	-0.2
Ca	fcc	21	31.2	21	31.2	21	31.2	14	22.6	15	19.3	15	19.3
Cd	hcp	87	103.6	87	100.7	43	121.2	11	27.9	39	40.5	28	30.9
Co	hcp	358	279	358	273.8	409	367	95	58.7	165	174.6	114	103.5
Cr	bcc	499	300	499	253	499	326	102	91.4	139	193.5	139	137.5
Cu	fcc	180	144.1	180	140.3	180	142.4	78	108.3	127	130.6	127	127
Fe	bcc	247	265.4	247	190.4	247	261.2	97	85	150	165	150	92.8
Ga	64	91	46	116	43.6	84	45.3	29	33.8	22	33.1	31	30
Ge	dc	104	61.6	104	59.2	104	61.7	56	5.9	37	53.3	37	53.8
Hf	hcp	184	329.1	184	328	194	253.9	52	73.6	70	234.9	68	69.2
In	225	42	72.4	42	72.2	43	75.8	6	14.6	23	35.1	33	31.1
Ir	fcc	576	247.6	576	247.3	576	245.6	252	179.4	231	231.4	231	226.7
K	bcc	3	13.6	3	13	3	14.2	3	9.2	4	2.6	4	4.4
Li	bcc	15	13.6	15	26.4	15	16.1	11	24.2	13	16.8	13	12.2
Mg	hcp	58	60	58	55.2	66	68.5	20	12.2	30	34.1	22	7.9
Mo	bcc	472	270.5	472	207.5	472	269	106	111.1	158	167	158	97.5
Mn	213	349	348.1	350	348.1	350	348.1	99	141.8	221	122.8	221	122.8
Na	bcc	9	20.3	9	20.4	9	20.4	7	14.9	7	11.5	7	11.5
Nb	bcc	233	238.1	233	237.9	233	237.8	11	169.3	145	226.9	145	226.9
Ni	fcc	276	283	276	279.4	276	272.8	132	128.6	159	224.4	159	211.3
Os	hcp	730	498.2	730	498.6	824	528.7	252	93.1	226	233.1	220	193.5
P	64	43	579.2	13	34	177	-1870.6	4	116.2	-3	-1098.4	32	1388.3
Pb	fcc	47	59.3	47	70.8	47	106.9	18	37.6	32	22.4	32	50.9
Pd	fcc	187	151	187	148.9	187	144.6	71	71.2	147	106	147	100.1
Pt	fcc	303	276	303	277	303	274.4	54	138	220	183.1	220	181
Rb	hcp	4	14.9	4	14.9	5	14.1	1	3.7	2	7.1	2	4.3
Re	hcp	607	633.6	607	633.6	670	689.3	156	126.6	273	267.5	214	211.1
Rh	fcc	397	233.3	397	228.1	397	232.8	177	100.7	182	183.4	182	186
Ru	hcp	559	784.9	559	784.9	635	945.8	181	214.8	178	336.6	166	262.6
S	148	4	4.6	4	6.7	2	4	1	1.6	0	2.4	1	1.9
Sb	166	91	68.4	91	67.3	38	36.4	27	17.7	24	20.2	21	25.7
Sc	hcp	98	61.8	98	61.8	89	75.4	28	34.3	34	-14	30	-27.8
Se	152	7	31.0	7	28.8	69	36.6	11	7.9	2	14.4	8	19.7
Si	dc	144	109.7	144	109.7	144	109.8	75	13.4	53	90.4	53	90.4
Sn	227	59	64.8	59	65.7	59	12.4	26	2.3	28	43	28	27.8
Ta	bcc	265	270.2	265	269.2	265	261.7	69	179.4	158	284.5	158	281.8
Te	152	30	22.9	30	22.6	66	18.8	29	9.3	7	3.3	21	4.4
Ti	hcp	177	343.7	177	343.3	191	408.2	42	77.4	83	153.8	76	92.7
Tl	hcp	32	46.5	32	54.7	48	89	7	11.2	21	50.1	22	20.6
V	bcc	276	147.6	276	117	276	151.7	16	17.2	131	130.7	131	117.9
W	bcc	510	286.2	510	364.4	510	360.8	143	53.4	201	216.1	201	213.4
Y	hcp	77	58.5	77	58.5	81	78.9	25	4	26	19.6	21	3.8
Zn	hcp	163	151.8	163	148.6	61	96.7	32	18.4	45	66.9	48	18.5
Zr	hcp	144	165.8	144	165.7	162	184.3	26	43.8	65	67.4	67	48.7

11 Supplementary Note 7

11.1 Normal Modes Analysis

Normal Modes analysis is a much more rigorous test of the interatomic potentials and typically done for single molecules and soft materials. The results are plotted in Supplementary Figure 8. We have additionally analyzed the normal modes using the RL trained force fields (for at least one representative element from each group of the Periodic table) and compared them against those from DFT computations. We observe that the computed normal modes match very well with the DFT computed normal modes. The agreement is quite remarkable ($R^2 \sim > 0.8$) considering the diversity of the cluster configurations sampled in this work.

The computational methodology is discussed in the following section.

11.2 Methods to Calculate Normal Modes [118, 119]

In order to calculate the Normal Modes, we first built the Hessian Matrix which is defined as:

$$H_{i,j} = \frac{\delta^2 E}{\delta x_i \delta x_j} \tag{19}$$

and the Hessian Matrix is generated by using finite displacements. Firstly, the atomic coordinate x_i is incremented by $\frac{1}{2}\Delta x_j$ and its gradients the gradients calculated, then the coordinate is decremented by Δx_j and the gradients re-calculated. The second derivative is then obtained from the difference of the two derivatives and the step size:

$$H_{i,j} = \frac{(\frac{\delta E}{\delta x_i})_{+0.5\Delta x_j} - (\frac{\delta E}{\delta x_i})_{-0.5\Delta x_j}}{\Delta x_j} \tag{20}$$

which is performed for all $3N$ Cartesian coordinates. Since, $H_{i,j} = H_{j,i}$ (i.e. Hessian Matrix is symmetric), the random errors that arise in the gradient calculation can be reduced (by a factor of $(1/2)^{(1/2)}$ by reformulating the Hessian as:

$$H_{i,j} = \frac{1}{2}(\frac{(\frac{\delta E}{\delta x_i})_{+0.5\Delta x_j} - (\frac{\delta E}{\delta x_i})_{-0.5\Delta x_j}}{\Delta x_j} + \frac{(\frac{\delta E}{\delta x_j})_{+0.5\Delta x_i} - (\frac{\delta E}{\delta x_j})_{-0.5\Delta x_i}}{\Delta x_i}) \tag{21}$$

In order to calculate the vibrational frequencies it must first be mass-weighted

$$H^m_{i,j} = \frac{H_{i,j}}{\sqrt{M_i M_j}} \tag{22}$$

Diagonalization of this matrix yields eigenvalues, λ_i, from which the vibrational frequencies, ν_i can be calculated using $\lambda = \nu^2$.

We determined reference frequencies by employing the approach in which the Hessian matrix is calculated using finite differences, as implemented in VASP [120, 121].

11.3 Additional Details on Dynamical Stability of Clusters

We further validate our HyBOP potentials by performing rigorous test of their dynamic stability. Specifically, we performed molecular dynamics simulations for over 40,000 clusters across each of the 54 elements. The clusters varied in their shape, size and the number of constituting atoms. LAMMPS software [122] was employed for canonical (NVT) molecular dynamics simulations for a period of 1 ns, with the results for some representative cases shown in Figures 152 - 258. We note that the dynamical stability of nanoclusters is amongst the most stringent test that many of the well-known potentials fail to accomplish. However, for the HyBOP potentials developed in this work, all of the 40,000 clusters across various size ranges and topologies tested were indeed found to be dynamically stable. This highlights both the accuracy of the developed potentials as well as the comprehensive sampling of the configuration space within our training set.

Supplementary Table 9: Temperature and other details during MD simulations of elemental nanoclusters

Element	Melting point in K (relevant phase)	Temperature during NVT run(K)	# of cluster tested	Element	Melting Point in K (relevant phase)	Temperature during NVT run(K)	# of cluster tested
Ag	1234.93(fcc)	300	1384	Ni	1728.30(fcc)	300	1076
Al	933.47(fcc)	300	1376	Os	3306(hcp)	300	199
As	1090(rho)	300	917	P	317.3 (white)	150	998
Au	1337.33(fcc)	300	1286	Pb	600.61(fcc)	300	569
B	2348(bet)	300	669	Pd	1828(fcc)	300	629
Ba	1000(bcc)	300	370	Pt	2041.5(fcc)	300	1236
Be	1560(bcc)	300	918	Rb	312.45(bcc)	150	536
Bi	544.5(rho)	200	1062	Re	3459(hcp)	300	243
C	4765.3(hex)	300	421	Rh	2237(fcc)	300	410
Ca	1115.15(bcc)	300	235	Ru	2607(hcp)	300	270
Cd	594.2(hcp)	200	1189	S	388.35(mono)	200	29
Co	1768(fcc)	300	932	Sb	903.78(rho)	300	1147
Cr	2180(bcc)	300	1060	Sc	1814(bcc)	300	1011
Cs	301.59(bcc)	150	1215	Se	493(hex)	300	615
Cu	1357.77(fcc)	300	1234	Si	1687(dia)	300	1161
Fe	1811.(bcc)	300	745	Sn	505.07(bct)	200	331
Ga	302.91(ort)	150	834	Sr	1050(bcc)	300	652
Ge	1211.4(dia)	300	825	Ta	3290(bcc)	300	209
Hf	2506(bcc)	300	986	Te	722.65(hex)	300	570
In	429.75(tet)	200	206	Ti	1941(bcc)	300	93
Ir	2719(fcc)	300	254	Tl	577(bcc)	200	1171
K	336.53(bcc)	150	1063	V	2183(bcc)	300	175
Li	453.6(bcc)	200	1061	W	3695(bcc)	300	379
Mg	923(hcp)	300	1210	Y	1799(bcc)	300	229
Mn	1519(bcc)	300	223	Zn	692.68(hcp)	300	1132
Mo	2896.(bcc)	300	1099	Zr	2127.85(bcc)	300	944
Na	370.87(bcc)	200	1367	Total			40361
Nb	2750(bcc)	300	206				

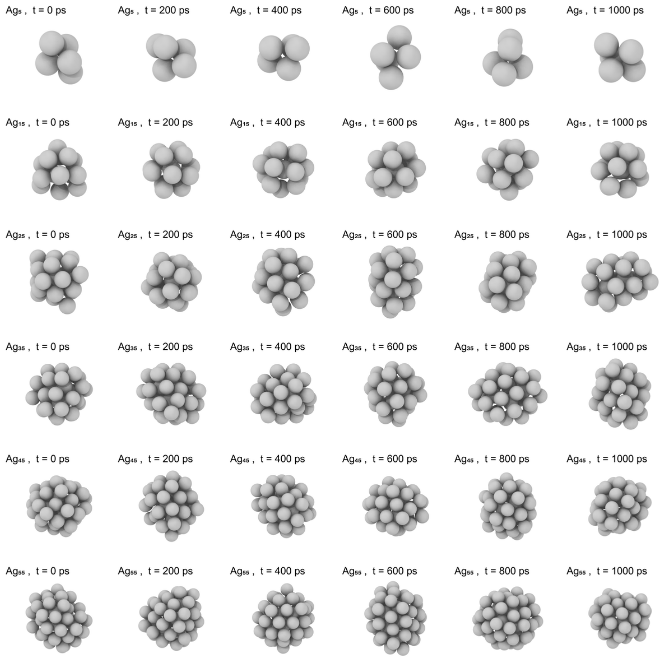

Supplementary Figure 152: MD simulation trajectories of Ag Nanoclusters

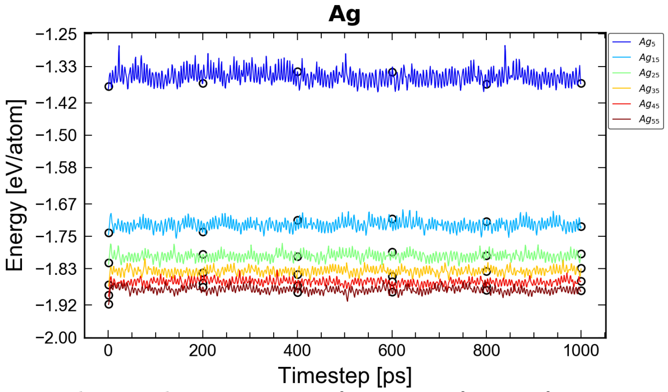

Supplementary Figure 153: The total energy as a function of time from exemplary MD simulations of Ag nanoclusters. Snapshots of MD simulation trajectories are provided in Supplementary Figure 152

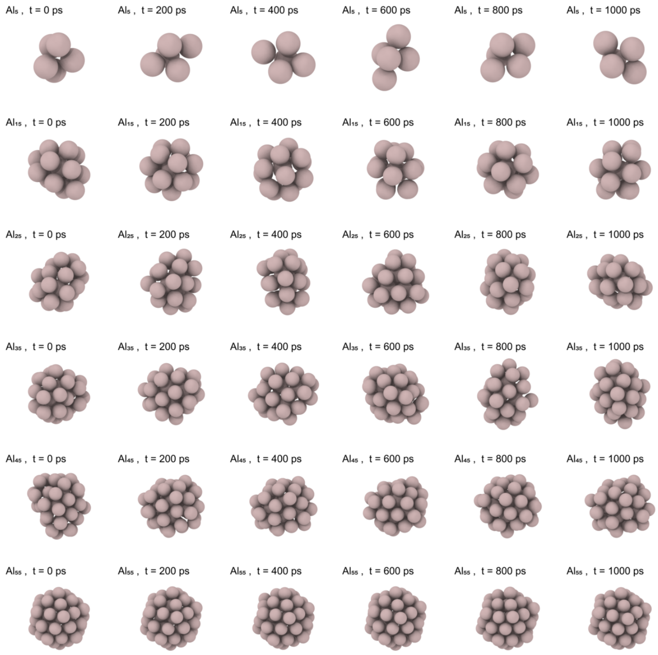

Supplementary Figure 154: MD simulation trajectories of Al Nanoclusters

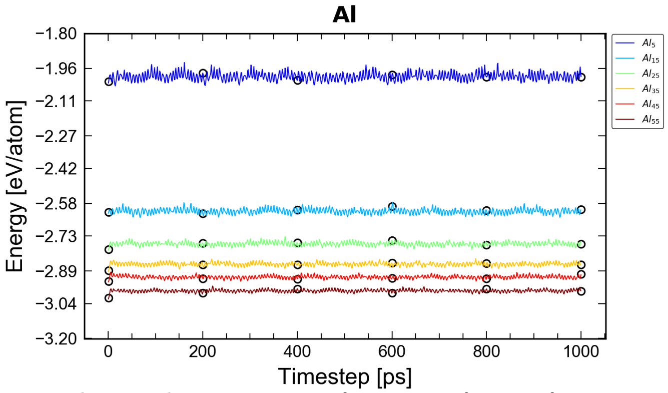

Supplementary Figure 155: The total energy as a function of time from exemplary MD simulations of Al nanoclusters. Snapshots of MD simulation trajectories are provided in Supplementary Figure 154

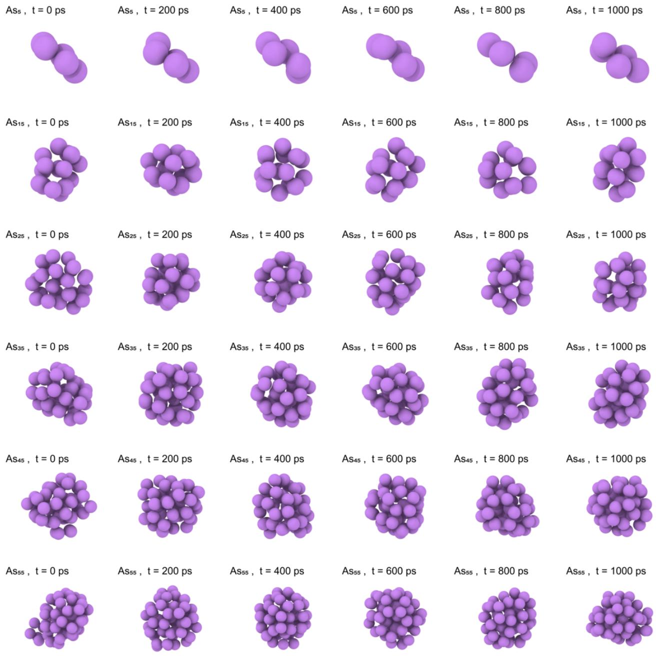

Supplementary Figure 156: MD simulation trajectories of As Nanoclusters

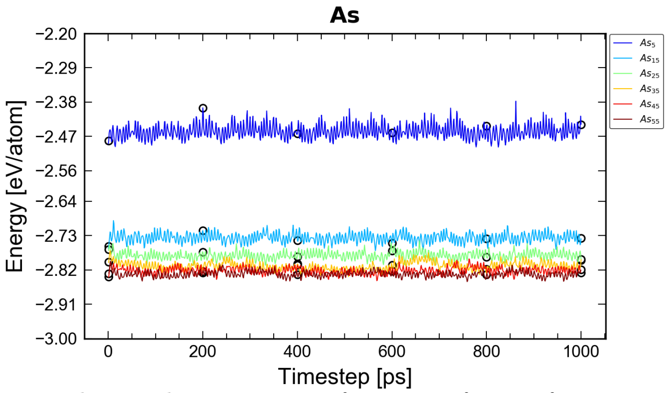

Supplementary Figure 157: The total energy as a function of time from exemplary MD simulations of Al nanoclusters. Snapshots of MD simulation trajectories are provided in Supplementary Figure 156

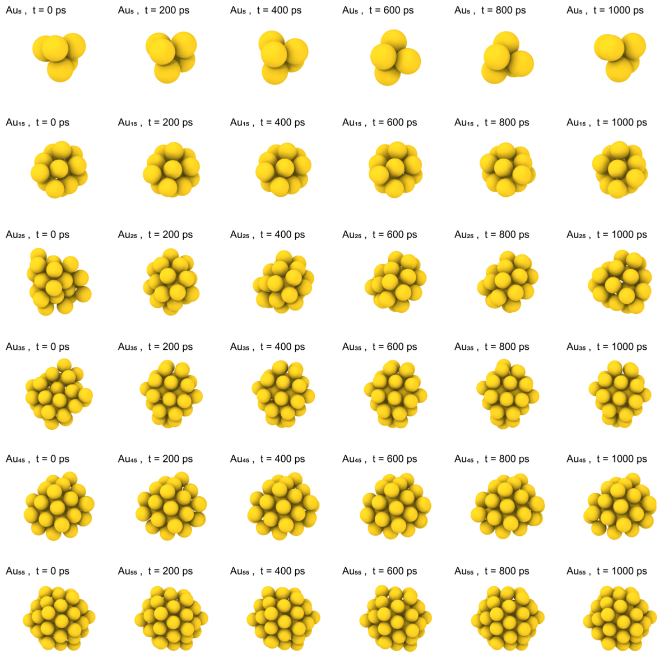

Supplementary Figure 158: MD simulation trajectories of Au Nanoclusters

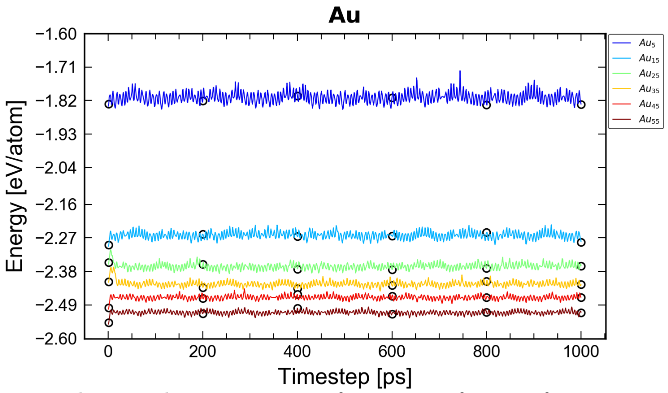

Supplementary Figure 159: The total energy as a function of time from exemplary MD simulations of Au nanoclusters. Snapshots of MD simulation trajectories are provided in Supplementary Figure 158

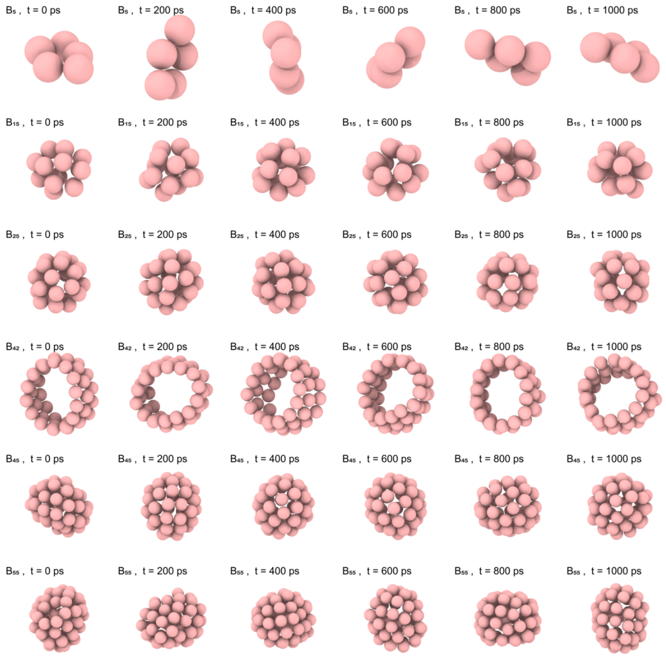

Supplementary Figure 160: MD simulation trajectories of B Nanoclusters

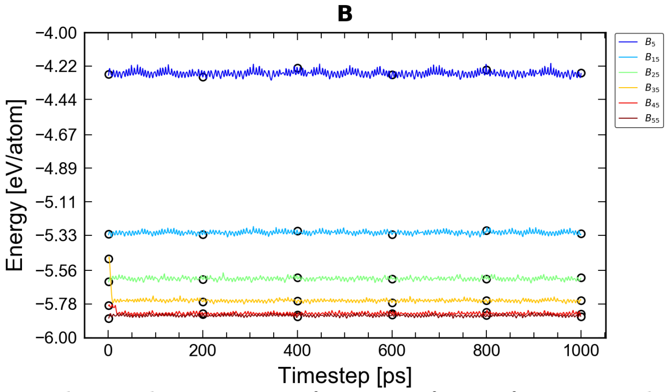

Supplementary Figure 161: The total energy as a function of time from exemplary MD simulations of B nanoclusters. Snapshots of MD simulation trajectories are provided in Supplementary Figure 160

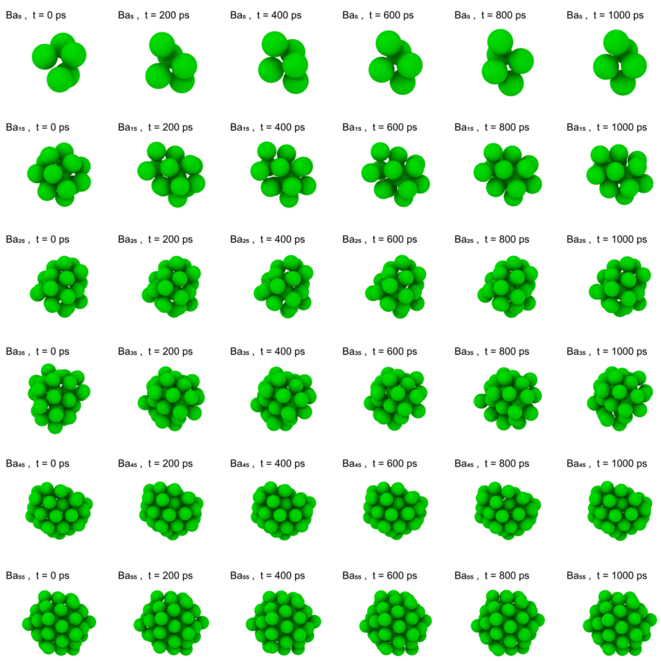

Supplementary Figure 162: MD simulation trajectories of Ba Nanoclusters

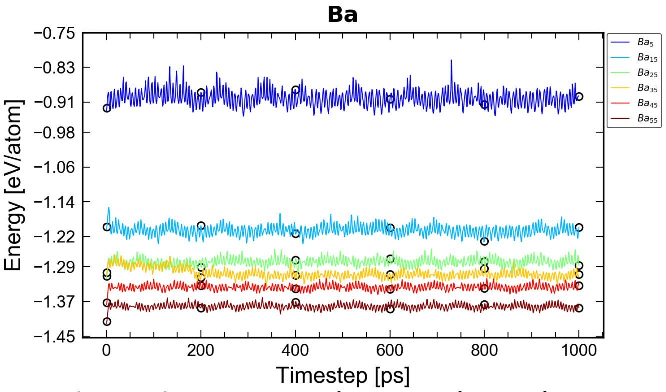

Supplementary Figure 163: The total energy as a function of time from exemplary MD simulations of Au nanoclusters. Snapshots of MD simulation trajectories are provided in Supplementary Figure 162

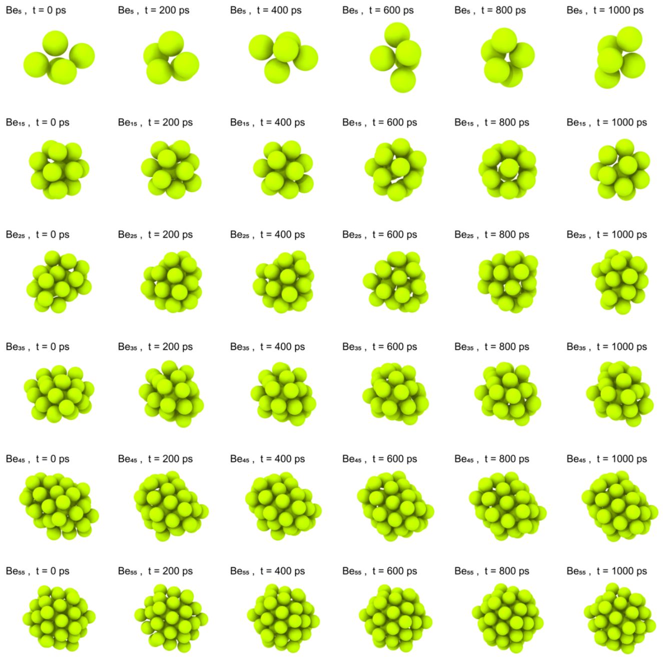

Supplementary Figure 164: MD simulation trajectories of Be Nanoclusters

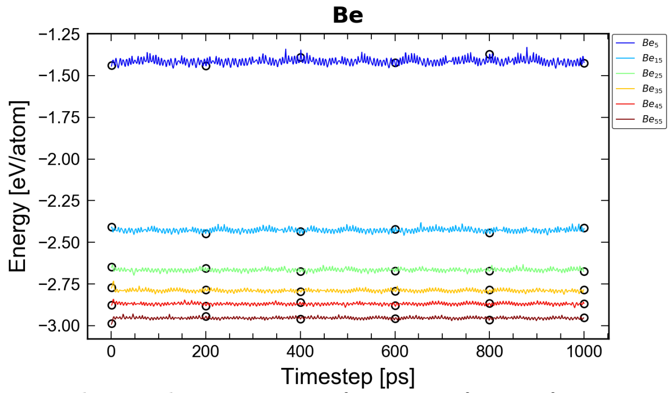

Supplementary Figure 165: The total energy as a function of time from exemplary MD simulations of Be nanoclusters. Snapshots of MD simulation trajectories are provided in Supplementary Figure 164

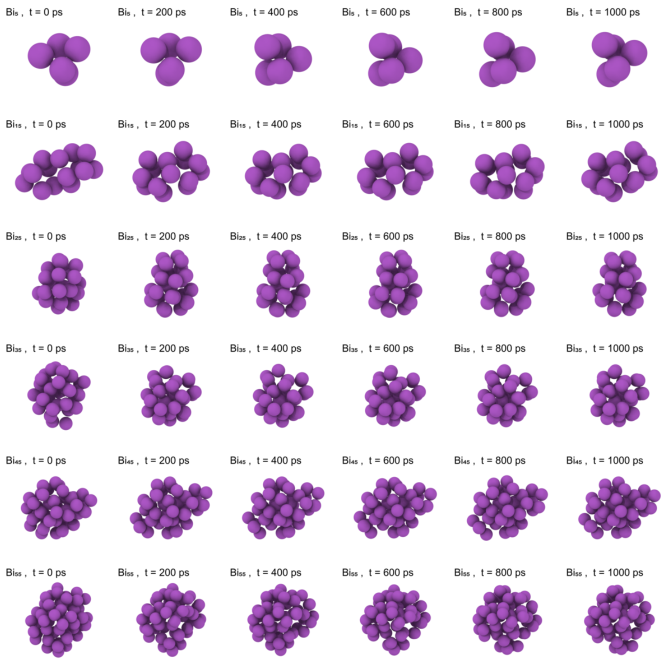

Supplementary Figure 166: MD simulation trajectories of Bi Nanoclusters

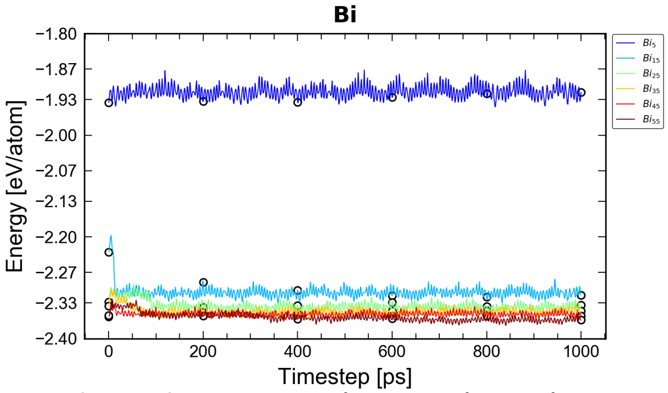

Supplementary Figure 167: The total energy as a function of time from exemplary MD simulations of Bi nanoclusters. Snapshots of MD simulation trajectories are provided in Supplementary Figure 166

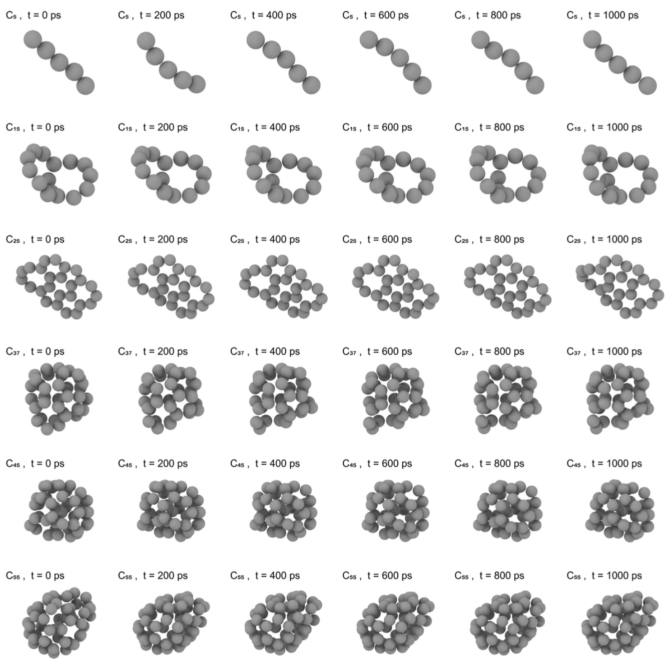

Supplementary Figure 168: MD simulation trajectories of C Nanoclusters

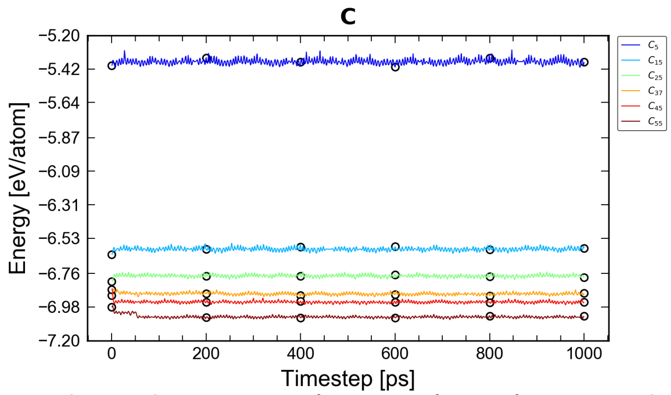

Supplementary Figure 169: The total energy as a function of time from exemplary MD simulations of C nanoclusters. Snapshots of MD simulation trajectories are provided in Supplementary Figure 168

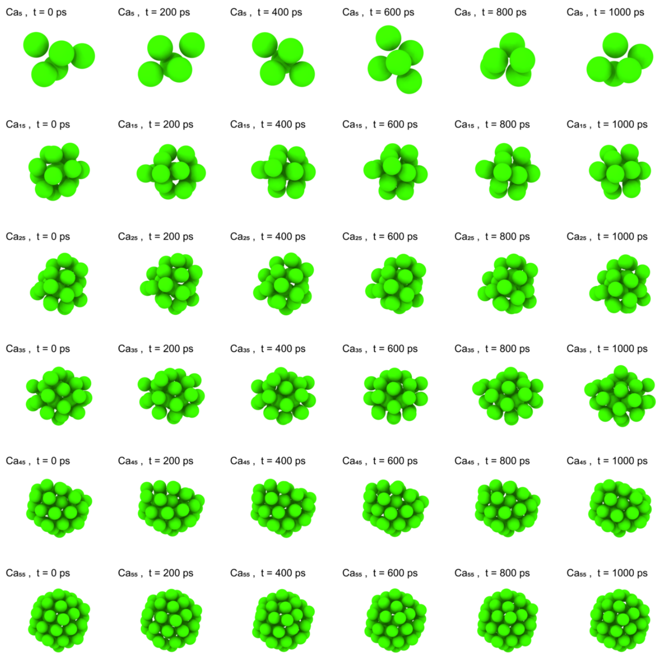

Supplementary Figure 170: MD simulation trajectories of Ca Nanoclusters

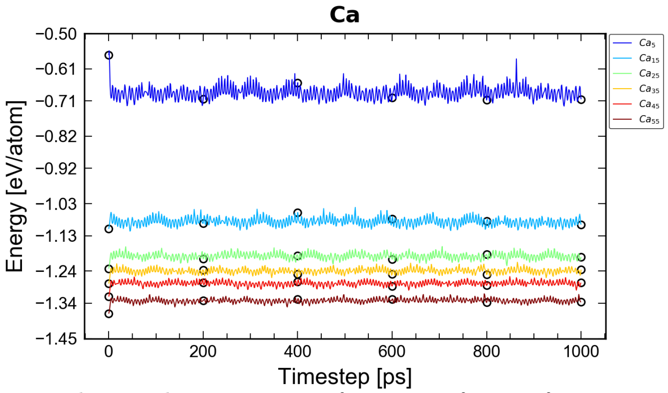

Supplementary Figure 171: The total energy as a function of time from exemplary MD simulations of Ca nanoclusters. Snapshots of MD simulation trajectories are provided in Supplementary Figure 170

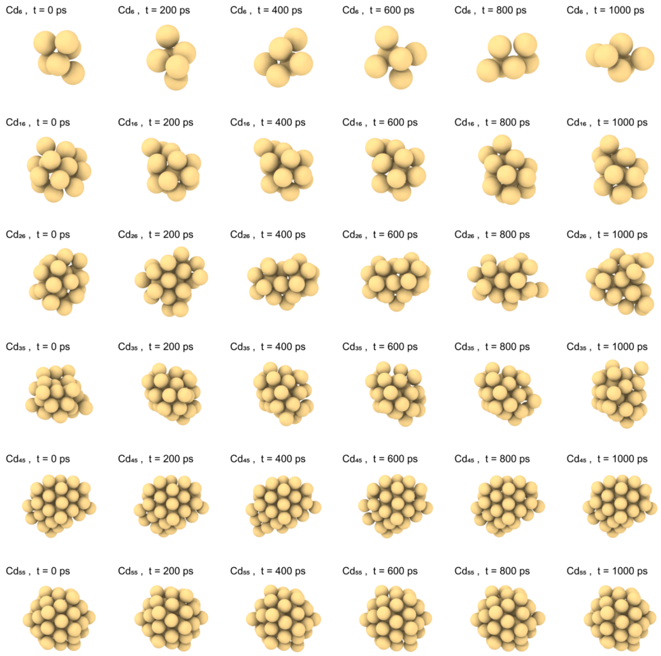

Supplementary Figure 172: MD simulation trajectories of Cd Nanoclusters

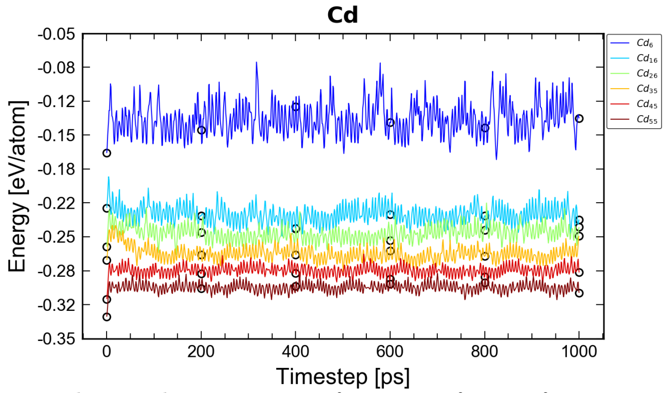

Supplementary Figure 173: The total energy as a function of time from exemplary MD simulations of Cd nanoclusters. Snapshots of MD simulation trajectories are provided in Supplementary Figure 172

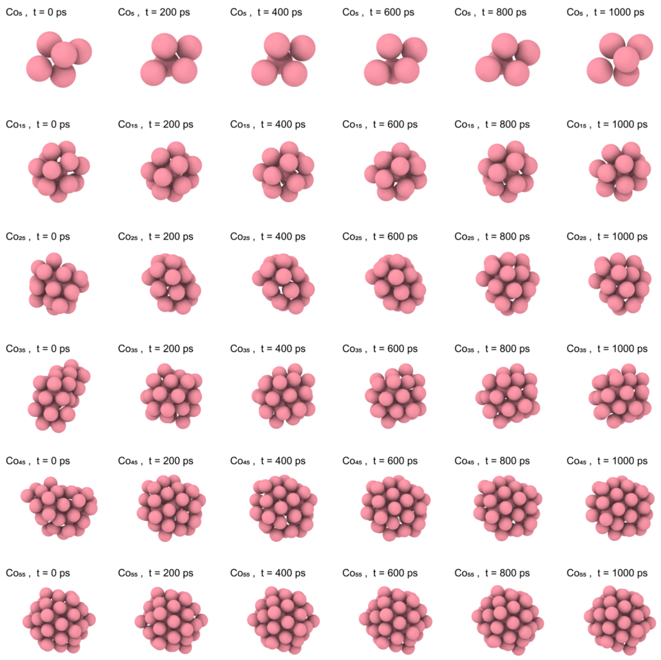

Supplementary Figure 174: MD simulation trajectories of Co Nanoclusters

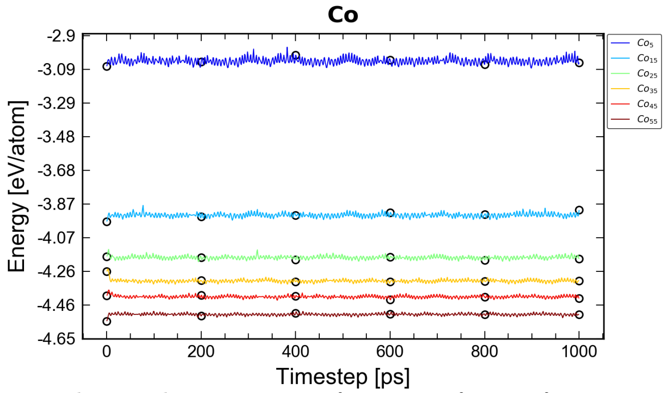

Supplementary Figure 175: The total energy as a function of time from exemplary MD simulations of Co nanoclusters. Snapshots of MD simulation trajectories are provided in Supplementary Figure 174

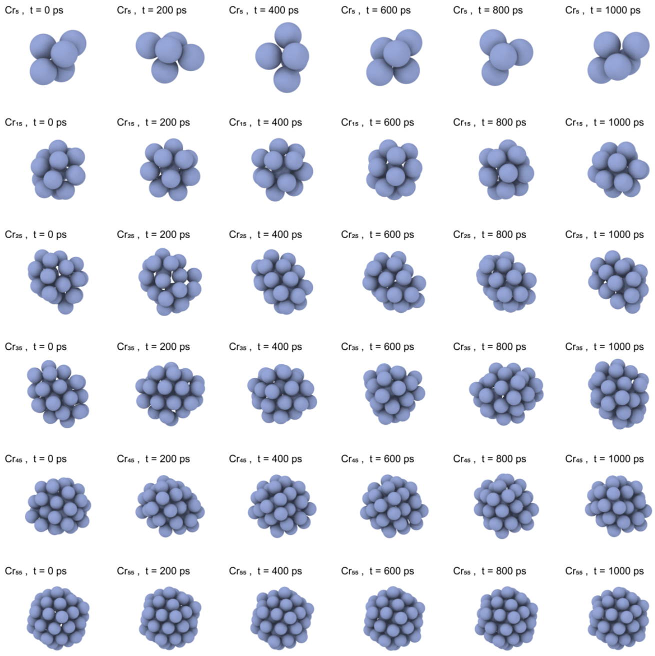

Supplementary Figure 176: MD simulation trajectories of Cr Nanoclusters

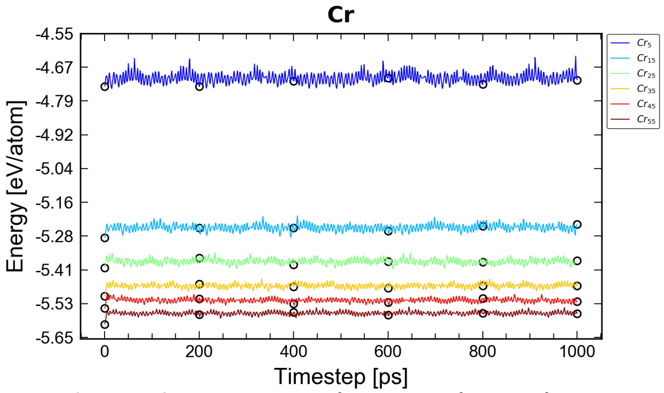

Supplementary Figure 177: The total energy as a function of time from exemplary MD simulations of Cr nanoclusters. Snapshots of MD simulation trajectories are provided in Supplementary Figure 176

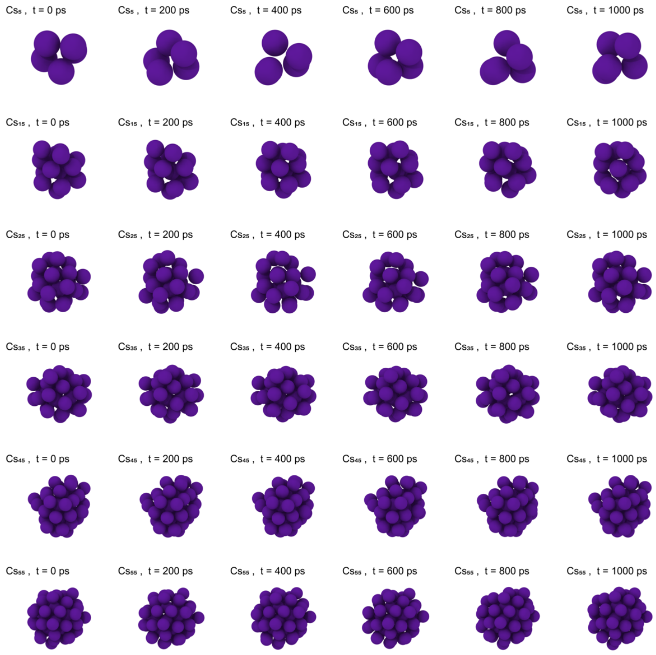

Supplementary Figure 178: MD simulation trajectories of Cs Nanoclusters

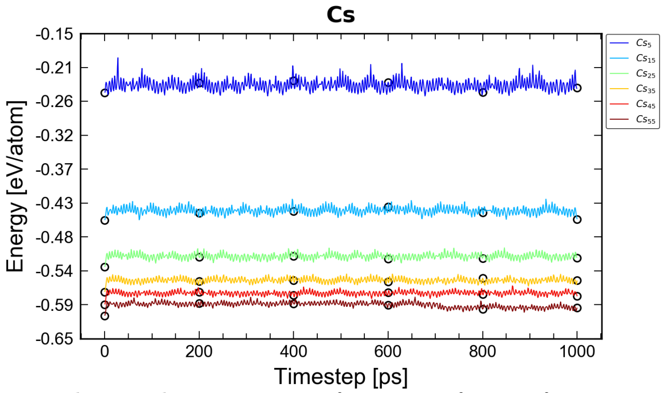

Supplementary Figure 179: The total energy as a function of time from exemplary MD simulations of Cs nanoclusters. Snapshots of MD simulation trajectories are provided in Supplementary Figure 178

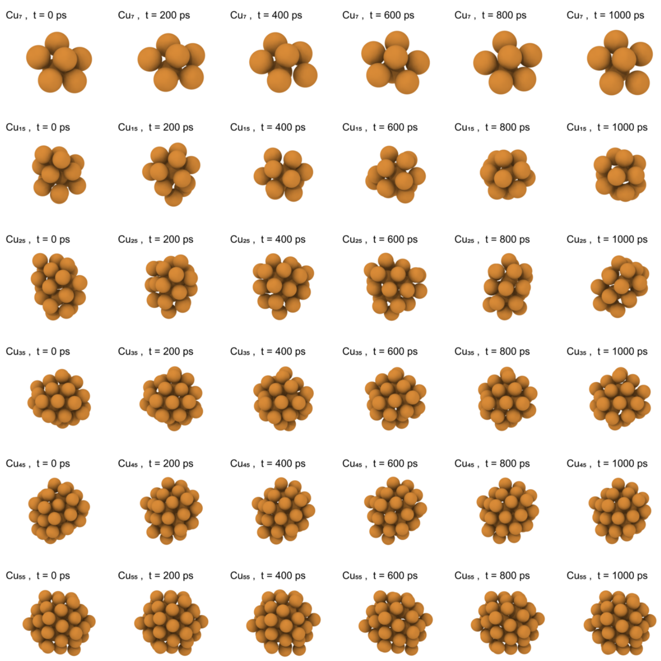

Supplementary Figure 180: MD simulation trajectories of Cu Nanoclusters

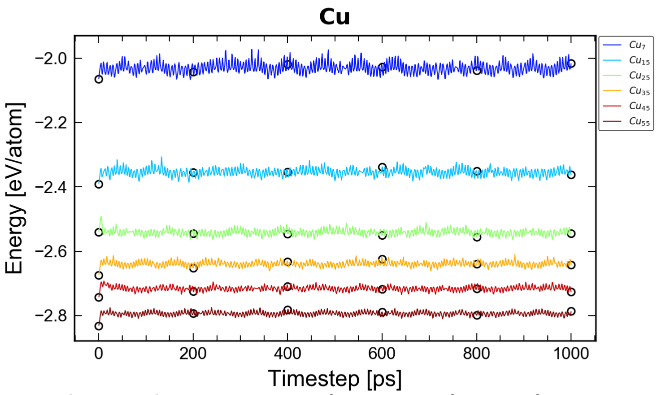

Supplementary Figure 181: The total energy as a function of time from exemplary MD simulations of Cu nanoclusters. Snapshots of MD simulation trajectories are provided in Supplementary Figure 180

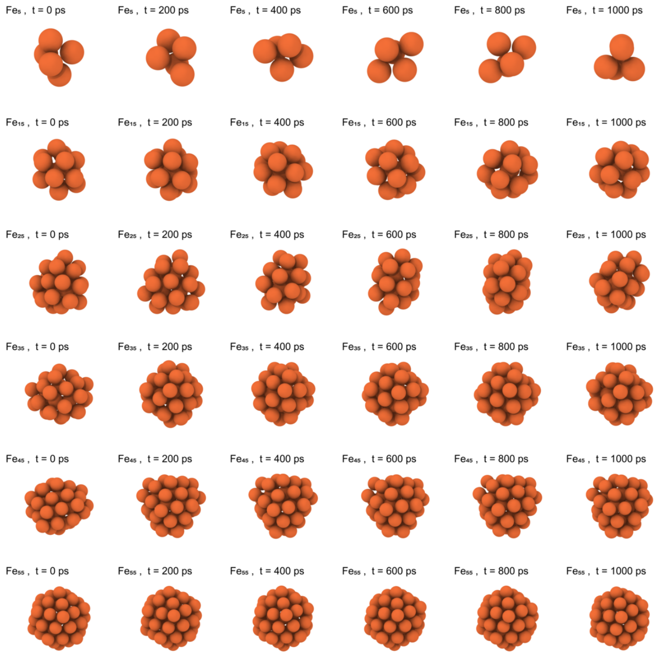

Supplementary Figure 182: MD simulation trajectories of Fe Nanoclusters

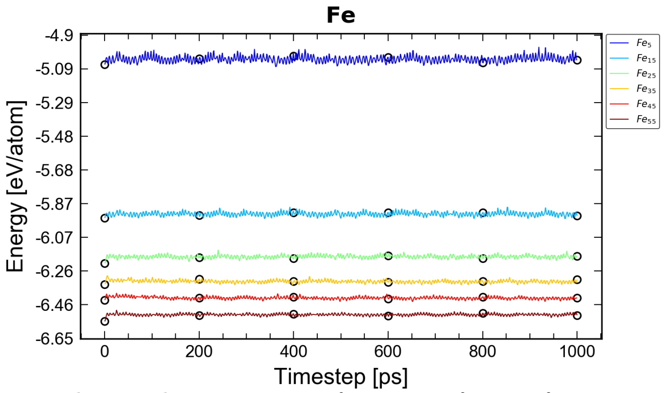

Supplementary Figure 183: The total energy as a function of time from exemplary MD simulations of Fe nanoclusters. Snapshots of MD simulation trajectories are provided in Supplementary Figure 182

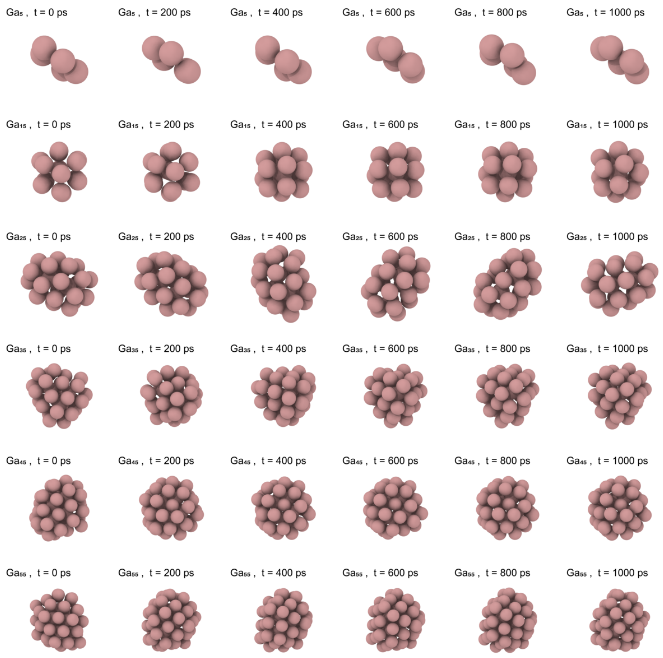

Supplementary Figure 184: MD simulation trajectories of Ga Nanoclusters

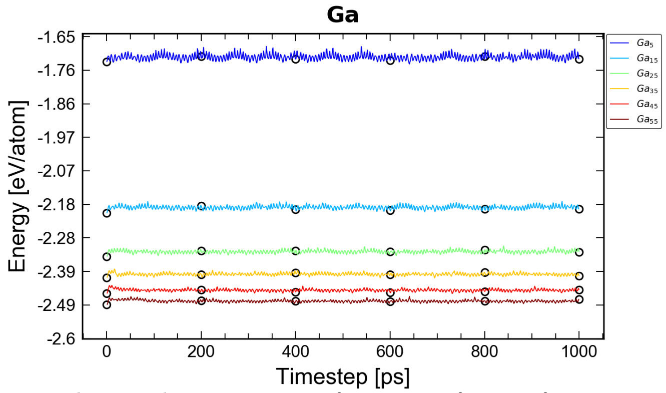

Supplementary Figure 185: The total energy as a function of time from exemplary MD simulations of Ga nanoclusters. Snapshots of MD simulation trajectories are provided in Supplementary Figure 184

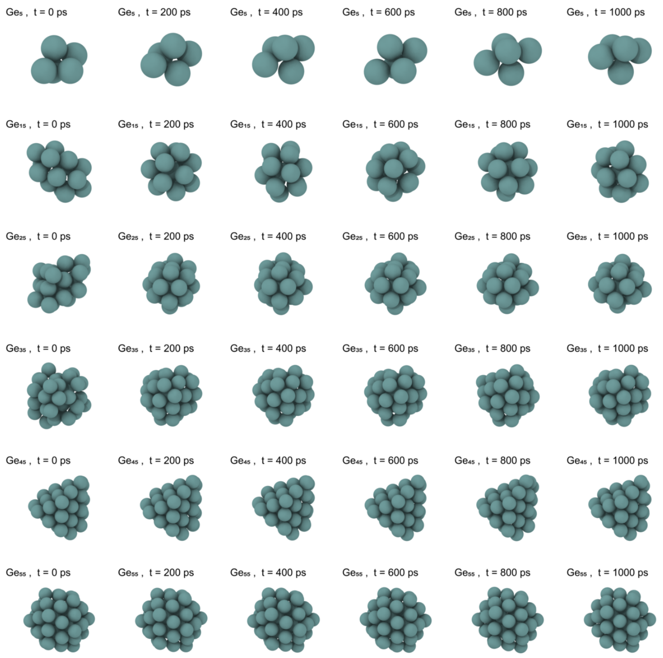

Supplementary Figure 186: MD simulation trajectories of Ge Nanoclusters

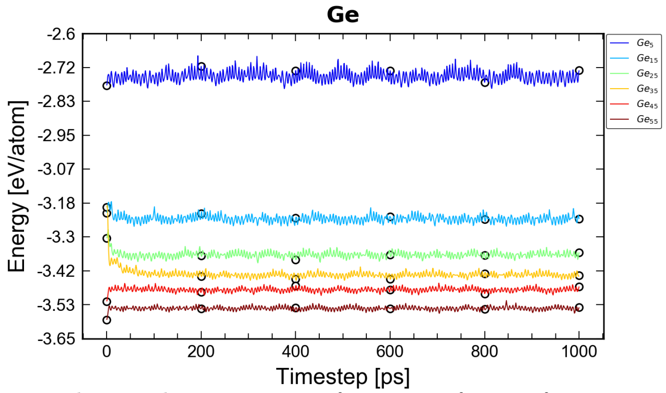

Supplementary Figure 187: The total energy as a function of time from exemplary MD simulations of Ge nanoclusters. Snapshots of MD simulation trajectories are provided in Supplementary Figure 186

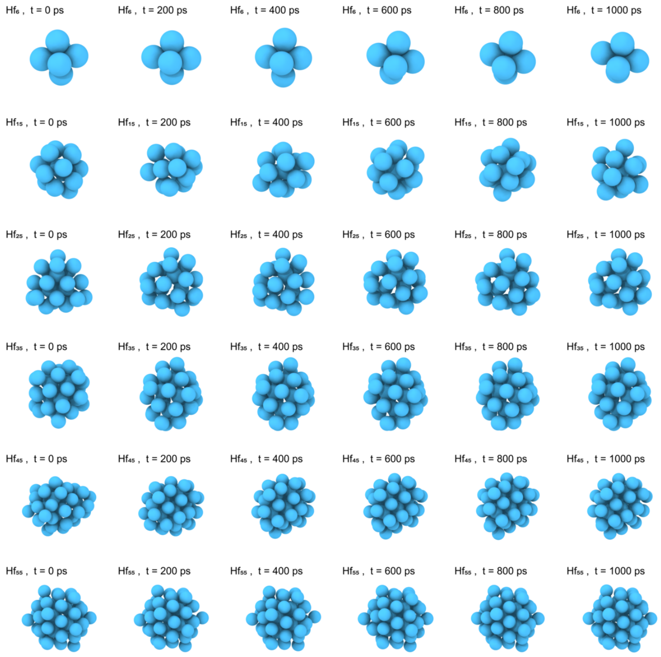

Supplementary Figure 188: MD simulation trajectories of Hf Nanoclusters

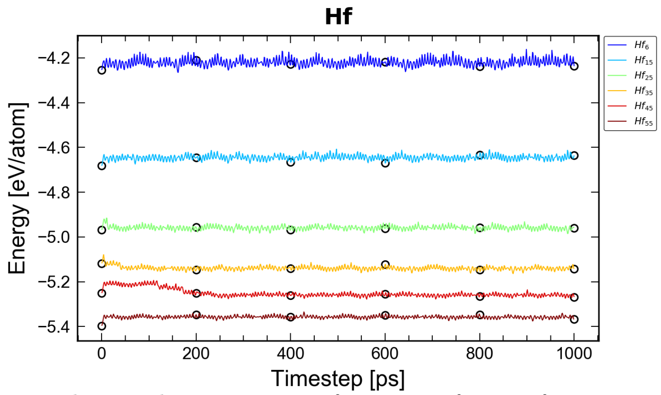

Supplementary Figure 189: The total energy as a function of time from exemplary MD simulations of Hf nanoclusters. Snapshots of MD simulation trajectories are provided in Supplementary Figure 188

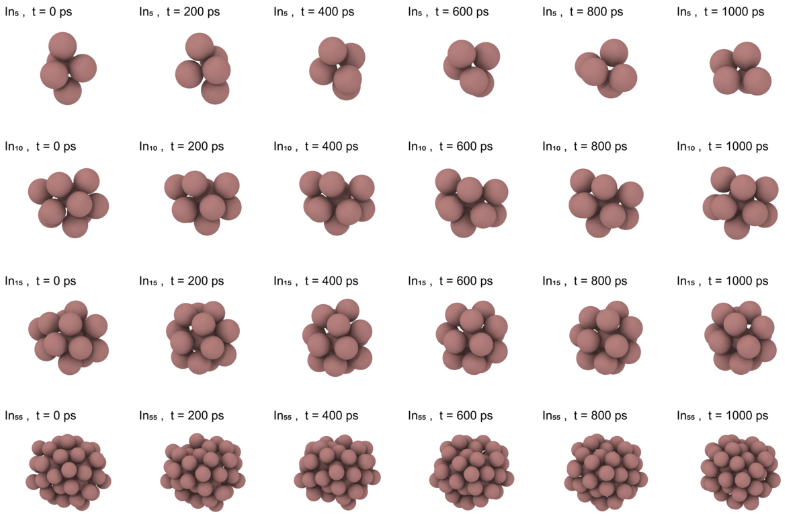

Supplementary Figure 190: MD simulation trajectories of In Nanoclusters

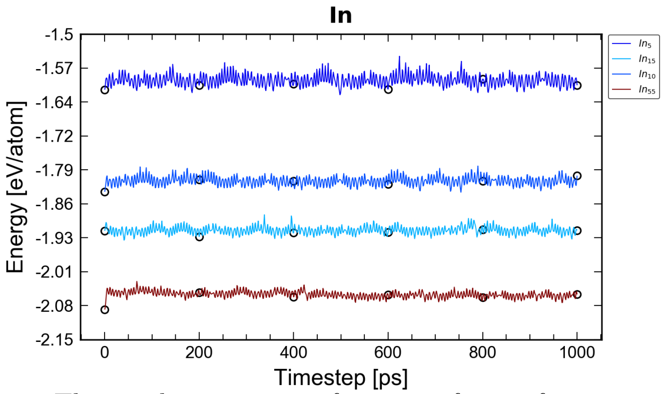

Supplementary Figure 191: The total energy as a function of time from exemplary MD simulations of Au nanoclusters. Snapshots of MD simulation trajectories are provided in Supplementary Figure 190

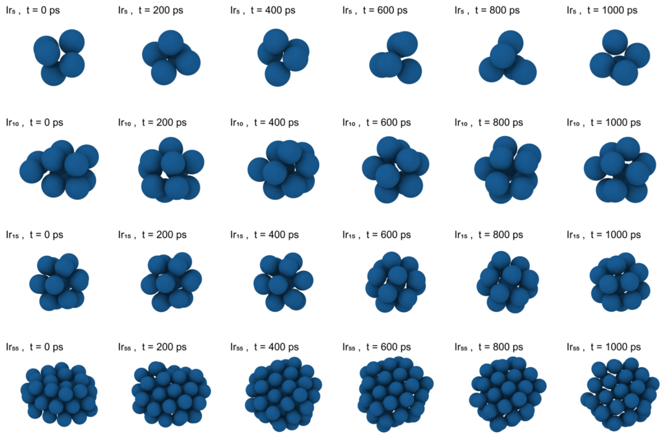

Supplementary Figure 192: MD simulation trajectories of Ir Nanoclusters

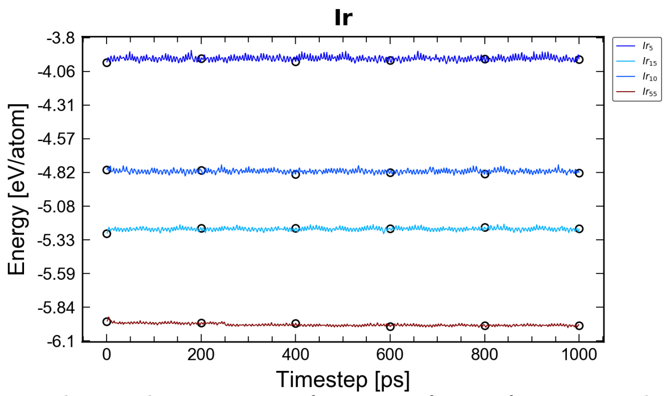

Supplementary Figure 193: The total energy as a function of time from exemplary MD simulations of Ir nanoclusters. Snapshots of MD simulation trajectories are provided in Supplementary Figure 192

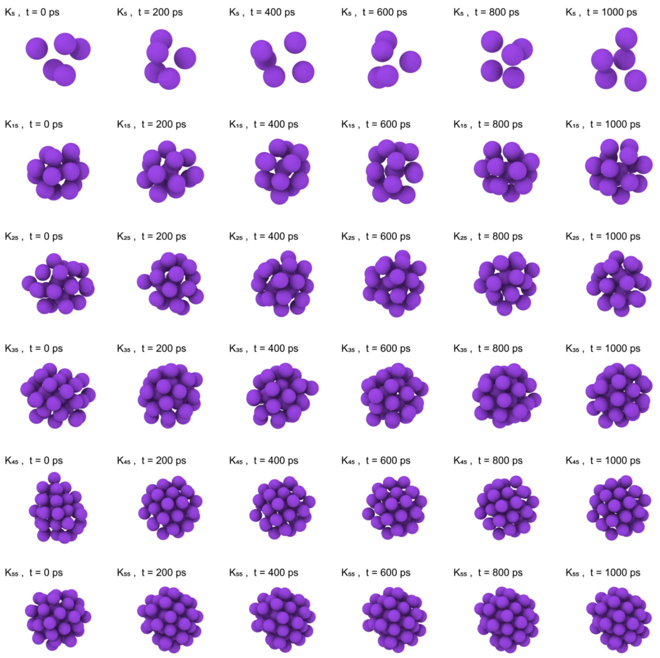

Supplementary Figure 194: MD simulation trajectories of K Nanoclusters

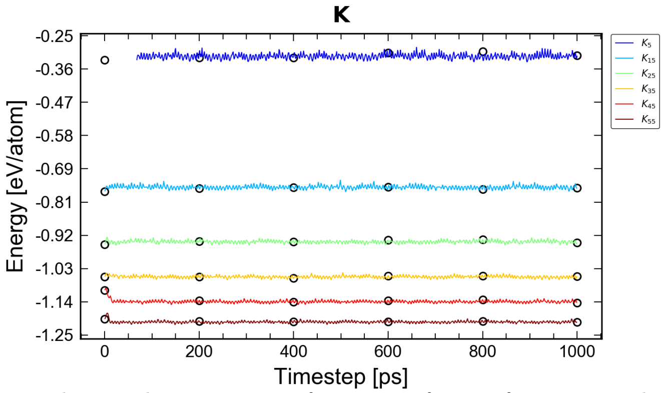

Supplementary Figure 195: The total energy as a function of time from exemplary MD simulations of K nanoclusters. Snapshots of MD simulation trajectories are provided in Supplementary Figure 194

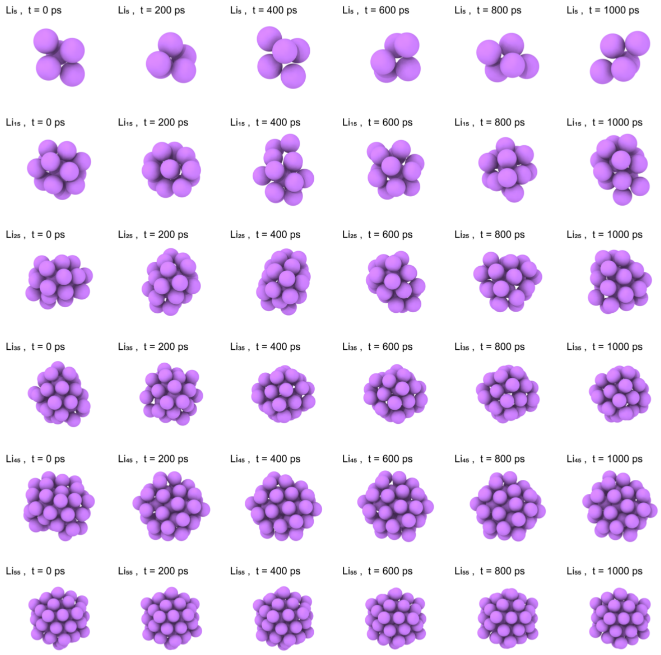

Supplementary Figure 196: MD simulation trajectories of Li Nanoclusters

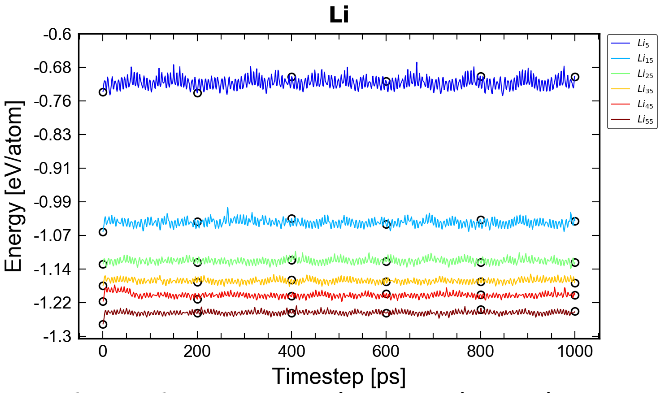

Supplementary Figure 197: The total energy as a function of time from exemplary MD simulations of Li nanoclusters. Snapshots of MD simulation trajectories are provided in Supplementary Figure 196

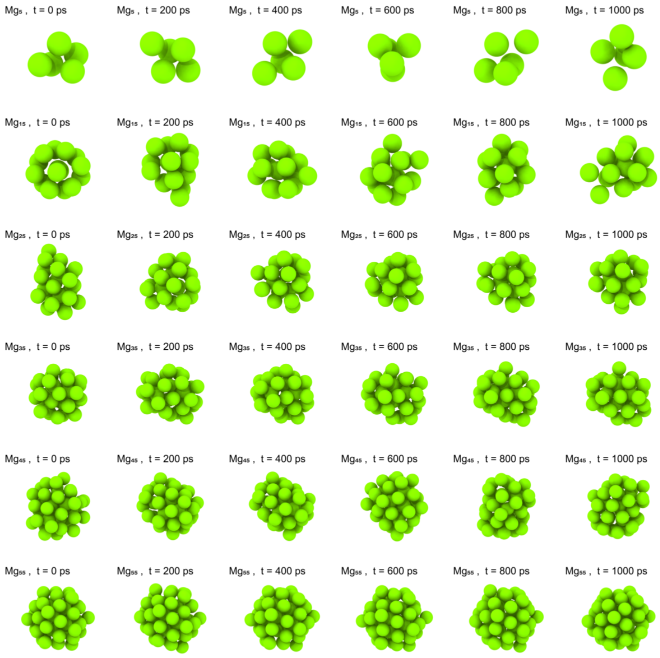

Supplementary Figure 198: MD simulation trajectories of Mg Nanoclusters

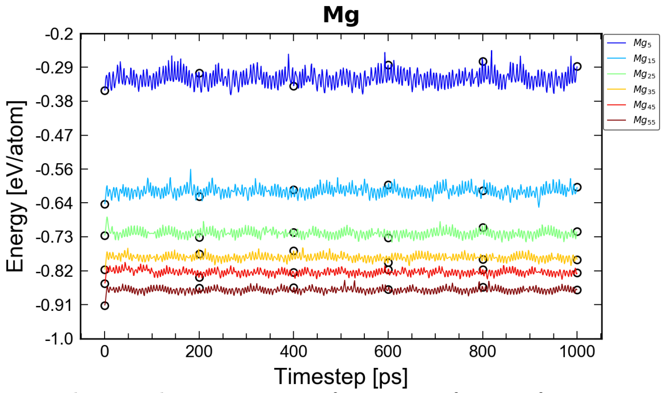

Supplementary Figure 199: The total energy as a function of time from exemplary MD simulations of Mg nanoclusters. Snapshots of MD simulation trajectories are provided in Supplementary Figure 198

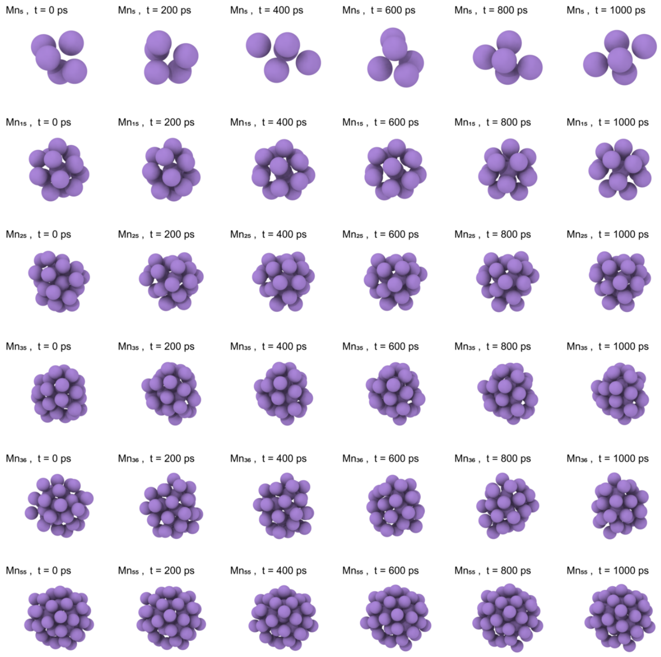

Supplementary Figure 200: MD simulation trajectories of Mn Nanoclusters

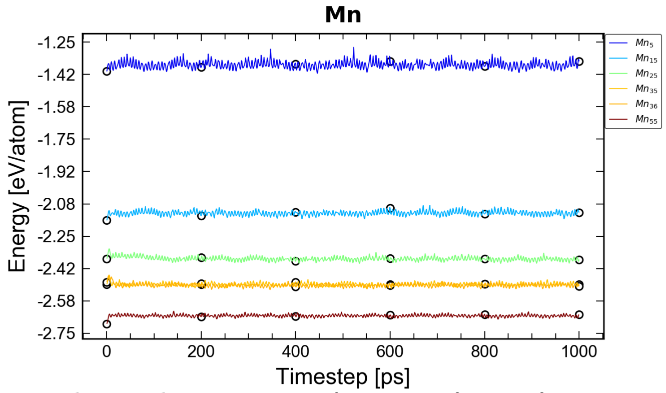

Supplementary Figure 201: The total energy as a function of time from exemplary MD simulations of Mn nanoclusters. Snapshots of MD simulation trajectories are provided in Supplementary Figure 200

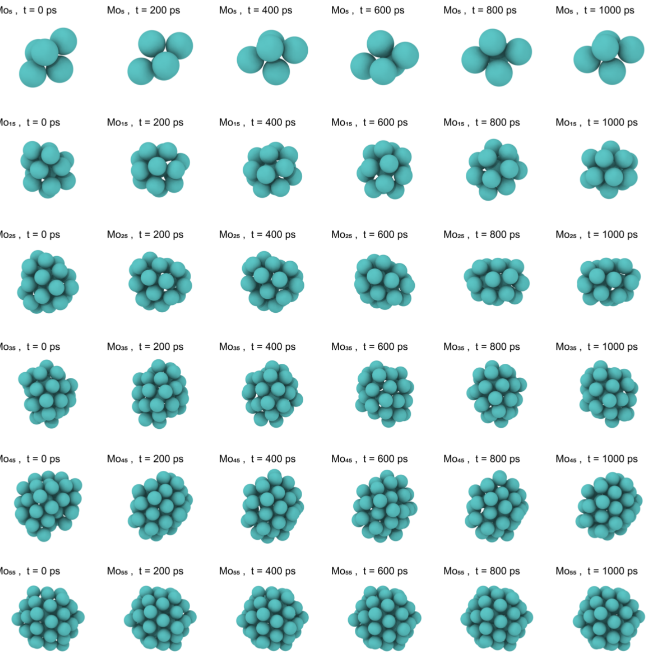

Supplementary Figure 202: MD simulation trajectories of Mo Nanoclusters

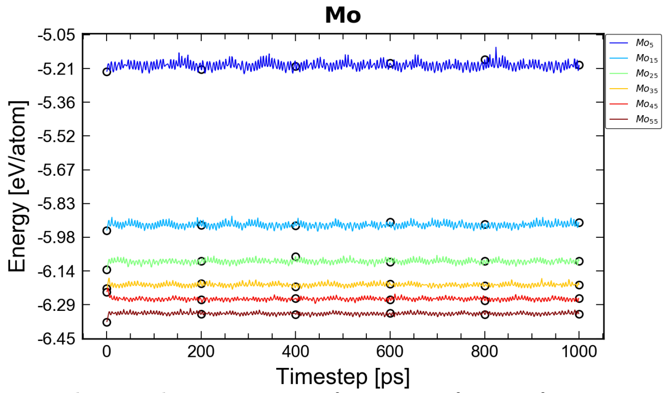

Supplementary Figure 203: The total energy as a function of time from exemplary MD simulations of Mo nanoclusters. Snapshots of MD simulation trajectories are provided in Supplementary Figure 202

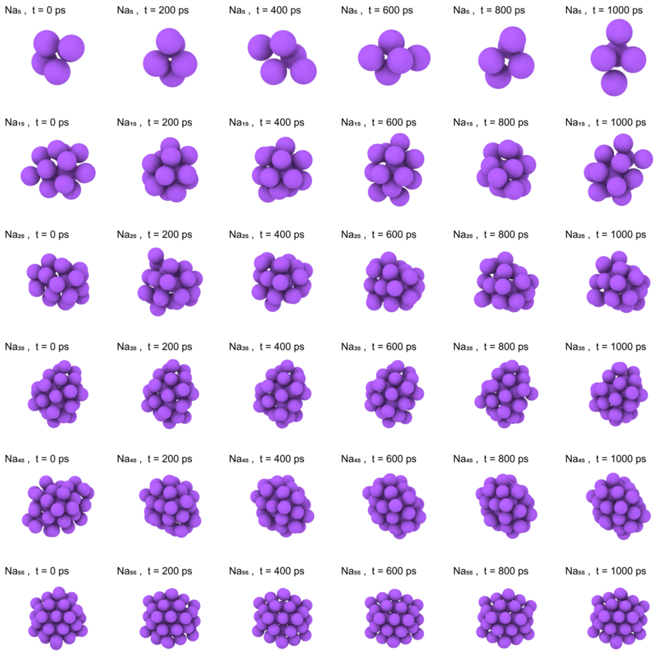

Supplementary Figure 204: MD simulation trajectories of Na Nanoclusters

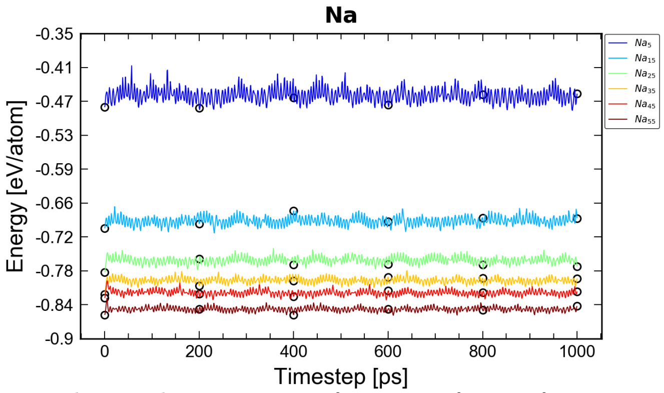

Supplementary Figure 205: The total energy as a function of time from exemplary MD simulations of Na nanoclusters. Snapshots of MD simulation trajectories are provided in Supplementary Figure 204

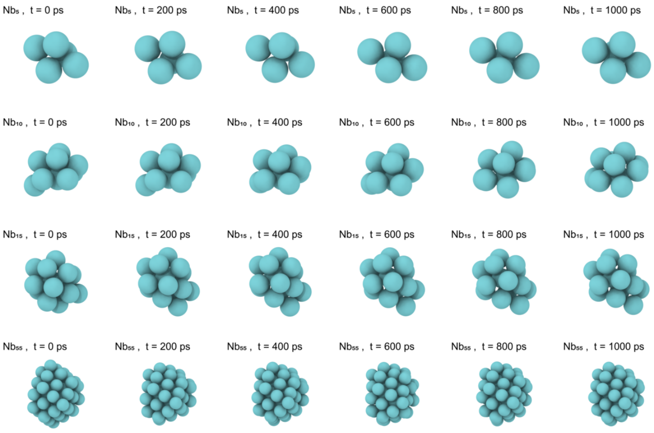

Supplementary Figure 206: MD simulation trajectories of Nb Nanoclusters

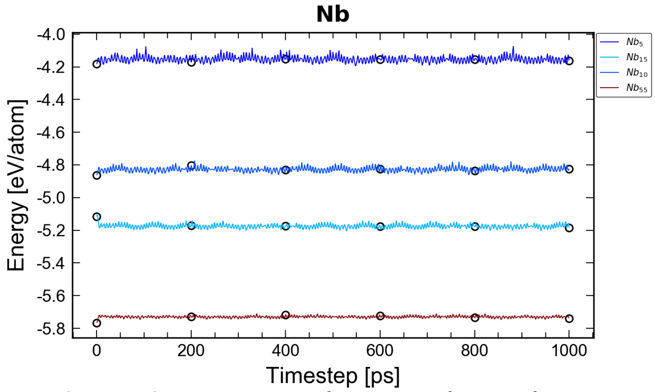

Supplementary Figure 207: The total energy as a function of time from exemplary MD simulations of Nb nanoclusters. Snapshots of MD simulation trajectories are provided in Supplementary Figure 206

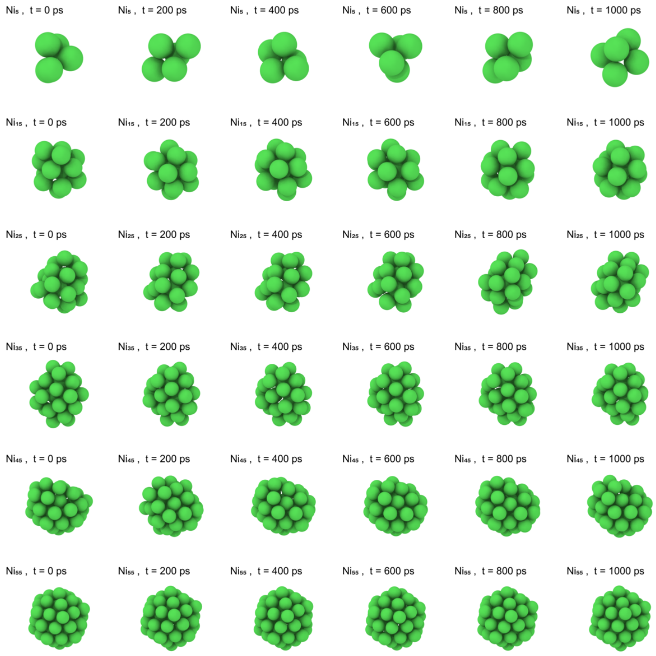

Supplementary Figure 208: MD simulation trajectories of Ni Nanoclusters

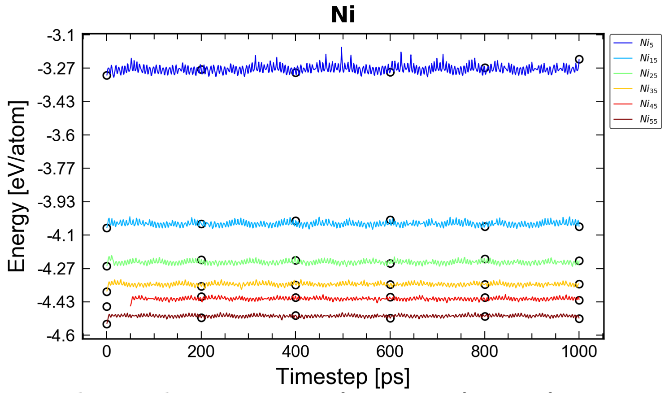

Supplementary Figure 209: The total energy as a function of time from exemplary MD simulations of Ni nanoclusters. Snapshots of MD simulation trajectories are provided in Supplementary Figure 208

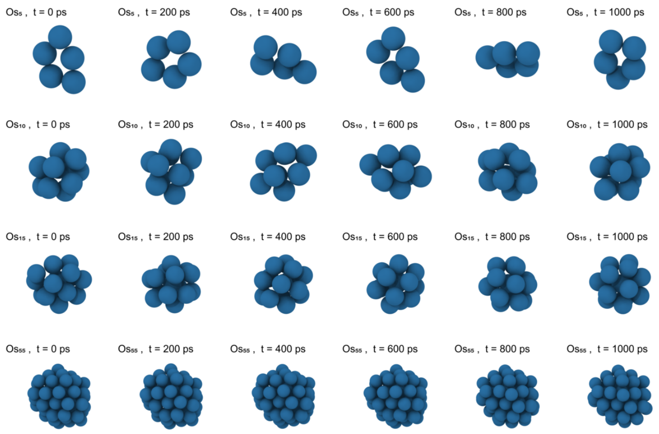

Supplementary Figure 210: MD simulation trajectories of Os Nanoclusters

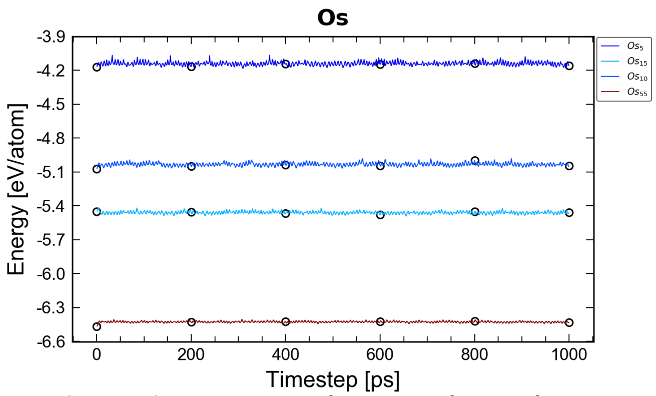

Supplementary Figure 211: The total energy as a function of time from exemplary MD simulations of Os nanoclusters. Snapshots of MD simulation trajectories are provided in Supplementary Figure 210

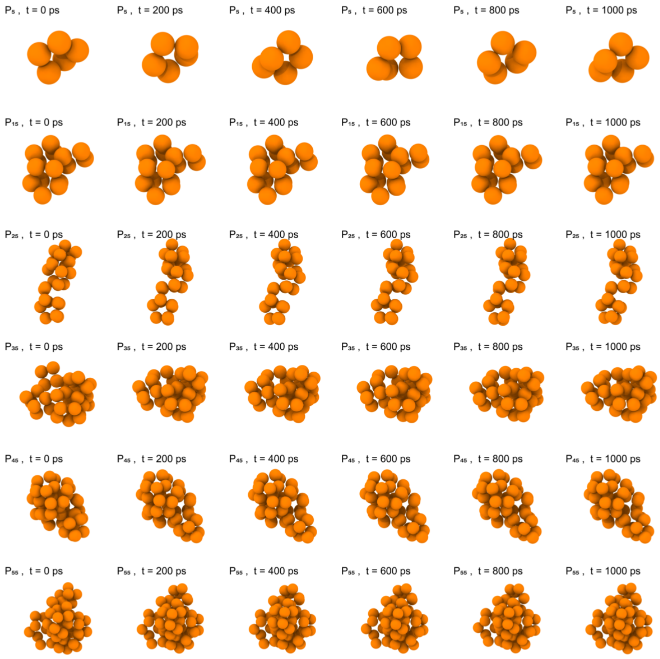

Supplementary Figure 212: MD simulation trajectories of P Nanoclusters

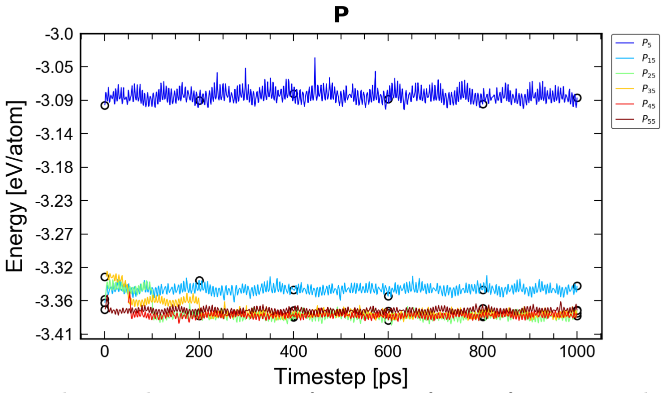

Supplementary Figure 213: The total energy as a function of time from exemplary MD simulations of P nanoclusters. Snapshots of MD simulation trajectories are provided in Supplementary Figure 212

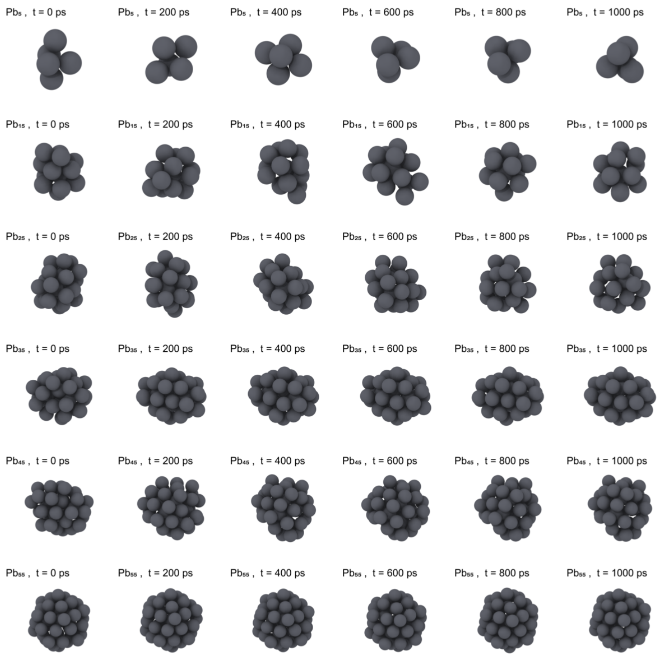

Supplementary Figure 214: MD simulation trajectories of Pb Nanoclusters

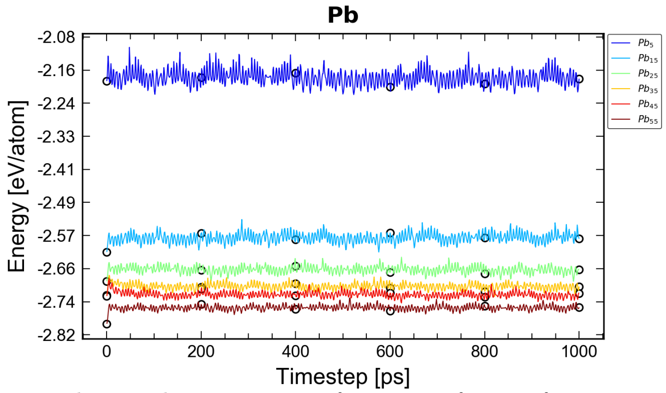

Supplementary Figure 215: The total energy as a function of time from exemplary MD simulations of Pb nanoclusters. Snapshots of MD simulation trajectories are provided in Supplementary Figure 214

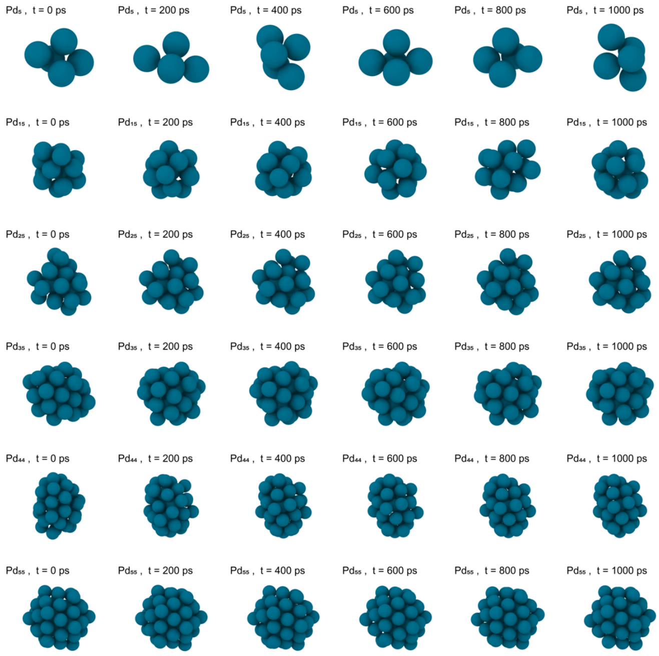

Supplementary Figure 216: MD simulation trajectories of Pd Nanoclusters

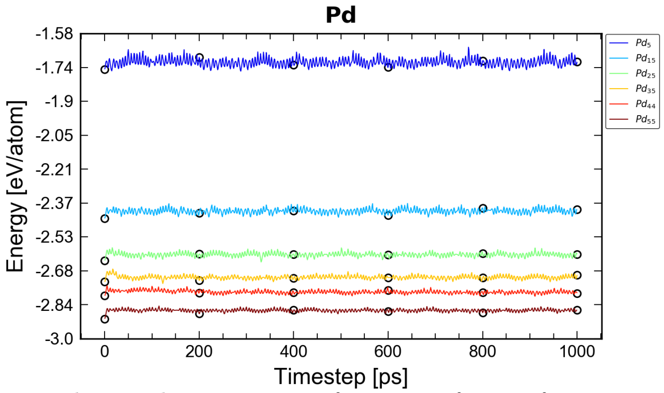

Supplementary Figure 217: The total energy as a function of time from exemplary MD simulations of Pd nanoclusters. Snapshots of MD simulation trajectories are provided in Supplementary Figure 216

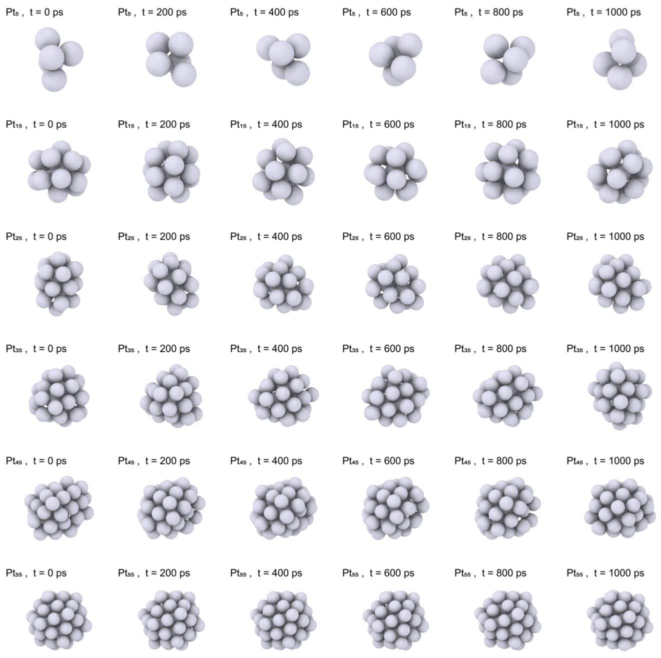

Supplementary Figure 218: MD simulation trajectories of Pt Nanoclusters

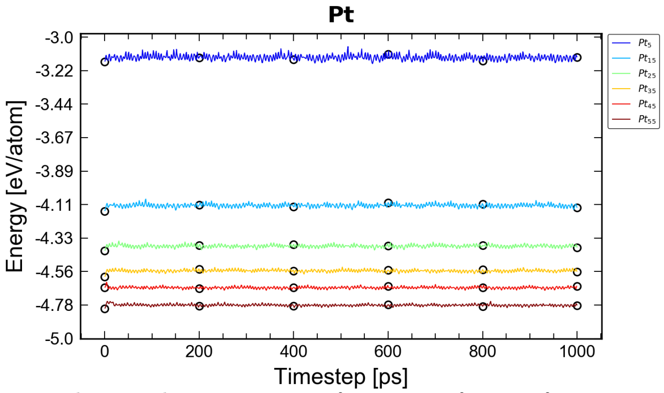

Supplementary Figure 219: The total energy as a function of time from exemplary MD simulations of Pt nanoclusters. Snapshots of MD simulation trajectories are provided in Supplementary Figure 218

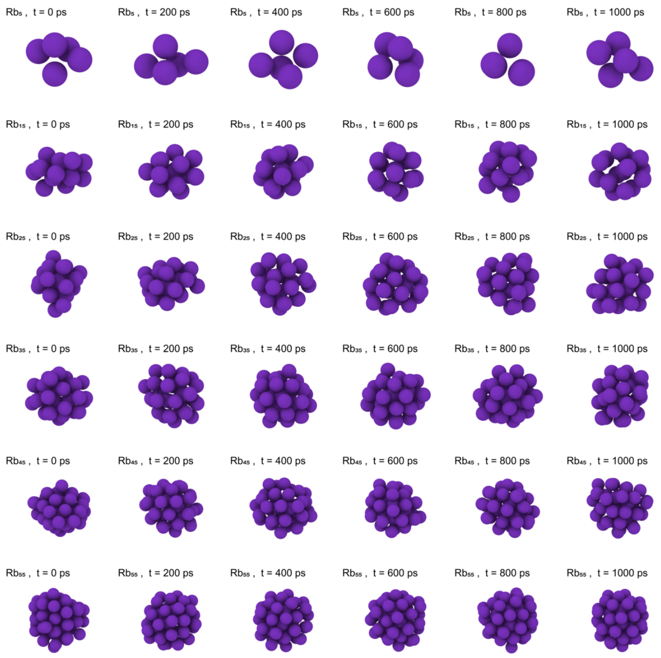

Supplementary Figure 220: MD simulation trajectories of Rb Nanoclusters

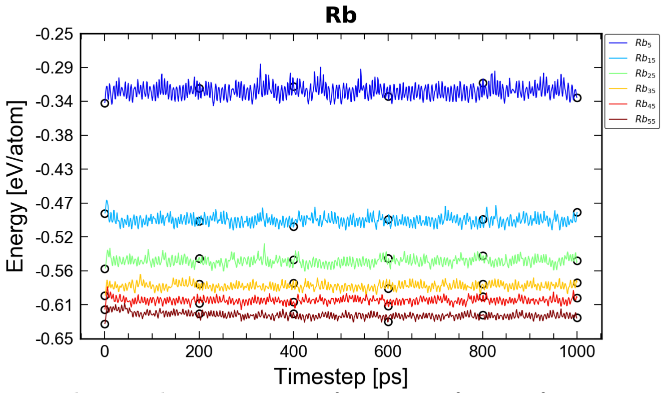

Supplementary Figure 221: The total energy as a function of time from exemplary MD simulations of Rb nanoclusters. Snapshots of MD simulation trajectories are provided in Supplementary Figure 220

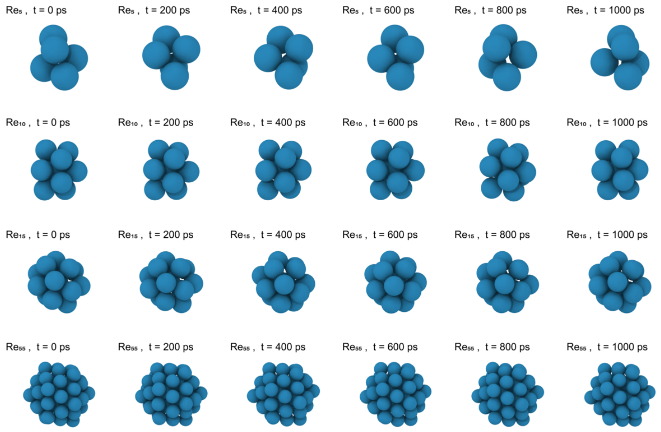

Supplementary Figure 222: MD simulation trajectories of Re Nanoclusters

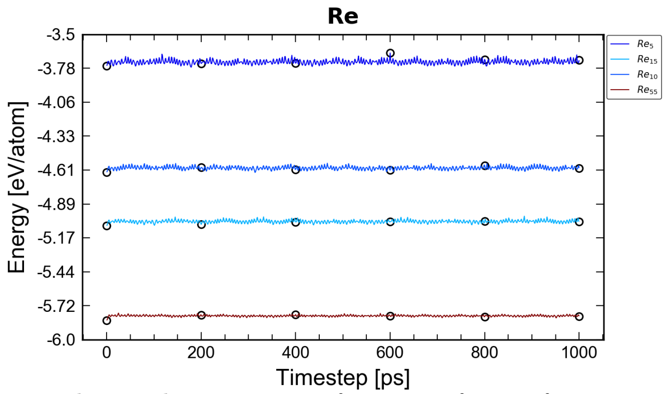

Supplementary Figure 223: The total energy as a function of time from exemplary MD simulations of Re nanoclusters. Snapshots of MD simulation trajectories are provided in Supplementary Figure 222

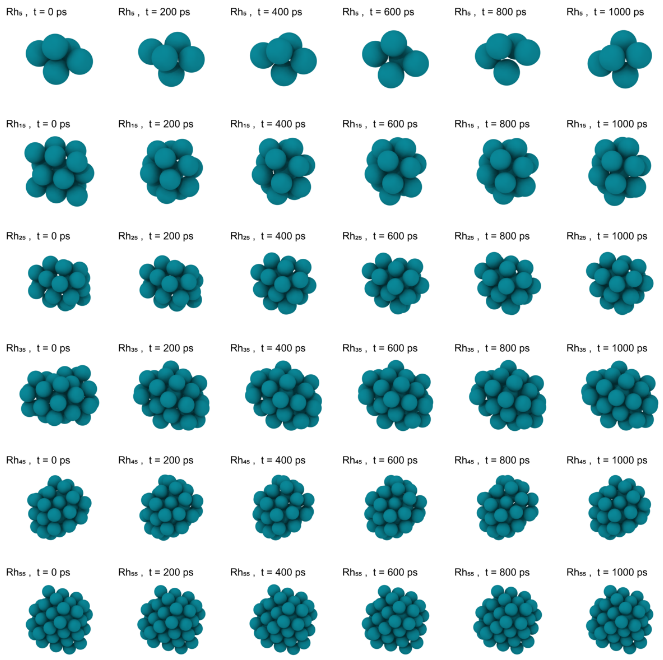

Supplementary Figure 224: MD simulation trajectories of Rh Nanoclusters

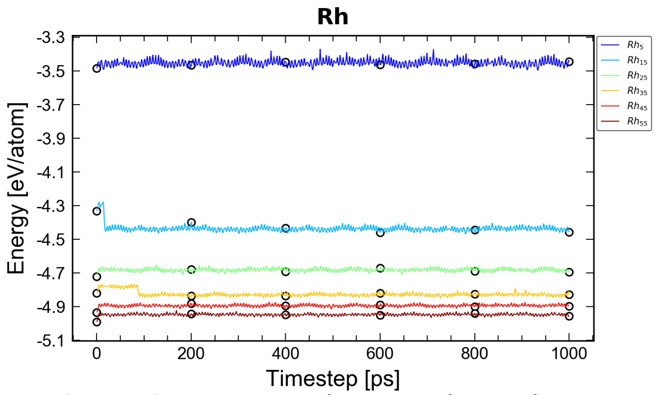

Supplementary Figure 225: The total energy as a function of time from exemplary MD simulations of Rh nanoclusters. Snapshots of MD simulation trajectories are provided in Supplementary Figure 224

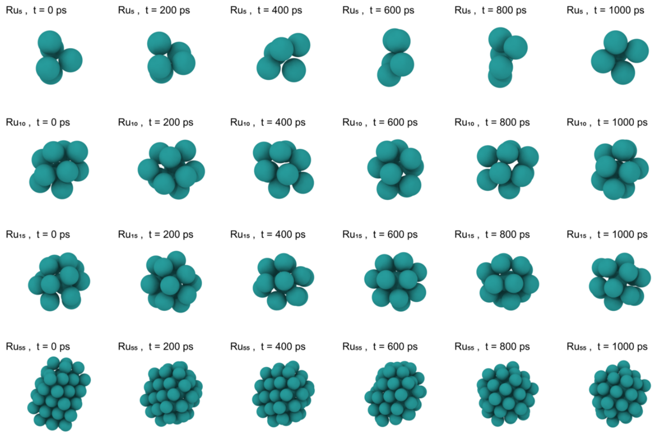

Supplementary Figure 226: MD simulation trajectories of Ru Nanoclusters

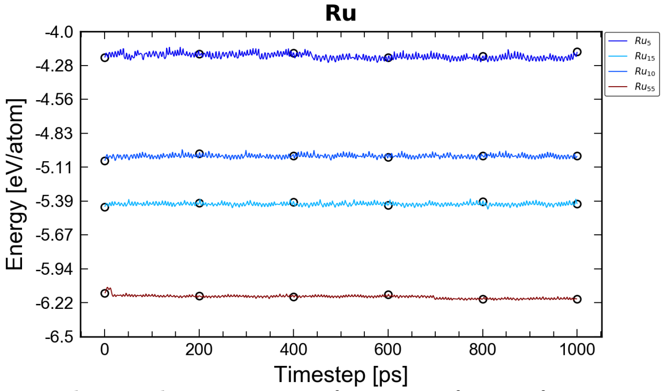

Supplementary Figure 227: The total energy as a function of time from exemplary MD simulations of Ru nanoclusters. Snapshots of MD simulation trajectories are provided in Supplementary Figure 226

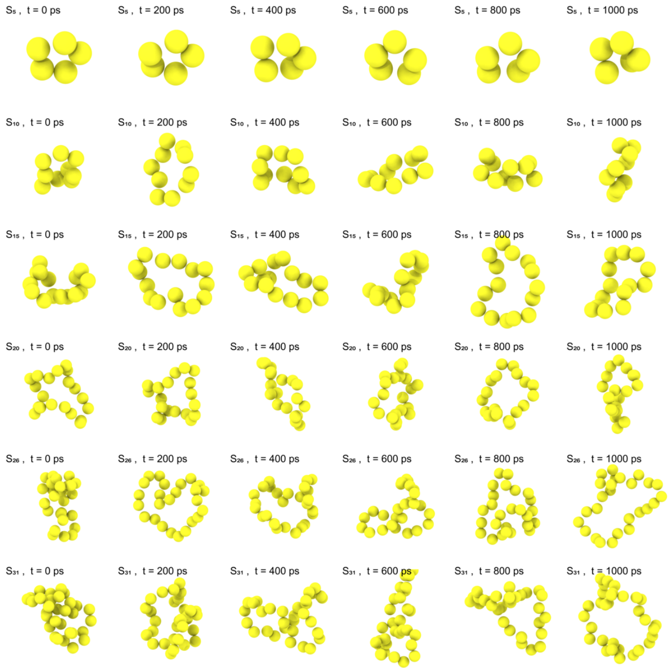

Supplementary Figure 228: MD simulation trajectories of S Nanoclusters

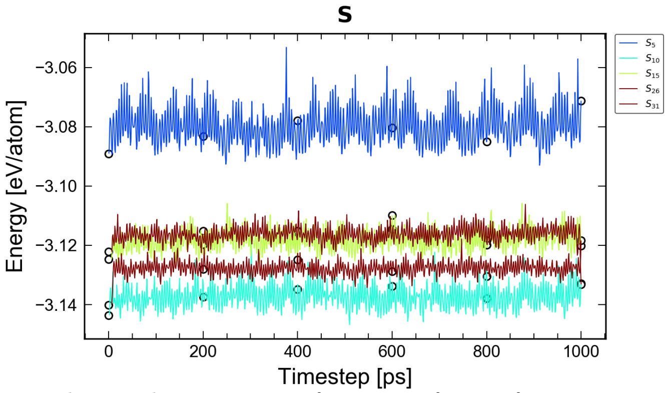

Supplementary Figure 229: The total energy as a function of time from an MD simulation of S nanoclusters. Snapshots of MD simulation trajectories are provided in Supplementary Figure 228

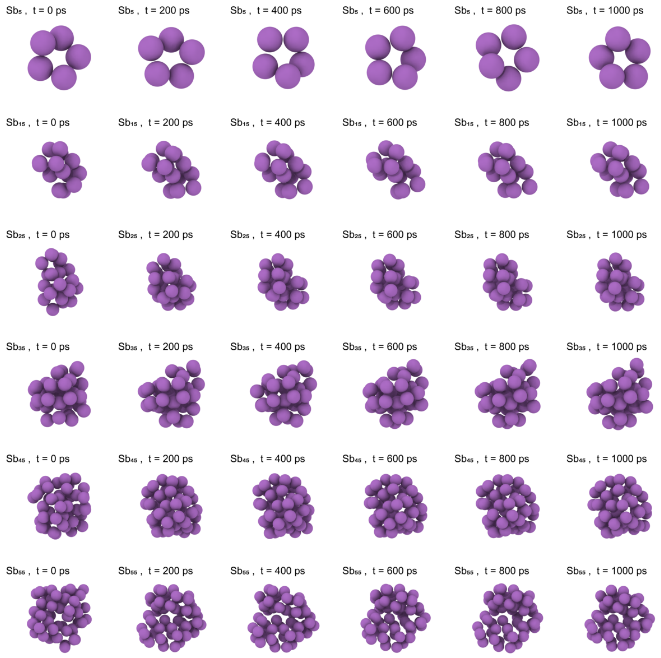

Supplementary Figure 230: MD simulation trajectories of Sb Nanoclusters

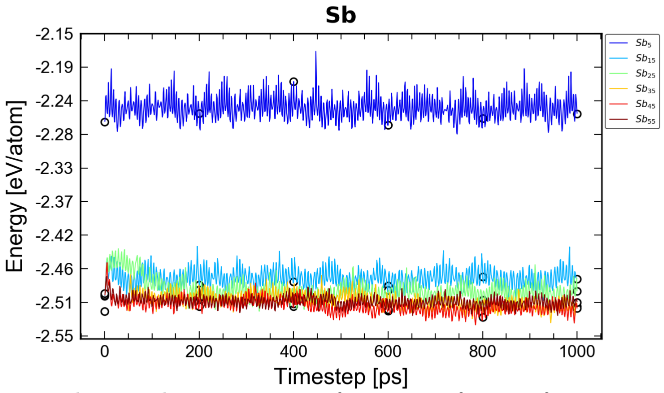

Supplementary Figure 231: The total energy as a function of time from exemplary MD simulations of Sb nanoclusters. Snapshots of MD simulation trajectories are provided in Supplementary Figure 230

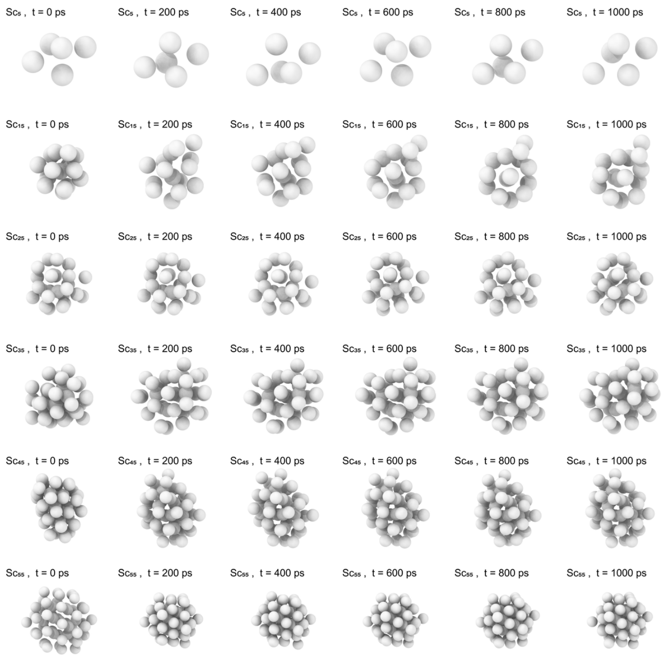

Supplementary Figure 232: MD simulation trajectories of Sc Nanoclusters

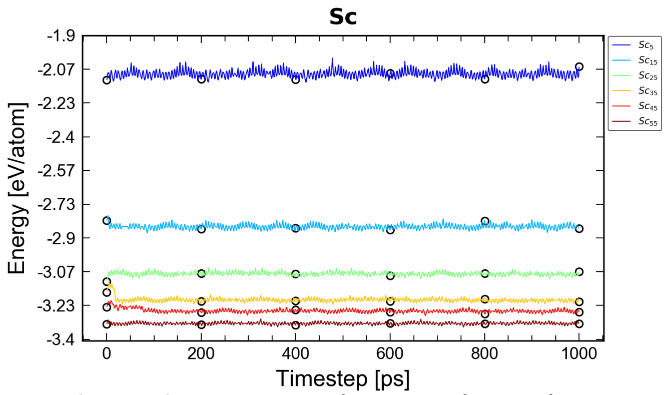

Supplementary Figure 233: The total energy as a function of time from exemplary MD simulations of Sc nanoclusters. Snapshots of MD simulation trajectories are provided in Supplementary Figure 232

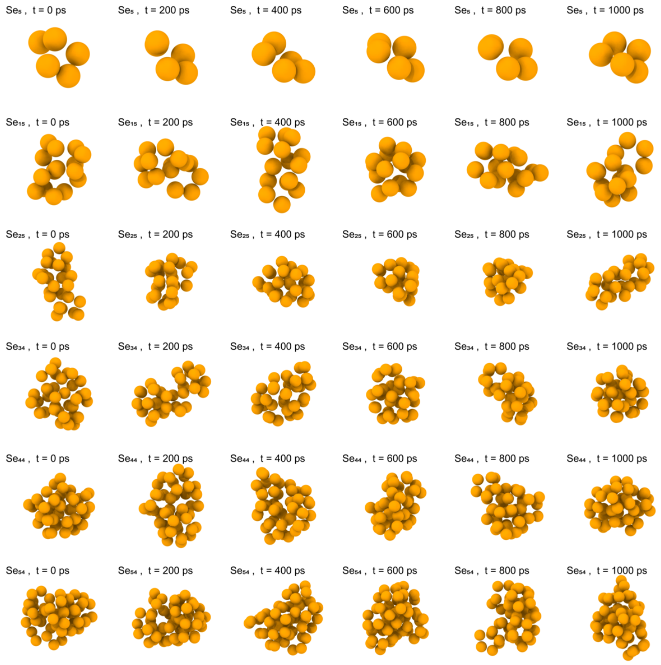

Supplementary Figure 234: MD simulation trajectories of Se Nanoclusters

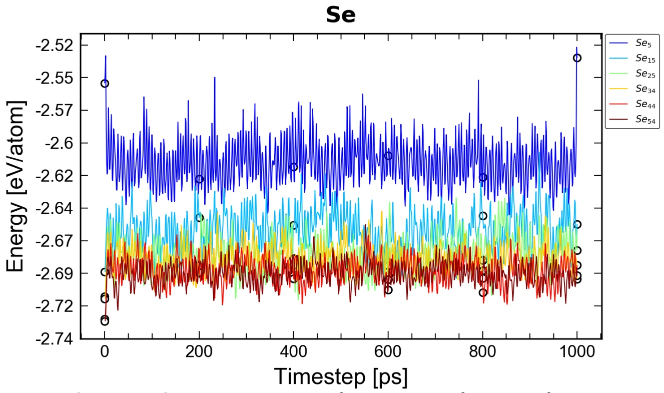

Supplementary Figure 235: The total energy as a function of time from exemplary MD simulations of Se nanoclusters. Snapshots of MD simulation trajectories are provided in Supplementary Figure 234

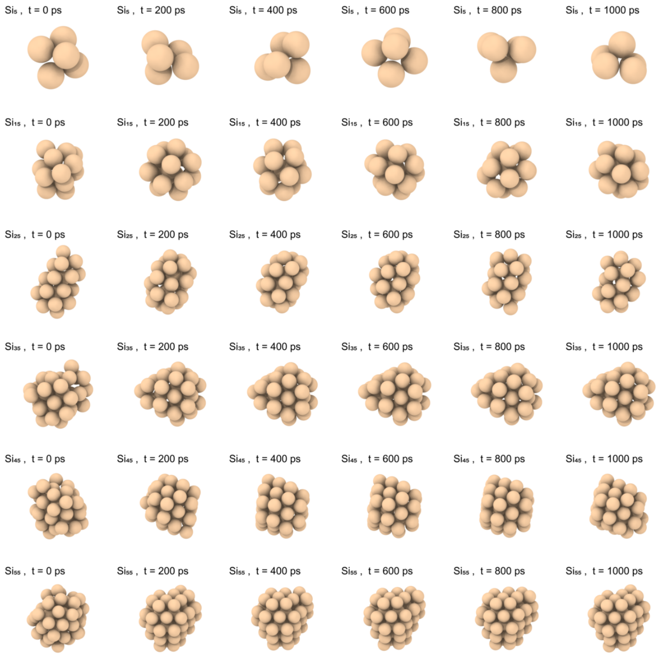

Supplementary Figure 236: MD simulation trajectories of Si Nanoclusters

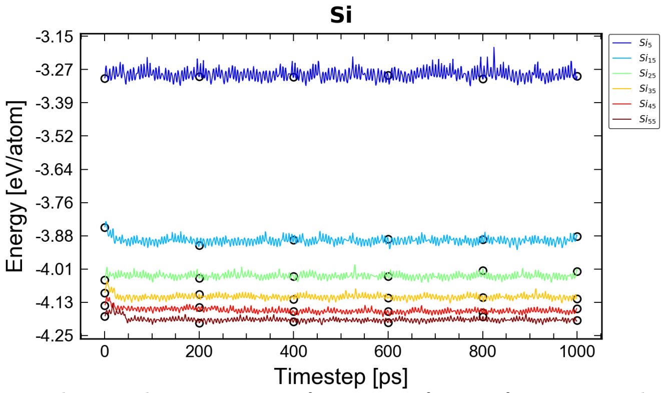

Supplementary Figure 237: The total energy as a function of time from exemplary MD simulations of Si nanoclusters. Snapshots of MD simulation trajectories are provided in Supplementary Figure 236

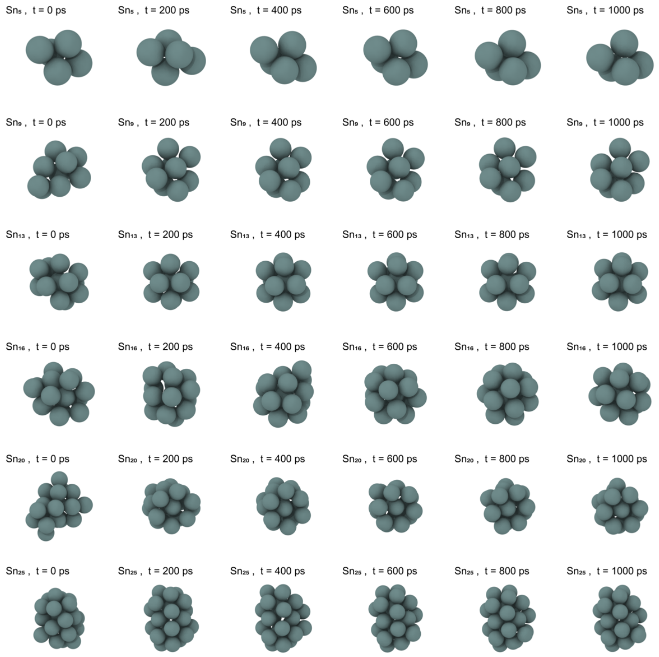

Supplementary Figure 238: MD simulation trajectories of Sn Nanoclusters

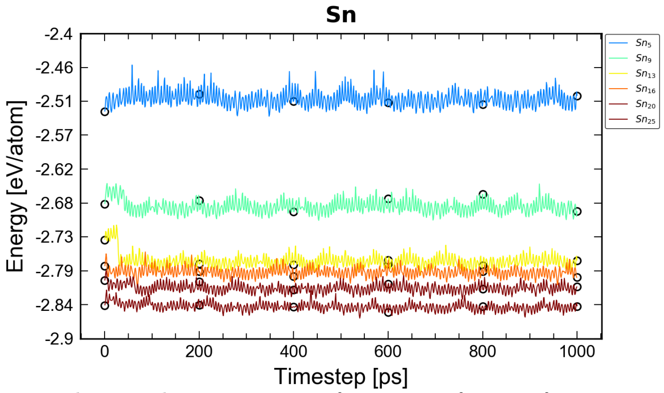

Supplementary Figure 239: The total energy as a function of time from exemplary MD simulations of Sn nanoclusters. Snapshots of MD simulation trajectories are provided in Supplementary Figure 238

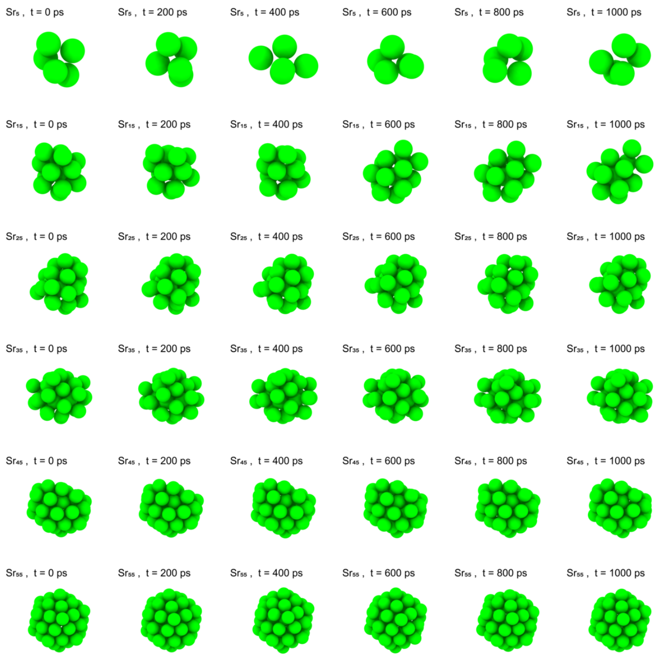

Supplementary Figure 240: MD simulation trajectories of Sr Nanoclusters

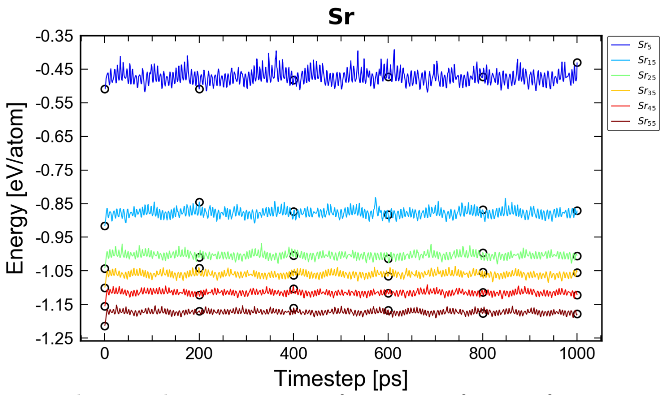

Supplementary Figure 241: The total energy as a function of time from exemplary MD simulations of Sr nanoclusters. Snapshots of MD simulation trajectories are provided in Supplementary Figure 240

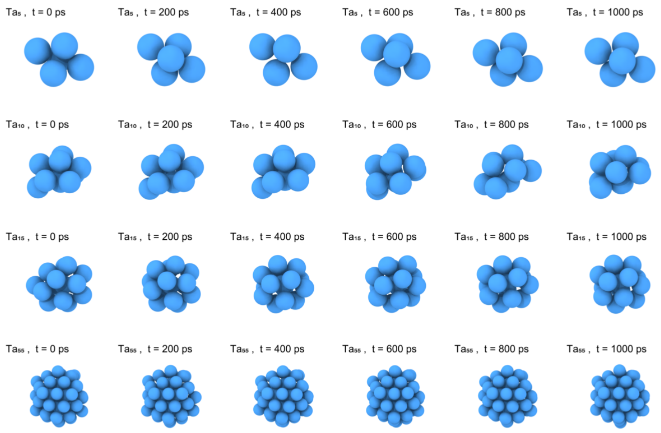

Supplementary Figure 242: MD simulation trajectories of Ta Nanoclusters

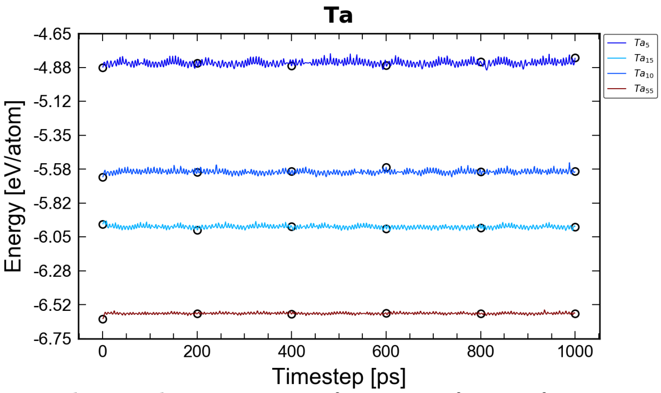

Supplementary Figure 243: The total energy as a function of time from exemplary MD simulations of Ta nanoclusters. Snapshots of MD simulation trajectories are provided in Supplementary Figure 242

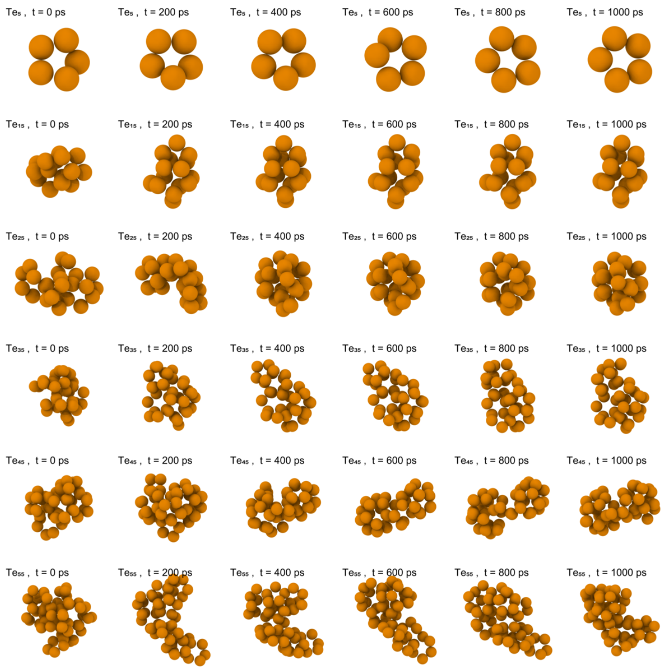

Supplementary Figure 244: MD simulation trajectories of Te Nanoclusters

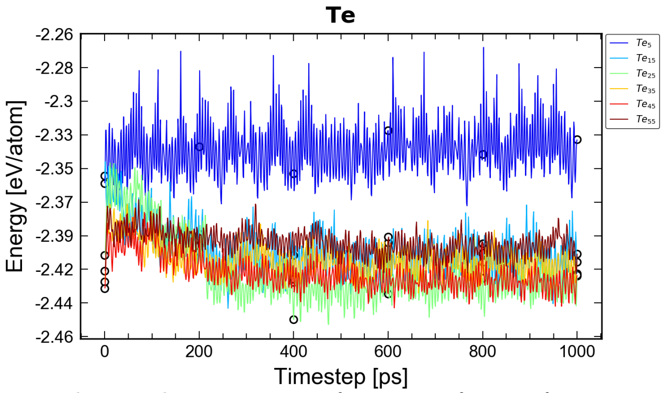

Supplementary Figure 245: The total energy as a function of time from exemplary MD simulations of Te nanoclusters. Snapshots of MD simulation trajectories are provided in Supplementary Figure 244

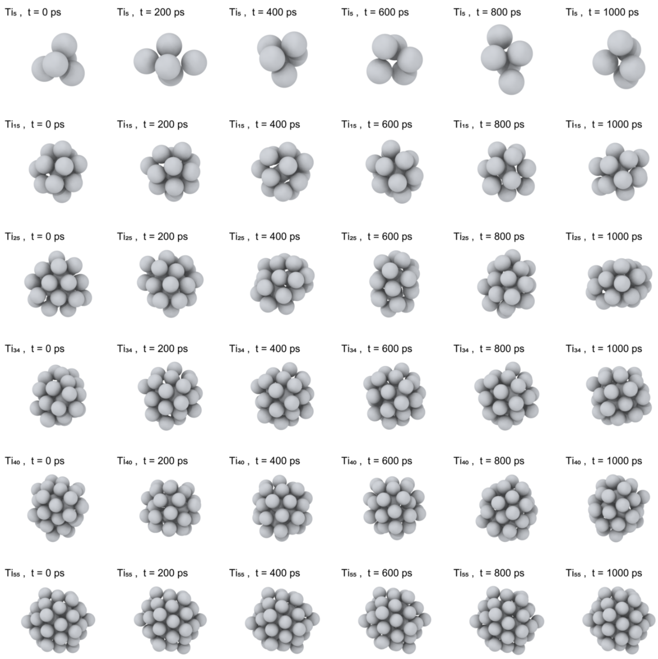

Supplementary Figure 246: MD simulation trajectories of Ti Nanoclusters

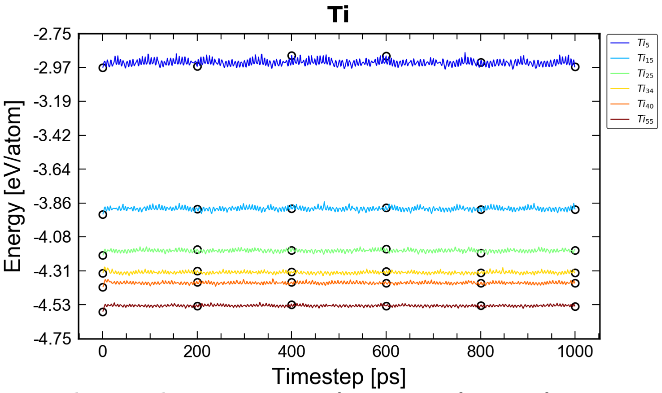

Supplementary Figure 247: The total energy as a function of time from exemplary MD simulations of Ti nanoclusters. Snapshots of MD simulation trajectories are provided in Supplementary Figure 246

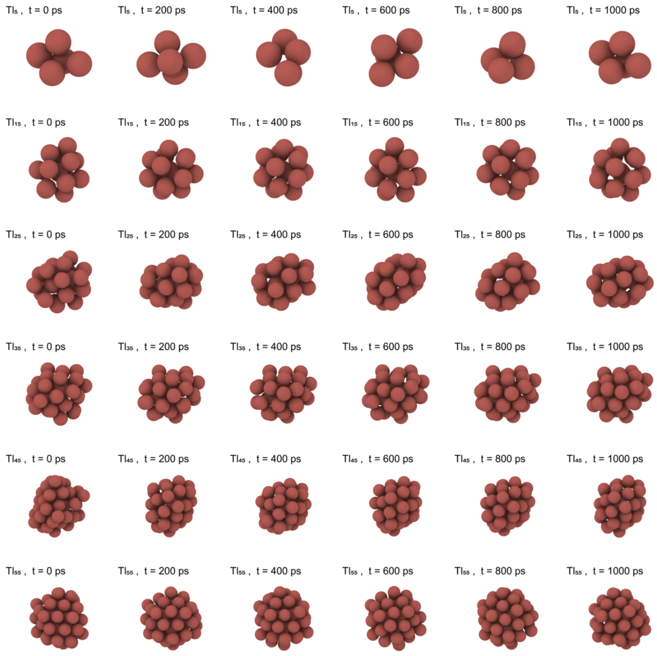

Supplementary Figure 248: MD simulation trajectories of Tl Nanoclusters

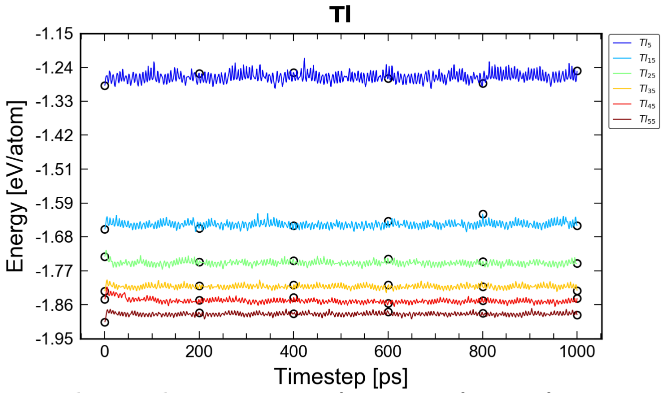

Supplementary Figure 249: The total energy as a function of time from exemplary MD simulations of Tl nanoclusters. Snapshots of MD simulation trajectories are provided in Supplementary Figure 248

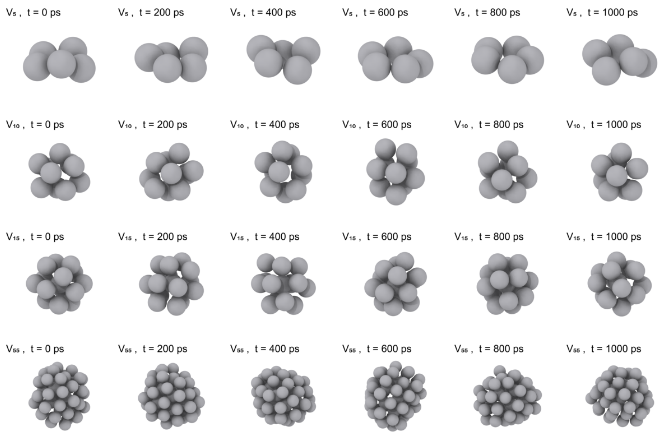

Supplementary Figure 250: MD simulation trajectories of V Nanoclusters

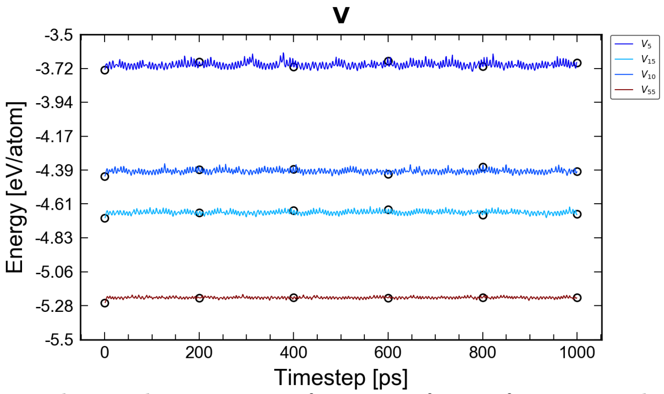

Supplementary Figure 251: The total energy as a function of time from exemplary MD simulations of V nanoclusters. Snapshots of MD simulation trajectories are provided in Supplementary Figure 250

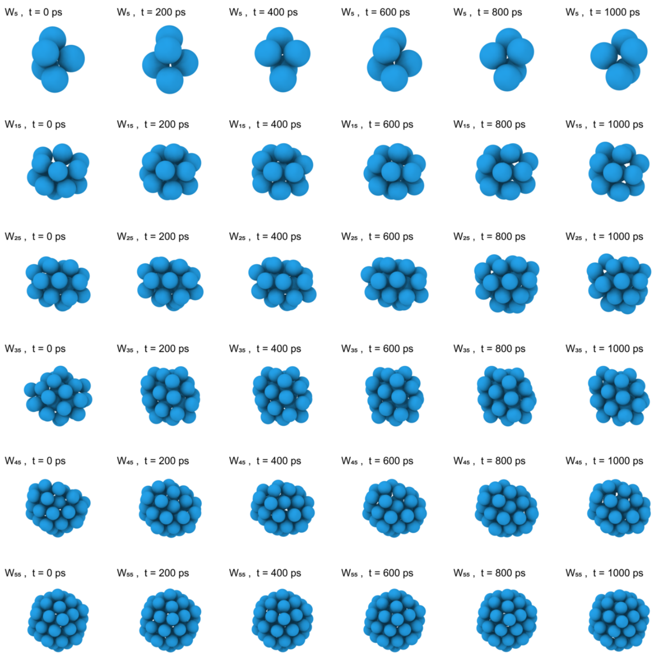

Supplementary Figure 252: MD simulation trajectories of W Nanoclusters

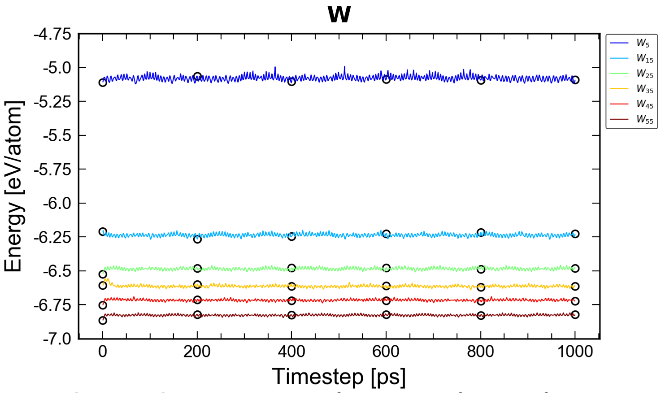

Supplementary Figure 253: The total energy as a function of time from exemplary MD simulations of W nanoclusters. Snapshots of MD simulation trajectories are provided in Supplementary Figure 252

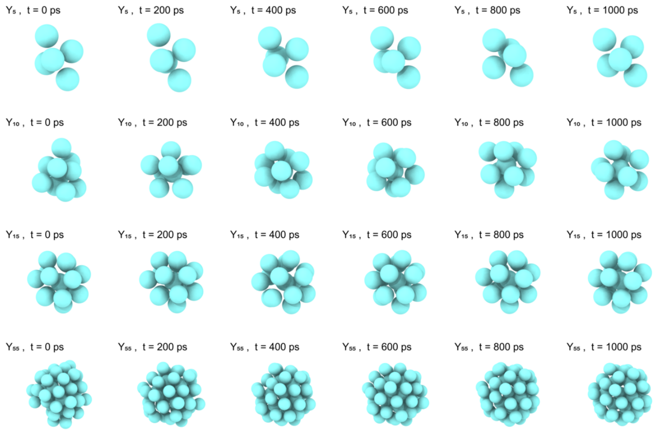

Supplementary Figure 254: MD simulation trajectories of Y Nanoclusters

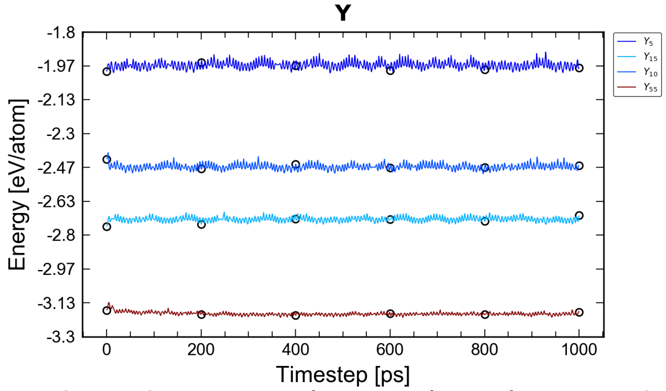

Supplementary Figure 255: The total energy as a function of time from exemplary MD simulations of Y nanoclusters. Snapshots of MD simulation trajectories are provided in Supplementary Figure 254

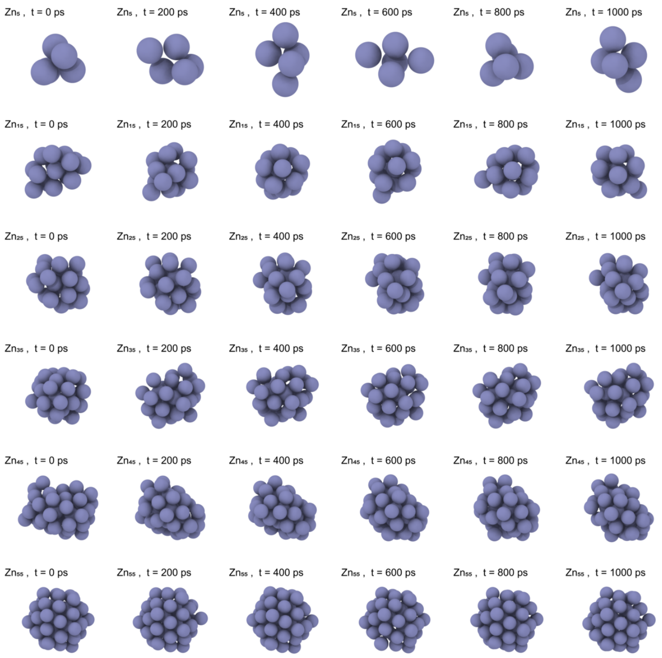

Supplementary Figure 256: MD simulation trajectories of Zn Nanoclusters

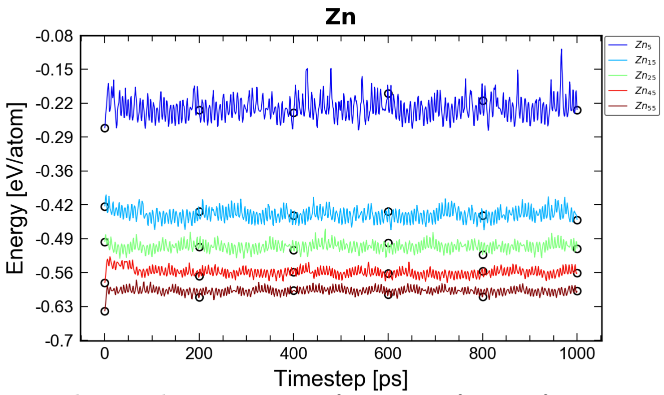

Supplementary Figure 257: The total energy as a function of time from exemplary MD simulations of Zn nanoclusters. Snapshots of MD simulation trajectories are provided in Supplementary Figure 256

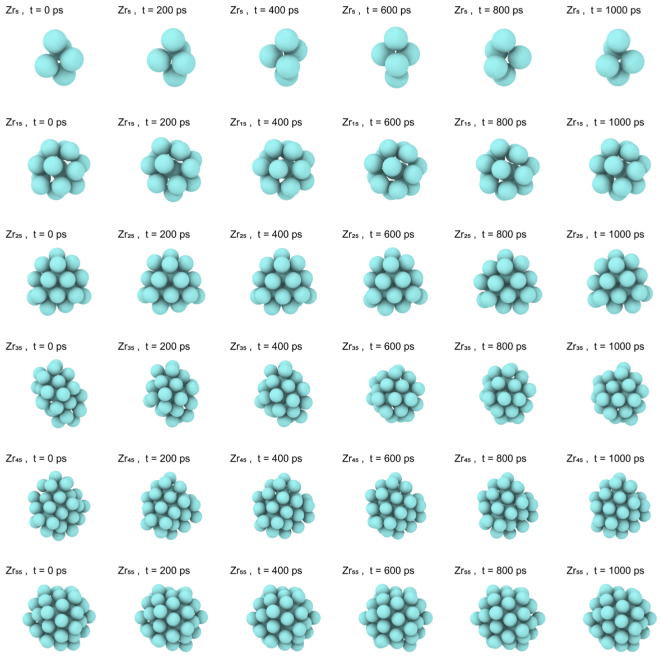

Supplementary Figure 258: MD simulation trajectories of Zr Nanoclusters

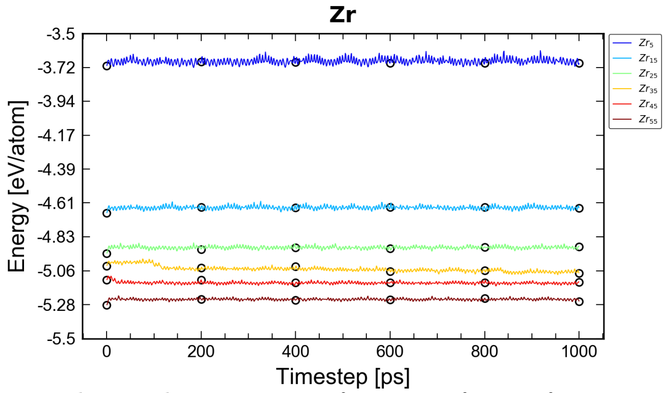

Supplementary Figure 259: The total energy as a function of time from exemplary MD simulations of Zr nanoclusters. Snapshots of MD simulation trajectories are provided in Supplementary Figure 258

11.4 Relative Stability Analysis

We first sort the isomers (P_i) of same cluster size based on DFT computed energies and calculated the relative energies $\Delta_{i,\ \mathrm{DFT}}$ w.r.t to lowest energy configuration of that particular size i.e. $E_{i,\ \mathrm{DFT}} - E_{min,\ \mathrm{DFT}}$. Next, we computed the energies using c-MCTS trained HyBOP on these sorted (based on the energy calculated using DFT) isomers. Similarly, We calculate the relative energies with HyBOP (i.e. $\Delta_{i,\ \mathrm{HyBOP}} = E_{i,\ \mathrm{HyBOP}} - E_{min,\ \mathrm{HyBOP}}$). Both these relative energies are plotted against their polymorphs as shown in Figs. 260-262.

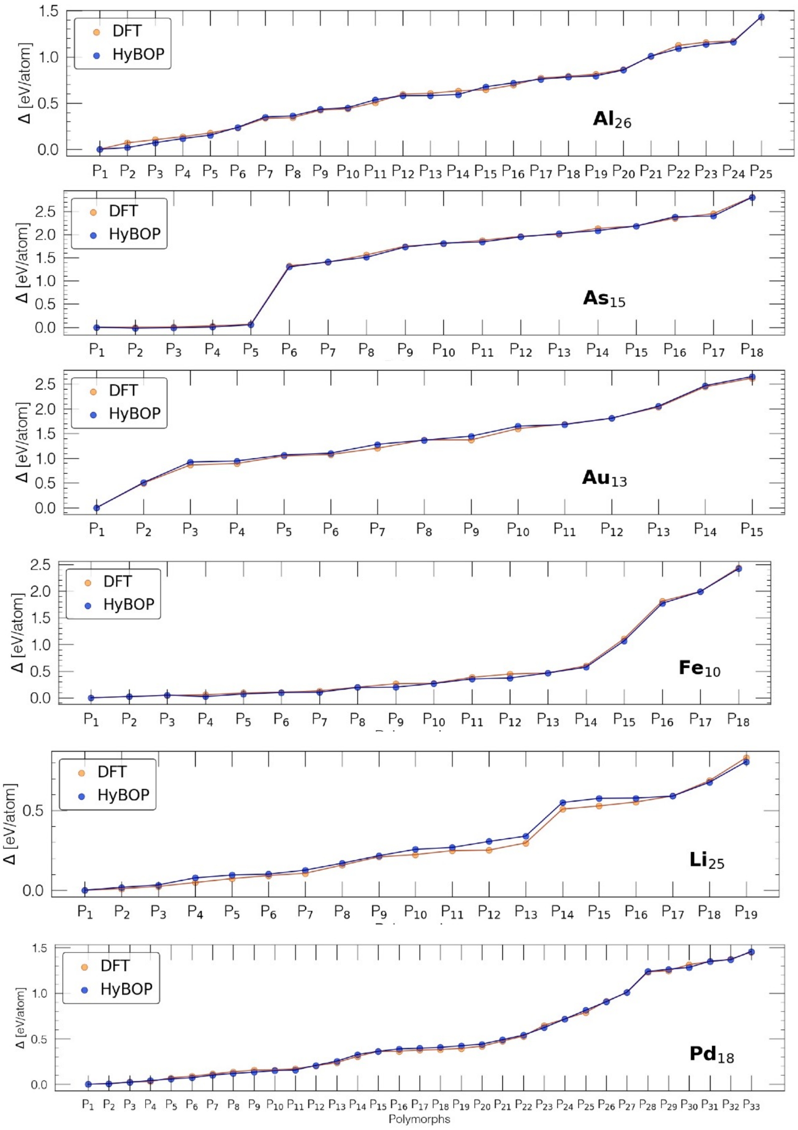

Supplementary Figure 260: Relative stability analysis for Al_{26}, As_{15}, Au_{13}, Fe_{10}, Li_{25}, and Pd_{18} clusters.

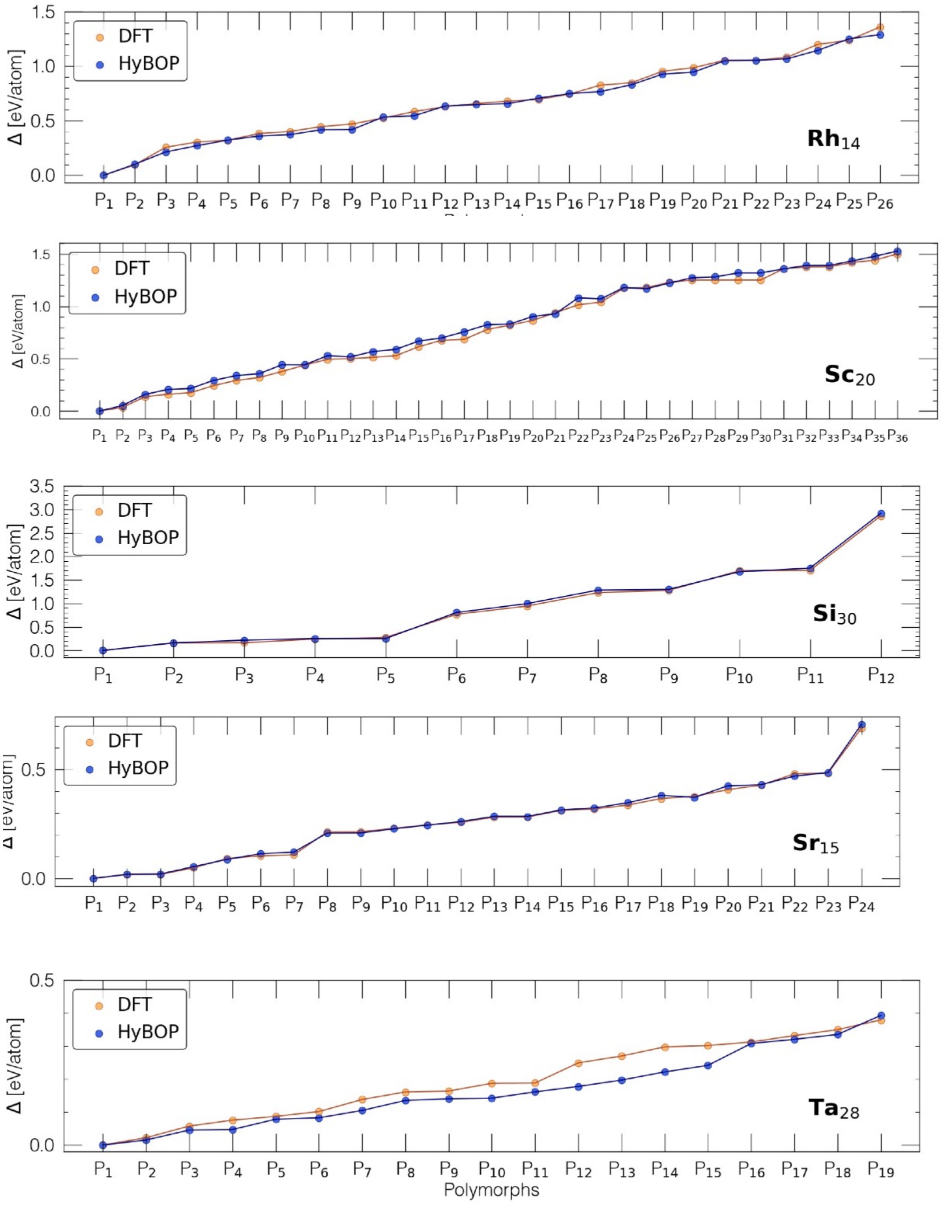

Supplementary Figure 261: Relative stability analysis for Rh_{14}, Sc_{20}, Si_{30}, Sr_{15}, and Ta_{28} clusters

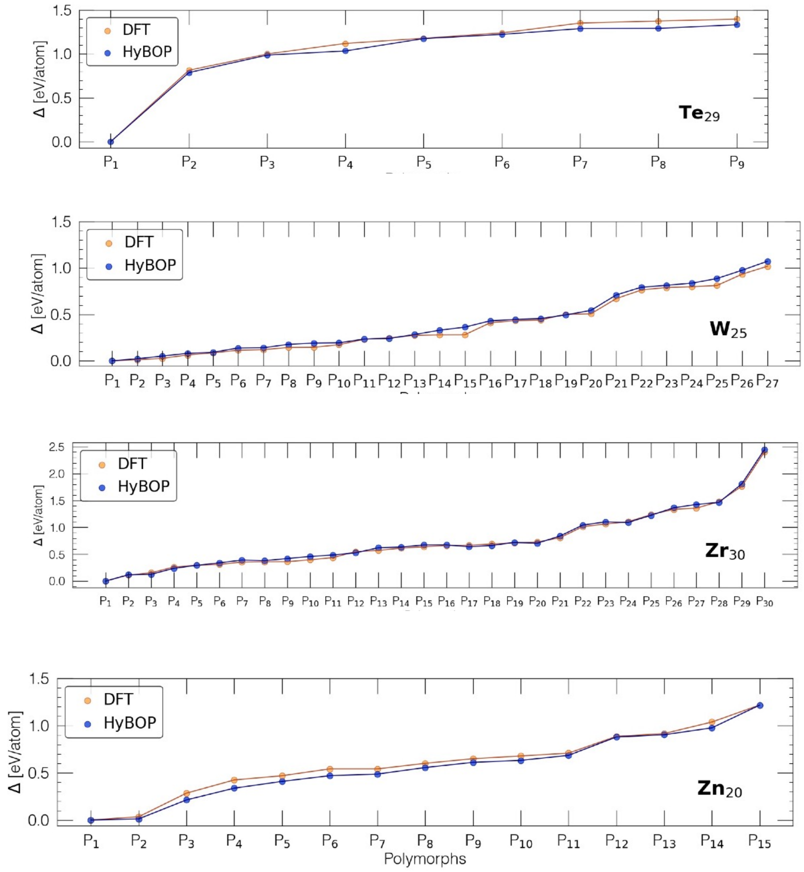

Supplementary Figure 262: Relative Stability for Te_{29}, W_{25}, Zr_{30}, and Zn_{20} clusters

12 Supplementary Note 8

12.1 Performance of c-MCTS trained High Dimensional Neural Network Potential

c-MCTS also can be used to train high-dimensional NN with » 1000's of parameters. A deep neural network (DNN) was used to learn the potential energy surface of different elemental clusters, including Al, C and Mo. It consisted of 2 fully connected (dense) hidden layers with 60 and 30 neurons, respectively. The last output layer had just one neuron, which signified the output (atomic) potential energy. Hyperbolic tangent (tanh) activation function was used in the hidden layers, while linear activation was used in the final output layer. The input layer consisted of atom centered symmetry functions (ASCF), which captured the atomic environment of each atom in the elemental clusters considered for model training. The ASCF fingerprints (53-dimensional) were obtained using Runner code with the parameter settings as provided here [123]. The Behler-Parrinello scheme was used to train the DNN, wherein the total energy of an elemental cluster is assumed to be sum of individual atomic energy contributions, which are obtained as the output of the DNN model. Two network structures and two target systems, Carbon and Aluminum, are evaluated for a total of four networks. Each network has a 10 node input layer and the first structure has a 30x30 hidden layer while the second has a 60x30 hidden layer. These result in thousands of network weights of 2581 and 5101 respectively.

The performance is on-par or better than the ADAM as implemented in Tensorflow. In particular, our c-MCTS optimization method has been been used to optimize the weights of neural network based potentials i.e. Behler–Parrinello type symmetry functions. The results are shown in Supplementary Table 10-11 and Supplementary Figure 263-264 and showcases that the c-MCTS scheme can be used to effortlessly train high-dimensional NN for capturing potential energy surface.

Supplementary Table 10: Summary table shows the performance of ADAM vs c-MCTS on predicting energies of Al, C, and Mo clusters

	MAE in ADAM trained NNP		MAE in c-MCTS trained NNP	
Element	Training Data (meV/atom)	Test Data (meV/atom)	Training Data (meV/atom)	Test Data (meV/atom)
Al	31.0	44.2	30.9	46.6
C	42.2	163.4	40.0	144.8
Mo	115.2	199.0	60.1	131.8

Supplementary Table 11: Performance of ADAM vs c-MCTS over the same training and test datasets: Tabulated MAEs for predicting energies and forces of the clusters of representative element Al.

	MAE in Al (meV/atom)	
Optimizer	Training	Test
ADAM	31.0	44.2
RuNNer	37.19	42.05
c-MCTS	30.9	46.6

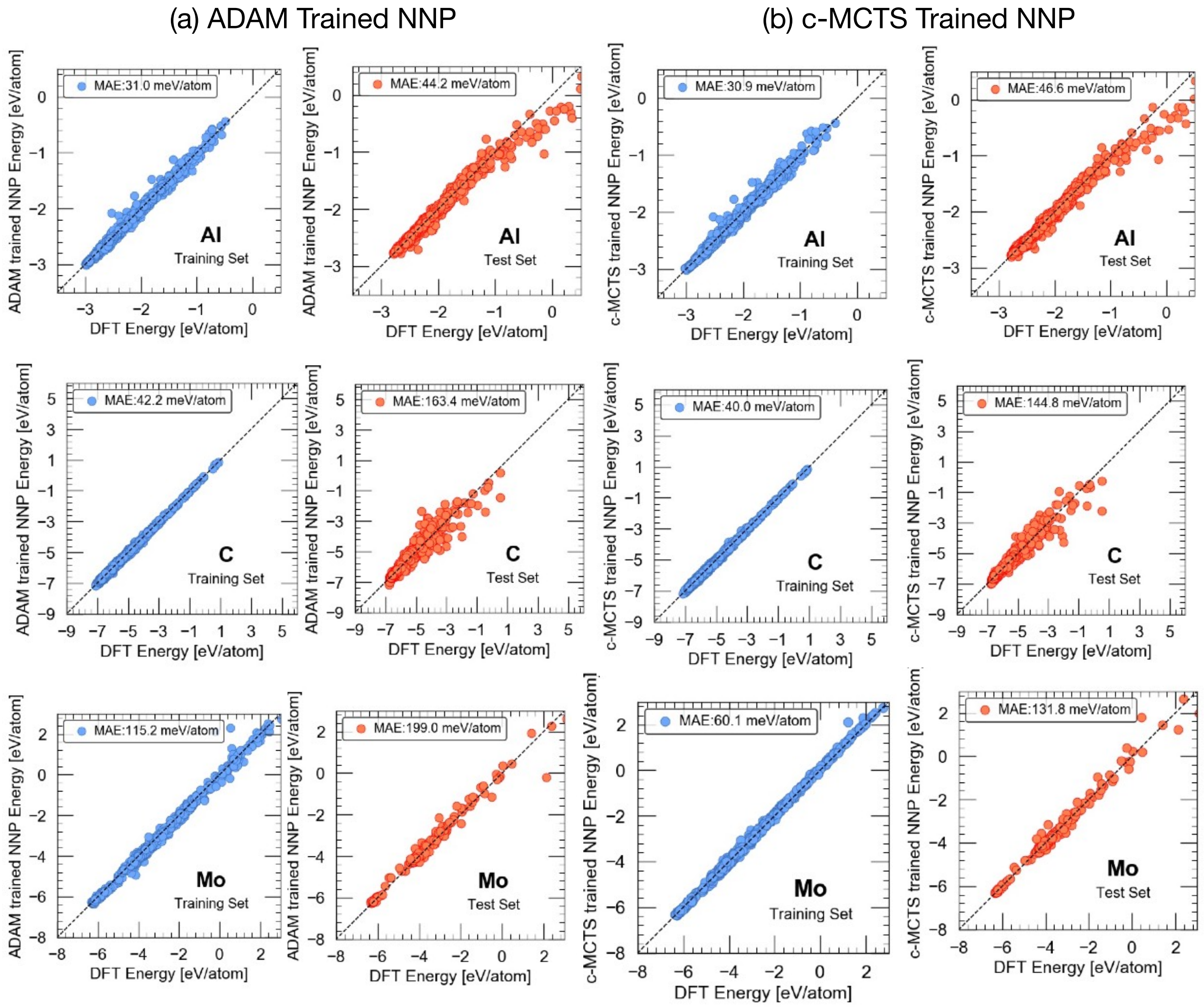

Supplementary Figure 263: Performance comparison between ADAM and c-MCTS trained NN potential for Al, C, and Mo clusters. We observe that c-MCTS trained NN potential performs consistently performs better on the Test data set for all these three elements.

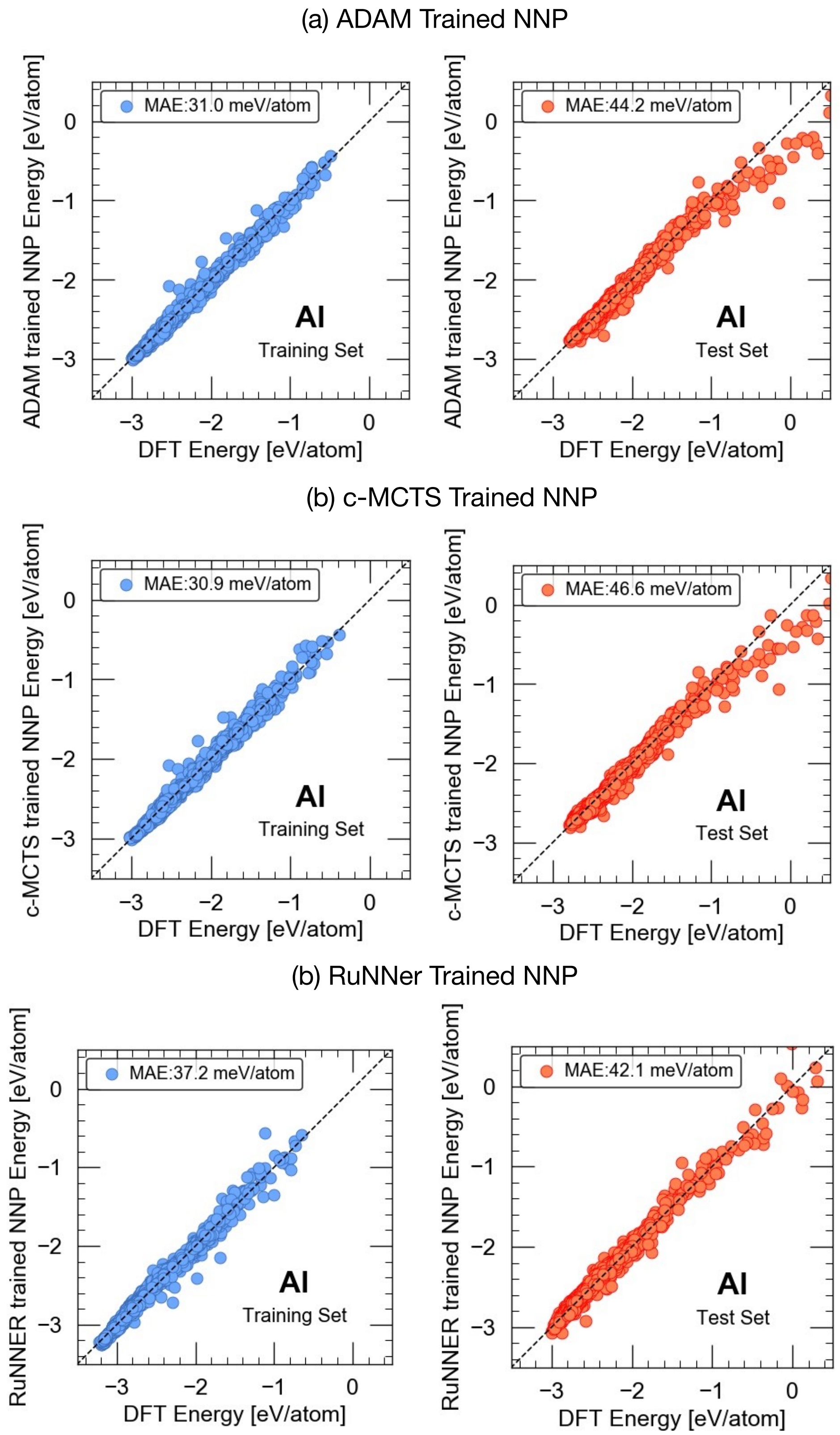

Supplementary Figure 264: Performance of ADAM (available from Tensorflow) trained NNP, c-MCTS (developed in this work) trained NNP, and RuNNer Code [124] (developed by Behler's group) trained NNP for predicting energies of Al clusters.

Supplementary References

[1] Chuanxi Wang, Lin Xu, Xiaowei Xu, Hao Cheng, Hongchen Sun, Quan Lin, and Chi Zhang. Near infrared ag/au alloy nanoclusters: tunable photoluminescence and cellular imaging. *Journal of colloid and interface science*, 416:274–279, 2014.

[2] JF Sanchez-Ramirez, U Pal, L Nolasco-Hernandez, J Mendoza-Alvarez, and JA Pescador-Rojas. Synthesis and optical properties of au-ag alloy nanoclusters with controlled composition. *Journal of Nanomaterials*, 2008, 2008.

[3] Sylvain Gelly, Levente Kocsis, Marc Schoenauer, Michele Sebag, David Silver, Csaba Szepesvári, and Olivier Teytaud. The grand challenge of computer go: Monte carlo tree search and extensions. *Communications of the ACM*, 55(3):106–113, 2012.

[4] Peter I Cowling, Edward J Powley, and Daniel Whitehouse. Information set monte carlo tree search. *IEEE Transactions on Computational Intelligence and AI in Games*, 4(2):120–143, 2012.

[5] David Silver, Julian Schrittwieser, Karen Simonyan, Ioannis Antonoglou, Aja Huang, Arthur Guez, Thomas Hubert, Lucas Baker, Matthew Lai, Adrian Bolton, et al. Mastering the game of go without human knowledge. *nature*, 550(7676):354–359, 2017.

[6] Boxin Tang. Orthogonal array-based latin hypercubes. *Journal of the American statistical association*, 88(424):1392–1397, 1993.

[7] Adrien Couëtoux, Jean-Baptiste Hoock, Nataliya Sokolovska, Olivier Teytaud, and Nicolas Bonnard. Continuous upper confidence trees. In *International Conference on Learning and Intelligent Optimization*, pages 433–445. Springer, 2011.

[8] Guillaume M JB Chaslot, Mark HM Winands, H JAAP VAN DEN HERIK, Jos WHM Uiterwijk, and Bruno Bouzy. Progressive strategies for monte-carlo tree search. *New Mathematics and Natural Computation*, 4(03):343–357, 2008.

[9] Jongmin Lee, Wonseok Jeon, Geon-Hyeong Kim, and Kee-Eung Kim. Monte-carlo tree search in continuous action spaces with value gradients. In *Proceedings of the AAAI Conference on Artificial Intelligence*, volume 34, pages 4561–4568, 2020.

[10] Momin Jamil and Xin-She Yang. A literature survey of benchmark functions for global optimisation problems. *International Journal of Mathematical Modelling and Numerical Optimisation*, 4(2):150–194, 2013.

[11] Test function and databases. https://www.sfu.ca/~ssurjano/optimization.html. Accessed: 2020-12-25.

[12] Elre Talea Oldewage et al. *The perils of particle swarm optimization in high dimensional problem spaces*. PhD thesis, University of Pretoria, 2017.

[13] Dongshu Wang, Dapei Tan, and Lei Liu. Particle swarm optimization algorithm: an overview. *Soft Computing*, 22(2):387–408, 2018.

[14] Bobak Shahriari, Kevin Swersky, Ziyu Wang, Ryan P Adams, and Nando De Freitas. Taking the human out of the loop: A review of bayesian optimization. *Proceedings of the IEEE*, 104(1):148–175, 2015.

[15] Daniel Russo, Benjamin Van Roy, Abbas Kazerouni, Ian Osband, and Zheng Wen. A tutorial on thompson sampling. *arXiv preprint arXiv:1707.02038*, 2017.

[16] Brian Rohr, Helge S Stein, Dan Guevarra, Yu Wang, Joel A Haber, Muratahan Aykol, Santosh K Suram, and John M Gregoire. Benchmarking the acceleration of materials discovery by sequential learning. *Chemical science*, 11(10):2696–2706, 2020.

[17] Rohit Batra, Le Song, and Rampi Ramprasad. Emerging materials intelligence ecosystems propelled by machine learning. *Nature Reviews Materials*, pages 1–24, 2020.

[18] Troy D. Loeffler, Sukriti Manna, Tarak K. Patra, Henry Chan, Badri Narayanan, and Subramanian Sankaranarayanan. Active learning a neural network model for gold clusters & bulk from sparse first principles training data. *ChemCatChem*, 12(19):4796–4806, 2020.

[19] Troy D Loeffler, Tarak K Patra, Henry Chan, Mathew Cherukara, and Subramanian KRS Sankaranarayanan. Active learning the potential energy landscape for water clusters from sparse training data. *The Journal of Physical Chemistry C*, 124(8):4907–4916, 2020.

[20] Troy D Loeffler, Tarak K Patra, Henry Chan, and Subramanian KRS Sankaranarayanan. Active learning a coarse-grained neural network model for bulk water from sparse training data. *Molecular Systems Design & Engineering*, 2020.

[21] Frank H Stillinger Jr. Rigorous basis of the frenkel-band theory of association equilibrium. *The Journal of Chemical Physics*, 38(7):1486–1494, 1963.

[22] The quantum cluster database. https://muellergroup.jhu.edu/qcd. Accessed: 2020-12-25.

[23] The cambridge cluster database. http://www-wales.ch.cam.ac.uk/CCD.html. Accessed: 2020-12-25.

[24] Prashun Gorai, Alex Ganose, Alireza Faghaninia, Anubhav Jain, and Vladan Stevanović. Computational discovery of promising new n-type dopable abx zintl thermoelectric materials. *Materials Horizons*, 2020.

[25] Romain Gautier, Xiuwen Zhang, Linhua Hu, Liping Yu, Yuyuan Lin, Tor OL Sunde, Danbee Chon, Kenneth R Poeppelmeier, and Alex Zunger. Prediction and accelerated laboratory discovery of previously unknown 18-electron abx compounds. *Nature chemistry*, 7(4):308, 2015.

[26] Benjamin C. Revard, William W. Tipton, and Richard G. Hennig. *Structure and Stability Prediction of Compounds with Evolutionary Algorithms*, pages 181–222. Springer International Publishing, Cham, 2014.

[27] Artem R Oganov, Andriy O Lyakhov, and Mario Valle. How evolutionary crystal structure prediction works and why. *Accounts of chemical research*, 44(3):227–237, 2011.

[28] Scott M Woodley and Richard Catlow. Crystal structure prediction from first principles. *Nature materials*, 7(12):937–946, 2008.

[29] Georg Kresse and Jürgen Furthmüller. Efficiency of ab-initio total energy calculations for metals and semiconductors using a plane-wave basis set. *Comput. Mater. Sci.*, 6(1):15–50, 1996.

[30] G. Kresse and J. Furthmüller. Efficient iterative schemes for ab initio total-energy calculations using a plane-wave basis set. *Phys. Rev. B*, 54:11169–11186, Oct 1996.

[31] Anubhav Jain, Shyue Ping Ong, Geoffroy Hautier, Wei Chen, William Davidson Richards, Stephen Dacek, Shreyas Cholia, Dan Gunter, David Skinner, Gerbrand Ceder, et al. Commentary: The materials project: A materials genome approach to accelerating materials innovation. *APL Mater.*, 1(1):011002, 2013.

[32] SKR Patil, SV Khare, Blair Richard Tuttle, JK Bording, and S Kodambaka. Mechanical stability of possible structures of ptn investigated using first-principles calculations. *Phys. Rev. B*, 73(10):104118, 2006.

[33] Sukriti Manna, Geoff L Brennecka, Vladan Stevanović, and Cristian V Ciobanu. Tuning the piezoelectric and mechanical properties of the aln system via alloying with yn and bn. *J. Appl. Phys.*, 122(10):105101, 2017.

[34] Dong Wu, Yachao Chen, Sukriti Manna, Kevin Talley, Andriy Zakutayev, Geoff L Brennecka, Cristian V Ciobanu, Paul Constantine, and Corinne E Packard. Characterization of elastic modulus across the $(Al_{1-x}Sc_x)$ n system using dft and substrate-effect-corrected nanoindentation. *IEEE Trans. Sonics Ultrason.*, 65(11):2167–2175, 2018.

[35] Sukriti Manna, Prashun Gorai, Geoff L Brennecka, Cristian V Ciobanu, and Vladan Stevanović. Large piezoelectric response of van der waals layered solids. *J. Mater. Chem. C*, 6(41):11035–11044, 2018.

[36] Sukriti Manna, Kevin R Talley, Prashun Gorai, John Mangum, Andriy Zakutayev, Geoff L Brennecka, Vladan Stevanović, and Cristian V Ciobanu. Enhanced piezoelectric response of aln via crn alloying. *Phys. Rev. Appl.*, 9(3):034026, 2018.

[37] Robert W McKinney, Prashun Gorai, Sukriti Manna, Eric Toberer, and Vladan Stevanović. Ionic vs. van der waals layered materials: identification and comparison of elastic anisotropy. *J. Mater. Chem. A*, 6(32):15828–15838, 2018.

[38] Maarten De Jong, Wei Chen, Thomas Angsten, Anubhav Jain, Randy Notestine, Anthony Gamst, Marcel Sluiter, Chaitanya Krishna Ande, Sybrand Van Der Zwaag, Jose J Plata, et al. Charting the complete elastic properties of inorganic crystalline compounds. *Scientific data*, 2(1):1–13, 2015.

[39] Soner Steiner, Sergii Khmelevskyi, Martijn Marsmann, and Georg Kresse. Calculation of the magnetic anisotropy with projected-augmented-wave methodology and the case study of disordered fe 1- x co x alloys. *Physical review B*, 93(22):224425, 2016.

[40] Mauricio J Piotrowski, Paulo Piquini, and Juarez LF Da Silva. Density functional theory investigation of 3 d, 4 d, and 5 d 13-atom metal clusters. *Physical Review B*, 81(15):155446, 2010.

[41] Mauricio J Piotrowski, Crina G Ungureanu, Polina Tereshchuk, Krys EA Batista, Anderson S Chaves, Diego Guedes-Sobrinho, and Juarez LF Da Silva. Theoretical study of the structural, energetic, and electronic properties of 55-atom metal nanoclusters: A dft investigation within van der waals corrections, spin–orbit coupling, and pbe+ u of 42 metal systems. *The Journal of Physical Chemistry C*, 120(50):28844–28856, 2016.

[42] Igor Ying Zhang and Andreas Grüneis. Coupled cluster theory in materials science. *Frontiers in Materials*, 6:123, 2019.

[43] Xuefei Xu, Wenjing Zhang, Mingsheng Tang, and Donald G Truhlar. Do practical standard coupled cluster calculations agree better than kohn–sham calculations with currently available functionals when compared to the best available experimental data for dissociation energies of bonds to 3 d transition metals? *Journal of chemical theory and computation*, 11(5):2036–2052, 2015.

[44] Chandler A Becker, Francesca Tavazza, Zachary T Trautt, and Robert A Buarque de Macedo. Considerations for choosing and using force fields and interatomic potentials in materials science and engineering. *Current Opinion in Solid State and Materials Science*, 17(6):277–283, 2013.

[45] Lucas M Hale, Zachary T Trautt, and Chandler A Becker. Evaluating variability with atomistic simulations: the effect of potential and calculation methodology on the modeling of lattice and elastic constants. *Modelling and Simulation in Materials Science and Engineering*, 26(5):055003, 2018.

[46] Murray S Daw and Michael I Baskes. Embedded-atom method: Derivation and application to impurities, surfaces, and other defects in metals. *Physical Review B*, 29(12):6443, 1984.

[47] Murray S Daw, Stephen M Foiles, and Michael I Baskes. The embedded-atom method: a review of theory and applications. *Materials Science Reports*, 9(7-8):251–310, 1993.

[48] MI Baskes. Application of the embedded-atom method to covalent materials: a semiempirical potential for silicon. *Physical review letters*, 59(23):2666, 1987.

[49] MI Baskes and RA Johnson. Modified embedded atom potentials for hcp metals. *Modelling and Simulation in Materials Science and Engineering*, 2(1):147, 1994.

[50] Michael I Baskes. Modified embedded-atom potentials for cubic materials and impurities. *Physical review B*, 46(5):2727, 1992.

[51] MJ Mishin. Mehl, and da papaconstantopoulos. *Acta Mater*, 53:4029, 2005.

[52] CV Singh and DH Warner. Mechanisms of guinier–preston zone hardening in the athermal limit. *Acta Materialia*, 58(17):5797–5805, 2010.

[53] Frank H Stillinger and Thomas A Weber. Computer simulation of local order in condensed phases of silicon. *Physical review B*, 31(8):5262, 1985.

[54] DG Pettifor and II Oleinik. Analytic bond-order potentials beyond tersoff-brenner. i. theory. *Physical review B*, 59(13):8487, 1999.

[55] DG Pettifor and II Oleinik. Bounded analytic bond-order potentials for σ and π bonds. *Physical review letters*, 84(18):4124, 2000.

[56] DA Murdick, XW Zhou, HNG Wadley, D Nguyen-Manh, R Drautz, and DG Pettifor. Analytic bond-order potential for the gallium arsenide system. *Physical Review B*, 73(4):045206, 2006.

[57] Donald K Ward, Xiao Wang Zhou, Bryan Matthew Wong, F Patrick Doty, and Jonathan A Zimmerman. Analytical bond-order potential for the cadmium telluride binary system. *Physical Review B*, 85(11):115206, 2012.

[58] Jerry Tersoff. Empirical interatomic potential for silicon with improved elastic properties. *Physical Review B*, 38(14):9902, 1988.

[59] J Nord, K Albe, P Erhart, and KJJoPCM Nordlund. Modelling of compound semiconductors: analytical bond-order potential for gallium, nitrogen and gallium nitride. *Journal of Physics: Condensed Matter*, 15(32):5649, 2003.

[60] GJ Ackland, G Tichy, V Vitek, and MW Finnis. Simple n-body potentials for the noble metals and nickel. *Philosophical Magazine A*, 56(6):735–756, 1987.

[61] PL Williams, Y Mishin, and JC Hamilton. An embedded-atom potential for the cu–ag system. *Modelling and Simulation in Materials Science and Engineering*, 14(5):817, 2006.

[62] AP Sutton and J Chen. Long-range finnis–sinclair potentials. *Philosophical Magazine Letters*, 61(3):139–146, 1990.

[63] Andrea Fortini, Mikhail I Mendelev, Sergey Buldyrev, and David Srolovitz. Asperity contacts at the nanoscale: Comparison of ru and au. *Journal of Applied Physics*, 104(7):074320, 2008.

[64] MI Mendelev, MJ Kramer, Chandler A Becker, and M Asta. Analysis of semi-empirical interatomic potentials appropriate for simulation of crystalline and liquid al and cu. *Philosophical Magazine*, 88(12):1723–1750, 2008.

[65] MI Pascuet and Julian Roberto Fernández. Atomic interaction of the meam type for the study of intermetallics in the al–u alloy. *Journal of Nuclear Materials*, 467:229–239, 2015.

[66] F Apostol and Y Mishin. Interatomic potential for the al-cu system. *Physical Review B*, 83(5):054116, 2011.

[67] Venkatesh Botu, Rohit Batra, James Chapman, and Rampi Ramprasad. Machine learning force fields: construction, validation, and outlook. *The Journal of Physical Chemistry C*, 121(1):511–522, 2017.

[68] Alan Nichol and Graeme J Ackland. Property trends in simple metals: An empirical potential approach. *Physical Review B*, 93(18):184101, 2016.

[69] XW Zhou, DK Ward, JE Martin, FB Van Swol, JL Cruz-Campa, and D Zubia. Stillinger-weber potential for the ii-vi elements zn-cd-hg-s-se-te. *Physical review B*, 88(8):085309, 2013.

[70] Karsten Albe, Kai Nordlund, Janne Nord, and Antti Kuronen. Modeling of compound semiconductors: Analytical bond-order potential for ga, as, and gaas. *Physical Review B*, 66(3):035205, 2002.

[71] XW Zhou, ME Foster, FB Van Swol, JE Martin, and Bryan M Wong. Analytical bond-order potential for the cd–te–se ternary system. *The Journal of Physical Chemistry C*, 118(35):20661–20679, 2014.

[72] Gang Wang, Yishuang Xu, Ping Qian, and Yanjing Su. Adp potential for the au-rh system and its application in element segregation of nanoparticles. *Computational Materials Science*, 186:110002, 2021.

[73] SR Wilson and MI Mendelev. A unified relation for the solid-liquid interface free energy of pure fcc, bcc, and hcp metals. *The Journal of Chemical Physics*, 144(14):144707, 2016.

[74] XW Zhou, RA Johnson, and HNG Wadley. Misfit-energy-increasing dislocations in vapor-deposited cofe/nife multilayers. *Physical Review B*, 69(14):144113, 2004.

[75] DE Smirnova, SV Starikov, and AM Vlasova. New interatomic potential for simulation of pure magnesium and magnesium hydrides. *Computational Materials Science*, 154:295–302, 2018.

[76] Jerry Tersoff. New empirical approach for the structure and energy of covalent systems. *Physical review B*, 37(12):6991, 1988.

[77] Joao F Justo, Martin Z Bazant, Efthimios Kaxiras, Vasily V Bulatov, and Sidney Yip. Interatomic potential for silicon defects and disordered phases. *Physical review B*, 58(5):2539, 1998.

[78] Priya Vashishta, Rajiv K Kalia, Aiichiro Nakano, and José Pedro Rino. Interaction potential for silicon carbide: A molecular dynamics study of elastic constants and vibrational density of states for crystalline and amorphous silicon carbide. *Journal of applied physics*, 101(10):103515, 2007.

[79] Yaojun A Du, Thomas J Lenosky, Richard G Hennig, Stefan Goedecker, and John W Wilkins. Energy landscape of silicon tetra-interstitials using an optimized classical potential. *physica status solidi (b)*, 248(9):2050–2055, 2011.

[80] GP Purja Pun and Y Mishin. Optimized interatomic potential for silicon and its application to thermal stability of silicene. *Physical Review B*, 95(22):224103, 2017.

[81] Alper Kınacı, Justin B Haskins, Cem Sevik, and Tahir Çağın. Thermal conductivity of bn-c nanostructures. *Physical Review B*, 86(11):115410, 2012.

[82] JH Los, JMH Kroes, K Albe, RM Gordillo, MI Katsnelson, and A Fasolino. Extended tersoff potential for boron nitride: Energetics and elastic properties of pristine and defective h-bn. *Physical Review B*, 96(18):184108, 2017.

[83] Anupriya Agrawal, Rohan Mishra, Logan Ward, Katharine M Flores, and Wolfgang Windl. An embedded atom method potential of beryllium. *Modelling and Simulation in Materials Science and Engineering*, 21(8):085001, 2013.

[84] Jesper Byggmästar, Etienne A Hodille, Y Ferro, and Kai Nordlund. Analytical bond order potential for simulations of beo 1d and 2d nanostructures and plasma-surface interactions. *Journal of Physics: Condensed Matter*, 30(13):135001, 2018.

[85] GJ Ackland and R Thetford. An improved n-body semi-empirical model for body-centred cubic transition metals. *Philosophical Magazine A*, 56(1):15–30, 1987.

[86] Hyoungki Park, Michael R Fellinger, Thomas J Lenosky, William W Tipton, Dallas R Trinkle, Sven P Rudin, Christopher Woodward, John W Wilkins, and Richard G Hennig. Ab initio based empirical potential used to study the mechanical properties of molybdenum. *Physical Review B*, 85(21):214121, 2012.

[87] R Ravelo, TC Germann, O Guerrero, Q An, and BL Holian. Shock-induced plasticity in tantalum single crystals: Interatomic potentials and large-scale molecular-dynamics simulations. *Physical Review B*, 88(13):134101, 2013.

[88] GP Purja Pun, KA Darling, LJ Kecskes, and Y Mishin. Angular-dependent interatomic potential for the cu–ta system and its application to structural stability of nano-crystalline alloys. *Acta Materialia*, 100:377–391, 2015.

[89] Steven J Stuart, Alan B Tutein, and Judith A Harrison. A reactive potential for hydrocarbons with intermolecular interactions. *The Journal of chemical physics*, 112(14):6472–6486, 2000.

[90] JH Los and A Fasolino. Intrinsic long-range bond-order potential for carbon: Performance in monte carlo simulations of graphitization. *Physical Review B*, 68(2):024107, 2003.

[91] XW Zhou, Donald K Ward, and Michael E Foster. An analytical bond-order potential for carbon. *Journal of computational chemistry*, 36(23):1719–1735, 2015.

[92] Thomas C O'Connor, Jan Andzelm, and Mark O Robbins. Airebo-m: A reactive model for hydrocarbons at extreme pressures. *The Journal of chemical physics*, 142(2):024903, 2015.

[93] Y Zhang, R Ashcraft, MI Mendelev, CZ Wang, and KF Kelton. Experimental and molecular dynamics simulation study of structure of liquid and amorphous ni62nb38 alloy. *The Journal of chemical physics*, 145(20):204505, 2016.

[94] Michael R Fellinger, Hyoungki Park, and John W Wilkins. Force-matched embedded-atom method potential for niobium. *Physical Review B*, 81(14):144119, 2010.

[95] DE Smirnova and SV Starikov. An interatomic potential for simulation of zr-nb system. *Computational Materials Science*, 129:259–272, 2017.

[96] RG Hennig, TJ Lenosky, DR Trinkle, SP Rudin, and JW Wilkins. Classical potential describes martensitic phase transformations between the α, β, and ω titanium phases. *Physical Review B*, 78(5):054121, 2008.

[97] MI Mendelev, TL Underwood, and GJ Ackland. Development of an interatomic potential for the simulation of defects, plasticity, and phase transformations in titanium. *The Journal of chemical physics*, 145(15):154102, 2016.

[98] GP Purja Pun and Y Mishin. Embedded-atom potential for hcp and fcc cobalt. *Physical Review B*, 86(13):134116, 2012.

[99] Seungwu Han, Luis A Zepeda-Ruiz, Graeme J Ackland, Roberto Car, and David J Srolovitz. Interatomic potential for vanadium suitable for radiation damage simulations. *Journal of applied physics*, 93(6):3328–3335, 2003.

[100] Pär AT Olsson. Semi-empirical atomistic study of point defect properties in bcc transition metals. *Computational materials science*, 47(1):135–145, 2009.

[101] CA Howells and Y Mishin. Angular-dependent interatomic potential for the binary ni–cr system. *Modelling and Simulation in Materials Science and Engineering*, 26(8):085008, 2018.

[102] Diana Farkas and Alfredo Caro. Model interatomic potentials for fe–ni–cr–co–al high-entropy alloys. *Journal of Materials Research*, pages 1–10, 2020.

[103] GJ Ackland, MI Mendelev, DJ Srolovitz, S Han, and AV Barashev. Development of an interatomic potential for phosphorus impurities in α-iron. *Journal of Physics: Condensed Matter*, 16(27):S2629, 2004.

[104] Mihai-Cosmin Marinica, Lisa Ventelon, MR Gilbert, L Proville, SL Dudarev, J Marian, G Bencteux, and F Willaime. Interatomic potentials for modelling radiation defects and dislocations in tungsten. *Journal of Physics: Condensed Matter*, 25(39):395502, 2013.

[105] Kun Wang, Wenjun Zhu, Meizhen Xiang, Yun Xu, Guomeng Li, and Jun Chen. Improved embedded-atom model potentials of pb at high pressure: application to investigations of plasticity and phase transition under extreme conditions. *Modelling and Simulation in Materials Science and Engineering*, 27(1):015001, 2018.

[106] MI Mendelev and Alexander H King. The interactions of self-interstitials with twin boundaries. *Philosophical Magazine*, 93(10-12):1268–1278, 2013.

[107] GJ Ackland, SJ Wooding, and DJ Bacon. Defect, surface and displacement-threshold properties of α-zirconium simulated with a many-body potential. *Philosophical Magazine A*, 71(3):553–565, 1995.

[108] Laurent Proville, David Rodney, and Mihai-Cosmin Marinica. Quantum effect on thermally activated glide of dislocations. *Nature materials*, 11(10):845–849, 2012.

[109] S Alireza Etesami and Ebrahim Asadi. Molecular dynamics for near melting temperatures simulations of metals using modified embedded-atom method. *Journal of Physics and Chemistry of Solids*, 112:61–72, 2018.

[110] Jesper Byggmästar and Fredric Granberg. Dynamical stability of radiation-induced c15 clusters in iron. *Journal of Nuclear Materials*, 528:151893, 2020.

[111] A Béré and Anna Serra. On the atomic structures, mobility and interactions of extended defects in gan: dislocations, tilt and twin boundaries. *Philosophical Magazine*, 86(15):2159–2192, 2006.

[112] Sayyed Jalil Mahdizadeh and Golnoosh Akhlamadi. Optimized tersoff empirical potential for germanene. *Journal of Molecular Graphics and Modelling*, 72:1–5, 2017.

[113] JJPRB Tersoff. Modeling solid-state chemistry: Interatomic potentials for multicomponent systems. *Physical review B*, 39(8):5566, 1989.

[114] Henry Chan, Mathew J Cherukara, Badri Narayanan, Troy D Loeffler, Chris Benmore, Stephen K Gray, and Subramanian KRS Sankaranarayanan. Machine learning coarse grained models for water. *Nature communications*, 10(1):1–14, 2019.

[115] SM Foiles, MI Baskes, and Murray S Daw. Embedded-atom-method functions for the fcc metals cu, ag, au, ni, pd, pt, and their alloys. *Physical review B*, 33(12):7983, 1986.

[116] Badri Narayanan, Alper Kinaci, Fatih G Sen, Michael J Davis, Stephen K Gray, Maria KY Chan, and Subramanian KRS Sankaranarayanan. Describing the diverse geometries of gold from nanoclusters to bulk : A first-principles-based hybrid bond-order potential. *The Journal of Physical Chemistry C*, 120(25):13787–13800, 2016.

[117] John A Keith, Donato Fantauzzi, Timo Jacob, and Adri CT Van Duin. Reactive forcefield for simulating gold surfaces and nanoparticles. *Physical Review B*, 81(23):235404, 2010.

[118] Sara Kadkhodaei and Axel van de Walle. A simple local expression for the prefactor in transition state theory. *The Journal of chemical physics*, 150(14):144105, 2019.

[119] Anthony B. Costa. A compute module partialhessian that calculates the hessian matrix for a group of atoms during a lammps simulation. https://bitbucket.org/numericalsolutions/lammps-hessian/src/master/, 2014.

[120] Georg Kresse and Jürgen Furthmüller. Efficient iterative schemes for ab initio total-energy calculations using a plane-wave basis set. *Physical review B*, 54(16):11169, 1996.

[121] Georg Kresse and J Hafner. Ab initio molecular dynamics for open-shell transition metals. *Physical Review B*, 48(17):13115, 1993.

[122] Steve Plimpton. Fast parallel algorithms for short-range molecular dynamics. *Journal of computational physics*, 117(1):1–19, 1995.

[123] Nongnuch Artrith and Jörg Behler. High-dimensional neural network potentials for metal surfaces: A prototype study for copper. *Physical Review B*, 85(4):045439, 2012.

[124] J Behler. Runner-a neural network code for high-dimensional potential-energy surfaces, georg-august universität göttingen, 2018.
