# Supplementary material for: Learning in continuous action space for developing high dimensional potential energy models
Source: Nat Commun. 2022 Jan 18;13:368. doi: 10.1038/s41467-021-27849-6 (PMC8766468; doi:10.1038/s41467-021-27849-6)
Supplement: Supplementary file 3 — Description of Additional Supplementary Files [file 41467_2021_27849_MOESM3_ESM.pdf]

Description of Additional Supplementary Files

File Name: Supplementary Data 1

Description: c-MCTS optimized parameters for all 54 elements based on the Hybrid Bond-Order Potential formalism. While all 19 parameters are included here, we note that the parameter m was kept constant during the c-MCTS

File Name: Supplementary Movie 1

Description: Video attachments for Continuous Mountain Car (MountainCarContinuous-v0).
Here, a neural network with hyperbolic tangent activation functions was trained using c-MCTS. A neural network with a node structure of 10X10X5 for a total of three hidden layers was used for both environments. The inputs and outputs of the network were sized to match the number of observable and number of controls in both cases. The reward was given by the OpenAI environment [1]. A single set of network parameters was tested on 5 random start positions and ran till the finished condition was returned by the OpenAI interface. No other information about the system apart from what was directly supplied by the default environment binding was given to the network.

File Name: Supplementary Movie 2

Description: Video attachments for Continuous Lunar Lander (LunarLanderContinuous-v2). Here, a neural network with hyperbolic tangent activation functions was trained using c-MCTS. A neural network with a node structure of 10X10X5 for a total of three hidden layers was used for both environments. The inputs and outputs of the network were sized to match the number of observable and number of controls in both cases. The reward was given by the OpenAI environment [1]. A single set of network parameters was tested on 5 random start positions and ran till the finished condition was returned by the OpenAI interface. No other information about the system apart from what was directly supplied by the default environment binding was given to the network.

[1] Brockman, Greg, Vicki Cheung, Ludwig Pettersson, Jonas Schneider, John Schulman, Jie Tang, and Wojciech Zaremba. "Openai gym." arXiv preprint arXiv:1606.01540 (2016).

File Name: Supplementary Movie 3

Description: Video attachment for sampling of different non-equilibrium configurations of Carbon.

File Name: Supplementary Software 1

Description: The NN potentials developed in this study and the other available MLIP methods such as GAP, SNAP, qSNAP, and MEGNET used for benchmarking (https://doi.org/10.5281/zenodo.5655543).
