# Supplementary material for: Learning in continuous action space for developing high dimensional potential energy models
Source: Nat Commun. 2022 Jan 18;13:368. doi: 10.1038/s41467-021-27849-6 (PMC8766468; doi:10.1038/s41467-021-27849-6)
Supplement: Supplementary file 8 — Supplementary Software 1 [file 41467_2021_27849_MOESM8_ESM.zip › Supplementary Softwares/KIM potentials.pdf]

The other available MLIP methods such as SNAP, qSNAP used for benchmarking are provided below:

Cu

SNAP

DOI 10.1021/acs.jpca.9b08723

Extended KIM ID
SNAP_ZuoChenLi_2019_Cu__MO_931672895580_000

KIM page
https://openkim.org/id/SNAP_ZuoChenLi_2019_Cu__MO_931672895580_000

q-SNAP

DOI: 10.1021/acs.jpca.9b08723.

Extended KIM ID
SNAP_ZuoChenLi_2019quadratic_Cu__MO_265210066873_000

KIM page
https://openkim.org/id/SNAP_ZuoChenLi_2019quadratic_Cu__MO_265210066873_000

Ge

SNAP

DOI: 10.1021/acs.jpca.9b08723.

Extended KIM ID
SNAP_ZuoChenLi_2019_Ge__MO_183216355174_000

KIM page
https://openkim.org/id/SNAP_ZuoChenLi_2019_Ge__MO_183216355174_000

q-SNAP

DOI: 10.1021/acs.jpca.9b08723.

Extended KIM ID
SNAP_ZuoChenLi_2019quadratic_Ge__MO_766484508139_000

KIM page
https://openkim.org/id/SNAP_ZuoChenLi_2019quadratic_Ge__MO_766484508139_000

Li

SNAP

DOI: 10.1021/acs.jpca.9b08723.

Extended KIM ID
SNAP_ZuoChenLi_2019_Li__MO_732106099012_000

KIM page
https://openkim.org/id/SNAP_ZuoChenLi_2019_Li__MO_732106099012_000

q-SNAP

DOI: 10.1021/acs.jpca.9b08723.

Extended KIM ID
SNAP_ZuoChenLi_2019quadratic_Li__MO_041269750353_000

KIM page
https://openkim.org/id/SNAP_ZuoChenLi_2019quadratic_Li__MO_041269750353_000

Mo

SNAP

DOI: 10.1021/acs.jpca.9b08723.

Extended KIM ID
SNAP_ZuoChenLi_2019_Mo__MO_014123846623_000

KIM page
https://openkim.org/id/SNAP_ZuoChenLi_2019_Mo__MO_014123846623_000

q-SNAP

DOI: 10.1021/acs.jpca.9b08723.

Extended KIM ID
SNAP_ZuoChenLi_2019_Mo__MO_014123846623_000

KIM page
https://openkim.org/id/SNAP_ZuoChenLi_2019quadratic_Mo__MO_692442138123_000

Ni

SNAP

DOI: 10.1021/acs.jpca.9b08723.

Extended KIM ID
SNAP_ZuoChenLi_2019_Ni__MO_365106510449_000

KIM page
https://openkim.org/id/SNAP_ZuoChenLi_2019_Ni__MO_365106510449_000

q-SNAP

DOI: 10.1021/acs.jpca.9b08723.

Extended KIM ID
SNAP_ZuoChenLi_2019quadratic_Ni__MO_263593395744_000

KIM page
https://openkim.org/id/SNAP_ZuoChenLi_2019quadratic_Ni__MO_263593395744_000

Si

SNAP

DOI: 10.1021/acs.jpca.9b08723.

Extended KIM ID
SNAP_ZuoChenLi_2019_Si__MO_869330304805_000

KIM page
https://openkim.org/id/SNAP_ZuoChenLi_2019_Si__MO_869330304805_000

q-SNAP

DOI: 10.1021/acs.jpca.9b08723.

Extended KIM ID
SNAP_ZuoChenLi_2019quadratic_Si__MO_721469752060_000

KIM page
https://openkim.org/id/SNAP_ZuoChenLi_2019quadratic_Si__MO_721469752060_000
